# Deep-sequencing transcriptome analysis of chilling tolerance mechanisms of a subnival alpine plant, *Chorispora bungeana*

**DOI:** 10.1186/1471-2229-12-222

**Published:** 2012-11-21

**Authors:** Zhiguang Zhao, Lingling Tan, Chunyan Dang, Hua Zhang, Qingbai Wu, Lizhe An

**Affiliations:** 1 Key Laboratory of Cell Activities and Stress Adaptations, Ministry of Education, School of Life Sciences, Lanzhou University, Lanzhou, 730000, China; 2 State Key Laboratory of Frozen Soil Engineering, Cold and Arid Regions Environmental and Engineering Research Institute, Chinese Academy of Sciences, Lanzhou, 730000, China

**Keywords:** Alpine plant, *Chorispora bungeana*, Chilling tolerance, Cold acclimation, Transcriptome

## Abstract

**Background:**

The plant tolerance mechanisms to low temperature have been studied extensively in the model plant Arabidopsis at the transcriptional level. However, few studies were carried out in plants with strong inherited cold tolerance. *Chorispora bungeana* is a subnival alpine plant possessing strong cold tolerance mechanisms. To get a deeper insight into its cold tolerance mechanisms, the transcriptome profiles of chilling-treated *C. bungeana* seedlings were analyzed by Illumina deep-sequencing and compared with Arabidopsis.

**Results:**

Two cDNA libraries constructed from mRNAs of control and chilling-treated seedlings were sequenced by Illumina technology. A total of 54,870 unigenes were obtained by *de novo* assembly, and 3,484 chilling up-regulated and 4,571 down-regulated unigenes were identified. The expressions of 18 out of top 20 up-regulated unigenes were confirmed by qPCR analysis. Functional network analysis of the up-regulated genes revealed some common biological processes, including cold responses, and molecular functions in *C. bungeana* and Arabidopsis responding to chilling. Karrikins were found as new plant growth regulators involved in chilling responses of *C. bungeana* and Arabidopsis. However, genes involved in cold acclimation were enriched in chilling up-regulated genes in Arabidopsis but not in *C. bungeana.* In addition, although transcription activations were stimulated in both *C. bungeana* and Arabidopsis, no *CBF* putative ortholog was up-regulated in *C. bungeana* while *CBF2* and *CBF3* were chilling up-regulated in Arabidopsis. On the other hand, up-regulated genes related to protein phosphorylation and auto-ubiquitination processes were over-represented in *C. bungeana* but not in Arabidopsis.

**Conclusions:**

We conducted the first deep-sequencing transcriptome profiling and chilling stress regulatory network analysis of *C. bungeana*, a subnival alpine plant with inherited cold tolerance. Comparative transcriptome analysis suggests that cold acclimation is not a major chilling tolerance mechanism of *C. bungeana*. Activation of protein phosphorylation and ubiquitination may confer chilling tolerance to *C. bungeana* in a more rapid and flexible way than cold acclimation. Such differences may have contributed to the differences in cold tolerance between *C. bungeana* and Arabidopsis. The results presented in this paper will be informative for gene discovery and the molecular mechanisms related to plant cold tolerance.

## Background

*Chorispora bungeana* Fisch. & C.A. Mey (*C. bungeana*) is a perennial subnival alpine plant that can survive freezing temperature [1]. In the natural environments where *C. bungeana* is growing (origin of Urumqi River in Tianshan Mountains, Xinjiang Autonomous Region, China), snowing and hailing often occur during favorable growing seasons, and air temperature fluctuates frequently ranging from above 20°C to below −10°C. *C. bungeana* in local environment can survive, grow and flower even in snow. Our previous studies performed at physiological and molecular levels showed that this plant has strong cold (chilling and freezing) tolerance [1-6]. However, little is known about its tolerance mechanisms, if any, distinguishing *C. bungeana* from other tropical or temperate plants.

Not all plants are always ready to tolerate freezing temperatures. However, studies have shown many plants are tolerant of freezing temperature after exposure to non-freezing low temperature, a phenomenon called cold acclimation [7,8]. In such a process, various physiological and biochemical changes occur in plant cells, which may confer subsequent acquired chilling and freezing tolerance to plants. For example, during cold acclimation, plants accumulate compatible solutes such as sucrose, raffinose and proline [9-12]; membrane compositions and behaviors are changed [13-16]; and the biosynthesis pathways of secondary metabolites such as flavonoids are activated [17,18].

The physiological and biochemical changes during plant cold acclimation result mainly from expression changes of cold-responsive (COR) genes. A large number of studies demonstrate that gene expression changes occur in a wide range of plant species in cold responses, and it is believed that differences in COR gene expressions contribute to differences in plant cold tolerance. For example, considerable differences in the members of COR genes were found in *Solanum commersonii* and *Solanum tuberosum*, which are closely related species that differ in cold acclimation abilities [19].

The expressions of COR genes in plant cold responses are under the control of some key transcription factors (TFs). The best characterized TFs involved in plant cold responses are a class of AP2/EFR TFs known as DREB/CBF [20-23], which regulate COR gene expressions by binding to the DRE/CRT cis-elements in the promoter regions of COR genes. In Arabidopsis, there are three major CBFs - CBF1, CBF2 and CBF3 (also known as DREB1b, DREB1c, and DREB1a, respectively) [24]. Constitutive expression of CBF1 and CBF3 can enhance freezing tolerance in non-acclimated Arabidopsis [25]. Moreover, by studying the interactions with CBFs pathway, the roles of some cellular or environmental factors, such as calcium [26], light [27], and circadian rhythm [28], in plant cold tolerance are revealed. Nonetheless, CBFs may not represent all TFs that regulate the expressions of COR genes and confer cold tolerance to plants. Although CBF over-expression increases the freezing tolerance of Arabidopsis, potato [29] and poplar [30], it does not increase the freezing tolerance of tomato [31] and rice [32]. Besides CBFs, some other TFs, such as ZAT12 and RAV1 [33,34], are also discovered to regulate the expressions of COR genes.

Given the importance of COR genes in plant cold tolerance, studying the cold responses at transcription level may be a key step to identify specific tolerance mechanisms of plants. During the last two decades, numerous studies were carried out to reveal the transcriptional regulatory network of plants in response to cold stress. However, most of the studies were performed with Arabidopsis and others were conducted with crops such as *Brassica napus*[35]*,* rice [36], barley [37] and potato [19]. Some studies were performed with species adapted to arctic or alpine cold environments, such as Draba [38,39] and Oxytropis [40], suggesting that plants may adapt to cold environments with different strategies and COR genes. However, due to lack of reference genome sequence, such studies are relatively few. Sequencing the genome of *Coccomyxa subellipsoidea* from the Antarctic suggested that gene losses and gains may contribute to low temperature adaptations [41], highlighting the importance of studying cold tolerance at whole genome or transcriptome level. Recently, the development of high-throughput deep-sequencing technologies makes it possible to study gene expressions at whole genome level without prior knowledge about reference genome sequence. In this study, we used Illumina deep-sequencing technology to study the transcriptome profiles of chilling-treated seedlings of *C. bungeana*.

*C. bungeana* is a Cruciferae species closely related to Arabidopsis. Our previous studies showed that the callus and suspension cells from *C. bungeana* were ready to endure freezing temperature (−4°C) without cold acclimation [3,6]. The aim of this study is to examine what kinds of mechanisms contribute to the specific cold tolerance of *C. bungeana*. Our results showed a complicated regulatory network of *C. bungeana* responding to chilling stress. By comparative transcriptome analysis, a large number of common chilling responding processes, including a newly found karrikins responding process, were found in both *C. bungeana* and Arabidopsis. Furthermore, our results implied the differences between *C. bungeana* and Arabidopsis in cold acclimation and TF regulation networks. Importantly, our results suggested that protein phosphorylation and ubiquitination might serve as rapid and flexible mechanisms for cold tolerance regulations in *C. bungeana*.

## Results and discussion

### Sequencing and de novo assembly of C. bungeana transcriptome

Two cDNA libraries were generated with mRNA from control (22°C) or 24 hours chilling-treated (2°C) plants of *C. bungeana* and sequenced by Illumina deep-sequencing. 41,499,576 and 40,009,694 clean reads of 90 bp were generated from control and chilling-treated cDNA libraries, respectively (Table 1). *De novo* assembly was carried out by Trinity method [42] and final unigenes were obtained by TGICL clustering [43]. Overviews of the assembly results were shown in Table 2. The sequence reads were finally assembled into 54,870 non-redundant unigenes, spanning a total of 48.7 Mb of sequence. All unigenes were longer than 200 bp. Mean length of final unigenes was 888 bp and N50 was 1401 bp. With the Trinity *de novo* assembly method, no N remained in the final unigenes. We also tried *de novo* assembly with SOAPdenovo program [44]. However, the assembly quality was worse than that of the Trinity method, with a mean length of 596 bp and N50 of 809 bp, and 13.9% of the final unigenes had at least one N remained (Table 3). The results were similar to the transcriptome assembly report of *Aegilops variabilis*[45], in which the assembly qualities of the Trinity method were superior to that of the SOAPdenovo method. Therefore, the assembly results from the Trinity method were used for all the following analysis.

**Table 1:** Statistics of deep-sequencing

**Sample**	**Total reads**	**Total nucleotides (nt)**	**Q20 percentage**	**N percentage**	**GC percentage**
Control	41,499,576	3,734,961,840	95.44%	0.01%	47.48%
Cold-stressed	40,009,694	3,600,872,460	95.92%	0.00%	47.55%

**Table 2:** Statistics of the assembly (unigene number and percentage) with the Trinity method

	**Control**	**Cold-stressed**	**Combined**
200-500nt	21,064 (45.52%)	26,284 (51.97%)	25,233 (45.99%)
500-1000nt	11,421 (24.68%)	12,215 (24.15%)	12,746 (23.23%)
1000-1500nt	6,190 (13.38%)	5,811 (11.49%)	7,290 (13.29%)
1500-2000nt	3,651 (7.89%)	3,193 (6.31%)	4,458 (8.12%)
> = 2000nt	3,946 (8.53%)	3,071 (6.07%)	5,143 (9.37%)
N50	1,335	1,136	1,401
Mean	868	754	888
All Unigene	46,272	50,574	54,870
Length of all Unigene (nt)	40,180,147	38,132,636	48,708,039

**Table 3:** Statistics of the assembly (unigene number and percentage) with the SOAPdenovo software

	**Control**	**Cold-stressed**	**Combined**
100-500nt	48701 (72.6%)	57007 (77.46%)	39728 (62.99%)
500-1000nt	12066 (17.99%)	11880 (16.14%)	14121 (22.39%)
1000-1500nt	3539 (5.28%)	2987 (4.06%)	4897 (7.76%)
1500-2000nt	1479 (2.2%)	1054 (1.43%)	2220 (3.52%)
> = 2000nt	1296 (1.93%)	663 (0.9%)	2108 (3.34%)
N50	634	502	809
Mean	474	413	596
All Unigene	67,081	73,591	63,074
Length of all Unigene (nt)	31,789,071	30,382,210	37,575,882

### Functional annotation of all the unigenes of C. bungeana

**Figure 1:**
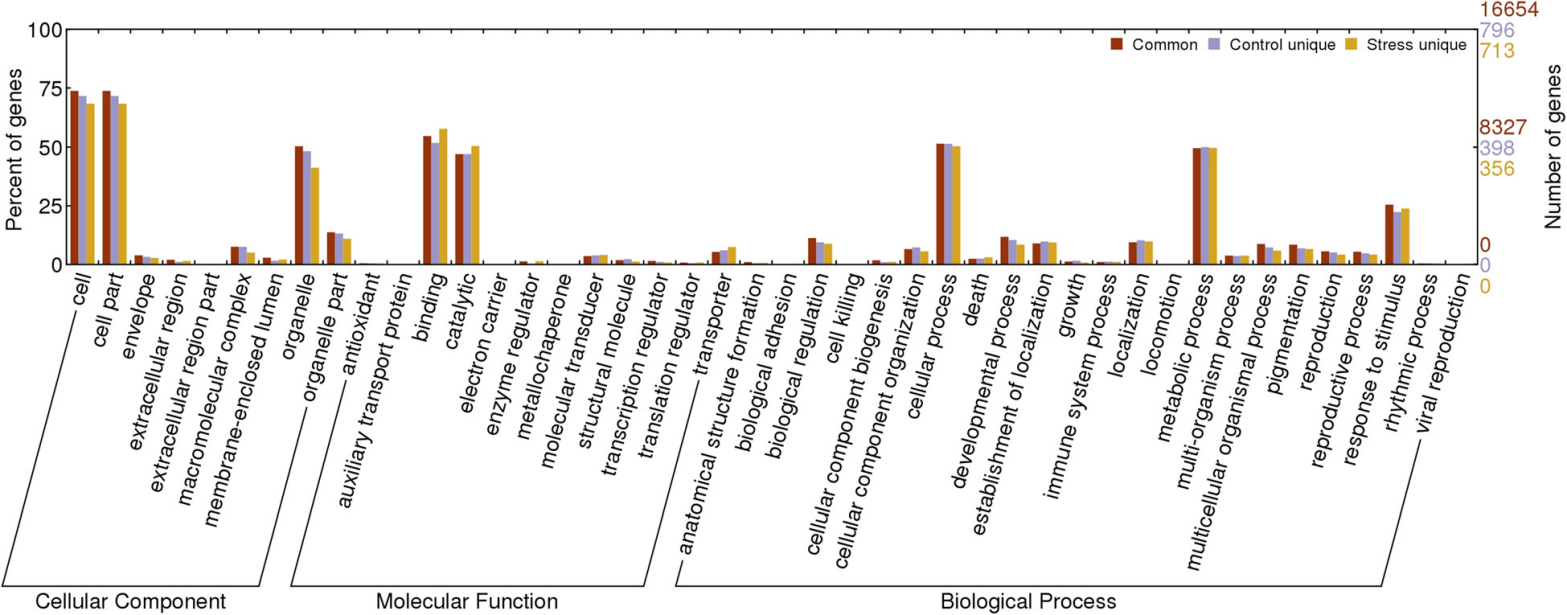
**Functional classifications of GO terms of all*C. bungeana*unigenes.**

Functions of the unigenes were annotated based on sequence similarities to sequences in the three public databases (NR, Swissprot and KEGG). Among the 54,870 non-redundant unigenes, 43,524 (79.4%) had at least one hit in BLASTX search with E-value < =1e-5 (Additional file 1). Functional classifications of GO terms of all unigenes were shown in Figure 1. In the category of biological process, the largest groups were “cellular process”, “metabolic process” and “response to stimulus”. In the category of molecular function, unigenes with “binding” and “catalytic” activities were the largest groups.

### Expression analysis, differential expression genes (DEGs) identification and qPCR verifications

The expressions of unigenes were analyzed with DEGseq R package. Firstly, we tried to identify DEGs by applying screening thresholds of 2 fold changes and Benjamini *q* value <0.001. We got 12,808 DEG candidates out of 52,753 expressed unigenes (Additional file 2). However, when we verified the expressions of the top 10 up-regulated and down-regulated unigenes by RT-PCR and qPCR, only 3 of them were amplified and none of them showed up or down-regulated trends in chilling-treated seedlings (data not shown). In addition, we found that 80% and 90% of the top 200 up and down-regulated unigenes presented only in one sample’s RNA-seq data, respectively. PCR amplification failures of the selected sequences suggested that such genes were most likely to be the artifacts of *de novo* assembly.

To identify DEGs accurately, we dropped off all unigenes with RPKM < 1 in both sequencing libraries before DEGseq analysis. By this method, 8,055 DEGs (25.7%; 3,484 up-regulated, 4,571 down-regulated) out of 31,295 unigenes with minimal 1.0 RPKM in both cDNA samples were identified (Additional file 3). The top 50 most up- or down-regulated unigenes were listed in Table 4 and Table 5, respectively. A number of genes involved in cold or other stresses showed up in the top 50 up-regulated list, such as putative orthologous genes (POGs) of *COR15A*, *ABR1*, pectin methylesterase inhibitor gene, *MAPKKK13*, heat shock transcription factor *A1E* and *LTI65* genes. A putative ortholog of Arabidopsis *COR15A*, which encodes a cryoprotective protein located to the chloroplast stroma [46], was identified as the most up-regulated unigene in *C. bungeana*.

**Table 4:** **Top 50 up-regulated unigenes of*C. bungeana*by chilling stress. The homologs of Arabidopsis genes were presented for functional description of unigenes**

**Unigene**	**log2 (Fold change)**	**AGI**	**Functional description**
CBT13817	7.47	AT2G42540	cold-regulated 15a (COR15A)
CBT52238	6.67	-	-
CBT6902	6.32	AT5G64750	ABA REPRESSOR1 (ABR1)
CBT7920	6.13	-	-
CBT13614	6.10	AT5G63450	cytochrome P450, family 94, subfamily B, polypeptide 1 (CYP94B1)
CBT4773	6.10	AT5G62360	Plant invertase/pectin methylesterase inhibitor superfamily protein
CBT52823	6.06	-	-
CBT22908	5.91	AT1G22810	Integrase-type DNA-binding superfamily protein
CBT13319	5.51	AT1G07150	mitogen-activated protein kinase kinase kinase 13 (MAPKKK13)
CBT47699	5.46	-	-
CBT15934	5.40	AT2G38240	2-oxoglutarate (2OG) and Fe(II)-dependent oxygenase superfamily protein
CBT47787	5.37	-	-
CBT47948	5.36	AT5G65140	Haloacid dehalogenase-like hydrolase (HAD) superfamily protein
CBT11719	5.25	AT5G17460	unknown protein
CBT25137	5.25	AT1G19670	chlorophyllase 1 (CLH1)
CBT22504	5.19	AT5G63450	cytochrome P450, family 94, subfamily B, polypeptide 1 (CYP94B1)
CBT45404	5.13	-	-
CBT19519	5.08	-	-
CBT1251	5.06	AT3G02990	heat shock transcription factor A1E (HSFA1E)
CBT22708	5.06	AT5G45860	PYR1-like 11 (PYL11)
CBT22442	5.05	AT4G34131	UDP-glucosyl transferase 73B3 (UGT73B3)
CBT28264	4.97	AT4G01870	tolB protein-related
CBT26921	4.97	AT1G11925	Stigma-specific Stig1 family protein
CBT11679	4.93	AT1G02400	gibberellin 2-oxidase 6 (GA2OX6)
CBT14428	4.92	AT3G06490	myb domain protein 108 (MYB108)
CBT19682	4.91	AT5G38780	S-adenosyl-L-methionine-dependent methyltransferases superfamily protein
CBT34609	4.90	-	-
CBT47700	4.89	-	-
CBT48057	4.83	-	-
CBT6326	4.79	AT3G04010	O-Glycosyl hydrolases family 17 protein
CBT17469	4.79	AT4G14690	EARLY LIGHT-INDUCIBLE PROTEIN 2 (ELIP2)
CBT47754	4.76	AT1G25220	anthranilate synthase beta subunit 1 (ASB1)
CBT1612	4.74	AT5G52300	LOW-TEMPERATURE-INDUCED 65 (LTI65)
CBT29537	4.73	AT2G33710	Integrase-type DNA-binding superfamily protein
CBT3801	4.68	AT1G57990	purine permease 18 (PUP18)
CBT17111	4.64	AT5G67600	unknown protein
CBT8371	4.61	AT2G46950	cytochrome P450, family 709, subfamily B, polypeptide 2 (CYP709B2)
CBT21020	4.61	-	-
CBT28985	4.59	AT1G65690	Late embryogenesis abundant (LEA) hydroxyproline-rich glycoprotein family
CBT336	4.59	AT3G24900	receptor like protein 39 (RLP39)
CBT845	4.57	AT2G34930	disease resistance family protein / LRR family protein
CBT18111	4.55	AT1G05530	UDP-glucosyl transferase 75B2 (UGT75B2)
CBT45699	4.55	-	-
CBT9147	4.52	-	-
CBT37125	4.51	-	-
CBT47516	4.50	AT2G43840	UDP-glycosyltransferase 74 F1 (UGT74F1)
CBT7419	4.50	AT1G64380	Integrase-type DNA-binding superfamily protein
CBT51514	4.47	-	-
CBT2368	4.46	-	-
CBT7125	4.44	AT1G26390	FAD-binding Berberine family protein

**Table 5:** **Top 50 down-regulated unigenes of*C. bungeana*by chilling stress. The homologs of Arabidopsis genes were presented for functional description of unigenes**

**Unigene**	**log2(fold change)**	**AGI**	**Computational_description**
CBT30334	−3.99	AT2G14660.1	unknown protein
CBT13943	−3.76	-	-
CBT2874	−3.74	-	-
CBT48038	−3.46	-	-
CBT3212	−3.44	-	-
CBT34638	−3.28	-	-
CBT8991	−3.27	-	-
CBT19662	−3.25	AT3G06145.1	unknown protein
CBT30596	−3.21	AT5G06950.4	AHBP-1B
CBT22861	−3.04	-	-
CBT7674	−3.02	-	-
CBT7734	−2.98	AT5G62280.1	Protein of unknown function (DUF1442)
CBT3347	−2.98	-	-
CBT31902	−2.94	-	-
CBT17505	−2.91	AT3G06740.1	GATA transcription factor 15 (GATA15)
CBT24596	−2.90	-	-
CBT15245	−2.90	-	-
CBT27066	−2.90	-	-
CBT2813	−2.89	AT4G20270.1	BARELY ANY MERISTEM 3 (BAM3)
CBT39819	−2.88	-	-
CBT2069	−2.87	-	-
CBT26600	−2.85	AT3G17668.1	ENHANCER OF ATNSI ACTIVITY (ENA)
CBT7789	−2.83	-	-
CBT7793	−2.83	AT3G48970.1	Heavy metal transport/detoxification superfamily protein
CBT38584	−2.82	AT3G60910.1	S-adenosyl-L-methionine-dependent methyltransferases superfamily protein
CBT5244	−2.81	AT3G52905.1	Polynucleotidyl transferase, ribonuclease H-like superfamily protein
CBT26673	−2.80	-	-
CBT27159	−2.78	AT5G55540.1	TORNADO 1 (TRN1)
CBT15242	−2.75	-	-
CBT13738	−2.73	AT3G54560.1	histone H2A 11 (HTA11)
CBT38583	−2.70	-	-
CBT31492	−2.69	-	-
CBT38499	−2.69	-	-
CBT29663	−2.68	-	-
CBT6741	−2.68	-	-
CBT30636	−2.67	-	-
CBT26681	−2.66	-	-
CBT23205	−2.65	AT5G54550.1	Protein of unknown function (DUF295)
CBT4916	−2.64	AT5G26860.1	lon protease 1 (LON1)
CBT2774	−2.63	-	-
CBT40023	−2.63	-	-
CBT3096	−2.62	AT1G80080.1	TOO MANY MOUTHS (TMM)
CBT39489	−2.62	-	-
CBT30184	−2.62	-	-
CBT3289	−2.62	AT1G03270.1	CBS domain-containing protein with a domain of unknown function (DUF21)
CBT30641	−2.61	-	-
CBT34154	−2.61	-	-
CBT34041	−2.60	-	-
CBT39400	−2.59	AT3G01690.1	alpha/beta-Hydrolases superfamily protein
CBT23404	−2.59	-	-

**Figure 2:**
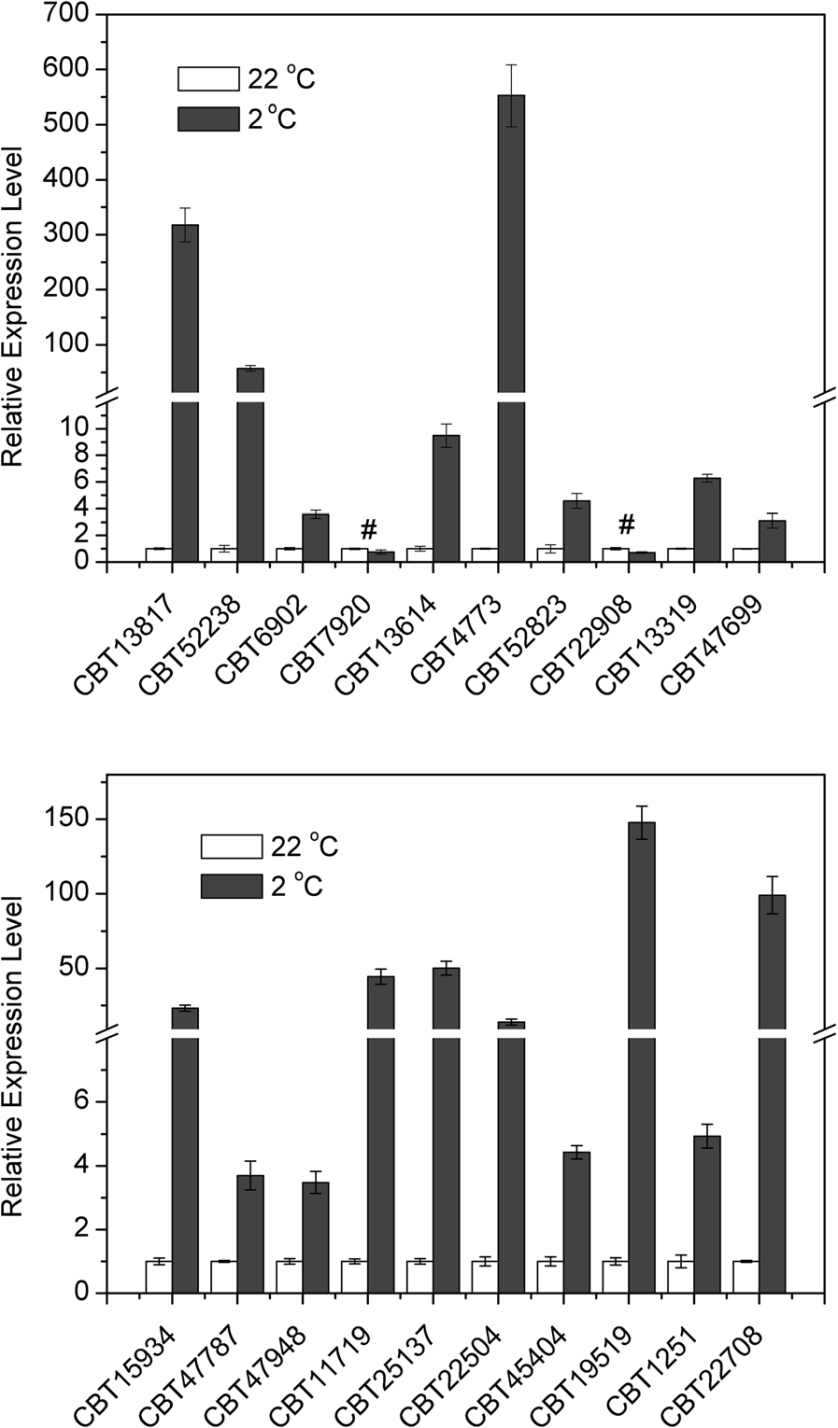
Expression analysis of top 20 up-regulated DEGs by qPCR.

The top 20 up-regulated DEGs were selected to verify the expressions of the indentified DEGs by qPCR analysis. To get more reliable quantification results, we performed an experiment in advance to screen reference genes for qPCR (see Methods for details), and the relative expression levels of unigenes were normalized to 3 stable expressed reference genes. The results showed that 18 of the top 20 up-regulated DEGs (90%) were verified to be up-regulated by qPCR analysis, although their fold changes differed from that of RNA-seq (Figure 2). Except for *CBT7920* and *CBT22908*, the expressions of all other tested unigenes showed at least 3-fold increases after 24-hour chilling treatment. The most up-regulated unigene were POGs encoded a plant invertase/pectin methylesterase inhibitor superfamily protein (*CBT4773*, 552 folds). *COR15A* (*CBT13817*, 318 folds) was also induced remarkably by chilling.

High throughput deep-sequencing is a powerful tool for DEGs screening, especially for species without available genomic information [45,47,48]. However, since Illumina sequencing is highly sensitive to templates presented in DNA samples, some traced transcripts or contaminants can be sequenced in one sample but not in other samples. This will have huge effects on the results of *de novo* assembly and increase false positive rate in DEGs identification. One strategy to reduce the false positive results is to set up biological repeats for sequencing and increase sequencing depth, but it will greatly increase the experimental costs. In this study, by simply applying an additional threshold (RPKM > =1) for DEGs screening without increasing costs, we got a high quality (confirmed by qPCR) list of chilling regulated DEGs.

### GO network analysis of up-regulated DEGs of C. bungeana in response to chilling stress and comparison with Arabidopsis

Since both *C. bungeana* and Arabidopsis are Cruciferae species, it is more reliable to use the well-established GO and KEGG annotation systems of Arabidopsis to analyze the functions of *C. bungeana* DEGs. GO term and KEGG pathway enrichment analysis of DEGs were conducted with BiNGO [49], a Cytoscape plugin assessing overrepresentation of ontologies in biological networks, using the list of all unigenes with a minimal RPKM of 1 in both sequencing libraries as a reference set. To compare the chilling responding network of *C. bungeana* with Arabidopsis, the networks of chilling-regulated DEGs of Arabidopsis were constructed using previously published RNA-seq and microarray data (referred to ATH-SR and ATH-MA, respectively; see Methods for details).

**Figure 3:**
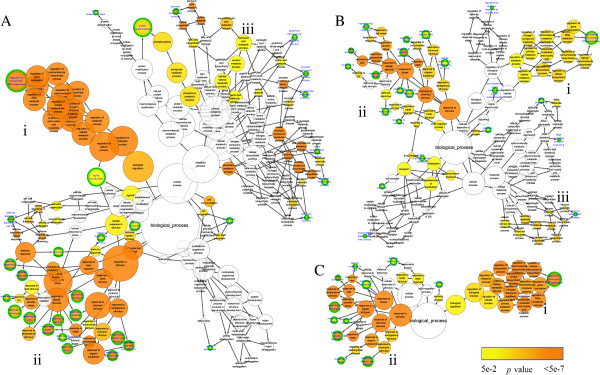
**Biological process network of over-representative GO terms of chilling up-regulated DEGs. A**, *C. bungeana*; **B**, ATH-SR; **C**, ATH-MA. Node size represented gene number in node and node filled color represented *p* value. White nodes were not significant over-representative terms. End nodes were indicated by green border and blue label. (**i**) cluster of “regulation processes”; (**ii**) cluster of “stimulus responses”; (**iii**) cluster of “metabolism processes”.

In chilling up-regulated DEGs of *C. bungeana* and Arabidopsis, two similar clusters in the networks of GO biological process, “regulation processes” and “stimulus responses”, were found among all three networks/datasets (Figure 3). In BiNGO constructed networks, most biological information can be inferred from end nodes and their relations with their source nodes such as gene numbers (node sizes) and *p* values (node colors) [49]. In “regulation processes” cluster of all three networks, genes involved in “regulation of transcription, DNA-dependent” accounted for the enrichments of all other nodes in this network branch since the end node was almost the same size and color as its source nodes, suggesting that transcriptional regulations might have common contributions in plants responding to chilling stress. In the cluster of “stimulus responses”, the network patterns showed that cellular responses to a wide range of stresses were aroused by chilling stress in both *C. bungeana* and Arabidopsis, which were probably due to the cross-tolerance mechanisms of plants. The cluster of “metabolism processes” comprised much more over-representative terms in the network of *C. bungeana* than that of Arabidopsis. “Flavonoid biosynthetic process” was the only over-representative term of this cluster presented in both *C. bungeana* and Arabidopsis (ATH-SR).

Twelve biological processes (end nodes in the networks) were found to be common in both *C. bungeana* and Arabidopsis (ATH-SR or ATH-MA), and ten of them were related to stimulus responses (Table 6). Genes “response to cold” were over-representative in all three networks, suggesting that our chilling stress treatments were efficient. However, the genes involved in “cold acclimation” did not over-represent in *C. bungeana* as did in Arabidopsis (Figure 3), indicating that cold acclimation mechanisms were not activated by chilling in *C. bungeana*. The results imply that *C. bungeana* may not have a cold acclimated mechanism or may have cold acclimated mechanisms different from that of Arabidopsis. For plants from temperate regions, cold acclimation is critical for them to tolerate freezing temperatures [8]. However, since cold acclimation requires a relatively long period of time to get freezing tolerance, such mechanisms may not be suitable for plants like *C. bungeana* in harsh environments. More rapid and efficient mechanisms are needed for such plants.

**Table 6:** Over-representative GO terms* in chilling-treated*C. bungeana*and Arabidopsis

**GO ID**	**GO functional description**	**Corrected*p*-value**
***Biological process:***		
80167	response to karrikin	1.59E-24
9611	response to wounding	9.61E-23
10200	response to chitin	1.61E-19
6355	regulation of transcription, DNA-dependent	3.72E-16
9414	response to water deprivation	1.89E-09
9737	response to abscisic acid stimulus	2.36E-08
9409	response to cold	1.10E-07
50832	defense response to fungus	1.20E-07
6979	response to oxidative stress	8.15E-06
9651	response to salt stress	2.92E-05
10224	response to UV-B	4.68E-04
9813	flavonoid biosynthetic process	7.10E-03
***Molecular function:***		
3700	sequence-specific DNA binding transcription factor activity	1.11E-23

* Only GO terms of the end nodes in the network were presented.

Besides abscisic acid [50] and chitin responses [51], which were known to be involved in cold tolerance of plants, the biological process “response to karrikin” was found to be a common response to chilling stress in both *C. bungeana* and Arabidopsis. To our knowledge, no previous study reported the involvement of karrikins in cold tolerance of plants. Karrikins are a new group of plant growth regulators discovered in smoke that can stimulate seed germination [52]. The biological and molecular functions of karrikins are largely unknown at present. Our results suggested that karrikins might play important roles in chilling tolerance of *C. bungeana* and Arabidopsis.

Nineteen biological processes were over-represented in chilling-treated *C. bungeana* but not in Arabidopsis. Nonetheless, it did not mean that such processes were specific to chilling responses of *C. bungeana* since most of them, such as salicylic acid [53,54], jasmonic acid [54], and immune response [55], were reported to be involved in chilling response of Arabidopsis or other plants. However, two processes, “protein phosphorylation” and “protein autoubiquitination”, should be emphasized. Post-translational modifications of pre-existing proteins are believed to be a rapid pathway to get tolerance in plant responses to chilling stress and have important roles in plant cold acclimation [8]. In alfafa, low temperature lead to rapid inhibition of PP2A activity, and in turn lead to phosphorylation of proteins involved in cold tolerance acquisitions [56,57]. Transcriptional activation of genes of several kinase families were also found under low temperature stress, such as MAP kinase family genes *MKK2*[58], *OsMEK1* and *OsMAP1*[59], CDPK family genes *OsCDPK7*[60,61], *OsCDPK13*[62] and *PaCDPK1*[63], and CIPK family genes *CIPK3*[64] and *CIPK7*[65]. Although many studies reported that certain protein kinases were activated and their transcriptional expression increased in response to cold stress, few studies reported that the expressions of protein kinases as a whole increased at transcriptome level. In our study, a large number of genes whose products were involved in protein phosphorylation were over-represented in chilling up-regulated DEGs in *C. bungeana*. Given the habitats of *C. bungeana*, in which the daytime temperatures fluctuate frequently and during almost the whole plant growing seasons, our results suggest that protein phosphorylation may be an important mechanism for rapid and flexible regulation of cold tolerance of *C. bungeana*.

Protein autoubiquitination may play similar roles as protein phosphorylation. In Arabidopsis, ubiquitination of ICE1 by HOS1 which leads to ICE1 degradation is vital for the activation of CBF pathways [66]. In this study, eight chilling up-regulated unigenes of *C. bungeana* were associated with protein ubiquitination, six of which might be involved directly in protein ubiquitination (Table 7). However, POGs of *HOS1* was not on the list. Therefore, the roles of protein ubiquitination in chilling responses of *C. bungeana* need further investigations.

**Table 7:** Chilling up-regulated unigenes annotated with ubiquitination function

**Unigene**	**AGI model**	**Functional description**
CBT4839	AT5G57740.1	ubiquitin ligase, XB3 ortholog 2 in Arabidopsis thaliana (XBAT32)
CBT21694	AT5G57740.1	ubiquitin ligase, XB3 ortholog 2 in Arabidopsis thaliana (XBAT32)
CBT25162	AT3G52450.1	U-box domain E3 ubiquitin ligase protein, plant U-box 22 (PUB22)
CBT24438	AT2G35930.1	U-box domain E3 ubiquitin ligase protein, plant U-box 23 (PUB23)
CBT12523	AT3G11840.1	U-box domain E3 ubiquitin ligase protein, plant U-box 24 (PUB24)
CBT9995	AT3G12630.1	A20/AN1-like zinc finger family protein
CBT15631	AT3G46620.1	zinc finger (C3HC4-type RING finger) family protein

**Figure 4:**
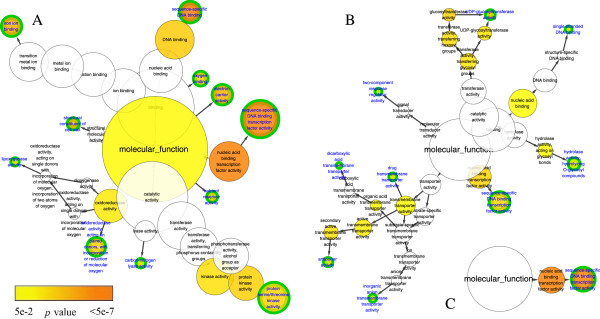
**Molecular function network of over-representative GO terms of chilling up-regulated DEGs.A**, *C. bungeana*; **B**, ATH-SR; **C**, ATH-MA. Node size represented gene number in node and node filled color represented *p* value. White nodes were not significant over-representative terms. End nodes were indicated by green border and blue label.

**Figure 5:**
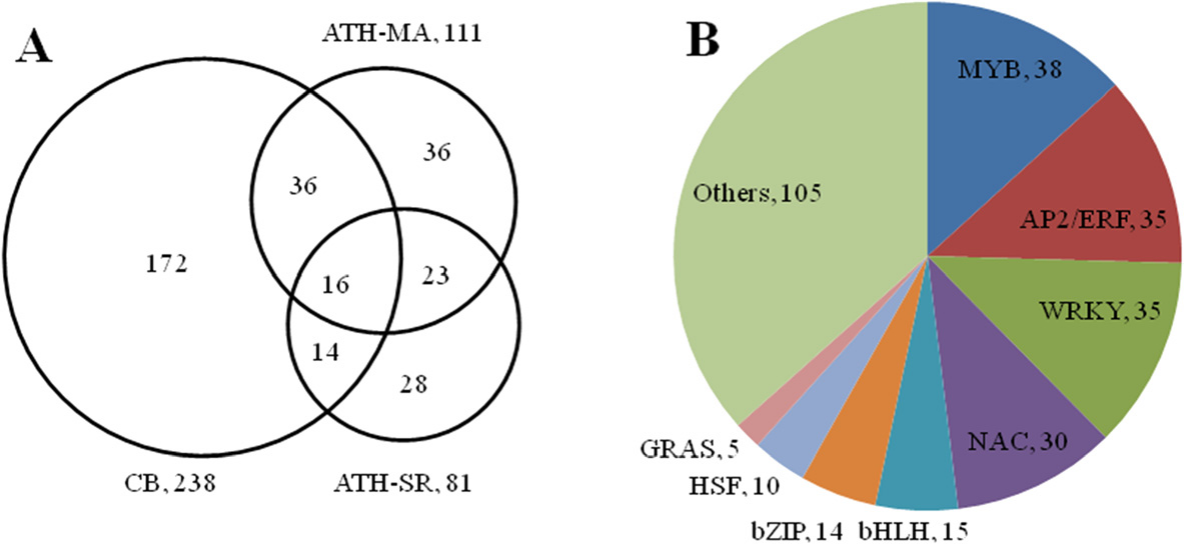
**Analysis of chilling up-regulated TFs. A**. Venn diagram of chilling up-regulated TFs in *C. bungeana* and Arabidopsis. **B**. Classification of chilling up-regulated transcription factors of *C. bungeana* by family.

Comparison of the molecular function networks of chilling up-regulated DEGs showed that only one term/node, “sequence-specific DNA binding transcription factor activity”, was in common in both *C. bungeana* and Arabidopsis (Figure 4, Table 6). It was consistent with the over-representative term of “regulation of transcription, DNA-dependent” in network of biological process. However, only a small amount of TF POGs of the three experiments were overlapped (Figure 5A), including *ZAT12/RHL41*, *COL1*, *TOC1* and *RAP2.7* orthologs (Table 8) which were reported to be involved in plant cold responses [33,34,67,68]. Surprisingly, none of the *CBFs* (*CBF1/DREB1b*, *CBF2/DREB1c* and *CBF3/DREF1a*) was on the list of overlapped TF genes though *CBF2* and *CBF3* were chilling up-regulated in Arabidopsis as was shown by both ATH-SR and ATH-AR data (Additional file 4). In fact, no ortholog of Arabidopsis *CBF1* or *CBF2* was found in the transcriptome of *C. bungeana*, while there were orthologs of *CBF3* and *CBF4* (data not shown). The results suggest that the transcriptional activation mechanism of *C. bungeana* differs greatly from that of Arabidopsis in chilling responses although they share some common mechanisms. Given the important roles of *CBFs* in plant cold acclimation, lack of *CBF* orthologs suggests that cold acclimation mechanisms may be weak in or absent from *C. bungeana*, consisting with the finding that genes involved in cold acclimation was not enriched in chilling up-regulated DEGs of *C. bungeana*. Classification results showed that MYB, AP2/ERF, WRKY and NAC family members represent the most abundant TFs in chilling up-regulated DEGs of *C. bungeana* (Figure 5B). The data are insightful for further investigation of specific tolerance mechanisms of *C. bungeana*.

**Table 8:** **Chilling up-regulated TFs overlapped in*C. bungeana*and Arabidopsis**

**Locus Id**	**All gene symbols**	**Description**
AT5G15850	COL1	Homologous to the flowering-time gene CONSTANS.
AT2G40140	CZF1; SZF2; ZFAR1	CZF1
AT5G05410	DREB2A	Encodes a transcription factor that specifically binds to DRE/CRT cis elements (responsive to drought and low-temperature stress)
AT3G02990	HSFA1E	Member of Heat Stress Transcription Factor (Hsf) family
AT2G28550	RAP2.7	Related to AP2.7 (RAP2.7)
AT5G59820	RHL41; ZAT12	Encodes a zinc finger protein involved in high light and cold acclimation
AT5G17300	RVE1	Myb-like transcription factor that regulates hypocotyl growth by regulating free auxin levels in a time-of-day specific manner.
AT4G18390	TEOSINTE BRANCHED 1; TCP2	TEOSINTE BRANCHED 1, cycloidea and PCF transcription factor 2 (TCP2)
AT5G61380	TOC1;PRR1	Pseudo response regulator involved in the generation of circadian rhythms.
AT2G47260	WRKY23	Encodes a member of WRKY Transcription Factor
AT2G38470	WRKY33	Member of the plant WRKY transcription factor family
AT1G80840	WRKY40	Pathogen-induced transcription factor
AT5G54470		B-box type zinc finger family protein
AT5G58620		Zinc finger (CCCH-type) family protein
AT1G43860		Sequence-specific DNA binding transcription factors
AT2G47890		B-box type zinc finger protein with CCT domain

**Figure 6:**
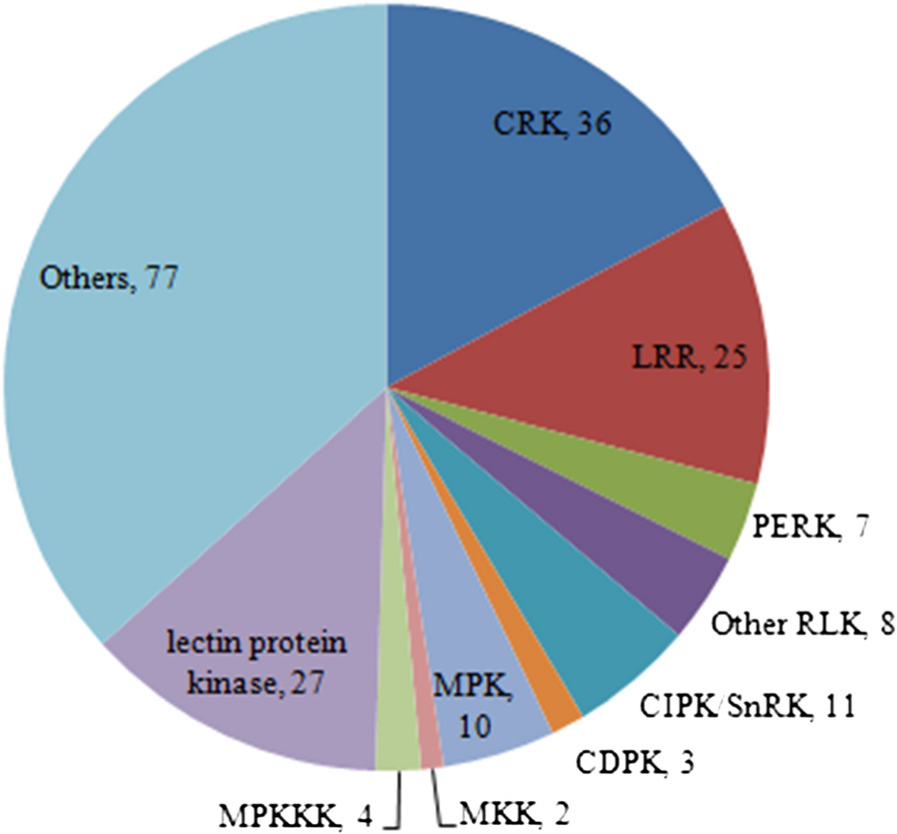
**Classification of chilling up-regulated protein kinases of *C. bungeana *by family.**

Ten terms/nodes in the network of *C. bungeana* were not in the networks of Arabidopsis (Figure 4, Table 9). Again, the over-representation of “protein serine/threonine kinase activity” was overlapped with “protein phosphorylation” in the network of biological process. The most abundant protein kinases in chilling up-regulated DEGs encoded cysteine-rich receptor-like protein kinases (CRK), whose roles in plant cold responses were largely unknown (Figure 6, Additional file 5). Genes for leucine-rich receptor-like protein kinases (LRR RLK) ranked the second. A small number of POGs of *CDPKs*, *CIPKs*, *MPKs*, *MKKs* and *MKKKs*, some of which have been reported to be involved in plant cold responses [58-65], were found in chilling up-regulated DEGs of *C. bungeana*.

**Table 9:** Over-representative GO terms* in chilling stressed*C. bungeana*but not in Arabidopsis

**GO ID**	**Description**	**Corrected*p*-value**
***Biological process:***		
*Stimulus responses related:*		
9751	response to salicylic acid stimulus	7.70E-10
9753	response to jasmonic acid stimulus	6.36E-09
52544	defense response by callose deposition in cell wall	3.95E-06
42742	defense response to bacterium	1.09E-05
45087	innate immune response	5.27E-03
71214	cellular response to abiotic stimulus	5.41E-03
42594	response to starvation	2.31E-02
*Metabolism processes:*		
9695	jasmonic acid biosynthetic process	3.95E-06
42343	indole glucosinolate metabolic process	6.93E-04
9074	aromatic amino acid family catabolic process	1.51E-03
9065	glutamine family amino acid catabolic process	1.43E-02
10120	camalexin biosynthetic process	2.15E-02
6558	L-phenylalanine metabolic process	1.97E-02
*Developmental processes:*		
9901	anther dehiscence	1.00E-02
48544	recognition of pollen	1.40E-02
*Others:*		
6468	protein phosphorylation	9.85E-04
71702	organic substance transport	1.28E-02
7165	signal transduction	1.57E-02
51865	protein autoubiquitination	2.15E-02
***Molecular function:***		
43565	sequence-specific DNA binding	5.53E-09
5506	iron ion binding	2.42E-05
9055	electron carrier activity	4.39E-05
5199	structural constituent of cell wall	9.06E-05
16705	oxidoreductase activity, acting on paired donors, with incorporation or reduction of molecular oxygen	5.20E-04
19825	oxygen binding	6.82E-04
45735	nutrient reservoir activity	9.46E-04
4674	protein serine/threonine kinase activity	9.60E-04
16165	lipoxygenase activity	9.74E-03
16840	carbon-nitrogen lyase activity	1.12E-02

* Only GO terms of the end nodes in the network were presented.

### KEGG pathway analysis of up-regulated DEGs of C. bungeana in response to chilling stress and comparison with Arabidopsis

**Figure 7:**
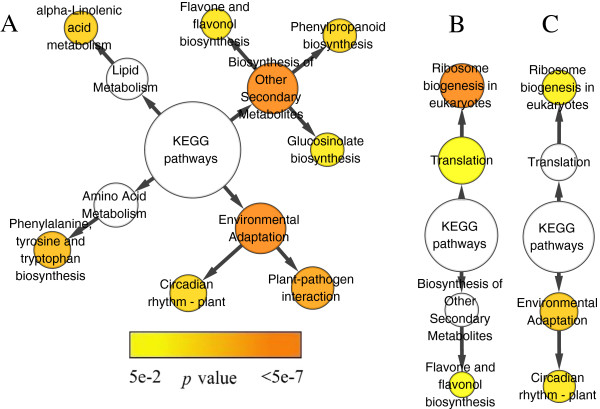
**KEGG pathway network of chilling up-regulated DEGs. A**, *C. bungeana*; **B**, ATH-SR; **C**, ATH-MA. Node size represented gene number in node and node filled color represented *p* value.

KEGG pathway network analysis showed that “Biosynthesis of Other Secondary Metabolites” and “Environmental Adaptation” were enriched in chilling up-regulated DEGs of *C. bungeana* (Figure 7). The over-representation of “Biosynthesis of Other Secondary Metabolites” was due to biosynthesis of three kinds of secondary metabolites: flavonoids, glucosinolates and phenylpropanoids; and the over-presentation of “Environmental Adaptation” was due to enrichment of genes involved in “plant-pathogen interaction” and “circadian rhythm” regulation. Besides, genes involved in alpha linolenic acid metabolism were also enriched. The phenylalanine/tyrosine/tryptophan biosynthesis pathway was overlapped with phenylpropanoid biosynthesis. In Arabidopsis, genes involved in flavonoids biosynthesis and circadian rhythm pathways were also enriched in chilling up-regulated DEGs.

All over-represented pathways in *C. bungeana*, regardless of whether they were enriched in Arabidopsis, had proved to be important in plant cold tolerance. For instance, circadian rhythm regulates the expression of CBFs [28,69], the core identified TFs that involved in plant cold tolerance. As another example, under chilling stress, plants preferentially accumulate polyunsaturated fatty acids such as linoleic and linolenic fatty acids [70-72], and genetically increasing of unsaturated fatty acids or lipids could enhance cold tolerance of transgenic plants, probably by maintaining membrane fluidity under cold stress [73,74]. Our previous findings indicated that cold tolerance of *C. bungeana* was correlated with changes in membrane lipids and membrane-associated enzymes [3]. Under chilling treatment, the proportion of unsaturated fatty acid in the plasma membrane increased significantly in callus of *C. bungeana*[75]. Paralleling to these results, KEGG analysis in this study showed that unigenes involved in "alpha-Linolenic acid metabolism" were enriched significantly in chilling up-regulated DEGs, suggesting that lipid metabolism, especially linolenic acid metabolism, might play a role in chilling tolerance of *C. bungeana*.

### GO network analysis of down-regulated DEGs of C. bungeana in response to chilling stress and comparison with Arabidopsis

**Figure 8:**
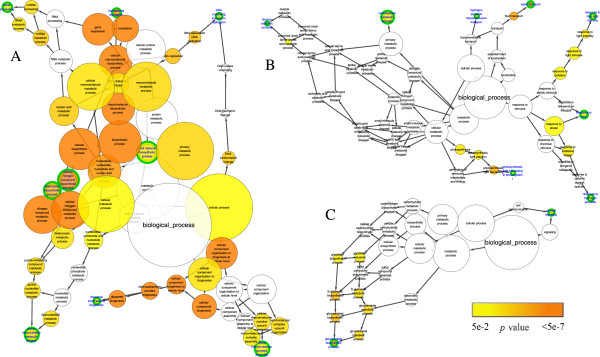
**Biological process network of over-representative GO terms of chilling stress down-regulated DEGs. A**, *C. bungeana*; **B**, ATH-SR; **C**, ATH-MA. Node size represented gene number in node and node filled color represented *p* value. White nodes were not significant over-representative terms. End nodes were indicated by green border and blue label.

**Figure 9:**
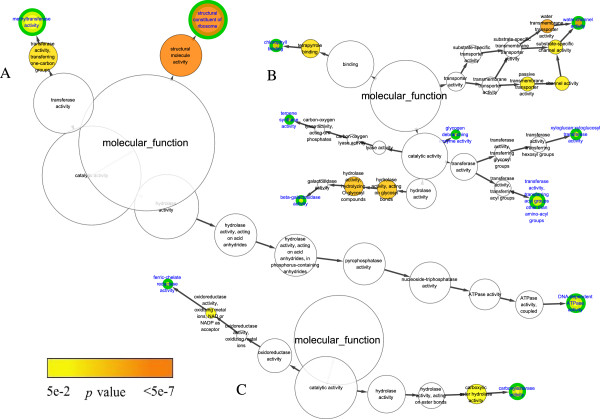
**Molecular function network of over-representative GO terms of chilling stress down-regulated DEGs.A**, *C. bungeana*; **B**, ATH-SR; **C**, ATH-MA. Node size represented gene number in node and node filled color represented *p* value. White nodes were not significant over-representative terms. End nodes were indicated by green border and blue label.

In chilling stress down-regulated DEGs of both *C. bungeana* and Arabidopsis, there were several over-represented terms in every biological process networks (Figure 8). However, no over-represented term was in common in *C. bungeana* and Arabidopsis. Furthermore, none of the over-represented term was the same between two networks of Arabidopsis, although both of them were related to chilling stressed down-regulated DEGs. Similar results were also found in the networks of molecular function (Figure 9). The huge discrepancy among the networks implied that the gene members of chilling stress down-regulated DEGs were highly variable, which might be affected by some subtle experimental details other than chilling temperatures only. It was hard to deduce an unbiased mechanism from their networks analysis. Therefore, no further analysis was performed for the down-regulated DEGs. 

## Conclusions

*C. bungeana* is a perennial subnival alpine plant with high capacity of chilling and freezing resistance. In recent years, much effort has been taken in our research group to reveal the cold tolerance mechanisms of this plant at physiological and molecular levels. In this paper, we provide the first study on the transcriptome of chilling stressed seedlings of *C. bungeana*. We got 54,870 assembled unigenes using the Trinity de novo assembly method, and a number of chilling regulated genes were identified, providing useful resources for gene mining to improve cold tolerance of plants. Furthermore, the comparison of the functional networks of chilling regulated genes in *C. bungeana* and Arabidopsis provided informative results, which could help us tell the differences in cold tolerance mechanisms between *C. bungeana* and Arabidopsis. We found that karrikins might be new plant growth regulators involved in chilling tolerance of plants. Although gene expressions at the transcriptional level were stimulated by chilling in both *C. bungeana* and Arabidopsis, their activation networks were different as suggested by TFs analysis. Cold acclimation mechanism may be weak in or absent from *C. bungeana* because of lack of some CBFs orthologs. Alternatively, protein phosphorylation and ubiquitination may serve as more rapid and flexible cold tolerance mechanisms for *C. bungeana* to adapt to the harsh cold environments.

## Methods

### Plant material, growth conditions and treatments

Plant regeneration of *C. bungeana* via somatic embryogenesis was performed as described by Wang *et al*. [76]. Callus was induced from matured seeds of *C. bungeana* on MS medium containing 4.0 mg l^-1^ GA3, 2.0 mg l^-1^ NAA, and 2.0 mg l^-1^ 2,4-D. Seedlings were regenerated from callus on MS medium containing 3% sucrose in about 3 weeks. Regenerated plants were transferred to new MS medium containing 3% sucrose and grown at 22°C with a 14 h photoperiod under 80 μmol m^-2^ s^-1^ fluorescent light for further 7 days before treatments. For each treatment, ten plants (roots, shoots and leaves) were randomly pooled and treated in MS liquid medium containing 3% sucrose at 22°C or 2°C. Chilling stress was initiated 4 hours after dawn (zeitgeber time 4; ZT4). Upon the treatment time reaching 24 hours, both control and chilling stressed samples were collected at the same time point and frozen immediately with liquid nitrogen.

### RNA extraction, cDNA library construction and RNA sequencing

For RNA sequencing, total RNA was extracted using TRIzol reagent (Invitrogen, Carlsbad, CA, USA). The quality of total RNA was checked using the NanoDrop Spectrometer (ND-1000 Spectrophotometer, Peqlab) and the Agilent 2100 Bioanalyzer (RNA Nano Chip, Agilent). High quality RNA samples (20 μg each) were sent to Beijing Genomics Institute (BGI, Shenzhen) for cDNA libraries construction and sequencing using Illumina HiSeq™ 2000. The cDNA library construction method and Illumina deep-sequencing processes were the same as described by Xu et al. [45].

### De novo assembly and sequences clustering

The Trinity method [42] was used for *de novo* assembly of the clean reads to generate Trinity unigenes, with optimized k-mer length of 25. Then, the Trinity unigenes of both libraries were clustered with TGICL software [43] to get sequences (final unigenes) that cannot be extended on either end. *De novo* assembly was also conducted with SOAPdenovo software [44] with optimized k-mer length of 41.

Files containing the raw read sequences and their quality scores are available from the National Center for Biotechnology Information (NCBI) Short Read Archive with the accession number: SRA054354. The Trinity unigenes have been deposited in the Transcriptome Shotgun Assembly Sequence Database (TSA) at NCBI [GenBank: JW988067-JW999999, KA000001-KA089547].

### Expression analysis and identification of differentially expressed genes (DEGs)

Clean reads were mapped back to assembled unigenes with SOAPaligner (version 2.21) [44] allowing maximum 2 mismatches. The reads with unique best hits were counted for each unigene. The expression level of *C. bungeana* unigene was normalized by the number of RPKM (reads per kilobase exon region per million mapped reads) [77]. Since Illumina sequencing method is highly sensitive, we only used a subset of unigenes which presented in both sequencing libraries with a minimal RPKM of 1 for DEGs analysis. Unigene expressions were analyzed using DEGseq R package [78] with MARS method. Chilling-regulated DEGs were identified with Benjamini *q* < 0.001 [79] and normalized fold change > =2.

For comparisons, two public available data sets of Arabidopsis were used in our study. One data set (referred to ATH-SR, means Arabidopsis short reads) was RNA sequencing data downloaded from NCBI Sequence Read Archive (SRA) database (http://www.ncbi.nlm.nih.gov), including a chilling-treated sample (4°C; SRA accession: SRX006193) and a control (21°C; SRA accession: SRX006704) sample [80]. After removing low quality reads (polyA/T/G/C sequences) and trimming off four NTs of both ends, all clean reads (28 NTs long) were mapped to Arabidopsis cDNAs (TAIR10) with SOAPaligner. DEGs identification was the same as described above. The DEGs and indentified gene with RPKM > =1 were listed in Additional file 6.

The other data set (referred to ATH-AR, means Arabidopsis array) was Affimetrix microarray data set (Expression Set: ME00325) [81] downloaded from TAIR (http://www.arabidopsis.org). Only cel files for 4 chilling-treated samples (2 for roots and 2 for shoots, 24-hour chilling-treated) and 4 control samples were used here. The cel files were imported into R and analyzed with Affy package [82]. Root and shoot arrays were analyzed separately. Probes expressed in all root or shoot arrays were considered to be presented probes (by mas5 present calls). Differential expressed probes were identified using mas5 method of with FDR corrected *p* < 0.05 and fold change > =2 and mapped to Arabidopsis transcripts. The gene lists of roots and shoots were combined together to get chilling regulated DEGs and all expressed genes for further analysis (Additional file 7).

### Functional categorization

We used two methods for functional categorization of unigenes.

To get general gene ontology (GO) annotations for all unigenes, sequences longer than 200 bp were aligned to three public databases (NR, Swiss-Prot and KEGG) by BLASTX with E-value < =1e-5. The GO annotations for the top blast hits were retrieved with Blast2GO program [83] and used to annotate the *C. bungeana* transcripts. GO functional classification was performed by WEGO website tool [84].

For GO terms and KEGG pathways enrichment analysis, we used the Arabidopsis annotation systems. Briefly, the sequences of all unigenes were aligned against Arabidopsis peptide database (TAIR10) using BLASTX program with E-value < =1e-5. The top blast hits were considered to be putative orthologous genes (POGs). Then the *C. bungeana* unigenes were annotated with GO (downloaded from TAIR) and KEGG annotations (ath00001.keg, from http://www.kegg.jp/) for Arabidopsis POGs, respectively. The ontology (GO and KEGG) enrichment was analyzed with BiNGO plugins [49] for Cytoscape [85], using hypergeometric test for statistical analysis. For *p* value correction, we used rigorous Bonferroni correction method. The cutoff *p* value after correction was 0.05. For ATH-SR dataset, since the stressed sample was pooled from seedlings subjected to various periods of chilling-treated (1, 2, 5, 10, 24 hours of stressed) [80], the expressions of DEGs specific to a certain stage might have been “normalized”. Therefore, to get more information, we used FDR method instead of Bonferronic method for *p* value correction to find over-representative terms with BiNGO.

### Quantitative real-time PCR (qPCR)

The gene-specific primers for real-time PCR analysis were designed using Primer Premier (version 5.0) software (PREMIER Biosoft). The specifities of primer pairs were confirmed by BLASTN with non-redundant unigene set of *C. bungeana* transcripts and the PCR products were checked by agrose electrophoresis to ensure single band amplifications. The primer sequences for all unigenes used in this study were listed in Additional file 8.

For qPCR analysis, total RNAs were extracted from control or chilling stressed *C. bungeana* seedlings (two biological repeats) with TRIZOL reagent and treated (20 μg RNA) with 1U DNase (TAKARA, Japan). cDNA was transcribed reversely from 1 μg of DNase-treated RNA with 200U M-MLV reverse transcriptase (Promega, USA) and analyzed with Platinum SYBR green qPCR supermix-UDG reagents (Invitrogen).

Before quantification of unigenes, the geNorm method was applied to select stable expressed unigenes in the four samples as reference genes [86]. A total of 8 candidate reference unigenes were selected for reference genes screening, including an *ACTIN2* ortholog, 3 unigenes showed stable expression levels in RNA-seq analysis and the other 4 unigenes were orthologs of recommended Arabidopsis reference genes [87]. The information of reference gene candidates and the geNorm analysis results were shown Additional file 8. Three unigenes (CBT10872/AT3G60800, CBT28565/AT5G27630 and CBT12464/AT2G28390) expressed most stably in control and chilling-treated samples were selected and used for all qPCR analysis.

qPCR analysis was performed with three technical repeats for each sample. The relative expression levels of unigenes were normalized with the three selected reference genes with Pfaffl method [86,88].

#### Availability of supporting data

The data sets supporting the results of this article are available in the NCBI GenBank repository, [http://www.ncbi.nlm.nih.gov/sites/nuccore?term=104929[BioProject]], and in the NCBI SRA repository, [http://www.ncbi.nlm.nih.gov/sra?term=SRA054354].

## Abbreviations

COR: Cold-responsive; CBF: CRT binding transcription factor; RNA-seq: RNA sequencing; DEG: Differentially expressed gene; GO: Gene ontology; KEGG: Kyoto Encyclopedia of Genes and Genomes; POG: Putative orthologous gene; qPCR: Quantitative real-time PCR; TF: Transcription factor.

## Competing interests

The authors declare that they have no competing interests.

## Authors’ contributions

ZZ designed the experiments and drafted the manuscript (zgzhao@lzu.edu.cn). LT contributed to data analysis and interpretation (tanll@lzu.edu.cn). CD prepared plant materials and carried out qPCR analysis (tsdcy2006@126.com). HZ participated in plant preparations (zhanghua@lzu.edu.cn). QW provided part of the financial support (qbwu@lzb.ac.cn). LA conceived of the study and provided financial support for the project (lizhean@lzu.edu.cn). All authors read and approved the final manuscript.

## Supplementary Material

Additional file 1 Complete list of unigenes with BLASTX hits. Click here for file

Additional file 2 Complete list of chilling regulated DEGs identified with fold change > =2 and q < 0.001. Click here for file

Additional file 3 Complete list of chilling regulated DEGs identified with fold change > =2, q < 0.001 and RPKM > =1. Click here for file

Additional file 4 **Chilling up-regulated TFs.** 1. List of chilling up-regulated TFs in both ATH-SR and ATH-MA. 2. List all chilling up-regulated TFs in Arabidopsis (ATH-SR or ATH-MA). 3. All chilling up-regulated TFs (orthologs) in *C. bungeana*. Click here for file

Additional file 5 List of chilling up-regulated protein serine/threonine kinase in C. bungeana. Click here for file

Additional file 6 **List of chilling regulated DEGs and all expressed genes of ATH-SR.** 1. List of chilling up-regulated DEGs (SR). 2. List of chilling down-regulated DEGs (SR). 3. List all genes RPKM > =1 (SR). Click here for file

Additional file 7 **List of chilling regulated DEGs and all expressed genes of ATH-MA.** 1. List of chilling up-regulated DEGs (MA). 2. List of chilling down-regulated DEGs (MA). 3. List all expressed genes (MA). Click here for file

Additional file 8 **Primers and reference gene selections.** 1. Primers for reference gene selection. 2. Primers for qPCR verification. 3. Unigenes for qPCR reference gene selection. 4. geNorm results of reference gene selection. Click here for file
