# Supplementary material for: Deep-sequencing transcriptome analysis of chilling tolerance mechanisms of a subnival alpine plant, Chorispora bungeana
Source: BMC Plant Biol. 2012 Nov 21;12:222. doi: 10.1186/1471-2229-12-222 (PMC3571968; doi:10.1186/1471-2229-12-222)
Supplement: Additional file 4 — Chilling up-regulated TFs. 1. List of chilling up-regulated TFs in both ATH-SR and ATH-MA. 2. List all chilling up-regulated TFs in Arabidopsis (ATH-SR or ATH-MA). 3. All chilling up-regulated TFs (orthologs) in C. bungeana. [file 1471-2229-12-222-S4.docx]

**1. Chilling up-regulated TFs in Arabidopsis (ATH-SR or ATH-MA)**

| AT5G48250 | B-box type zinc finger protein with CCT domain |
| --- | --- |
| AT5G54470 | B-box type zinc finger family protein |
| AT4G29190 | Zinc finger C-x8-C-x5-C-x3-H type family protein |
| AT2G43500 | Plant regulator RWP-RK family protein |
| AT3G45260 | C2H2-like zinc finger protein |
| AT1G76580 | Squamosa promoter-binding protein-like (SBP domain) transcription factor family protein |
| AT4G12750 | Homeodomain-like transcriptional regulator |
| AT5G58620 | zinc finger (CCCH-type) family protein |
| AT1G43860 | sequence-specific DNA binding transcription factors |
| AT2G47890 | B-box type zinc finger protein with CCT domain |
| AT2G40140 | CZF1 |
| AT2G24500 | FZF |
| AT1G49720 | abscisic acid responsive element-binding factor 1 (ABF1) |
| AT2G45660 | AGAMOUS-like 20 (AGL20) |
| AT1G77850 | auxin response factor 17 (ARF17) |
| AT1G18400 | BR enhanced expression 1 (BEE1) |
| AT4G25470 | C-repeat/DRE binding factor 2 (CBF2) |
| AT5G15850 | CONSTANS-like 1 (COL1) |
| AT3G50260 | cooperatively regulated by ethylene and jasmonate 1 (CEJ1) |
| AT4G25480 | dehydration response element B1A (DREB1A) |
| AT5G05410 | DRE-binding protein 2A (DREB2A) |
| AT2G23340 | DREB and EAR motif protein 3 (DEAR3) |
| AT3G15210 | ethylene responsive element binding factor 4 (ERF4) |
| AT3G02990 | heat shock transcription factor A1E (HSFA1E) |
| AT5G39760 | homeobox protein 23 (HB23) |
| AT1G46768 | related to AP2 1 (RAP2.1) |
| AT2G28550 | related to AP2.7 (RAP2.7) |
| AT5G59820 | RESPONSIVE TO HIGH LIGHT 41 (RHL41) |
| AT5G17300 | REVEILLE 1 (RVE1) |
| AT5G37260 | REVEILLE 2 (RVE2) |
| AT2G31380 | salt tolerance homologue (STH) |
| AT1G27730 | salt tolerance zinc finger (STZ) |
| AT4G18390 | TEOSINTE BRANCHED 1, cycloidea and PCF transcription factor 2 (TCP2) |
| AT5G61380 | TIMING OF CAB EXPRESSION 1 (TOC1) |
| AT4G31800 | WRKY DNA-binding protein 18 (WRKY18) |
| AT2G47260 | WRKY DNA-binding protein 23 (WRKY23) |
| AT2G38470 | WRKY DNA-binding protein 33 (WRKY33) |
| AT1G80840 | WRKY DNA-binding protein 40 (WRKY40) |
| AT5G04340 | zinc finger of Arabidopsis thaliana 6 (ZAT6) |

**2. List all chilling up-regulated TFs in Arabidopsis (ATH-SR or ATH-MA)**

| AT1G69570 | Dof-type zinc finger DNA-binding family protein |
| --- | --- |
| AT2G33710 | Integrase-type DNA-binding superfamily protein |
| AT5G48250 | B-box type zinc finger protein with CCT domain |
| AT1G26790 | Dof-type zinc finger DNA-binding family protein |
| AT5G54470 | B-box type zinc finger family protein |
| AT3G45260 | C2H2-like zinc finger protein |
| AT5G52020 | Integrase-type DNA-binding superfamily protein |
| AT1G76580 | Squamosa promoter-binding protein-like (SBP domain) transcription factor family protein |
| AT3G19360 | Zinc finger (CCCH-type) family protein |
| AT5G58620 | zinc finger (CCCH-type) family protein |
| AT1G43860 | sequence-specific DNA binding transcription factors |
| AT1G14580 | C2H2-like zinc finger protein |
| AT5G01200 | Duplicated homeodomain-like superfamily protein |
| AT1G62310 | transcription factor jumonji (jmjC) domain-containing protein |
| AT5G52660 | Homeodomain-like superfamily protein |
| AT1G74840 | Homeodomain-like superfamily protein |
| AT5G58900 | Homeodomain-like transcriptional regulator |
| AT2G43140 | basic helix-loop-helix (bHLH) DNA-binding superfamily protein |
| AT2G44940 | Integrase-type DNA-binding superfamily protein |
| AT1G05805 | basic helix-loop-helix (bHLH) DNA-binding superfamily protein |
| AT2G21320 | B-box zinc finger family protein |
| AT2G43500 | Plant regulator RWP-RK family protein |
| AT4G29190 | Zinc finger C-x8-C-x5-C-x3-H type family protein |
| AT5G44260 | Zinc finger C-x8-C-x5-C-x3-H type family protein |
| AT2G41900 | CCCH-type zinc finger protein with ARM repeat domain |
| AT5G51190 | Integrase-type DNA-binding superfamily protein |
| AT5G04760 | Duplicated homeodomain-like superfamily protein |
| AT4G12750 | Homeodomain-like transcriptional regulator |
| AT2G47890 | B-box type zinc finger protein with CCT domain |
| AT1G01720 | ATAF1 |
| AT5G08130 | BIM1 |
| AT2G40140 | CZF1 |
| AT2G24500 | FZF |
| AT4G23810 | WRKY53 |
| AT1G08970 | nuclear factor Y, subunit C9 (NF-YC9) |
| AT1G49720 | abscisic acid responsive element-binding factor 1 (ABF1) |
| AT1G71692 | AGAMOUS-like 12 (AGL12) |
| AT2G45660 | AGAMOUS-like 20 (AGL20) |
| AT5G10510 | AINTEGUMENTA-like 6 (AIL6) |
| AT3G05800 | AtBS1(activation-tagged BRI1 suppressor 1)-interacting factor 1 (AIF1) |
| AT1G77850 | auxin response factor 17 (ARF17) |
| AT5G49450 | basic leucine-zipper 1 (bZIP1) |
| AT1G13600 | basic leucine-zipper 58 (bZIP58) |
| AT1G42990 | basic region/leucine zipper motif 60 (BZIP60) |
| AT1G75410 | BEL1-like homeodomain 3 (BLH3) |
| AT2G23760 | BEL1-like homeodomain 4 (BLH4) |
| AT1G18400 | BR enhanced expression 1 (BEE1) |
| AT4G25490 | C-repeat/DRE binding factor 1 (CBF1) |
| AT4G25470 | C-repeat/DRE binding factor 2 (CBF2) |
| AT2G46830 | circadian clock associated 1 (CCA1) |
| AT5G15850 | CONSTANS-like 1 (COL1) |
| AT3G02380 | CONSTANS-like 2 (COL2) |
| AT5G57660 | CONSTANS-like 5 (COL5) |
| AT3G07650 | CONSTANS-like 9 (COL9) |
| AT3G50260 | cooperatively regulated by ethylene and jasmonate 1 (CEJ1) |
| AT1G68550 | Integrase-type DNA-binding superfamily protein |
| AT4G23750 | cytokinin response factor 2 (CRF2) |
| AT5G53290 | cytokinin response factor 3 (CRF3) |
| AT4G27950 | cytokinin response factor 4 (CRF4) |
| AT4G25480 | dehydration response element B1A (DREB1A) |
| AT2G38340 | Integrase-type DNA-binding superfamily protein |
| AT1G51700 | DOF zinc finger protein 1 (DOF1) |
| AT5G05410 | DRE-binding protein 2A (DREB2A) |
| AT3G11020 | DRE/CRT-binding protein 2B (DREB2B) |
| AT2G23340 | DREB and EAR motif protein 3 (DEAR3) |
| AT2G36010 | E2F transcription factor 3 (E2F3) |
| AT1G28370 | ERF domain protein 11 (ERF11) |
| AT1G28360 | ERF domain protein 12 (ERF12) |
| AT5G44210 | erf domain protein 9 (ERF9) |
| AT3G25730 | ethylene response DNA binding factor 3 (EDF3) |
| AT5G47220 | ethylene responsive element binding factor 2 (ERF2) |
| AT3G15210 | ethylene responsive element binding factor 4 (ERF4) |
| AT5G47230 | ethylene responsive element binding factor 5 (ERF5) |
| AT1G73730 | ETHYLENE-INSENSITIVE3-like 3 (EIL3) |
| AT4G34590 | G-box binding factor 6 (GBF6) |
| AT3G22830 | heat shock transcription factor A6B (HSFA6B) |
| AT1G67970 | heat shock transcription factor A8 (HSFA8) |
| AT5G62020 | heat shock transcription factor B2A (HSFB2A) |
| AT3G24520 | heat shock transcription factor C1 (HSFC1) |
| AT3G02990 | heat shock transcription factor A1E (HSFA1E) |
| AT2G26150 | heat shock transcription factor A2 (HSFA2) |
| AT5G67060 | HECATE 1 (HEC1) |
| AT1G20693 | high mobility group B2 (HMGB2) |
| AT1G20696 | high mobility group B3 (HMGB3) |
| AT3G61890 | homeobox 12 (HB-12) |
| AT2G46680 | homeobox 7 (HB-7) |
| AT5G06710 | homeobox from Arabidopsis thaliana (HAT14) |
| AT5G39760 | homeobox protein 23 (HB23) |
| AT1G14687 | homeobox protein 32 (HB32) |
| AT1G70920 | homeobox-leucine zipper protein 18 (HB18) |
| AT3G17609 | HY5-homolog (HYH) |
| AT3G13810 | indeterminate(ID)-domain 11 (IDD11) |
| AT1G25250 | indeterminate(ID)-domain 16 (IDD16) |
| AT2G02070 | indeterminate(ID)-domain 5 (IDD5) |
| AT1G55110 | indeterminate(ID)-domain 7 (IDD7) |
| AT3G23030 | indole-3-acetic acid inducible 2 (IAA2) |
| AT5G25890 | indole-3-acetic acid inducible 28 (IAA28) |
| AT4G32280 | indole-3-acetic acid inducible 29 (IAA29) |
| AT3G20810 | 2-oxoglutarate (2OG) and Fe(II)-dependent oxygenase superfamily protein |
| AT1G78600 | light-regulated zinc finger protein 1 (LZF1) |
| AT5G49330 | myb domain protein 111 (MYB111) |
| AT1G48000 | myb domain protein 112 (MYB112) |
| AT4G34990 | myb domain protein 32 (MYB32) |
| AT1G66390 | myb domain protein 90 (MYB90) |
| AT5G62470 | myb domain protein 96 (MYB96) |
| AT5G67300 | myb domain protein r1 (MYBR1) |
| AT1G01010 | NAC domain containing protein 1 (NAC001) |
| AT5G63790 | NAC domain containing protein 102 (NAC102) |
| AT1G52890 | NAC domain containing protein 19 (NAC019) |
| AT1G64105 | NAC domain containing protein 27 (NAC027) |
| AT1G77450 | NAC domain containing protein 32 (NAC032) |
| AT3G04060 | NAC domain containing protein 46 (NAC046) |
| AT3G10500 | NAC domain containing protein 53 (NAC053) |
| AT3G49530 | NAC domain containing protein 62 (NAC062) |
| AT1G32870 | NAC domain protein 13 (NAC13) |
| AT5G20730 | NON-PHOTOTROPHIC HYPOCOTYL (NPH4) |
| AT3G50410 | OBF binding protein 1 (OBP1) |
| AT5G60850 | OBF binding protein 4 (OBP4) |
| AT2G19810 | CCCH-type zinc finger family protein |
| AT5G61270 | phytochrome-interacting factor7 (PIF7) |
| AT1G13260 | related to ABI3/VP1 1 (RAV1) |
| AT1G46768 | related to AP2 1 (RAP2.1) |
| AT1G53910 | related to AP2 12 (RAP2.12) |
| AT3G14230 | related to AP2 2 (RAP2.2) |
| AT1G43160 | related to AP2 6 (RAP2.6) |
| AT4G06746 | related to AP2 9 (RAP2.9) |
| AT2G28550 | related to AP2.7 (RAP2.7) |
| AT4G27410 | RESPONSIVE TO DESICCATION 26 (RD26) |
| AT5G59820 | RESPONSIVE TO HIGH LIGHT 41 (RHL41) |
| AT5G17300 | REVEILLE 1 (RVE1) |
| AT5G37260 | REVEILLE 2 (RVE2) |
| AT1G66470 | ROOT HAIR DEFECTIVE6 (RHD6) |
| AT2G31380 | salt tolerance homologue (STH) |
| AT1G27730 | salt tolerance zinc finger (STZ) |
| AT3G55980 | salt-inducible zinc finger 1 (SZF1) |
| AT3G54990 | SCHLAFMUTZE (SMZ) |
| AT4G18390 | TEOSINTE BRANCHED 1, cycloidea and PCF transcription factor 2 (TCP2) |
| AT1G22070 | TGA1A-related gene 3 (TGA3) |
| AT5G61380 | TIMING OF CAB EXPRESSION 1 (TOC1) |
| AT5G11590 | TINY2 (TINY2) |
| AT4G09820 | TRANSPARENT TESTA 8 (TT8) |
| AT4G13640 | unfertilized embryo sac 16 (UNE16) |
| AT4G31800 | WRKY DNA-binding protein 18 (WRKY18) |
| AT2G47260 | WRKY DNA-binding protein 23 (WRKY23) |
| AT2G30250 | WRKY DNA-binding protein 25 (WRKY25) |
| AT5G07100 | WRKY DNA-binding protein 26 (WRKY26) |
| AT4G18170 | WRKY DNA-binding protein 28 (WRKY28) |
| AT5G24110 | WRKY DNA-binding protein 30 (WRKY30) |
| AT2G38470 | WRKY DNA-binding protein 33 (WRKY33) |
| AT1G80840 | WRKY DNA-binding protein 40 (WRKY40) |
| AT2G46130 | WRKY DNA-binding protein 43 (WRKY43) |
| AT5G49520 | WRKY DNA-binding protein 48 (WRKY48) |
| AT5G04340 | zinc finger of Arabidopsis thaliana 6 (ZAT6) |

**3. All chilling up-regulated TFs (orthologs) in *C. bungeana*.**

| **Unigene** | **AGI** | **Functional description** | **Family** |
| --- | --- | --- | --- |
| CBT4667 | AT3G24650 | ABA INSENSITIVE 3 (ABI3) | - |
| CBT11636 | AT2G36080 | AP2/B3-like transcriptional factor family protein | - |
| CBT21800 | AT2G25900 | ATCTH | - |
| CBT5072 | AT4G35390 | AT-hook protein of GA feedback 1 (AGF1) | - |
| CBT27987 | AT5G62000 | auxin response factor 2 (ARF2) | - |
| CBT14676 | AT3G21150 | B-box 32 (BBX32) | - |
| CBT8494 | AT3G21150 | B-box 32 (BBX32) | - |
| CBT20394 | AT4G27310 | B-box type zinc finger family protein | - |
| CBT25625 | AT5G54470 | B-box type zinc finger family protein | - |
| CBT5533 | AT2G47890 | B-box type zinc finger protein with CCT domain | - |
| CBT6913 | AT4G39070 | B-box zinc finger family protein | - |
| CBT5294 | AT4G36870 | BEL1-like homeodomain 2 (BLH2) | - |
| CBT5731 | AT4G34610 | BEL1-like homeodomain 6 (BLH6) | - |
| CBT24863 | AT2G27990 | BEL1-like homeodomain 8 (BLH8) | - |
| CBT17035 | AT2G27990 | BEL1-like homeodomain 8 (BLH8) | - |
| CBT2263 | AT2G28710 | C2H2-type zinc finger family protein | - |
| CBT10720 | AT5G03510 | C2H2-type zinc finger family protein | - |
| CBT28837 | AT3G46070 | C2H2-type zinc finger family protein | - |
| CBT1864 | AT1G73805 | Calmodulin binding protein-like | - |
| CBT27140 | AT1G73805 | Calmodulin binding protein-like | - |
| CBT6647 | AT1G73805 | Calmodulin binding protein-like | - |
| CBT9652 | AT2G19810 | CCCH-type zinc finger family protein | - |
| CBT29247 | AT5G15850 | CONSTANS-like 1 (COL1) | - |
| CBT8154 | AT5G24930 | CONSTANS-like 4 (COL4) | - |
| CBT15621 | AT5G57660 | CONSTANS-like 5 (COL5) | - |
| CBT4630 | AT3G07650 | CONSTANS-like 9 (COL9) | - |
| CBT12573 | AT5G39660 | cycling DOF factor 2 (CDF2) | - |
| CBT23498 | AT2G40140 | CZF1 | - |
| CBT6062 | AT2G40140 | CZF1 | - |
| CBT2217 | AT1G29160 | Dof-type zinc finger DNA-binding family protein | - |
| CBT7897 | AT1G69570 | Dof-type zinc finger DNA-binding family protein | - |
| CBT26052 | AT1G69570 | Dof-type zinc finger DNA-binding family protein | - |
| CBT18452 | AT2G25930 | EARLY FLOWERING 3 (ELF3) | - |
| CBT41474 | AT2G25930 | EARLY FLOWERING 3 (ELF3) | - |
| CBT11632 | AT5G23150 | ENHANCER OF AG-4 2 (HUA2) | - |
| CBT45493 | AT1G73730 | ETHYLENE-INSENSITIVE3-like 3 (EIL3) | - |
| CBT38471 | AT1G73730 | ETHYLENE-INSENSITIVE3-like 3 (EIL3) | - |
| CBT17097 | AT1G76890 | GT2 | - |
| CBT52282 | AT1G76890 | GT2 | - |
| CBT24923 | AT2G45160 | HAIRY MERISTEM 1 (HAM1) | - |
| CBT25277 | AT2G45160 | HAIRY MERISTEM 1 (HAM1) | - |
| CBT26664 | AT2G45160 | HAIRY MERISTEM 1 (HAM1) | - |
| CBT25321 | AT3G60630 | HAIRY MERISTEM 2 (HAM2) | - |
| CBT18804 | AT3G60630 | HAIRY MERISTEM 2 (HAM2) | - |
| CBT7393 | AT5G62940 | HIGH CAMBIAL ACTIVITY2 (HCA2) | - |
| CBT47582 | AT1G20696 | high mobility group B3 (HMGB3) | - |
| CBT18953 | AT2G46680 | homeobox 7 (HB-7) | - |
| CBT9743 | AT4G40060 | homeobox protein 16 (HB16) | - |
| CBT49936 | AT4G36740 | homeobox protein 40 (HB40) | - |
| CBT5887 | AT2G22430 | homeobox protein 6 (HB6) | - |
| CBT3133 | AT2G33880 | homeobox-3 (HB-3) | - |
| CBT23175 | AT1G70920 | homeobox-leucine zipper protein 18 (HB18) | - |
| CBT53003 | AT3G60390 | homeobox-leucine zipper protein 3 (HAT3) | - |
| CBT42850 | AT3G60390 | homeobox-leucine zipper protein 3 (HAT3) | - |
| CBT2391 | AT2G44910 | homeobox-leucine zipper protein 4 (HB4) | - |
| CBT27882 | AT2G44910 | homeobox-leucine zipper protein 4 (HB4) | - |
| CBT872 | AT3G61150 | homeodomain GLABROUS 1 (HDG1) | - |
| CBT4995 | AT3G17609 | HY5-homolog (HYH) | - |
| CBT16877 | AT1G13300 | HYPERSENSITIVITY TO LOW PI-ELICITED PRIMARY ROOT SHORTENING 1 (HRS1) | - |
| CBT4789 | AT3G13810 | indeterminate(ID)-domain 11 (IDD11) | - |
| CBT5594 | AT3G13810 | indeterminate(ID)-domain 11 (IDD11) | - |
| CBT12814 | AT3G50700 | indeterminate(ID)-domain 2 (IDD2) | - |
| CBT15726 | AT3G50700 | indeterminate(ID)-domain 2 (IDD2) | - |
| CBT39314 | AT1G55110 | indeterminate(ID)-domain 7 (IDD7) | - |
| CBT23358 | AT1G51950 | indole-3-acetic acid inducible 18 (IAA18) | - |
| CBT5455 | AT3G23030 | indole-3-acetic acid inducible 2 (IAA2) | - |
| CBT52280 | AT5G11060 | KNOTTED1-like homeobox gene 4 (KNAT4) | - |
| CBT4280 | AT5G02840 | LHY/CCA1-like 1 (LCL1) | - |
| CBT49687 | AT4G00260 | maternal effect embryo arrest 45 (MEE45) | - |
| CBT10998 | AT2G22770 | NAI1 | - |
| CBT5955 | AT3G57670 | NO TRANSMITTING TRACT (NTT) | - |
| CBT18978 | AT3G14020 | nuclear factor Y, subunit A6 (NF-YA6) | - |
| CBT21306 | AT3G16500 | phytochrome-associated protein 1 (PAP1) | - |
| CBT16516 | AT1G08620 | PKDM7D | - |
| CBT25053 | AT3G02150 | plastid transcription factor 1 (PTF1) | - |
| CBT29973 | AT5G02030 | REPLUMLESS (RPL) | - |
| CBT17054 | AT5G59820 | RESPONSIVE TO HIGH LIGHT 41 (RHL41) | - |
| CBT17630 | AT1G06040 | SALT TOLERANCE (STO) | - |
| CBT6063 | AT3G55980 | salt-inducible zinc finger 1 (SZF1) | - |
| CBT15821 | AT1G43860 | sequence-specific DNA binding transcription factors | - |
| CBT18540 | AT1G62360 | SHOOT MERISTEMLESS (STM) | - |
| CBT53231 | AT1G62360 | SHOOT MERISTEMLESS (STM) | - |
| CBT12702 | AT2G22540 | SHORT VEGETATIVE PHASE (SVP) | - |
| CBT31629 | AT1G64860 | sigma factor A (SIGA) | - |
| CBT31628 | AT1G64860 | sigma factor A (SIGA) | - |
| CBT4167 | AT5G24120 | sigma factor E (SIGE) | - |
| CBT24508 | AT1G03790 | SOMNUS (SOM) | - |
| CBT10057 | AT1G20980 | squamosa promoter binding protein-like 14 (SPL14) | - |
| CBT9762 | AT1G58100 | TCP family transcription factor | - |
| CBT29007 | AT4G18390 | TEOSINTE BRANCHED 1, cycloidea and PCF transcription factor 2 (TCP2) | - |
| CBT4793 | AT4G18390 | TEOSINTE BRANCHED 1, cycloidea and PCF transcription factor 2 (TCP2) | - |
| CBT4740 | AT5G61380 | TIMING OF CAB EXPRESSION 1 (TOC1) | - |
| CBT7397 | AT5G61380 | TIMING OF CAB EXPRESSION 1 (TOC1) | - |
| CBT25289 | AT5G46910 | Transcription factor jumonji (jmj) family protein / zinc finger (C5HC2 type) family protein | - |
| CBT11166 | AT1G76900 | tubby like protein 1 (TLP1) | - |
| CBT4597 | AT1G76900 | tubby like protein 1 (TLP1) | - |
| CBT8456 | AT4G00050 | unfertilized embryo sac 10 (UNE10) | - |
| CBT16886 | AT4G00050 | unfertilized embryo sac 10 (UNE10) | - |
| CBT29475 | AT5G59340 | WUSCHEL related homeobox 2 (WOX2) | - |
| CBT24800 | AT5G58620 | zinc finger (CCCH-type) family protein | - |
| CBT30566 | AT1G69600 | zinc finger homeodomain 1 (ZFHD1) | - |
| CBT16867 | AT1G69600 | zinc finger homeodomain 1 (ZFHD1) | - |
| CBT17661 | AT1G10480 | zinc finger protein 5 (ZFP5) | - |
| CBT26543 | AT2G41940 | zinc finger protein 8 (ZFP8) | - |
| CBT4104 | AT3G19580 | zinc-finger protein 2 (ZF2) | - |
| CBT51847 | AT2G46510 | ABA-inducible BHLH-type transcription factor (AIB) | bHLH |
| CBT52021 | AT3G05800 | AtBS1(activation-tagged BRI1 suppressor 1)-interacting factor 1 (AIF1) | bHLH |
| CBT21816 | AT1G72210 | basic helix-loop-helix (bHLH) DNA-binding superfamily protein | bHLH |
| CBT10034 | AT5G57150 | basic helix-loop-helix (bHLH) DNA-binding superfamily protein | bHLH |
| CBT22204 | AT2G42280 | basic helix-loop-helix (bHLH) DNA-binding superfamily protein | bHLH |
| CBT22390 | AT1G61660 | basic helix-loop-helix (bHLH) DNA-binding superfamily protein | bHLH |
| CBT3716 | AT1G05805 | basic helix-loop-helix (bHLH) DNA-binding superfamily protein | bHLH |
| CBT31336 | AT4G28800 | basic helix-loop-helix (bHLH) DNA-binding superfamily protein | bHLH |
| CBT13983 | AT4G36060 | basic Helix-Loop-Helix 11 (bHLH11) | bHLH |
| CBT52524 | AT3G25710 | basic helix-loop-helix 32 (BHLH32) | BHLH |
| CBT42731 | AT1G69010 | BES1-interacting Myc-like protein 2 (BIM2) | bHLH |
| CBT52468 | AT4G30980 | LJRHL1-like 2 (LRL2) | bHLH |
| CBT19432 | AT1G02340 | LONG HYPOCOTYL IN FAR-RED (HFR1) | bHLH |
| CBT47842 | AT3G59060 | phytochrome interacting factor 3-like 6 (PIL6) | bHLH |
| CBT7275 | AT4G36930 | SPATULA (SPT) | bHLH |
| CBT24171 | AT3G19290 | ABRE binding factor 4 (ABF4) | bZIP |
| CBT5612 | AT5G49450 | basic leucine-zipper 1 (bZIP1) | bZIP |
| CBT13071 | AT4G37730 | basic leucine-zipper 7 (bZIP7) | bZIP |
| CBT17462 | AT3G62420 | basic region/leucine zipper motif 53 (BZIP53) | bZIP |
| CBT15426 | AT1G42990 | basic region/leucine zipper motif 60 (BZIP60) | bZIP |
| CBT17676 | AT1G42990 | basic region/leucine zipper motif 60 (BZIP60) | bZIP |
| CBT11523 | AT1G19490 | Basic-leucine zipper (bZIP) transcription factor family protein | bZIP |
| CBT6903 | AT5G06839 | bZIP transcription factor family protein | bZIP |
| CBT7611 | AT4G35040 | bZIP19 | bZIP |
| CBT5478 | AT3G44460 | DPBF2 | bZIP |
| CBT5874 | AT5G11260 | ELONGATED HYPOCOTYL 5 (HY5) | bZIP |
| CBT21859 | AT2G41070 | ENHANCED EM LEVEL (EEL) | bZIP |
| CBT1627 | AT2G46270 | G-box binding factor 3 (GBF3) | bZIP |
| CBT12623 | AT4G34590 | G-box binding factor 6 (GBF6) | bZIP |
| CBT6902 | AT5G64750 | ABA REPRESSOR1 (ABR1) | ERF |
| CBT5309 | AT5G57390 | AINTEGUMENTA-like 5 (AIL5) | ERF |
| CBT18269 | AT5G10510 | AINTEGUMENTA-like 6 (AIL6) | ERF |
| CBT24117 | AT3G11580 | AP2/B3-like transcriptional factor family protein | ERF |
| CBT21857 | AT5G17430 | BABY BOOM (BBM) | ERF |
| CBT22669 | AT5G17430 | BABY BOOM (BBM) | ERF |
| CBT17077 | AT5G53290 | cytokinin response factor 3 (CRF3) | ERF |
| CBT16658 | AT2G46310 | cytokinin response factor 5 (CRF5) | ERF |
| CBT16707 | AT3G61630 | cytokinin response factor 6 (CRF6) | ERF |
| CBT13358 | AT3G11020 | DRE/CRT-binding protein 2B (DREB2B) | ERF |
| CBT4791 | AT5G67190 | DREB and EAR motif protein 2 (DEAR2) | ERF |
| CBT28969 | AT5G05410 | DRE-binding protein 2A (DREB2A) | ERF |
| CBT24358 | AT5G05410 | DRE-binding protein 2A (DREB2A) | ERF |
| CBT11409 | AT5G05410 | DRE-binding protein 2A (DREB2A) | ERF |
| CBT28524 | AT5G44210 | erf domain protein 9 (ERF9) | ERF |
| CBT19193 | AT4G17500 | ethylene responsive element binding factor 1 (ERF-1) | ERF |
| CBT7712 | AT4G17500 | ethylene responsive element binding factor 1 (ERF-1) | ERF |
| CBT23167 | AT5G47220 | ethylene responsive element binding factor 2 (ERF2) | ERF |
| CBT53025 | AT5G47230 | ethylene responsive element binding factor 5 (ERF5) | ERF |
| CBT25640 | AT5G47230 | ethylene responsive element binding factor 5 (ERF5) | ERF |
| CBT7419 | AT1G64380 | Integrase-type DNA-binding superfamily protein | ERF |
| CBT19931 | AT3G16280 | Integrase-type DNA-binding superfamily protein | ERF |
| CBT29537 | AT2G33710 | Integrase-type DNA-binding superfamily protein | ERF |
| CBT23041 | AT2G38340 | Integrase-type DNA-binding superfamily protein | ERF |
| CBT22824 | AT1G21910 | Integrase-type DNA-binding superfamily protein | ERF |
| CBT5588 | AT4G39780 | Integrase-type DNA-binding superfamily protein | ERF |
| CBT22908 | AT1G22810 | Integrase-type DNA-binding superfamily protein | ERF |
| CBT24192 | AT1G79700 | Integrase-type DNA-binding superfamily protein | ERF |
| CBT9908 | AT5G61890 | Integrase-type DNA-binding superfamily protein | ERF |
| CBT17191 | AT1G66390 | myb domain protein 90 (MYB90) | ERF |
| CBT11643 | AT1G13260 | related to ABI3/VP1 1 (RAV1) | ERF |
| CBT12423 | AT4G36900 | related to AP2 10 (RAP2.10) | ERF |
| CBT12589 | AT3G14230 | related to AP2 2 (RAP2.2) | ERF |
| CBT6695 | AT5G13330 | related to AP2 6l (Rap2.6L) | ERF |
| CBT27899 | AT2G28550 | related to AP2.7 (RAP2.7) | ERF |
| CBT5818 | AT4G36710 | GRAS family transcription factor | GRAS |
| CBT22599 | AT2G37650 | GRAS family transcription factor | GRAS |
| CBT16394 | AT5G41920 | GRAS family transcription factor | GRAS |
| CBT45547 | AT4G17230 | SCARECROW-like 13 (SCL13) | GRAS |
| CBT23407 | AT4G37650 | SHORT ROOT (SHR) | GRAS |
| CBT7141 | AT4G11660 | AT-HSFB2B | HSF |
| CBT24011 | AT5G16820 | heat shock factor 3 (HSF3) | HSF |
| CBT18775 | AT4G36990 | heat shock factor 4 (HSF4) | HSF |
| CBT28562 | AT4G18880 | heat shock transcription factor A4A (HSF A4A) | HSF |
| CBT40014 | AT4G18880 | heat shock transcription factor A4A (HSF A4A) | HSF |
| CBT11511 | AT1G67970 | heat shock transcription factor A8 (HSFA8) | HSF |
| CBT14950 | AT5G62020 | heat shock transcription factor B2A (HSFB2A) | HSF |
| CBT15736 | AT3G24520 | heat shock transcription factor C1 (HSFC1) | HSF |
| CBT1251 | AT3G02990 | heat shock transcription factor A1E (HSFA1E) | HSF |
| CBT937 | AT5G03720 | heat shock transcription factor A3 (HSFA3) | HSF |
| CBT5323 | AT5G04760 | Duplicated homeodomain-like superfamily protein | MYB |
| CBT11758 | AT5G28300 | Duplicated homeodomain-like superfamily protein | MYB |
| CBT9904 | AT5G04760 | Duplicated homeodomain-like superfamily protein | MYB |
| CBT11801 | AT5G05090 | Homeodomain-like superfamily protein | MYB |
| CBT22603 | AT2G40260 | Homeodomain-like superfamily protein | MYB |
| CBT1072 | AT3G10760 | Homeodomain-like superfamily protein | MYB |
| CBT4082 | AT1G01520 | Homeodomain-like superfamily protein | MYB |
| CBT23312 | AT5G01380 | Homeodomain-like superfamily protein | MYB |
| CBT1419 | AT2G40260 | Homeodomain-like superfamily protein | MYB |
| CBT27453 | AT5G47660 | Homeodomain-like superfamily protein | MYB |
| CBT14598 | AT5G45580 | Homeodomain-like superfamily protein | MYB |
| CBT28599 | AT2G38250 | Homeodomain-like superfamily protein | MYB |
| CBT20454 | AT4G17695 | KANADI 3 (KAN3) | MYB |
| CBT16800 | AT4G17695 | KANADI 3 (KAN3) | MYB |
| CBT10926 | AT1G01060 | LATE ELONGATED HYPOCOTYL (LHY) | MYB |
| CBT16139 | AT1G01060 | LATE ELONGATED HYPOCOTYL (LHY) | MYB |
| CBT14428 | AT3G06490 | myb domain protein 108 (MYB108) | MYB |
| CBT22122 | AT3G55730 | myb domain protein 109 (MYB109) | MYB |
| CBT847 | AT3G62610 | myb domain protein 11 (MYB11) | MYB |
| CBT28806 | AT5G49330 | myb domain protein 111 (MYB111) | MYB |
| CBT28759 | AT1G74080 | myb domain protein 122 (MYB122) | MYB |
| CBT11047 | AT3G23250 | myb domain protein 15 (MYB15) | MYB |
| CBT912 | AT3G23250 | myb domain protein 15 (MYB15) | MYB |
| CBT13188 | AT2G47190 | myb domain protein 2 (MYB2) | MYB |
| CBT6575 | AT1G22640 | myb domain protein 3 (MYB3) | MYB |
| CBT14210 | AT1G74650 | myb domain protein 31 (MYB31) | MYB |
| CBT11668 | AT1G74650 | myb domain protein 31 (MYB31) | MYB |
| CBT17359 | AT4G34990 | myb domain protein 32 (MYB32) | MYB |
| CBT12987 | AT4G38620 | myb domain protein 4 (MYB4) | MYB |
| CBT26829 | AT5G54230 | myb domain protein 49 (MYB49) | MYB |
| CBT17461 | AT1G18570 | myb domain protein 51 (MYB51) | MYB |
| CBT29453 | AT4G01680 | myb domain protein 55 (MYB55) | MYB |
| CBT15796 | AT4G37260 | myb domain protein 73 (MYB73) | MYB |
| CBT5847 | AT5G62470 | myb domain protein 96 (MYB96) | MYB |
| CBT970 | AT5G67300 | myb domain protein r1 (MYBR1) | MYB |
| CBT42935 | AT4G21440 | MYB-like 102 (MYB102) | MYB |
| CBT23127 | AT3G46640 | PHYTOCLOCK 1 (PCL1) | MYB |
| CBT1439 | AT5G17300 | REVEILLE 1 (RVE1) | MYB |
| CBT6049 | AT5G18270 | Arabidopsis NAC domain containing protein 87 (ANAC087) | NAC |
| CBT47829 | AT1G01720 | ATAF1 | NAC |
| CBT11397 | AT1G01720 | ATAF1 | NAC |
| CBT9249 | AT3G15170 | CUP-SHAPED COTYLEDON1 (CUC1) | NAC |
| CBT8945 | AT3G15170 | CUP-SHAPED COTYLEDON1 (CUC1) | NAC |
| CBT11217 | AT1G12260 | NAC 007 (NAC007) | NAC |
| CBT14791 | AT3G15510 | NAC domain containing protein 2 (NAC2) | NAC |
| CBT18302 | AT5G39610 | NAC domain containing protein 6 (NAC6) | NAC |
| CBT21852 | AT1G01010 | NAC domain containing protein 1 (NAC001) | NAC |
| CBT7248 | AT1G56010 | NAC domain containing protein 1 (NAC1) | NAC |
| CBT8838 | AT1G28470 | NAC domain containing protein 10 (NAC010) | NAC |
| CBT489 | AT5G63790 | NAC domain containing protein 102 (NAC102) | NAC |
| CBT12609 | AT5G66300 | NAC domain containing protein 105 (NAC105) | NAC |
| CBT13872 | AT1G52890 | NAC domain containing protein 19 (NAC019) | NAC |
| CBT47972 | AT3G29035 | NAC domain containing protein 3 (NAC3) | NAC |
| CBT29340 | AT1G77450 | NAC domain containing protein 32 (NAC032) | NAC |
| CBT22561 | AT1G77450 | NAC domain containing protein 32 (NAC032) | NAC |
| CBT13280 | AT3G04060 | NAC domain containing protein 46 (NAC046) | NAC |
| CBT8667 | AT3G04060 | NAC domain containing protein 46 (NAC046) | NAC |
| CBT11543 | AT3G04070 | NAC domain containing protein 47 (NAC047) | NAC |
| CBT25431 | AT3G04070 | NAC domain containing protein 47 (NAC047) | NAC |
| CBT28372 | AT3G49530 | NAC domain containing protein 62 (NAC062) | NAC |
| CBT13392 | AT4G01550 | NAC domain containing protein 69 (NAC069) | NAC |
| CBT21751 | AT4G01550 | NAC domain containing protein 69 (NAC069) | NAC |
| CBT5590 | AT5G07680 | NAC domain containing protein 80 (NAC080) | NAC |
| CBT3894 | AT1G69490 | NAC-like, activated by AP3/PI (NAP) | NAC |
| CBT17081 | AT1G52880 | NO APICAL MERISTEM (NAM) | NAC |
| CBT2941 | AT4G27410 | RESPONSIVE TO DESICCATION 26 (RD26) | NAC |
| CBT11729 | AT4G27410 | RESPONSIVE TO DESICCATION 26 (RD26) | NAC |
| CBT28815 | AT1G71930 | vascular related NAC-domain protein 7 (VND7) | NAC |
| CBT8632 | AT2G37260 | TRANSPARENT TESTA GLABRA 2 (TTG2) | WRKY |
| CBT13771 | AT4G31550 | WRKY DNA-binding protein 11 (WRKY11) | WRKY |
| CBT27300 | AT4G31550 | WRKY DNA-binding protein 11 (WRKY11) | WRKY |
| CBT10815 | AT4G39410 | WRKY DNA-binding protein 13 (WRKY13) | WRKY |
| CBT29355 | AT1G30650 | WRKY DNA-binding protein 14 (WRKY14) | WRKY |
| CBT29423 | AT1G30650 | WRKY DNA-binding protein 14 (WRKY14) | WRKY |
| CBT9641 | AT2G24570 | WRKY DNA-binding protein 17 (WRKY17) | WRKY |
| CBT38517 | AT2G47260 | WRKY DNA-binding protein 23 (WRKY23) | WRKY |
| CBT274 | AT2G30250 | WRKY DNA-binding protein 25 (WRKY25) | WRKY |
| CBT275 | AT2G30250 | WRKY DNA-binding protein 25 (WRKY25) | WRKY |
| CBT6370 | AT5G52830 | WRKY DNA-binding protein 27 (WRKY27) | WRKY |
| CBT1335 | AT4G18170 | WRKY DNA-binding protein 28 (WRKY28) | WRKY |
| CBT25906 | AT5G24110 | WRKY DNA-binding protein 30 (WRKY30) | WRKY |
| CBT52460 | AT4G22070 | WRKY DNA-binding protein 31 (WRKY31) | WRKY |
| CBT5027 | AT4G22070 | WRKY DNA-binding protein 31 (WRKY31) | WRKY |
| CBT24703 | AT2G38470 | WRKY DNA-binding protein 33 (WRKY33) | WRKY |
| CBT15170 | AT1G69810 | WRKY DNA-binding protein 36 (WRKY36) | WRKY |
| CBT6024 | AT1G13960 | WRKY DNA-binding protein 4 (WRKY4) | WRKY |
| CBT6023 | AT1G13960 | WRKY DNA-binding protein 4 (WRKY4) | WRKY |
| CBT24621 | AT1G80840 | WRKY DNA-binding protein 40 (WRKY40) | WRKY |
| CBT18427 | AT5G49520 | WRKY DNA-binding protein 48 (WRKY48) | WRKY |
| CBT9972 | AT1G69310 | WRKY DNA-binding protein 57 (WRKY57) | WRKY |
| CBT13173 | AT5G01900 | WRKY DNA-binding protein 62 (WRKY62) | WRKY |
| CBT3549 | AT1G29280 | WRKY DNA-binding protein 65 (WRKY65) | WRKY |
| CBT13117 | AT3G58710 | WRKY DNA-binding protein 69 (WRKY69) | WRKY |
| CBT23166 | AT3G56400 | WRKY DNA-binding protein 70 (WRKY70) | WRKY |
| CBT14358 | AT3G56400 | WRKY DNA-binding protein 70 (WRKY70) | WRKY |
| CBT990 | AT5G15130 | WRKY DNA-binding protein 72 (WRKY72) | WRKY |
| CBT18049 | AT5G46350 | WRKY DNA-binding protein 8 (WRKY8) | WRKY |
| CBT24867 | AT4G01250 | WRKY22 | WRKY |
| CBT27588 | AT4G23550 | WRKY29 | WRKY |
| CBT19553 | AT4G04450 | WRKY42 | WRKY |
| CBT18769 | AT4G01720 | WRKY47 | WRKY |
| CBT5374 | AT4G23810 | WRKY53 | WRKY |
| CBT4734 | AT1G62300 | WRKY6 | WRKY |
