# Supplementary material for: Deep-sequencing transcriptome analysis of chilling tolerance mechanisms of a subnival alpine plant, Chorispora bungeana
Source: BMC Plant Biol. 2012 Nov 21;12:222. doi: 10.1186/1471-2229-12-222 (PMC3571968; doi:10.1186/1471-2229-12-222)
Supplement: Additional file 5 — List of chilling up-regulated protein serine/threonine kinase in C. bungeana. [file 1471-2229-12-222-S5.docx]

| **Unigene** | **AGI model** | **Description** | **Family** |
| --- | --- | --- | --- |
| CBT7411 | AT1G66910 | Protein kinase superfamily protein | - |
| CBT24467 | AT1G51800 | Leucine-rich repeat protein kinase family protein | - |
| CBT26019 | AT4G17660 | Protein kinase superfamily protein | - |
| CBT24058 | AT4G18950 | Integrin-linked protein kinase family | - |
| CBT46973 | AT4G33430 | BRI1-associated receptor kinase (BAK1) | - |
| CBT16405 | AT3G17410 | Protein kinase superfamily protein | - |
| CBT23891 | AT5G66790 | Protein kinase superfamily protein | - |
| CBT20344 | AT3G57710 | Protein kinase superfamily protein | - |
| CBT29017 | AT3G25250 | AGC2-1 | - |
| CBT28862 | AT1G49160 | WNK7 | - |
| CBT18557 | AT4G18250 | receptor serine/threonine kinase, putative | - |
| CBT6094 | AT3G57710 | Protein kinase superfamily protein | - |
| CBT7609 | AT3G24190 | Protein kinase superfamily protein | - |
| CBT4379 | AT5G63370 | Protein kinase superfamily protein | - |
| CBT23455 | AT3G57120 | Protein kinase superfamily protein | - |
| CBT18068 | AT5G38280 | PR5-like receptor kinase (PR5K) | - |
| CBT38786 | AT1G67470 | Protein kinase superfamily protein | - |
| CBT27388 | AT1G25390 | Protein kinase superfamily protein | - |
| CBT20499 | AT3G57120 | Protein kinase superfamily protein | - |
| CBT18194 | AT5G10440 | cyclin d4 | - |
| CBT13563 | AT1G79680 | WALL ASSOCIATED KINASE (WAK)-LIKE 10 (WAKL10) | - |
| CBT18817 | AT2G23770 | protein kinase family protein / peptidoglycan-binding LysM domain-containing protein | - |
| CBT27731 | AT5G46080 | Protein kinase superfamily protein | - |
| CBT41605 | AT5G01540 | lectin receptor kinase a4.1 (LECRKA4.1) | - |
| CBT19197 | AT3G08730 | protein-serine kinase 1 (PK1) | - |
| CBT48326 | AT4G38470 | ACT-like protein tyrosine kinase family protein | - |
| CBT24806 | AT1G66880 | Protein kinase superfamily protein | - |
| CBT24238 | AT1G79680 | WALL ASSOCIATED KINASE (WAK)-LIKE 10 (WAKL10) | - |
| CBT4484 | AT2G02710 | PAS/LOV protein B (PLPB) | - |
| CBT46995 | AT3G14370 | WAG2 | - |
| CBT18314 | AT1G03740 | Protein kinase superfamily protein | - |
| CBT17982 | AT1G16130 | wall associated kinase-like 2 (WAKL2) | - |
| CBT4203 | AT4G33430 | BRI1-associated receptor kinase (BAK1) | - |
| CBT9404 | AT4G17660 | Protein kinase superfamily protein | - |
| CBT51806 | AT3G57710 | Protein kinase superfamily protein | - |
| CBT24250 | AT5G13160 | avrPphB susceptible 1 (PBS1) | - |
| CBT29280 | AT3G25250 | AGC2-1 | - |
| CBT47653 | AT5G61560 | U-box domain-containing protein kinase family protein | - |
| CBT6903 | AT5G06839 | bZIP transcription factor family protein | - |
| CBT49903 | AT3G09240 | Protein kinase protein with tetratricopeptide repeat domain | - |
| CBT9941 | AT3G08730 | protein-serine kinase 1 (PK1) | - |
| CBT5267 | AT5G35580 | Protein kinase superfamily protein | - |
| CBT20038 | AT2G26290 | root-specific kinase 1 (ARSK1) | - |
| CBT17942 | AT5G45190 | Cyclin family protein | - |
| CBT17941 | AT5G45190 | Cyclin family protein | - |
| CBT42840 | AT2G47060 | Protein kinase superfamily protein | - |
| CBT9528 | AT5G55560 | Protein kinase superfamily protein | - |
| CBT42757 | AT5G22050 | Protein kinase superfamily protein | - |
| CBT6636 | AT1G14370 | protein kinase 2A (APK2A) | - |
| CBT24135 | AT2G44680 | casein kinase II beta subunit 4 (CKB4) | - |
| CBT14018 | AT1G53165 | ATMAP4K ALPHA1 | - |
| CBT7492 | AT2G34650 | PINOID (PID) | - |
| CBT12210 | AT1G76360 | Protein kinase superfamily protein | - |
| CBT38759 | AT1G63500 | Protein kinase protein with tetratricopeptide repeat domain | - |
| CBT42443 | AT3G15354 | SPA1-related 3 (SPA3) | - |
| CBT7398 | AT1G53050 | Protein kinase superfamily protein | - |
| CBT821 | AT3G55950 | CRINKLY4 related 3 (CCR3) | - |
| CBT48238 | AT1G11440 | BEST Arabidopsis thaliana protein match is: glycine-rich protein (TAIR:AT3G29075.1) | - |
| CBT5293 | AT1G62400 | high leaf temperature 1 (HT1) | - |
| CBT24295 | AT2G37710 | receptor lectin kinase (RLK) | - |
| CBT28445 | AT3G57120 | Protein kinase superfamily protein | - |
| CBT22916 | AT5G01540 | lectin receptor kinase a4.1 (LECRKA4.1) | - |
| CBT16536 | AT2G39660 | botrytis-induced kinase1 (BIK1) | - |
| CBT22314 | AT1G30640 | Protein kinase family protein | - |
| CBT22052 | AT2G02220 | phytosulfokin receptor 1 (PSKR1) | - |
| CBT7276 | AT3G46160 | Protein kinase superfamily protein | - |
| CBT22051 | AT2G46340 | SUPPRESSOR OF PHYA-105 1 (SPA1) | - |
| CBT18772 | AT5G63370 | Protein kinase superfamily protein | - |
| CBT13104 | AT1G79680 | WALL ASSOCIATED KINASE (WAK)-LIKE 10 (WAKL10) | - |
| CBT13301 | AT3G22750 | Protein kinase superfamily protein | - |
| CBT45525 | AT1G63500 | Protein kinase protein with tetratricopeptide repeat domain | - |
| CBT28324 | AT1G25390 | Protein kinase superfamily protein | - |
| CBT11893 | AT2G24360 | Protein kinase superfamily protein | - |
| CBT8533 | AT2G37710 | receptor lectin kinase (RLK) | - |
| CBT42297 | AT3G59350 | Protein kinase superfamily protein | - |
| CBT30488 | AT2G16750 | Protein kinase protein with adenine nucleotide alpha hydrolases-like domain | - |
| CBT861 | AT3G19300 | Protein kinase superfamily protein | - |
| CBT20493 | AT3G59700 | lectin-receptor kinase (HLECRK) | - |
| CBT7113 | AT3G59700 | lectin-receptor kinase (HLECRK) | - |
| CBT18655 | AT1G76040 | calcium-dependent protein kinase 29 (CPK29) | CDPK |
| CBT13562 | AT1G50700 | calcium-dependent protein kinase 33 (CPK33) | CDPK |
| CBT17411 | AT4G35310 | calmodulin-domain protein kinase 5 (CPK5) | CDPK |
| CBT20357 | AT2G38490 | CBL-interacting protein kinase 22 (CIPK22) | CIPK/SnRK |
| CBT15061 | AT2G34180 | CBL-interacting protein kinase 13 (CIPK13) | CIPK/SnRK |
| CBT7489 | AT4G18700 | CBL-interacting protein kinase 12 (CIPK12) | CIPK/SnRK |
| CBT18847 | AT5G57630 | CBL-interacting protein kinase 21 (CIPK21) | CIPK/SnRK |
| CBT38924 | AT3G23000 | CBL-interacting protein kinase 7 (CIPK7) | CIPK/SnRK |
| CBT10378 | AT1G01140 | CBL-interacting protein kinase 9 (CIPK9) | CIPK/SnRK |
| CBT28945 | AT4G14580 | CBL-interacting protein kinase 4 (CIPK4) | CIPK/SnRK |
| CBT4701 | AT2G30360 | SOS3-interacting protein 4 (SIP4) | CIPK/SnRK |
| CBT12855 | AT4G40010 | SNF1-related protein kinase 2.7 (SNRK2.7) | CIPK/SnRK |
| CBT13105 | AT2G38490 | CBL-interacting protein kinase 22 (CIPK22) | CIPK/SnRK |
| CBT3872 | AT1G78290 | Protein kinase superfamily protein | CIPK/SnRK |
| CBT16604 | AT4G04540 | cysteine-rich RLK (RECEPTOR-like protein kinase) 39 (CRK39) | CRK |
| CBT12676 | AT4G23190 | cysteine-rich RLK (RECEPTOR-like protein kinase) 11 (CRK11) | CRK |
| CBT10986 | AT4G23160 | cysteine-rich RLK (RECEPTOR-like protein kinase) 8 (CRK8) | CRK |
| CBT6773 | AT4G23180 | cysteine-rich RLK (RECEPTOR-like protein kinase) 10 (CRK10) | CRK |
| CBT4245 | AT1G70520 | cysteine-rich RLK (RECEPTOR-like protein kinase) 2 (CRK2) | CRK |
| CBT9455 | AT4G21410 | cysteine-rich RLK (RECEPTOR-like protein kinase) 29 (CRK29) | CRK |
| CBT39403 | AT4G23270 | cysteine-rich RLK (RECEPTOR-like protein kinase) 19 (CRK19) | CRK |
| CBT28648 | AT4G11530 | cysteine-rich RLK (RECEPTOR-like protein kinase) 34 (CRK34) | CRK |
| CBT39406 | AT4G23130 | cysteine-rich RLK (RECEPTOR-like protein kinase) 5 (CRK5) | CRK |
| CBT18973 | AT4G11480 | cysteine-rich RLK (RECEPTOR-like protein kinase) 32 (CRK32) | CRK |
| CBT14418 | AT4G23130 | cysteine-rich RLK (RECEPTOR-like protein kinase) 5 (CRK5) | CRK |
| CBT10942 | AT4G23190 | cysteine-rich RLK (RECEPTOR-like protein kinase) 11 (CRK11) | CRK |
| CBT28398 | AT4G11470 | cysteine-rich RLK (RECEPTOR-like protein kinase) 31 (CRK31) | CRK |
| CBT24032 | AT4G04490 | cysteine-rich RLK (RECEPTOR-like protein kinase) 36 (CRK36) | CRK |
| CBT7304 | AT4G23160 | cysteine-rich RLK (RECEPTOR-like protein kinase) 8 (CRK8) | CRK |
| CBT42899 | AT4G23250 | EMBRYO DEFECTIVE 1290 (EMB1290) | CRK |
| CBT13438 | AT4G23130 | cysteine-rich RLK (RECEPTOR-like protein kinase) 5 (CRK5) | CRK |
| CBT18700 | AT4G23180 | cysteine-rich RLK (RECEPTOR-like protein kinase) 10 (CRK10) | CRK |
| CBT29485 | AT4G04540 | cysteine-rich RLK (RECEPTOR-like protein kinase) 39 (CRK39) | CRK |
| CBT24529 | AT4G23160 | cysteine-rich RLK (RECEPTOR-like protein kinase) 8 (CRK8) | CRK |
| CBT6904 | AT4G23200 | cysteine-rich RLK (RECEPTOR-like protein kinase) 12 (CRK12) | CRK |
| CBT52038 | AT4G23230 | cysteine-rich RLK (RECEPTOR-like protein kinase) 15 (CRK15) | CRK |
| CBT24130 | AT4G23180 | cysteine-rich RLK (RECEPTOR-like protein kinase) 10 (CRK10) | CRK |
| CBT5637 | AT4G23220 | cysteine-rich RLK (RECEPTOR-like protein kinase) 14 (CRK14) | CRK |
| CBT47485 | AT4G04490 | cysteine-rich RLK (RECEPTOR-like protein kinase) 36 (CRK36) | CRK |
| CBT25446 | AT4G23160 | cysteine-rich RLK (RECEPTOR-like protein kinase) 8 (CRK8) | CRK |
| CBT12854 | AT4G23130 | cysteine-rich RLK (RECEPTOR-like protein kinase) 5 (CRK5) | CRK |
| CBT19097 | AT4G23190 | cysteine-rich RLK (RECEPTOR-like protein kinase) 11 (CRK11) | CRK |
| CBT21279 | AT4G23160 | cysteine-rich RLK (RECEPTOR-like protein kinase) 8 (CRK8) | CRK |
| CBT6144 | AT4G23160 | cysteine-rich RLK (RECEPTOR-like protein kinase) 8 (CRK8) | CRK |
| CBT24892 | AT4G23160 | cysteine-rich RLK (RECEPTOR-like protein kinase) 8 (CRK8) | CRK |
| CBT45798 | AT4G23150 | cysteine-rich RLK (RECEPTOR-like protein kinase) 7 (CRK7) | CRK |
| CBT24 | AT4G23160 | cysteine-rich RLK (RECEPTOR-like protein kinase) 8 (CRK8) | CRK |
| CBT23 | AT4G23160 | cysteine-rich RLK (RECEPTOR-like protein kinase) 8 (CRK8) | CRK |
| CBT26 | AT4G23160 | cysteine-rich RLK (RECEPTOR-like protein kinase) 8 (CRK8) | CRK |
| CBT25 | AT4G23160 | cysteine-rich RLK (RECEPTOR-like protein kinase) 8 (CRK8) | CRK |
| CBT9562 | AT1G61390 | S-locus lectin protein kinase family protein | lectin protein kinase |
| CBT19021 | AT1G73080 | PEP1 receptor 1 (PEPR1) | lectin protein kinase |
| CBT1067 | AT1G11300 | protein serine/threonine kinases | lectin protein kinase |
| CBT18137 | AT1G61610 | S-locus lectin protein kinase family protein | lectin protein kinase |
| CBT12680 | AT5G35370 | S-locus lectin protein kinase family protein | lectin protein kinase |
| CBT13283 | AT2G32800 | AP4.3A | lectin protein kinase |
| CBT16951 | AT5G55830 | Concanavalin A-like lectin protein kinase family protein | lectin protein kinase |
| CBT10558 | AT1G15530 | Concanavalin A-like lectin protein kinase family protein | lectin protein kinase |
| CBT28623 | AT4G11900 | S-locus lectin protein kinase family protein | lectin protein kinase |
| CBT14911 | AT5G60270 | Concanavalin A-like lectin protein kinase family protein | lectin protein kinase |
| CBT22745 | AT4G28350 | Concanavalin A-like lectin protein kinase family protein | lectin protein kinase |
| CBT21164 | AT2G19130 | S-locus lectin protein kinase family protein | lectin protein kinase |
| CBT17733 | AT1G61380 | S-domain-1 29 (SD1-29) | lectin protein kinase |
| CBT17732 | AT1G61370 | S-locus lectin protein kinase family protein | lectin protein kinase |
| CBT38744 | AT1G61360 | S-locus lectin protein kinase family protein | lectin protein kinase |
| CBT17729 | AT1G61360 | S-locus lectin protein kinase family protein | lectin protein kinase |
| CBT17728 | AT1G61360 | S-locus lectin protein kinase family protein | lectin protein kinase |
| CBT46815 | AT1G61380 | S-domain-1 29 (SD1-29) | lectin protein kinase |
| CBT11754 | AT1G61360 | S-locus lectin protein kinase family protein | lectin protein kinase |
| CBT34579 | AT1G73080 | PEP1 receptor 1 (PEPR1) | lectin protein kinase |
| CBT24138 | AT5G03140 | Concanavalin A-like lectin protein kinase family protein | lectin protein kinase |
| CBT24298 | AT5G60270 | Concanavalin A-like lectin protein kinase family protein | lectin protein kinase |
| CBT22915 | AT1G73080 | PEP1 receptor 1 (PEPR1) | lectin protein kinase |
| CBT8136 | AT1G61480 | S-locus lectin protein kinase family protein | lectin protein kinase |
| CBT20400 | AT4G21390 | B120 | lectin protein kinase |
| CBT16259 | AT2G19130 | S-locus lectin protein kinase family protein | lectin protein kinase |
| CBT30622 | AT1G61380 | S-domain-1 29 (SD1-29) | lectin protein kinase |
| CBT2025 | AT1G51820 | Leucine-rich repeat protein kinase family protein | LRR |
| CBT18855 | AT2G23300 | Leucine-rich repeat protein kinase family protein | LRR |
| CBT11930 | AT1G51820 | Leucine-rich repeat protein kinase family protein | LRR |
| CBT11932 | AT1G51820 | Leucine-rich repeat protein kinase family protein | LRR |
| CBT16204 | AT5G20480 | EF-TU receptor (EFR) | LRR |
| CBT39432 | AT1G27190 | Leucine-rich repeat protein kinase family protein | LRR |
| CBT13403 | AT1G66830 | Leucine-rich repeat protein kinase family protein | LRR |
| CBT18668 | AT1G51820 | Leucine-rich repeat protein kinase family protein | LRR |
| CBT29109 | AT4G39270 | Leucine-rich repeat protein kinase family protein | LRR |
| CBT22083 | AT1G09970 | LRR XI-23 | LRR |
| CBT26000 | AT1G17230 | Leucine-rich receptor-like protein kinase family protein | LRR |
| CBT5830 | AT2G23300 | Leucine-rich repeat protein kinase family protein | LRR |
| CBT24502 | AT5G63930 | Leucine-rich repeat protein kinase family protein | LRR |
| CBT10248 | AT5G25930 | Protein kinase family protein with leucine-rich repeat domain | LRR |
| CBT18831 | AT5G07280 | EXCESS MICROSPOROCYTES1 (EMS1) | LRR |
| CBT11929 | AT1G51820 | Leucine-rich repeat protein kinase family protein | LRR |
| CBT1258 | AT4G30520 | Leucine-rich repeat protein kinase family protein | LRR |
| CBT23424 | AT4G08850 | Leucine-rich repeat receptor-like protein kinase family protein | LRR |
| CBT20510 | AT1G35710 | Protein kinase family protein with leucine-rich repeat domain | LRR |
| CBT12879 | AT1G51790 | Leucine-rich repeat protein kinase family protein | LRR |
| CBT24984 | AT2G23950 | Leucine-rich repeat protein kinase family protein | LRR |
| CBT12659 | AT1G74360 | Leucine-rich repeat protein kinase family protein | LRR |
| CBT14995 | AT1G56130 | Leucine-rich repeat transmembrane protein kinase | LRR |
| CBT19230 | AT4G37250 | Leucine-rich repeat protein kinase family protein | LRR |
| CBT1529 | AT5G46330 | FLAGELLIN-SENSITIVE 2 (FLS2) | LRR |
| CBT12562 | AT1G51660 | mitogen-activated protein kinase kinase 4 (MKK4) | MKK |
| CBT21851 | AT1G73500 | MAP kinase kinase 9 (MKK9) | MKK |
| CBT30448 | AT4G01370 | MAP kinase 4 (MPK4) | MPK |
| CBT28144 | AT3G45640 | mitogen-activated protein kinase 3 (MPK3) | MPK |
| CBT10870 | AT1G53510 | mitogen-activated protein kinase 18 (MPK18) | MPK |
| CBT24126 | AT3G14720 | MAP kinase 19 (MPK19) | MPK |
| CBT24125 | AT3G14720 | MAP kinase 19 (MPK19) | MPK |
| CBT45413 | AT2G01450 | MAP kinase 17 (MPK17) | MPK |
| CBT1625 | AT2G18170 | MAP kinase 7 (MPK7) | MPK |
| CBT17108 | AT1G53510 | mitogen-activated protein kinase 18 (MPK18) | MPK |
| CBT11195 | AT4G01370 | MAP kinase 4 (MPK4) | MPK |
| CBT4532 | AT2G43790 | MAP kinase 6 (MPK6) | MPK |
| CBT5611 | AT5G67080 | mitogen-activated protein kinase kinase kinase 19 (MAPKKK19) | MPKKK |
| CBT13319 | AT1G07150 | mitogen-activated protein kinase kinase kinase 13 (MAPKKK13) | MPKKK |
| CBT24973 | AT2G30040 | mitogen-activated protein kinase kinase kinase 14 (MAPKKK14) | MPKKK |
| CBT16266 | AT5G66850 | mitogen-activated protein kinase kinase kinase 5 (MAPKKK5) | MPKKK |
| CBT15244 | AT1G34210 | somatic embryogenesis receptor-like kinase 2 (SERK2) | Other RLK |
| CBT18145 | AT1G69270 | receptor-like protein kinase 1 (RPK1) | Other RLK |
| CBT19812 | AT5G07180 | ERECTA-like 2 (ERL2) | Other RLK |
| CBT18057 | AT4G21380 | receptor kinase 3 (RK3) | Other RLK |
| CBT14744 | AT2G05940 | Protein kinase superfamily protein | Other RLK |
| CBT15963 | AT2G05940 | Protein kinase superfamily protein | Other RLK |
| CBT21539 | AT5G48380 | BAK1-interacting receptor-like kinase 1 (BIR1) | Other RLK |
| CBT18389 | AT5G39020 | Malectin/receptor-like protein kinase family protein | Other RLK |
| CBT28460 | AT1G68690 | Protein kinase superfamily protein | PERK |
| CBT23064 | AT5G38560 | Protein kinase superfamily protein | PERK |
| CBT5206 | AT3G24550 | proline extensin-like receptor kinase 1 (PERK1) | PERK |
| CBT45383 | AT1G68690 | Protein kinase superfamily protein | PERK |
| CBT12772 | AT5G38560 | Protein kinase superfamily protein | PERK |
| CBT6826 | AT1G68690 | Protein kinase superfamily protein | PERK |
| CBT6940 | AT1G23540 | INFLORESCENCE GROWTH INHIBITOR 1 (IGI1) | PERK |
