# Supplementary material for: Deep-sequencing transcriptome analysis of chilling tolerance mechanisms of a subnival alpine plant, Chorispora bungeana
Source: BMC Plant Biol. 2012 Nov 21;12:222. doi: 10.1186/1471-2229-12-222 (PMC3571968; doi:10.1186/1471-2229-12-222)
Supplement: Additional file 7 — List of chilling regulated DEGs and all expressed genes of ATH-MA. 1. List of chilling up-regulated DEGs (MA). 2. List of chilling down-regulated DEGs (MA). 3. List all expressed genes (MA). [file 1471-2229-12-222-S7.docx]

**1. List of chilling up-regulated DEGs (MA)**

AT5G42900, AT2G42540, AT5G62360, AT1G11210, AT1G51090, AT4G17090, AT4G33980, AT2G42530, AT1G09350, AT1G16850, AT1G09950, AT4G25490, AT4G25470, AT1G49720, AT3G22910, AT3G17130, AT2G22590, AT4G30650, AT2G02120, AT1G17615, AT5G20790, AT4G25480, AT5G48250, AT3G45970, AT4G01870, AT4G18280, AT1G48100, AT2G38465, AT1G73480, AT1G64890, AT4G22270, AT1G08900, AT1G08890, AT2G43500, AT5G40010, AT4G12000, AT1G01470, AT5G24470, AT3G61190, AT5G13200, AT5G48070, AT4G39670, AT5G57560, AT1G21670, AT4G24960, AT5G26340, AT3G57760, AT4G12480, AT4G14690, AT1G25400, AT5G54470, AT1G21790, AT5G04340, AT1G27730, AT2G02000, AT2G02010, AT1G02310, AT2G40080, AT2G26820, AT2G30400, AT4G36500, AT1G76590, AT4G16820, AT5G64870, AT5G37260, AT3G50970, AT1G17170, AT3G02380, AT1G56300, AT3G15990, AT4G15130, AT2G16700, AT4G31800, AT2G43620, AT4G38580, AT1G61340, AT5G40390, AT2G45560, AT5G65600, AT5G23220, AT2G17840, AT2G28900, AT1G62570, AT4G25380, AT1G21120, AT5G17300, AT3G12320, AT2G35960, AT1G27760, AT4G12430, AT4G12432, AT3G03640, AT1G20440, AT5G61560, AT1G21360, AT5G42570, AT1G53035, AT2G40140, AT5G55970, AT2G46830, AT3G55760, AT5G28080, AT5G05410, AT1G58420, AT2G31360, AT1G80110, AT5G51830, AT5G49450, AT5G49448, AT3G61890, AT2G22500, AT1G71950, AT5G65300, AT5G41610, AT1G80130, AT5G64620, AT2G18210, AT3G07350, AT5G39580, AT4G23920, AT2G04040, AT3G28340, AT2G28550, AT3G55940, AT5G49480, AT2G35710, AT5G61810, AT3G27880, AT1G47710, AT5G24110, AT2G22860, AT5G62350, AT1G09070, AT1G75860, AT1G36770, AT3G28530, AT2G33710, AT1G03905, AT5G57510, AT4G16860, AT1G30620, AT5G39760, AT1G70420, AT4G23450, AT4G27970, AT3G54150, AT5G61380, AT3G12700, AT5G06980, AT5G44260, AT5G64905, AT1G67970, AT1G30360, AT5G17050, AT4G22590, AT4G22592, AT2G26530, AT5G39050, AT2G20142, AT1G14540, AT1G04500, AT1G32920, AT3G50260, AT1G69570, AT3G49530, AT2G16500, AT1G76580, AT5G48410, AT5G61370, AT5G57220, AT4G35180, AT4G19200, AT5G57660, AT4G09110, AT5G13750, AT4G26080, AT2G33830, AT1G53430, AT1G30720, AT1G30730, AT1G02400, AT3G55740, AT4G15430, AT2G21620, AT5G58070, AT5G53590, AT5G53588, AT3G13720, AT5G20910, AT2G20680, AT3G56710, AT1G80380, AT1G51820, AT1G70920, AT2G23810, AT3G20520, AT1G19200, AT1G27910, AT3G57880, AT2G46790, AT2G46670, AT4G29190, AT2G39650, AT5G62630, AT1G55450, AT1G28600, AT4G38470, AT1G02270, AT3G13710, AT3G13700, AT3G44450, AT1G51700, AT3G47860, AT4G05020, AT5G47060, AT1G06000, AT5G04760, AT1G10410, AT3G05580, AT3G10300, AT1G33590, AT4G18070, AT5G49520, AT1G13930, AT5G01750, AT1G61740, AT3G50950, AT1G48000, AT3G61060, AT3G09520, AT1G06780, AT4G29440, AT4G26370, AT3G10500, AT2G38340, AT2G22080, AT2G22190, AT5G52660, AT4G34990, AT5G44110, AT4G16146, AT2G30250, AT2G26290, AT5G39040, AT3G05500, AT1G08930, AT2G31380, AT1G74840, AT1G27100, AT4G25810, AT5G07440, AT3G09920, AT5G12140, AT3G08710, AT5G05190, AT2G16365, AT1G18890, AT3G04060, AT5G13190, AT3G51550, AT3G27210, AT1G22770, AT3G13380, AT1G66880, AT4G02880, AT3G62690, AT1G13600, AT3G55470, AT1G30370, AT3G54990, AT5G56340, AT4G23630, AT1G11960, AT1G33420, AT5G07100, AT5G06300, AT4G20890, AT1G75410, AT3G47380, AT4G23870, AT3G62720, AT3G14230, AT5G43450, AT3G08720, AT5G06860, AT3G24190, AT1G53050, AT5G16040, AT3G05880, AT3G15890, AT5G42050, AT2G33820, AT5G01850, AT2G41180, AT1G58360, AT3G53260, AT1G43620, AT1G73540, AT5G47880, AT3G50410, AT1G01010, AT1G14870, AT1G14880, AT4G27940, AT5G47650, AT5G40780, AT3G14050, AT3G15450, AT5G57830, AT3G08760, AT3G61580, AT1G32870, AT5G37500, AT1G06250, AT3G23605, AT1G53920, AT5G62130, AT4G35500, AT4G21560, AT5G66210, AT1G76160, AT3G61260, AT3G21650, AT1G73730, AT3G15430, AT5G62020, AT3G45640, AT5G20730, AT2G17280, AT2G30140, AT3G59700, AT1G02930, AT1G02920, AT2G17270, AT3G23820, AT1G07570, AT3G13330, AT1G43860, AT2G44790, AT1G71050, AT5G57100, AT3G15310, AT4G01090, AT5G18470, AT1G78340, AT5G10510, AT5G56630, AT5G16980, AT1G08550, AT2G27260, AT2G18890, AT5G54840, AT3G45260, AT5G22630, AT2G28570, AT3G62700, AT3G03170, AT4G17140, AT3G44720, AT3G27290, AT3G24530, AT2G43320, AT1G71040, AT3G24840, AT2G39400, AT1G42990, AT5G42720, AT4G25650, AT1G19110, AT5G55380, AT2G40900, AT2G47550, AT1G17500, AT3G21690, AT4G02920, AT3G17510, AT2G12550, AT5G40170, AT3G13690, AT4G18090, AT3G11210, AT4G30790, AT5G66200, AT5G60920, AT5G14310, AT1G73650, AT1G32940, AT2G23340, AT2G47420, AT1G67850, AT4G17260, AT4G17720, AT4G17550, AT4G15770, AT4G15910, AT4G14580, AT4G14230, AT4G14270, AT1G25260, AT1G25320, AT1G28200, AT5G22060, AT5G04170, AT5G04250, AT1G73390, AT1G73500, AT1G30240, AT1G46768, AT1G26270, AT5G11090, AT5G28820, AT5G20830, AT5G20160, AT5G20180, AT5G20570, AT5G20230, AT3G56880, AT3G51895, AT5G17030, AT5G16840, AT5G15960, AT5G15970, AT5G16010, AT5G15950, AT5G15948, AT5G15850, AT5G15750, AT1G31660, AT5G28150, AT5G27520, AT5G27280, AT5G26770, AT5G26570, AT5G25760, AT5G26040, AT5G25540, AT5G67300, AT5G66540, AT5G66460, AT5G64080, AT5G64310, AT5G64260, AT5G63990, AT5G63790, AT5G63320, AT5G62920, AT5G62440, AT5G62470, AT5G61820, AT5G60680, AT5G60850, AT5G60540, AT5G59950, AT5G59820, AT5G59550, AT5G59240, AT5G58690, AT5G58700, AT5G58600, AT5G58650, AT5G58670, AT5G58620, AT5G58290, AT5G57280, AT5G57110, AT5G57050, AT5G55990, AT5G55850, AT5G56030, AT5G55450, AT5G55610, AT5G55400, AT5G55180, AT5G55200, AT5G54910, AT5G54960, AT5G52580, AT5G52820, AT5G52640, AT5G52310, AT5G52300, AT5G52040, AT5G50840, AT5G50360, AT5G49550, AT5G47240, AT5G46860, AT5G47090, AT5G46330, AT5G45750, AT5G44670, AT5G44730, AT5G43960, AT5G43440, AT5G42150, AT5G42010, AT5G41670, AT5G41460, AT5G40880, AT5G49200, AT5G40540, AT5G39570, AT5G38720, AT5G37780, AT5G35735, AT5G24570, AT5G23850, AT5G18525, AT5G18130, AT5G17760, AT5G17190, AT5G15190, AT5G14270, AT5G14520, AT5G14230, AT5G11740, AT5G11420, AT5G10790, AT5G10100, AT5G09590, AT5G08500, AT5G07920, AT5G08130, AT5G07060, AT5G07070, AT5G06550, AT5G05220, AT5G03200, AT5G01340, AT5G01200, AT3G62840, AT3G62660, AT3G62240, AT3G60360, AT3G59350, AT3G58660, AT3G58680, AT3G57340, AT3G57550, AT3G56090, AT2G39800, AT3G55610, AT3G55510, AT3G55430, AT3G54980, AT3G54820, AT3G54680, AT3G54400, AT3G53960, AT3G53990, AT3G53460, AT3G53620, AT3G52040, AT3G52500, AT3G50380, AT3G49220, AT3G49120, AT3G49110, AT3G48510, AT3G48250, AT3G47420, AT3G47160, AT3G46620, AT3G46440, AT3G45960, AT3G44735, AT4G39730, AT4G39260, AT4G39090, AT4G39270, AT4G39320, AT4G38380, AT4G38150, AT4G37470, AT4G36010, AT4G35940, AT4G35300, AT4G34910, AT4G34590, AT4G34588, AT4G34740, AT4G34120, AT4G33905, AT4G33920, AT4G33700, AT4G33300, AT4G32280, AT4G32190, AT4G32020, AT4G31290, AT4G31140, AT4G31160, AT4G30350, AT4G29950, AT4G29510, AT4G28703, AT4G28540, AT4G28720, AT4G28320, AT4G27820, AT4G27500, AT4G27410, AT4G27520, AT4G27720, AT4G27130, AT4G27380, AT4G26750, AT4G26670, AT4G25340, AT4G25640, AT4G25630, AT4G24990, AT4G24570, AT4G24220, AT4G24440, AT4G22330, AT4G22220, AT4G20480, AT4G20070, AT4G19390, AT4G18700, AT4G18240, AT4G18390, AT4G13640, AT4G13850, AT4G17950, AT4G18010, AT4G12750, AT4G12470, AT4G12280, AT4G12290, AT4G12040, AT4G11350, AT4G10960, AT4G10955, AT4G10620, AT4G10040, AT4G09570, AT4G09020, AT4G07410, AT4G05520, AT4G04860, AT4G04340, AT4G02370, AT4G02380, AT4G02710, AT4G02730, AT4G02400, AT4G02340, AT4G01560, AT4G01130, AT1G22070, AT1G22270, AT1G19180, AT1G58270, AT1G20693, AT1G20696, AT1G16840, AT1G13560, AT1G55110, AT3G12580, AT3G12270, AT5G43620, AT1G66500, AT3G06200, AT3G19960, AT3G13670, AT3G18600, AT3G22120, AT3G23990, AT3G14200, AT3G15210, AT3G15352, AT3G20500, AT3G27300, AT3G27260, AT3G14890, AT3G28007, AT3G01210, AT3G13810, AT3G16800, AT3G12670, AT3G23030, AT3G23000, AT3G25140, AT3G12980, AT3G17020, AT3G23170, AT3G20720, AT3G20810, AT3G19340, AT3G19360, AT3G14560, AT3G14595, AT3G24520, AT3G17820, AT3G15730, AT3G22840, AT3G22830, AT3G16180, AT3G15460, AT3G16740, AT3G22330, AT3G22310, AT3G06035, AT3G02555, AT3G06530, AT3G06500, AT3G04240, AT3G02750, AT3G02990, AT3G04460, AT3G09760, AT3G09540, AT3G05890, AT3G04010, AT3G03990, AT3G05630, AT3G05660, AT3G05640, AT3G10020, AT3G10530, AT3G10410, AT3G09440, AT3G08880, AT3G09350, AT3G02320, AT3G01310, AT3G02220, AT3G01560, AT3G11410, AT3G11420, AT3G07640, AT3G01150, AT3G05220, AT1G13270, AT3G16310, AT1G19050, AT1G20450, AT1G20630, AT1G20490, AT1G28060, AT1G27930, AT1G52280, AT1G63160, AT1G77450, AT1G60890, AT1G29370, AT1G29350, AT1G29395, AT1G29340, AT1G73700, AT1G73630, AT1G52890, AT1G68552, AT1G68550, AT1G74450, AT1G68470, AT1G68500, AT1G80480, AT1G80270, AT1G69250, AT1G69260, AT1G69870, AT1G69830, AT2G47260, AT1G53310, AT1G55900, AT1G62310, AT1G62320, AT1G62430, AT1G78420, AT1G43890, AT1G21460, AT1G02660, AT1G53440, AT1G53500, AT1G53590, AT1G07420, AT1G07360, AT1G75400, AT1G04945, AT1G32860, AT1G12845, AT1G20100, AT1G05805, AT1G26800, AT1G26665, AT1G35720, AT1G79660, AT1G18850, AT1G07870, AT1G01720, AT1G33230, AT1G01140, AT1G18320, AT3G10110, AT1G18480, AT1G18400, AT1G76180, AT1G80840, AT1G80750, AT1G22570, AT1G02820, AT1G02860, AT1G77850, AT1G78070, AT1G77810, AT1G53910, AT1G70782, AT1G70780, AT1G64100, AT1G64105, AT1G21660, AT1G31280, AT1G62710, AT1G16280, AT1G10770, AT1G10760, AT1G20840, AT1G14720, AT1G20920, AT1G59830, AT1G65700, AT1G79490, AT1G54410, AT2G27200, AT1G10590, AT1G10560, AT2G14170, AT2G03820, AT2G28720, AT2G22170, AT2G28600, AT2G42520, AT2G21660, AT1G04400, AT1G26850, AT2G21520, AT2G04350, AT2G36320, AT2G36390, AT2G36410, AT2G36580, AT3G52990, AT2G36220, AT2G21130, AT2G21180, AT2G22400, AT1G54690, AT1G22730, AT1G60190, AT1G54770, AT1G60140, AT1G09240, AT1G61870, AT1G61730, AT1G10090, AT1G30820, AT2G17670, AT2G17660, AT1G08970, AT1G08920, AT1G09780, AT1G22990, AT1G08580, AT1G23070, AT1G77120, AT1G67310, AT1G55500, AT1G05100, AT2G20370, AT2G16710, AT2G06530, AT2G46620, AT2G15970, AT2G25355, AT2G24500, AT2G35650, AT2G01640, AT2G25620, AT2G25625, AT2G19580, AT2G24240, AT2G24100, AT2G40780, AT2G02100, AT2G02070, AT2G38905, AT2G28840, AT2G27840, AT2G46680, AT2G38670, AT2G47970, AT2G47770, AT2G47840, AT2G47890, AT2G35290, AT2G24050, AT2G29630, AT2G19810, AT2G28930, AT2G44860, AT2G26150, AT2G25950, AT2G45820, AT2G34585, AT2G45900, AT2G45980, AT2G45730, AT2G18915, AT2G39130, AT2G38470, AT2G41190, AT2G41430, AT2G37520, AT2G23150, AT2G19450, AT2G23760, AT2G34750, AT2G34780, AT2G34930, AT2G45500, AT2G45660, AT2G30600, AT2G41900, AT2G42270

**2. List of chilling up-regulated DEGs (MA)**

AT3G08940, AT1G48690, AT1G80340, AT3G29970, AT5G17450, AT5G09990, AT1G33055, AT5G10040, AT3G44880, AT5G35670, AT1G72220, AT5G39890, AT4G33560, AT1G43800, AT5G57785, AT2G31790, AT3G06840, AT4G27450, AT1G28400, AT2G22330, AT1G31320, AT4G17870, AT2G37380, AT4G24780, AT2G47560, AT2G18050, AT5G65640, AT1G72200, AT4G33960, AT5G62720, AT4G02290, AT5G67430, AT2G16390, AT2G21560, AT4G23510, AT4G31910, AT4G22680, AT2G22420, AT4G31940, AT4G13050, AT3G26290, AT4G01450, AT1G49470, AT3G50880, AT4G18020, AT2G25590, AT1G61590, AT3G09480, AT3G07310, AT3G25510, AT1G31770, AT1G24100, AT1G31950, AT1G52720, AT2G22850, AT3G60490, AT5G34930, AT3G01980, AT1G31350, AT1G07350, AT3G09490, AT5G24290, AT5G37690, AT1G22700, AT5G40970, AT1G52080, AT5G12900, AT4G04830, AT3G51910, AT5G39850, AT4G38660, AT1G64200, AT4G36860, AT1G74090, AT5G06270, AT3G13650, AT5G53880, AT1G05710, AT5G53080, AT4G15390, AT4G31920, AT1G31170, AT3G14780, AT3G44540, AT1G35250, AT5G27400, AT3G11240, AT1G04040, AT1G34040, AT1G34060, AT1G78020, AT2G13690, AT2G38780, AT2G29660, AT3G51440, AT5G42630, AT4G32690, AT2G27810, AT5G46790, AT1G23020, AT5G57900, AT1G80800, AT2G03450, AT1G72630, AT1G70620, AT4G37300, AT3G23150, AT5G13910, AT2G45830, AT1G13080, AT4G01350, AT5G59920, AT1G62800, AT1G23030, AT4G37550, AT3G24450, AT1G26930, AT3G50640, AT1G65000, AT4G30400, AT3G43800, AT1G14900, AT1G03820, AT5G56610, AT5G10770, AT3G22550, AT1G06760, AT5G56220, AT2G38370, AT4G35420, AT5G18780, AT3G25290, AT5G19120, AT3G25190, AT1G72060, AT3G01360, AT4G25260, AT5G26660, AT3G07410, AT5G27950, AT5G46150, AT5G12110, AT1G24280, AT4G18610, AT2G20515, AT4G31530, AT1G27210, AT4G24180, AT1G54050, AT3G22968, AT3G22970, AT4G35410, AT4G37730, AT2G02770, AT3G11470, AT1G52550, AT1G59580, AT1G80870, AT4G13770, AT2G47440, AT2G23600, AT2G23590, AT3G14920, AT3G06440, AT4G14640, AT1G02850, AT1G30910, AT5G07820, AT1G07020, AT3G02875, AT1G58150, AT3G22800, AT4G37200, AT4G05590, AT5G65050, AT5G65060, AT5G22920, AT4G22790, AT5G13610, AT2G28410, AT4G31020, AT2G26740, AT2G26750, AT3G02690, AT4G22756, AT1G30500, AT1G80310, AT2G19310, AT3G58620, AT2G33480, AT1G14360, AT5G33280, AT2G34150, AT4G30760, AT5G20450, AT3G14570, AT5G49350, AT3G02180, AT4G17070, AT2G43600, AT5G48790, AT1G31800, AT1G73160, AT5G49360, AT4G38090, AT5G20550, AT4G18550, AT2G41820, AT5G38980, AT5G47100, AT5G57130, AT5G05860, AT1G65370, AT3G62370, AT4G11410, AT3G50630, AT5G09300, AT4G34600, AT4G32240, AT2G23700, AT3G46800, AT5G02520, AT2G31110, AT3G07900, AT5G67390, AT1G30200, AT5G59780, AT1G47330, AT1G08350, AT1G30510, AT5G27100, AT4G32730, AT3G26450, AT1G03030, AT2G24390, AT2G41760, AT5G55120, AT1G70790, AT5G43822, AT3G52370, AT3G27770, AT5G54080, AT3G03040, AT3G04940, AT5G65830, AT5G09995, AT3G63170, AT4G19400, AT4G29120, AT5G16560, AT1G14140, AT5G03570, AT2G34050, AT3G20260, AT1G17350, AT3G29780, AT5G51550, AT2G18290, AT1G12240, AT1G52340, AT5G40980, AT1G47780, AT1G47786, AT3G56740, AT3G51290, AT4G11130, AT4G23493, AT2G34430, AT2G34420, AT5G37360, AT4G13630, AT5G56020, AT4G17010, AT1G63260, AT1G29952, AT1G29951, AT1G29950, AT1G70210, AT4G17600, AT2G21830, AT4G08300, AT4G27900, AT5G38030, AT3G23570, AT3G57330, AT5G43490, AT5G48610, AT5G54050, AT3G11880, AT5G09740, AT4G00335, AT1G53165, AT5G11350, AT1G31180, AT5G14200, AT5G24100, AT3G28700, AT1G51680, AT5G46030, AT5G41000, AT3G18210, AT3G54900, AT5G10030, AT4G18630, AT3G06140, AT4G36660, AT1G28340, AT3G22220, AT1G22050, AT1G22170, AT3G44940, AT2G42390, AT5G64760, AT2G38170, AT5G11690, AT1G80860, AT5G43850, AT4G14010, AT2G32450, AT1G21920, AT1G10240, AT1G31330, AT1G67100, AT1G69780, AT5G12970, AT4G30900, AT5G52440, AT4G03500, AT3G22460, AT3G45850, AT1G12430, AT3G02240, AT5G18860, AT5G18890, AT1G30110, AT4G01897, AT4G23440, AT2G01220, AT2G24250, AT1G18880, AT3G51470, AT5G04910, AT5G37680, AT1G15640, AT5G22730, AT3G16870, AT3G18490, AT3G24315, AT4G21190, AT4G27260, AT4G11830, AT3G11745, AT1G70280, AT4G31450, AT1G62820, AT1G49350, AT3G56840, AT5G45920, AT4G32560, AT5G58787, AT4G33130, AT5G53020, AT5G65380, AT3G49590, AT1G76500, AT4G35470, AT2G01950, AT3G49050, AT1G62960, AT4G28750, AT1G20270, AT2G07360, AT5G50760, AT4G13950, AT5G19140, AT1G76150, AT1G02810, AT1G34470, AT5G55550, AT2G33180, AT2G32380, AT5G23405, AT2G22740, AT2G01890, AT1G79040, AT5G14220, AT5G53800, AT2G41770, AT2G35830, AT2G35810, AT4G16110, AT5G19670, AT5G09850, AT5G19570, AT5G01270, AT1G07170, AT3G16200, AT2G20610, AT2G37470, AT4G39040, AT1G75140, AT1G47740, AT2G23180, AT2G40890, AT2G45350, AT2G45330, AT5G23600, AT1G67830, AT1G58807, AT1G59124, AT1G44446, AT4G15550, AT4G16980, AT4G15545, AT4G14130, AT4G14100, AT4G15800, AT4G17790, AT4G15510, AT4G16410, AT4G17370, AT4G17540, AT4G16680, AT4G15920, AT4G14920, AT4G14540, AT1G56700, AT5G04190, AT1G66900, AT1G66920, AT1G66910, AT1G30260, AT1G32220, AT1G47128, AT1G26150, AT5G11060, AT5G19860, AT5G19590, AT5G13180, AT5G08330, AT5G10690, AT5G21070, AT5G21170, AT5G21105, AT5G08350, AT5G08370, AT5G19290, AT5G20220, AT5G20580, AT5G19310, AT5G20380, AT5G19950, AT5G20935, AT1G31820, AT4G36540, AT3G51830, AT1G57600, AT5G15830, AT5G34850, AT5G35170, AT5G27710, AT5G27350, AT5G26820, AT5G26210, AT5G26220, AT5G25490, AT5G25630, AT5G25140, AT5G24870, AT5G67280, AT5G67440, AT5G67580, AT5G67260, AT5G67070, AT5G66740, AT5G66590, AT5G65760, AT5G65780, AT5G64900, AT5G64370, AT5G64380, AT5G63530, AT5G63780, AT5G63180, AT5G63470, AT3G48590, AT5G62930, AT5G63060, AT5G61340, AT5G61420, AT5G61130, AT5G61250, AT5G60800, AT5G59130, AT5G58770, AT5G58260, AT5G58375, AT5G57780, AT5G57800, AT5G57930, AT5G56860, AT5G56260, AT5G56380, AT5G55620, AT5G55910, AT5G55380, AT5G55480, AT5G55130, AT5G55000, AT5G54980, AT5G54250, AT5G54630, AT5G53940, AT5G54130, AT5G53900, AT5G53200, AT5G53340, AT5G53170, AT5G52280, AT5G52190, AT5G51850, AT5G52120, AT5G51560, AT5G51010, AT5G50530, AT5G50640, AT5G50740, AT5G50160, AT5G49740, AT5G49730, AT5G49760, AT5G49100, AT5G48730, AT5G48490, AT5G47780, AT5G47970, AT5G47050, AT5G46470, AT5G46830, AT5G46780, AT5G46390, AT5G45410, AT5G45020, AT5G44680, AT5G44860, AT5G44190, AT5G44400, AT5G44410, AT5G42680, AT5G40890, AT5G40250, AT5G40330, AT5G40380, AT5G38140, AT5G36930, AT5G35790, AT5G35490, AT5G24430, AT5G24210, AT5G23920, AT5G23060, AT5G18850, AT5G18650, AT5G18630, AT5G18140, AT5G18390, AT5G16650, AT5G15350, AT5G15170, AT5G14430, AT5G14450, AT5G14460, AT5G13640, AT5G13730, AT5G13770, AT5G13400, AT5G11380, AT5G07240, AT5G06870, AT5G06265, AT5G06340, AT5G06280, AT5G05690, AT5G05670, AT5G05480, AT5G04490, AT5G04810, AT5G03430, AT5G03610, AT5G03350, AT5G03380, AT5G02940, AT5G02710, AT5G02760, AT5G02120, AT2G13910, AT5G02360, AT5G01920, AT5G01015, AT3G62410, AT3G62330, AT3G61780, AT3G62010, AT3G61430, AT3G61550, AT3G61010, AT3G60910, AT3G60290, AT3G60220, AT3G60070, AT3G59300, AT3G59060, AT3G59390, AT3G58120, AT3G58040, AT3G57190, AT3G56480, AT3G56360, AT3G56370, AT3G56140, AT3G55630, AT3G55420, AT3G54220, AT3G53470, AT3G53350, AT3G51950, AT3G51960, AT3G51420, AT3G50790, AT3G51150, AT3G50750, AT3G50070, AT3G50270, AT3G49160, AT3G48200, AT3G48460, AT3G47540, AT3G46540, AT3G46450, AT3G45210, AT3G44300, AT3G44310, AT4G39900, AT4G39770, AT4G39510, AT4G38950, AT4G38690, AT4G38860, AT4G38840, AT4G38430, AT4G37980, AT4G38350, AT4G38520, AT4G37560, AT4G37610, AT4G36220, AT4G37530, AT4G37520, AT4G37430, AT4G35880, AT4G36030, AT4G34630, AT4G34215, AT4G34030, AT4G34138, AT4G34090, AT4G33740, AT4G33580, AT4G33150, AT4G31600, AT4G30993, AT4G30340, AT4G30520, AT4G30450, AT4G29750, AT4G29220, AT4G29260, AT4G28780, AT4G28640, AT4G28270, AT4G28080, AT4G26850, AT4G26950, AT4G26760, AT4G26530, AT4G25780, AT4G25720, AT4G24760, AT4G24810, AT4G24700, AT4G24340, AT4G24350, AT4G24050, AT4G23690, AT4G23660, AT4G23260, AT4G22690, AT4G22710, AT4G22730, AT4G22753, AT4G22120, AT4G22340, AT4G21810, AT4G21510, AT4G20760, AT4G19985, AT4G19660, AT4G19820, AT4G19830, AT4G19860, AT4G19530, AT4G19160, AT4G19170, AT4G19420, AT4G19500, AT4G19510, AT4G19045, AT4G18970, AT4G18590, AT4G18570, AT4G18210, AT4G18220, AT4G13830, AT4G13510, AT4G13670, AT4G12980, AT4G13020, AT4G12970, AT4G12390, AT4G10470, AT4G10400, AT4G09920, AT4G10060, AT4G10120, AT4G09900, AT4G09670, AT4G08870, AT4G08900, AT4G09350, AT4G04850, AT4G04955, AT4G03200, AT4G03070, AT4G02850, AT4G02800, AT4G02430, AT4G02050, AT4G01080, AT4G01330, AT4G01070, AT4G00780, AT4G00050, AT4G00020, AT1G25375, AT1G18620, AT1G19670, AT4G10150, AT4G10160, AT4G10270, AT2G40610, AT2G40540, AT2G33530, AT1G67040, AT1G67080, AT1G20330, AT1G18020, AT1G17990, AT1G18010, AT1G18000, AT1G22370, AT1G22330, AT1G19150, AT1G55130, AT1G30130, AT3G12520, AT3G12150, AT1G69530, AT1G35910, AT3G11170, AT5G05580, AT3G11090, AT1G75280, AT1G75220, AT1G36280, AT4G18440, AT1G31550, AT1G66130, AT1G66150, AT1G33260, AT3G28270, AT3G52340, AT3G30300, AT3G13750, AT3G16857, AT3G22150, AT3G21390, AT3G24040, AT3G23880, AT3G14210, AT3G19710, AT3G15270, AT3G13222, AT3G23750, AT3G23805, AT3G15090, AT3G27820, AT3G15570, AT3G26570, AT3G30775, AT2G17970, AT2G04230, AT1G55360, AT3G10840, AT3G24770, AT3G25740, AT3G25805, AT3G12690, AT3G27420, AT3G23080, AT3G15900, AT3G18750, AT3G16910, AT3G25540, AT3G17040, AT3G25560, AT3G27540, AT3G21250, AT3G21200, AT3G16230, AT3G14620, AT3G26085, AT3G26100, AT3G23590, AT3G26890, AT3G15770, AT3G23310, AT3G26810, AT3G17470, AT3G22420, AT3G17330, AT3G02580, AT3G02450, AT3G06850, AT3G06750, AT3G07010, AT3G04290, AT3G04520, AT3G02870, AT3G02720, AT3G09920, AT3G09580, AT3G11945, AT3G10720, AT3G11750, AT3G04730, AT3G04000, AT3G03190, AT3G02050, AT3G10420, AT3G10160, AT3G10150, AT3G09980, AT3G10080, AT3G01370, AT3G01440, AT3G02020, AT5G14060, AT3G07540, AT3G09210, AT3G11700, AT3G02170, AT3G10230, AT3G10260, AT3G05430, AT3G01660, AT3G01690, AT3G09150, AT1G69160, AT1G06420, AT1G01620, AT1G01490, AT1G44000, AT1G19000, AT1G15790, AT1G12400, AT1G21270, AT1G20540, AT1G35340, AT1G28110, AT1G55310, AT1G63180, AT1G77670, AT1G64400, AT1G29660, AT1G29430, AT5G27780, AT1G66220, AT1G73602, AT1G73600, AT1G76730, AT1G53710, AT1G76530, AT1G68790, AT1G68840, AT1G68780, AT5G33210, AT1G35460, AT1G74670, AT1G68520, AT1G80520, AT1G70580, AT1G63800, AT1G69420, AT1G69370, AT1G70550, AT1G74100, AT1G72430, AT1G11080, AT2G41720, AT2G43820, AT1G32520, AT1G15020, AT1G49200, AT3G06690, AT1G06290, AT1G06830, AT1G53560, AT1G17360, AT1G01430, AT1G01320, AT1G07440, AT1G19835, AT1G19700, AT1G12775, AT1G20160, AT1G48480, AT1G44920, AT1G79720, AT1G53160, AT1G07640, AT1G21060, AT1G01800, AT1G01630, AT1G33240, AT1G49580, AT1G33110, AT1G27690, AT1G32750, AT1G76110, AT1G16080, AT1G30520, AT1G11410, AT1G11580, AT1G10650, AT1G15960, AT1G11440, AT1G80780, AT1G80690, AT1G22430, AT1G64680, AT1G64670, AT1G33811, AT1G35612, AT1G80050, AT1G80180, AT1G77930, AT1G77870, AT1G74720, AT1G68585, AT1G64260, AT1G72820, AT1G47420, AT1G50280, AT1G17140, AT1G31360, AT1G31300, AT1G15290, AT1G15260, AT1G14120, AT1G06510, AT1G14030, AT1G16410, AT1G16400, AT1G16390, AT1G28600, AT1G28610, AT1G16350, AT1G11700, AT1G11780, AT1G14700, AT1G64850, AT1G64780, AT1G64720, AT1G59960, AT1G23390, AT1G23400, AT1G23860, AT1G23360, AT2G18162, AT2G18160, AT2G17550, AT2G16090, AT1G05560, AT1G10610, AT2G22090, AT2G20500, AT2G04160, AT2G22250, AT2G22240, AT2G31670, AT2G03980, AT2G17033, AT2G01870, AT2G16280, AT2G16270, AT2G04850, AT2G25270, AT2G04690, AT1G04290, AT1G04350, AT2G04790, AT1G04520, AT1G26920, AT1G31230, AT1G60000, AT1G60010, AT2G46450, AT2G24550, AT2G36590, AT2G42760, AT2G42690, AT2G22530, AT2G28470, AT1G79220, AT1G78680, AT1G61900, AT1G03270, AT1G10360, AT1G27320, AT1G10380, AT1G09415, AT1G55750, AT3G61420, AT1G04680, AT1G04640, AT1G09750, AT1G70000, AT1G62250, AT1G03410, AT1G03400, AT2G17450, AT1G61190, AT1G61180, AT1G61300, AT1G61310, AT1G60690, AT1G76990, AT1G27120, AT1G27150, AT1G61500, AT1G03940, AT1G03495, AT1G62660, AT1G62540, AT1G51360, AT1G51400, AT1G23480, AT2G36630, ATMG00590, AT2G07718, AT2G42960, AT2G42950, AT2G20790, AT2G37170, AT2G37180, AT2G46580, AT2G15530, AT2G20110, AT2G27330, AT2G32090, AT2G03550, AT2G32100, AT2G48020, AT2G18560, AT2G18570, AT2G24220, AT2G24270, AT2G07680, AT2G37860, AT2G37790, AT2G45150, AT2G45170, AT2G27800, AT5G27300, AT2G29340, AT2G29320, AT2G29360, AT2G01420, AT2G46650, AT2G01450, AT2G41290, AT2G47800, AT2G47630, AT2G35155, AT2G35260, AT2G46250, AT2G35390, AT2G25830, AT2G19780, AT2G28950, AT2G29970, AT2G26910, AT2G44630, AT2G26080, AT2G45810, AT2G34620, AT2G45740, AT2G18910, AT2G39470, AT2G39280, AT2G34310, AT2G38310, AT2G41120, AT2G41040, AT2G38180, AT2G14750, AT2G32540, AT2G32530, AT2G32560, AT2G23390, AT2G37720, AT2G22990, AT2G22980, AT2G23780, AT2G30150, AT2G44230, AT2G39930, AT2G39890, AT2G40020, AT2G44210, AT2G44420, AT2G26250, AT2G26180, AT2G30490, AT2G30540, AT2G30520, AT2G30460, AT2G32180, AT2G32650, AT2G30790, AT2G39700, AT2G26710,

**3. List all expressed genes (MA)**

ATMG00640, ATMG00650, ATMG00660, ATMG00670, ATMG00690, ATMG00750, AT2G07686, ATMG00820, AT2G07783, ATMG00830, ATMG00850, AT2G07682, ATMG00890, AT2G07751, ATMG00990, ATMG01000, AT2G07674, ATMG01010, ATMG01020, ATMG01040, AT2G07664, ATMG00510, AT2G07712, ATMG00520, AT2G07663, ATMG00530, AT2G07776, ATMG00570, AT2G07716, ATMG00580, ATMG00600, AT2G07809, ATMG00630, ATCG01060, ATCG01070, ATCG01080, ATCG01090, ATCG01100, ATCG01110, ATCG01120, ATCG00065, ATCG01230, ATCG00905, ATMG00010, ATMG00070, ATMG00080, ATMG00090, ATMG00110, ATMG00130, ATMG00160, AT2G07723, ATMG00180, ATMG00260, AT2G07799, ATMG00270, AT2G07731, ATMG00280, AT2G07732, ATMG00310, AT2G07737, ATMG00400, ATMG00450, AT2G07708, ATMG00500, ATMG00490, ATCG01020, ATCG01040, ATCG01050, ATCG00570, ATCG00580, ATCG00590, ATCG00600, ATCG00630, ATCG00640, ATCG00650, ATCG00660, ATCG00670, ATCG00680, ATCG00690, ATCG00700, ATCG00710, ATCG00720, ATCG00730, ATCG00740, ATCG00750, ATCG00760, ATCG00770, ATCG00780, ATCG00790, ATCG00800, ATCG00810, ATCG00820, ATCG01310, ATCG00830, ATCG00840, ATCG01300, ATCG00860, ATCG01280, ATCG00870, ATCG01270, ATCG01250, ATCG00890, ATCG00900, ATCG01240, ATCG01000, ATCG01130, ATCG01010, ATCG00150, ATCG00160, ATCG00170, ATCG00180, ATCG00190, ATCG00210, ATCG00220, ATCG00270, ATCG00280, ATCG00300, ATCG00330, ATCG00340, ATCG00350, ATCG00360, ATCG00380, ATCG00420, ATCG00430, ATCG00440, ATCG00470, ATCG00480, ATCG00490, ATCG00500, ATCG00510, ATCG00520, ATCG00530, ATCG00540, ATCG00550, ATCG00560, ATCG00080, ATCG00120, ATCG00130, ATCG00140, AT2G26550, AT2G26570, AT2G26360, AT2G26630, AT2G26380, AT2G26390, AT2G26400, AT2G26410, AT2G26420, AT2G26560, AT2G26600, AT2G26530, AT2G26540, AT2G26430, AT2G26500, AT2G26590, AT2G26510, ATCG00020, ATCG00040, ATCG00050, ATCG00070, AT2G23320, AT2G26440, AT2G26450, AT2G26460, AT2G26470, AT2G26480, AT2G39720, AT2G39770, AT2G39730, AT2G39760, AT2G39795, AT2G39725, AT2G39750, AT2G39840, AT2G39780, AT2G23200, AT2G23170, AT2G23340, AT2G23330, AT2G23300, AT2G23270, AT2G23310, AT2G23290, AT2G23350, AT2G39830, AT2G45290, AT2G40900, AT2G40950, AT2G40815, AT2G40840, AT2G40860, AT2G40880, AT2G40935, AT2G40940, AT2G40830, AT2G40930, AT2G40890, AT2G41600, AT2G41610, AT2G41650, AT2G41690, AT2G41510, AT2G41520, AT2G41550, AT2G41540, AT2G41660, AT2G41630, AT2G41530, AT2G41620, AT2G41560, AT2G41680, AT2G41640, AT2G39810, AT2G47610, AT2G47420, AT2G47450, AT2G47580, AT2G47460, AT2G47600, AT2G45340, AT2G45330, AT5G23600, AT2G45320, AT2G45310, AT4G38280, AT2G45250, AT2G45210, AT2G45460, AT2G45430, AT2G45420, AT2G45400, AT2G45450, AT2G45240, AT2G45440, AT2G45200, AT2G45280, AT2G45220, AT2G47620, AT2G47590, AT2G47550, AT2G47490, AT5G12470, AT5G12480, AT2G33070, AT2G33240, AT2G33210, AT2G33180, AT2G33170, AT2G33120, AT2G33110, AT2G33150, AT2G33220, AT2G47560, AT2G47540, AT2G47520, AT2G47500, AT2G47470, AT2G47440, AT5G12390, AT5G12400, AT5G12410, AT5G12420, AT5G12430, AT5G12440, AT1G67760, AT1G67710, AT1G67650, AT1G67680, AT1G67660, AT1G67690, AT1G67780, AT1G67810, AT1G67820, AT1G67740, AT1G67750, AT1G67800, AT1G67700, AT1G67730, AT1G67850, AT1G67840, AT1G67720, AT5G12270, AT5G12280, AT5G12290, AT5G12310, AT5G12330, AT5G12340, AT5G12350, AT5G12370, AT1G44575, AT1G67830, AT4G16143, AT1G59218, AT1G58848, AT1G58807, AT1G59124, AT1G58808, AT1G59171, AT1G58889, AT1G59265, AT3G29970, AT1G08410, AT3G29810, AT4G25620, AT4G25610, AT4G25580, AT4G25560, AT4G25550, AT4G25540, AT4G25520, AT4G25570, AT1G44446, AT1G44414, AT1G44350, AT1G44318, AT4G17230, AT4G15415, AT4G16760, AT4G17490, AT4G17615, AT4G17500, AT4G15440, AT4G14680, AT4G14110, AT4G15090, AT4G14640, AT4G15340, AT4G14150, AT4G16360, AT4G14385, AT4G16563, AT4G17740, AT4G17070, AT4G14060, AT4G16570, AT4G14500, AT4G14960, AT4G16430, AT4G17250, AT4G14950, AT4G14360, AT4G16780, AT4G15550, AT4G17730, AT4G17270, AT4G16845, AT4G15560, AT4G14990, AT4G13980, AT4G14210, AT4G14030, AT4G14040, AT4G14880, AT4G14910, AT4G16420, AT4G15470, AT4G16490, AT4G16155, AT4G15093, AT4G16660, AT4G14340, AT4G16100, AT4G16370, AT4G16510, AT4G15010, AT4G17530, AT4G16350, AT4G17190, AT4G17695, AT4G17010, AT4G15630, AT4G17215, AT4G14690, AT4G16770, AT4G17486, AT4G15140, AT4G13950, AT4G14320, AT4G14600, AT4G15420, AT4G14800, AT4G14000, AT4G15610, AT4G16980, AT4G16146, AT4G16444, AT4G15545, AT4G16500, AT4G17260, AT4G14130, AT4G14100, AT4G16850, AT4G14465, AT4G14365, AT4G14930, AT4G14410, AT4G15940, AT4G14615, AT4G15800, AT4G16566, AT4G16442, AT4G16120, AT4G14420, AT4G16447, AT4G16720, AT4G15830, AT4G15475, AT4G16640, AT4G17090, AT4G14890, AT4G17770, AT4G16690, AT4G16830, AT4G17640, AT4G15490, AT4G17600, AT4G17390, AT4G13940, AT4G17560, AT4G15900, AT4G14430, AT4G16330, AT4G17790, AT4G17460, AT4G15120, AT4G15790, AT4G17720, AT4G14965, AT4G16265, AT4G15510, AT4G16840, AT4G15000, AT4G14716, AT4G14710, AT4G17620, AT4G17660, AT4G17690, AT4G17750, AT4G17760, AT4G17785, AT4G17800, AT4G17810, AT4G16790, AT4G14020, AT4G14010, AT4G17520, AT4G16410, AT4G17650, AT4G16520, AT4G16260, AT4G16450, AT4G17420, AT4G14870, AT4G14560, AT4G14900, AT4G17340, AT4G17040, AT4G17670, AT4G17590, AT4G17610, AT4G17150, AT4G17160, AT4G17170, AT4G17180, AT4G17200, AT4G17220, AT4G17240, AT4G17280, AT4G17300, AT4G17310, AT4G17330, AT4G17350, AT4G17370, AT4G17410, AT4G17430, AT4G17483, AT4G17510, AT4G17540, AT4G17550, AT4G17570, AT4G17060, AT4G17080, AT4G17140, AT4G16620, AT4G16650, AT4G16670, AT4G16680, AT4G16700, AT4G16710, AT4G16750, AT4G16800, AT4G16820, AT4G16860, AT4G16890, AT4G16920, AT4G16950, AT4G16940, AT4G16960, AT4G16970, AT4G16990, AT4G17000, AT4G17020, AT4G17030, AT4G16600, AT4G16610, AT4G15980, AT4G16010, AT4G16020, AT4G16060, AT4G16110, AT4G16130, AT4G16140, AT4G16170, AT4G16180, AT4G16190, AT4G16210, AT4G16240, AT4G16250, AT4G16270, AT4G16280, AT4G16340, AT4G16390, AT4G16400, AT4G16440, AT4G16460, AT4G16470, AT4G16480, AT4G15640, AT4G15730, AT4G15740, AT4G15770, AT4G15780, AT4G15810, AT4G15820, AT4G15840, AT4G15850, AT4G15880, AT4G15890, AT4G15910, AT4G15920, AT4G15930, AT4G15950, AT4G15955, AT4G15570, AT4G15590, AT4G15100, AT4G15110, AT4G15130, AT4G15160, AT4G15200, AT4G15230, AT4G15260, AT4G15270, AT4G15280, AT4G15290, AT4G15300, AT4G15310, AT4G15330, AT4G15350, AT4G15360, AT4G15370, AT4G15380, AT4G15390, AT4G15400, AT4G15410, AT4G15430, AT4G15460, AT4G15480, AT4G15500, AT4G15520, AT4G14580, AT4G14590, AT4G14605, AT4G14610, AT4G14630, AT4G14650, AT4G14660, AT4G14695, AT4G14720, AT4G14750, AT4G14760, AT4G14770, AT4G14780, AT4G14790, AT4G14810, AT4G14820, AT4G14840, AT4G14850, AT4G14920, AT4G14940, AT4G14970, AT4G14980, AT4G15020, AT4G15030, AT4G15060, AT4G14550, AT4G14570, AT4G14190, AT4G14200, AT4G14220, AT4G14230, AT4G14240, AT4G14270, AT4G14290, AT4G14300, AT4G14330, AT4G14350, AT4G14368, AT4G14380, AT4G14390, AT4G14440, AT4G14470, AT4G14490, AT4G14510, AT4G13990, AT4G14070, AT4G14096, AT4G14103, AT4G14160, AT1G56700, AT1G56600, AT1G56580, AT1G25360, AT1G25350, AT1G25290, AT1G25280, AT1G25250, AT1G25240, AT1G25230, AT1G24822, AT1G25097, AT1G24996, AT1G25260, AT1G25370, AT1G25275, AT1G25320, AT1G24764, AT1G24706, AT1G24735, AT1G24881, AT1G24800, AT1G25055, AT1G25211, AT1G25141, AT4G13960, AT1G56540, AT1G56720, AT1G28260, AT1G28240, AT1G28220, AT1G28190, AT1G28250, AT1G28280, AT1G28200, AT1G28330, AT1G28210, AT1G28230, AT1G56680, AT1G56670, AT1G56660, AT1G56610, AT1G56590, AT1G56560, AT5G08750, AT5G22030, AT5G22000, AT5G22220, AT5G22060, AT1G28320, AT1G28290, AT5G04240, AT5G04260, AT5G04170, AT5G04270, AT5G04200, AT5G04160, AT5G04250, AT5G04220, AT5G04290, AT5G04390, AT5G04310, AT5G04320, AT5G04330, AT5G04340, AT5G04360, AT5G04370, AT5G04280, AT5G08670, AT5G08690, AT5G08740, AT5G33390, AT5G04110, AT1G73460, AT1G73450, AT1G73430, AT1G73400, AT1G73390, AT1G73370, AT1G73360, AT1G73350, AT1G73340, AT1G73490, AT1G73470, AT1G73500, AT1G73440, AT1G73380, AT1G73480, AT1G73410, AT1G73330, AT1G44160, AT1G44130, AT1G44110, AT1G44100, AT1G44170, AT1G51070, AT1G51100, AT1G51140, AT1G51090, AT1G51060, AT1G25682, AT1G25988, AT1G35210, AT1G35190, AT1G35140, AT1G32240, AT1G66900, AT1G66890, AT1G27880, AT1G27850, AT1G19750, AT1G27840, AT1G33600, AT1G33580, AT1G33610, AT1G33590, AT1G30220, AT1G30240, AT1G30300, AT1G30290, AT1G30210, AT1G30270, AT1G30260, AT1G73540, AT1G73530, AT1G45688, AT1G45976, AT1G32190, AT1G32150, AT1G32130, AT1G32120, AT1G32090, AT1G32200, AT1G32210, AT1G32100, AT1G32220, AT1G32170, AT1G32160, AT1G32230, AT1G45230, AT1G45545, AT1G46264, AT1G46840, AT1G47128, AT1G46768, AT1G58470, AT1G58440, AT1G38065, AT1G38131, AT1G26090, AT1G26160, AT1G26100, AT1G26310, AT1G26270, AT1G26110, AT1G57870, AT1G57820, AT1G48840, AT1G48850, AT1G42220, AT1G42190, AT1G58450, AT1G58410, AT1G58390, AT1G58420, AT1G58380, AT1G58684, AT1G59359, AT1G58983, AT1G26170, AT1G26150, AT1G26130, AT5G13480, AT5G13520, AT5G13530, AT5G13510, AT5G13500, AT5G13490, AT5G13550, AT5G28240, AT5G28290, AT5G28300, AT5G01010, AT1G58060, AT1G58050, AT1G58070, AT1G57990, AT1G57980, AT1G58080, AT1G58030, AT1G26330, AT1G26300, AT1G26260, AT1G26240, AT1G26230, AT1G26190, AT5G09420, AT5G09462, AT5G09460, AT5G09463, AT5G09461, AT5G09500, AT5G09300, AT5G09440, AT5G09510, AT5G09390, AT5G09450, AT5G09480, AT5G09220, AT5G09270, AT5G09310, AT5G09230, AT5G09250, AT5G09400, AT5G11020, AT5G11030, AT5G11040, AT5G11060, AT5G11100, AT5G11110, AT5G11090, AT5G11070, AT5G09380, AT5G09410, AT5G19690, AT5G19600, AT5G19860, AT5G19620, AT5G19780, AT5G19770, AT5G28740, AT5G28790, AT5G28800, AT5G28820, AT5G28750, AT5G28770, AT5G24740, AT5G24750, AT5G24770, AT5G24780, AT5G24760, AT5G09240, AT5G09290, AT5G09320, AT5G09330, AT5G19840, AT5G19850, AT5G19750, AT5G19680, AT5G19760, AT5G19630, AT5G19820, AT5G19490, AT5G19500, AT5G19520, AT5G19530, AT5G19540, AT5G19510, AT5G19570, AT5G19550, AT5G28500, AT5G28530, AT5G28510, AT5G42020, AT5G28540, AT5G19590, AT5G19660, AT5G19670, AT5G19710, AT5G19720, AT5G19730, AT5G19790, AT5G19800, AT5G19830, AT5G20730, AT5G20680, AT5G13070, AT5G13080, AT5G13110, AT5G13150, AT5G13140, AT5G13100, AT5G13050, AT5G13090, AT5G13120, AT5G13160, AT5G13180, AT5G20610, AT5G20620, AT5G20640, AT5G20660, AT5G20690, AT5G20700, AT5G20760, AT5G20770, AT5G20810, AT5G20830, AT5G20650, AT5G20820, AT5G20790, AT5G20720, AT5G08415, AT5G08290, AT5G08410, AT5G08320, AT5G08335, AT5G08440, AT5G08330, AT5G10650, AT5G10660, AT5G10700, AT5G10720, AT5G10730, AT5G10695, AT5G10690, AT5G10710, AT5G21120, AT5G21070, AT5G21060, AT5G21170, AT5G21090, AT5G21105, AT5G21160, AT5G08280, AT5G08350, AT5G08300, AT5G08370, AT5G19420, AT5G19460, AT5G19470, AT5G19480, AT5G19370, AT5G19290, AT5G19440, AT5G19380, AT5G19450, AT5G19430, AT5G28850, AT5G28900, AT5G28840, AT5G08310, AT5G08340, AT5G08380, AT5G08390, AT5G08400, AT5G08430, AT5G08450, AT5G08420, AT5G19320, AT5G19330, AT5G19340, AT5G19350, AT5G19400, AT5G19410, AT5G20290, AT5G20220, AT5G20160, AT5G20150, AT5G20240, AT5G20180, AT5G20130, AT5G20410, AT5G20280, AT5G20430, AT5G20440, AT5G20450, AT5G20460, AT5G20470, AT5G20490, AT5G20480, AT5G20540, AT5G20580, AT5G20600, AT5G20570, AT5G20520, AT5G20590, AT5G20500, AT5G20550, AT5G19300, AT5G19310, AT5G20270, AT5G20400, AT5G20230, AT5G28640, AT5G28650, AT5G28700, AT5G28630, AT5G20120, AT5G20170, AT5G20200, AT5G20250, AT5G20300, AT5G20320, AT5G20350, AT5G20360, AT5G20380, AT5G20060, AT5G19875, AT5G20070, AT5G20990, AT5G20850, AT5G20960, AT5G20930, AT5G20885, AT5G19870, AT5G19900, AT5G19910, AT5G19920, AT5G19970, AT5G19980, AT5G20110, AT5G20050, AT5G19990, AT5G20000, AT5G19960, AT5G19890, AT5G19930, AT5G19950, AT5G20040, AT5G20010, AT5G20020, AT5G19940, AT5G20030, AT5G20090, AT5G20080, AT5G19855, AT5G21040, AT5G20900, AT4G36400, AT5G32470, AT5G32450, AT5G32440, AT3G61510, AT5G28360, AT5G28370, AT5G28460, AT3G61520, AT5G28400, AT5G28320, AT5G28430, AT3G60930, AT5G20840, AT5G20860, AT5G20870, AT5G20950, AT5G20980, AT5G21050, AT5G20910, AT5G21010, AT5G20890, AT5G20920, AT5G20880, AT4G37000, AT4G36410, AT4G37090, AT4G37010, AT4G36810, AT4G36530, AT4G37240, AT4G36750, AT4G37040, AT4G36610, AT4G37020, AT4G36970, AT4G36960, AT4G36870, AT4G36420, AT4G36730, AT4G36930, AT4G36480, AT4G36988, AT4G36990, AT4G37180, AT4G36380, AT4G36920, AT4G36800, AT4G36760, AT4G37210, AT4G37120, AT4G36900, AT4G36890, AT4G36590, AT4G36910, AT4G37200, AT4G36430, AT4G37160, AT4G36710, AT4G37080, AT4G36630, AT4G36550, AT4G37280, AT4G36830, AT4G36390, AT4G36670, AT4G36790, AT4G37130, AT4G37050, AT4G37250, AT4G36440, AT4G37170, AT4G36640, AT4G36680, AT4G36880, AT4G37220, AT4G37070, AT4G37060, AT4G37260, AT4G36650, AT4G36690, AT1G31840, AT1G31830, AT1G31820, AT1G31810, AT1G31790, AT1G31780, AT1G31814, AT1G31860, AT1G31817, AT1G31812, AT1G31800, AT4G37110, AT4G36500, AT4G37150, AT4G36620, AT4G36540, AT4G37270, AT4G37190, AT4G36740, AT4G36660, AT4G36940, AT4G36580, AT4G36860, AT4G36780, AT4G36980, AT1G31910, AT1G31870, AT1G31850, AT3G56880, AT3G56800, AT3G56820, AT3G56860, AT3G56710, AT3G56910, AT3G56690, AT3G56750, AT3G51770, AT3G51830, AT3G51850, AT3G51860, AT3G51870, AT3G51840, AT3G51890, AT3G51780, AT3G51800, AT3G51820, AT3G51790, AT3G51895, AT3G51880, AT1G31930, AT3G56850, AT3G56870, AT3G56900, AT3G56740, AT3G56680, AT1G16560, AT1G16640, AT1G16680, AT1G16690, AT1G16610, AT1G16540, AT1G16590, AT1G16670, AT3G43590, AT3G43610, AT3G43600, AT3G44820, AT3G44850, AT3G44880, AT3G44890, AT3G44860, AT3G44870, AT3G56720, AT3G56730, AT3G56760, AT3G56810, AT3G56830, AT3G56840, AT1G16650, AT1G16570, AT4G08010, AT1G39190, AT3G42721, AT1G40150, AT1G41690, AT1G41600, AT1G41115, AT1G41660, AT1G41630, AT1G40230, AT1G51850, AT1G51880, AT1G51890, AT1G51910, AT1G51920, AT1G51940, AT1G51860, AT1G51840, AT1G51830, AT1G51950, AT1G57620, AT1G57660, AT1G57860, AT1G77360, AT1G77370, AT1G77410, AT1G77400, AT1G77380, AT1G77330, AT1G77350, AT1G58150, AT1G58160, AT1G58170, AT1G58180, AT1G58190, AT1G58100, AT1G58110, AT1G57540, AT1G57560, AT1G57570, AT1G57590, AT1G57600, AT1G57630, AT1G57680, AT1G57700, AT5G17530, AT1G77310, AT5G17160, AT5G16990, AT5G17030, AT5G16870, AT5G16880, AT5G17060, AT5G16950, AT5G17020, AT5G17420, AT5G17430, AT5G17400, AT5G17410, AT5G17450, AT5G17470, AT5G17510, AT5G17520, AT5G17460, AT5G17440, AT5G17600, AT5G17650, AT5G17560, AT5G17610, AT5G17620, AT5G17570, AT5G17630, AT5G17640, AT5G16780, AT5G16800, AT5G16810, AT5G16820, AT5G16760, AT5G16840, AT5G16830, AT5G16710, AT5G16750, AT5G16860, AT5G16900, AT5G16910, AT5G16930, AT5G16940, AT5G16970, AT5G16980, AT5G17010, AT5G17040, AT5G17050, AT5G17070, AT5G17140, AT5G16720, AT5G16730, AT5G16770, AT5G15980, AT5G16060, AT5G16070, AT5G15960, AT5G15970, AT5G15930, AT5G16000, AT5G16040, AT5G15910, AT5G16010, AT5G16050, AT5G15950, AT5G15948, AT5G16100, AT5G16140, AT5G16180, AT5G16190, AT5G16200, AT5G16210, AT5G16220, AT5G16230, AT5G16260, AT5G16270, AT5G16280, AT5G16240, AT5G16130, AT5G16160, AT5G16250, AT5G16110, AT5G16120, AT5G16150, AT5G16715, AT5G15410, AT5G15490, AT5G15630, AT5G15680, AT5G15710, AT5G15740, AT5G15760, AT5G15770, AT5G15790, AT5G15810, AT5G15830, AT5G15850, AT5G15860, AT5G15750, AT5G15640, AT5G15730, AT5G15725, AT5G15870, AT5G15880, AT5G15890, AT5G15920, AT5G15940, AT5G15520, AT5G15460, AT5G15600, AT5G15400, AT5G15020, AT5G15090, AT5G14970, AT5G14910, AT5G15050, AT5G14920, AT5G15070, AT5G15420, AT5G15440, AT5G15450, AT5G15470, AT5G15480, AT5G15510, AT5G15540, AT5G15550, AT5G15570, AT5G15580, AT5G15610, AT5G15530, AT5G14940, AT5G14950, AT5G14960, AT1G31660, AT1G31650, AT1G31770, AT1G31760, AT5G14720, AT5G14730, AT5G14750, AT5G14770, AT5G14880, AT5G14790, AT5G14800, AT5G14780, AT5G14740, AT5G14760, AT5G14850, AT5G14900, AT5G14930, AT1G31710, AT5G35370, AT5G35390, AT5G35330, AT5G35320, AT5G35360, AT5G35410, AT5G36270, AT5G36280, AT5G36290, AT5G36220, AT5G36230, AT5G36250, AT1G48920, AT1G48910, AT1G48880, AT2G45300, AT1G48860, AT1G48900, AT1G50730, AT1G50740, AT1G29710, AT1G31730, AT5G34850, AT5G34895, AT5G34900, AT5G34883, AT5G34885, AT5G34908, AT5G59620, AT5G34920, AT5G35100, AT5G35080, AT5G35130, AT5G35160, AT5G35170, AT5G35190, AT5G35200, AT5G35220, AT5G35210, AT5G35250, AT5G35260, AT5G35270, AT5G35180, AT5G34830, AT5G30520, AT5G30450, AT5G30470, AT5G30510, AT5G30490, AT5G30420, AT5G33200, AT5G33280, AT5G33290, AT5G33300, AT5G34450, AT5G27990, AT5G28020, AT5G28050, AT5G28080, AT5G28130, AT5G28150, AT5G28160, AT5G28180, AT5G28170, AT5G28220, AT5G28960, AT5G28910, AT5G28940, AT5G29000, AT5G28010, AT5G28040, AT5G28060, AT5G27630, AT5G27650, AT5G27660, AT5G27680, AT5G27670, AT5G27710, AT5G27740, AT5G27800, AT5G27720, AT5G27840, AT5G27750, AT5G27760, AT5G27770, AT5G27820, AT5G27700, AT5G27730, AT5G27830, AT5G27920, AT5G27930, AT5G27850, AT5G27950, AT5G27970, AT5G27620, AT5G27150, AT5G27330, AT5G27340, AT5G27395, AT5G27400, AT5G27460, AT5G27490, AT5G27530, AT5G27540, AT5G27550, AT5G27420, AT5G27450, AT5G27520, AT5G27470, AT5G27350, AT5G27320, AT5G27360, AT5G27430, AT5G27380, AT5G27410, AT5G27600, AT5G27610, AT5G27280, AT5G27290, AT5G27210, AT5G27010, AT5G06350, AT5G27020, AT5G26770, AT5G26790, AT5G26940, AT5G26780, AT5G26830, AT5G27000, AT5G26980, AT5G27100, AT5G27110, AT5G27140, AT5G27120, AT5G27220, AT5G27240, AT5G27270, AT5G26910, AT5G26920, AT5G26960, AT5G26990, AT5G26260, AT5G26310, AT5G26330, AT5G26570, AT5G26360, AT5G26340, AT5G26600, AT5G27960, AT5G26650, AT5G26680, AT5G26720, AT5G26610, AT5G26731, AT5G26740, AT5G26660, AT5G26710, AT5G26760, AT5G26820, AT5G26850, AT5G26860, AT5G26880, AT5G26890, AT5G26200, AT5G26280, AT5G26210, AT5G25930, AT5G25890, AT5G25760, AT5G25940, AT5G25900, AT5G27640, AT5G25780, AT5G26010, AT5G26020, AT5G26030, AT5G26130, AT5G26110, AT5G26000, AT5G25980, AT5G26040, AT5G26180, AT5G26190, AT5G26220, AT5G26230, AT5G26240, AT5G26270, AT5G25490, AT5G25500, AT5G25520, AT5G25530, AT5G25540, AT5G25560, AT5G25580, AT5G25590, AT5G25630, AT5G25640, AT5G25480, AT5G25570, AT5G25475, AT5G25510, AT5G25610, AT5G25770, AT5G25800, AT5G25810, AT5G25820, AT5G25830, AT5G25880, AT5G25910, AT5G25280, AT5G25460, AT5G25090, AT5G25080, AT5G25110, AT5G25100, AT5G25060, AT5G25240, AT5G25260, AT5G25250, AT5G25270, AT5G25210, AT5G25220, AT5G25170, AT5G25190, AT5G25160, AT5G25350, AT5G25360, AT5G25370, AT5G25380, AT5G25400, AT5G25415, AT5G25440, AT5G25450, AT5G25050, AT5G25070, AT5G25120, AT5G25140, AT5G04880, AT5G04820, AT5G04850, AT5G04830, AT5G04870, AT5G04890, AT5G24670, AT5G24690, AT5G24710, AT5G24800, AT5G24830, AT5G24840, AT5G24850, AT5G24860, AT5G24870, AT5G24880, AT5G24900, AT5G24950, AT5G24960, AT5G24970, AT5G24890, AT5G24810, AT5G24910, AT5G25020, AT5G24990, AT5G67530, AT5G04840, AT5G04860, AT2G10560, AT5G67200, AT5G67290, AT5G67280, AT5G67300, AT5G67340, AT5G67350, AT5G67360, AT5G67400, AT5G67430, AT5G67450, AT5G67460, AT5G67470, AT5G67420, AT5G67390, AT5G67370, AT5G67440, AT5G67380, AT5G67330, AT5G67320, AT5G67570, AT5G67520, AT5G67490, AT5G67500, AT5G67560, AT5G67600, AT5G67510, AT5G67590, AT5G67580, AT5G67480, AT5G67630, AT5G66960, AT5G66970, AT5G67000, AT5G67050, AT5G66985, AT5G67030, AT5G67090, AT5G67100, AT5G67210, AT5G67230, AT5G67240, AT5G67250, AT5G67260, AT5G67110, AT5G67130, AT5G67070, AT5G67160, AT5G67270, AT5G67150, AT5G67220, AT5G66880, AT5G66850, AT5G66930, AT5G66540, AT5G66650, AT5G66560, AT5G66440, AT5G66670, AT5G66700, AT5G66710, AT5G66730, AT5G66750, AT5G66770, AT5G66680, AT5G66690, AT5G66760, AT5G66780, AT5G66810, AT5G66820, AT5G66890, AT5G66910, AT5G66900, AT5G66940, AT5G66950, AT5G66800, AT5G66920, AT5G66815, AT5G66640, AT5G66490, AT5G66570, AT5G66590, AT5G66410, AT5G66510, AT5G66420, AT5G66000, AT5G66055, AT5G66140, AT5G66130, AT5G66030, AT5G66290, AT5G66300, AT5G66310, AT5G66320, AT5G66330, AT5G66360, AT5G66390, AT5G66380, AT5G66350, AT5G66280, AT5G66400, AT5G66460, AT5G66470, AT5G66520, AT5G66530, AT5G66610, AT5G66620, AT5G66630, AT5G66240, AT5G66040, AT5G66160, AT5G65870, AT5G65830, AT5G65880, AT5G65950, AT5G65960, AT5G65910, AT5G65930, AT5G65970, AT5G65940, AT5G65900, AT5G65990, AT5G66010, AT5G66050, AT5G66060, AT5G66070, AT5G66080, AT5G66100, AT5G66180, AT5G66190, AT5G66200, AT5G66230, AT5G66170, AT5G66210, AT5G66120, AT5G66090, AT5G66250, AT5G65560, AT5G65570, AT5G65580, AT5G65600, AT5G65630, AT5G65670, AT5G65660, AT5G65650, AT5G65640, AT5G65620, AT5G65700, AT5G65710, AT5G65750, AT5G65760, AT5G65770, AT5G65780, AT5G65810, AT5G65685, AT5G65720, AT5G65790, AT5G65840, AT5G65850, AT5G65860, AT5G65520, AT5G65530, AT5G65540, AT5G65550, AT5G65260, AT5G65250, AT5G65280, AT5G65110, AT5G65300, AT5G65205, AT5G65320, AT5G65350, AT5G65370, AT5G65440, AT5G65450, AT5G65460, AT5G65470, AT5G65490, AT5G65430, AT5G65390, AT5G65420, AT5G65310, AT5G65360, AT5G65380, AT5G65480, AT5G65500, AT5G65240, AT5G65290, AT5G65210, AT5G65120, AT5G65220, AT5G65270, AT5G65180, AT5G64990, AT5G64890, AT5G64950, AT5G65000, AT5G64870, AT5G64830, AT5G65020, AT5G64880, AT5G65040, AT5G64900, AT5G64850, AT5G64905, AT5G64860, AT5G65050, AT5G65060, AT5G65010, AT5G64920, AT5G64960, AT5G64813, AT5G64840, AT5G65090, AT5G65100, AT5G65140, AT5G65160, AT5G65170, AT5G65230, AT5G64940, AT5G64970, AT5G64980, AT5G64580, AT5G64600, AT5G64610, AT5G64640, AT5G64660, AT5G64680, AT5G64690, AT5G64700, AT5G64710, AT5G64620, AT5G64650, AT5G64560, AT5G64670, AT5G64630, AT5G64740, AT5G64780, AT5G64730, AT5G64760, AT5G64816, AT5G64930, AT5G64500, AT5G64460, AT5G64440, AT5G64470, AT5G64530, AT5G64550, AT5G64552, AT5G64570, AT5G64140, AT5G64080, AT5G64220, AT5G64270, AT5G64300, AT5G64342, AT5G64340, AT5G64343, AT5G64341, AT5G64360, AT5G64370, AT5G64390, AT5G64420, AT5G64380, AT5G64310, AT5G64260, AT5G64350, AT5G64240, AT5G64250, AT5G64410, AT5G64400, AT5G64280, AT5G64230, AT5G64330, AT5G64290, AT5G64480, AT5G64490, AT5G64510, AT5G64180, AT5G64160, AT5G64100, AT5G63840, AT5G63910, AT5G63920, AT5G63880, AT5G63890, AT5G63850, AT5G63905, AT5G63870, AT5G63860, AT5G63930, AT5G63940, AT5G63950, AT5G63970, AT5G63980, AT5G64000, AT5G64010, AT5G64030, AT5G63990, AT5G64050, AT5G64040, AT5G64070, AT5G09350, AT5G64090, AT5G64170, AT5G64200, AT5G64110, AT5G64120, AT5G64130, AT5G64150, AT5G63510, AT5G63530, AT5G63440, AT5G63600, AT5G63610, AT5G63640, AT5G63660, AT5G63680, AT5G63700, AT5G63740, AT5G63760, AT5G63770, AT5G63780, AT5G63810, AT5G63830, AT5G63790, AT5G63650, AT5G63620, AT5G63590, AT5G63670, AT5G63800, AT5G63710, AT5G63580, AT5G63560, AT5G63450, AT5G63480, AT5G63140, AT5G63200, AT5G63220, AT5G63260, AT5G63290, AT5G63320, AT5G63110, AT5G63280, AT5G63150, AT5G63190, AT5G63135, AT5G63310, AT5G63120, AT5G63380, AT5G63400, AT5G63410, AT5G63370, AT5G63420, AT5G63470, AT3G48590, AT5G63490, AT5G63520, AT5G63570, AT5G63130, AT5G62860, AT5G62910, AT5G62930, AT5G62940, AT5G62950, AT5G62960, AT5G62790, AT5G62740, AT5G62890, AT5G62880, AT5G62920, AT5G62900, AT5G62760, AT5G62980, AT5G62990, AT5G63000, AT5G63010, AT5G63050, AT5G63060, AT5G63040, AT5G63030, AT5G63080, AT5G63100, AT5G62800, AT5G62810, AT5G62830, AT5G62550, AT5G62570, AT5G62580, AT5G62600, AT5G62620, AT5G62610, AT5G62520, AT5G62500, AT5G62540, AT5G62575, AT5G62480, AT5G62530, AT5G62460, AT5G62670, AT5G62680, AT5G62710, AT5G62690, AT5G62700, AT5G62720, AT5G62630, AT5G62640, AT5G62650, AT5G62730, AT5G62770, AT5G62290, AT5G62350, AT5G62430, AT5G62440, AT5G62470, AT5G62160, AT5G62170, AT5G62180, AT5G62200, AT5G62050, AT5G62100, AT5G62210, AT5G62070, AT5G62190, AT5G62090, AT5G62130, AT5G62000, AT5G62165, AT5G62220, AT5G62230, AT5G62260, AT5G62280, AT5G62310, AT5G62330, AT5G62340, AT5G62360, AT5G62390, AT5G62410, AT5G62150, AT5G61820, AT5G61830, AT5G61880, AT5G61900, AT5G61790, AT5G61760, AT5G61840, AT5G61770, AT5G61810, AT5G61865, AT5G61910, AT5G61920, AT5G61940, AT5G61980, AT5G61990, AT5G61970, AT5G61960, AT5G61930, AT5G62020, AT5G62030, AT5G62040, AT5G61510, AT5G61640, AT5G61740, AT5G61780, AT5G61800, AT5G61430, AT5G61310, AT5G61450, AT5G61340, AT5G61410, AT5G61440, AT5G61380, AT5G61470, AT5G61480, AT5G61460, AT5G61540, AT5G61560, AT5G61570, AT5G61580, AT5G61730, AT5G61690, AT5G61700, AT5G61590, AT5G61660, AT5G61500, AT5G61600, AT5G61670, AT5G61530, AT5G61360, AT5G61420, AT5G61370, AT5G60980, AT5G60920, AT5G61010, AT5G61020, AT5G61040, AT5G61060, AT5G61090, AT5G61130, AT5G61140, AT5G61150, AT5G61170, AT5G61190, AT5G61260, AT5G61240, AT5G61250, AT5G61210, AT5G61220, AT5G61230, AT5G61228, AT5G61030, AT5G61280, AT5G61300, AT5G61330, AT5G61350, AT5G60760, AT5G60750, AT5G60670, AT5G60680, AT5G60660, AT5G60620, AT5G60640, AT5G60690, AT5G60720, AT5G60790, AT5G60800, AT5G60820, AT5G60840, AT5G60860, AT5G60870, AT5G60890, AT5G60850, AT5G60900, AT5G60930, AT5G60950, AT5G61000, AT5G60960, AT5G60990, AT5G60940, AT5G60630, AT5G60710, AT5G60730, AT5G60740, AT5G60250, AT5G60270, AT5G60280, AT5G60350, AT5G60160, AT5G60200, AT5G60300, AT5G60360, AT5G60400, AT5G60410, AT5G60440, AT5G60460, AT5G60520, AT5G60550, AT5G60548, AT5G60570, AT5G60600, AT5G60490, AT5G60540, AT5G60590, AT5G60450, AT1G07940, AT1G07930, AT5G60390, AT1G07920, AT5G60530, AT5G60020, AT5G60030, AT5G59960, AT5G59870, AT5G59910, AT5G59950, AT5G59850, AT5G59820, AT5G59890, AT5G59845, AT5G59880, AT5G60040, AT5G60090, AT5G60120, AT5G60150, AT5G60100, AT5G60170, AT5G60190, AT5G60210, AT5G60220, AT5G60230, AT5G59930, AT5G59940, AT5G59980, AT5G59420, AT5G59520, AT5G59540, AT1G61210, AT5G59630, AT5G59650, AT5G59680, AT5G59700, AT5G59740, AT5G59760, AT5G59770, AT5G59800, AT5G59720, AT5G59970, AT5G59690, AT5G59730, AT5G59750, AT5G59710, AT5G59780, AT5G59830, AT5G59840, AT5G59900, AT5G59500, AT5G59560, AT5G59510, AT5G59460, AT5G59480, AT5G59450, AT5G59550, AT5G59250, AT5G59260, AT5G59270, AT5G59300, AT5G59340, AT5G59360, AT5G59350, AT5G59320, AT5G59290, AT5G59140, AT5G59150, AT5G59220, AT5G59380, AT5G59410, AT5G59430, AT5G59490, AT5G59530, AT5G59590, AT5G59600, AT5G59610, AT5G59400, AT5G59440, AT5G59370, AT5G59210, AT5G59240, AT5G58940, AT5G58960, AT5G58980, AT5G59010, AT5G59020, AT5G59030, AT5G58970, AT5G59000, AT5G58920, AT5G58950, AT5G59050, AT5G59070, AT5G59080, AT5G59090, AT5G59120, AT5G59160, AT5G59180, AT5G58870, AT5G58890, AT5G58900, AT5G58910, AT5G58930, AT5G58590, AT5G58630, AT5G58660, AT5G58690, AT5G58700, AT5G58740, AT5G58750, AT5G58760, AT5G58770, AT5G58784, AT5G58790, AT5G58800, AT5G58810, AT5G58600, AT5G58640, AT5G58730, AT5G58720, AT5G58710, AT5G58787, AT5G58650, AT5G58670, AT5G58620, AT5G58780, AT5G58570, AT5G58560, AT5G58170, AT5G58270, AT5G58190, AT5G58280, AT5G58290, AT5G58430, AT5G58330, AT5G58420, AT5G58375, AT5G58370, AT5G58350, AT5G58380, AT5G58230, AT5G58440, AT5G58450, AT5G58460, AT5G58470, AT5G58480, AT5G58500, AT5G58510, AT5G58520, AT5G58530, AT5G58540, AT5G58550, AT5G58575, AT5G58490, AT5G57910, AT5G57860, AT5G57840, AT5G57990, AT5G57870, AT5G58020, AT5G58040, AT5G58030, AT5G58090, AT5G58100, AT5G58110, AT5G58130, AT5G58150, AT5G58070, AT5G58060, AT5G58140, AT5G58200, AT5G58210, AT5G58300, AT5G58220, AT5G58410, AT5G58240, AT5G58160, AT5G58250, AT5G57900, AT1G25155, AT5G57890, AT1G25083, AT1G24909, AT1G24807, AT1G25220, AT5G57815, AT5G57630, AT5G57620, AT5G57580, AT5G57530, AT5G57670, AT5G57710, AT5G57720, AT5G57740, AT5G57770, AT5G57700, AT5G57785, AT5G57800, AT5G57830, AT5G57850, AT5G57880, AT5G57920, AT5G57930, AT5G57940, AT5G57960, AT5G57970, AT5G57980, AT5G58003, AT5G58010, AT5G57950, AT5G57360, AT5G57345, AT5G57290, AT5G57300, AT5G57350, AT5G57340, AT5G57390, AT5G57400, AT5G57420, AT5G57460, AT5G57440, AT5G57370, AT5G57410, AT5G57480, AT5G57510, AT5G57540, AT5G57590, AT5G57610, AT5G57650, AT5G57660, AT5G57500, AT5G57490, AT5G57655, AT5G57560, AT5G57280, AT5G57330, AT5G57060, AT5G57040, AT5G56980, AT5G57000, AT5G56940, AT5G57030, AT5G57110, AT5G57140, AT5G57160, AT5G57190, AT5G57120, AT5G57170, AT5G57100, AT5G57150, AT5G57090, AT5G57130, AT5G57220, AT5G57230, AT5G56870, AT5G56950, AT5G56970, AT5G57050, AT5G57070, AT5G57080, AT5G57020, AT5G56580, AT5G56590, AT5G56600, AT5G56540, AT5G56610, AT5G56620, AT5G56670, AT5G56720, AT5G56730, AT5G56740, AT5G56710, AT5G56750, AT5G56760, AT5G56630, AT5G56650, AT5G56660, AT5G56790, AT5G56900, AT5G56910, AT5G56350, AT5G56360, AT5G56320, AT5G56520, AT5G56130, AT5G56140, AT5G56160, AT5G56170, AT5G56180, AT5G56200, AT5G56150, AT5G56190, AT5G55990, AT5G56220, AT5G56230, AT5G56240, AT5G56250, AT5G56260, AT5G56270, AT5G56280, AT5G56290, AT5G56300, AT5G56340, AT5G56380, AT5G56460, AT5G56500, AT5G56450, AT5G55840, AT5G55850, AT5G55710, AT5G55630, AT5G55620, AT5G55700, AT5G55760, AT5G55640, AT5G55860, AT5G55910, AT5G55900, AT5G55920, AT5G55980, AT5G55970, AT5G55940, AT5G55960, AT5G56010, AT5G56000, AT5G56020, AT5G56030, AT5G56040, AT5G56080, AT5G56090, AT5G56100, AT5G55800, AT5G55820, AT5G55830, AT5G55500, AT5G55520, AT5G55530, AT5G55540, AT5G55560, AT5G55340, AT5G55550, AT5G55590, AT5G55600, AT5G55610, AT5G55650, AT5G55660, AT5G55670, AT5G55730, AT5G55740, AT5G55770, AT5G55780, AT5G55790, AT5G55380, AT5G55390, AT5G55400, AT5G55480, AT5G55490, AT5G55060, AT5G55070, AT5G55080, AT5G55090, AT5G55120, AT5G55170, AT5G55210, AT5G55220, AT5G55230, AT5G55260, AT5G55290, AT5G55300, AT5G55180, AT5G55200, AT5G55140, AT5G55160, AT5G55250, AT5G55280, AT5G55100, AT5G55190, AT5G55130, AT5G55310, AT5G55360, AT5G55370, AT5G54870, AT5G55000, AT5G55050, AT5G54590, AT5G54540, AT5G54690, AT5G54700, AT5G54720, AT5G54730, AT5G54760, AT5G54750, AT5G54770, AT5G54780, AT5G54790, AT5G54830, AT5G54840, AT5G54850, AT5G54860, AT5G54890, AT5G54910, AT5G54950, AT5G54960, AT5G54970, AT5G54980, AT5G55040, AT5G54800, AT5G54880, AT5G54940, AT5G54900, AT5G54930, AT5G54855, AT5G54670, AT5G54270, AT5G54260, AT5G54400, AT5G54390, AT5G54420, AT5G54440, AT5G54470, AT5G54480, AT5G54500, AT5G54510, AT5G54490, AT5G54430, AT5G54520, AT5G54570, AT5G54610, AT5G54680, AT5G54660, AT5G54580, AT5G54600, AT5G54640, AT5G54650, AT5G54630, AT5G54370, AT5G54380, AT5G54310, AT5G54290, AT5G54030, AT5G54040, AT5G54050, AT5G54060, AT5G53880, AT5G53940, AT5G54090, AT5G54130, AT5G54140, AT5G54080, AT5G54110, AT5G54200, AT5G54170, AT5G54160, AT5G54180, AT5G54230, AT5G54280, AT5G54300, AT5G53970, AT5G53990, AT5G54020, AT5G53660, AT5G53560, AT5G53650, AT5G53540, AT5G53530, AT5G53570, AT5G53620, AT5G53490, AT5G53820, AT5G53800, AT5G53830, AT5G53770, AT5G53760, AT5G53840, AT5G53850, AT5G53860, AT5G53870, AT5G53890, AT5G53900, AT5G53950, AT5G53580, AT5G53590, AT5G53588, AT5G53200, AT5G53120, AT5G53140, AT5G53130, AT5G53220, AT5G53250, AT5G53290, AT5G53320, AT5G53350, AT5G53360, AT5G53400, AT5G53330, AT5G53280, AT5G53340, AT5G53370, AT5G53310, AT5G53430, AT5G53440, AT5G53460, AT5G53480, AT5G53470, AT5G53450, AT5G53420, AT5G53500, AT5G53510, AT5G53550, AT5G52910, AT5G52950, AT5G52980, AT5G52900, AT5G52920, AT5G52975, AT5G52960, AT5G52870, AT5G52840, AT5G52880, AT5G53020, AT5G53050, AT5G53060, AT5G53070, AT5G53090, AT5G53100, AT5G53110, AT5G53080, AT5G53000, AT5G53150, AT5G53160, AT5G53170, AT5G53180, AT5G52560, AT5G52830, AT5G52850, AT5G52530, AT5G52540, AT5G52550, AT5G52552, AT5G52570, AT5G52580, AT5G52630, AT5G52670, AT5G52680, AT5G52690, AT5G52710, AT5G52740, AT5G52760, AT5G52820, AT5G52750, AT5G52660, AT5G52810, AT5G52650, AT5G52640, AT5G52390, AT5G58990, AT5G52370, AT5G52450, AT5G52420, AT5G52310, AT5G52440, AT5G52520, AT5G52180, AT5G52220, AT5G52230, AT5G52260, AT5G52280, AT5G52210, AT5G52250, AT5G52190, AT5G52240, AT5G52200, AT5G52300, AT5G52320, AT5G52330, AT5G52350, AT5G52380, AT5G52400, AT5G52410, AT5G52430, AT5G52460, AT5G52470, AT5G52510, AT5G52040, AT5G52170, AT5G51810, AT5G51710, AT5G51880, AT5G51840, AT5G51700, AT5G51820, AT5G51830, AT5G51890, AT5G51910, AT5G51940, AT5G51960, AT5G51980, AT5G51990, AT5G52030, AT5G52060, AT5G52070, AT5G52120, AT5G51970, AT5G52010, AT5G52020, AT5G52110, AT5G51410, AT5G51460, AT5G51480, AT5G51500, AT5G51545, AT5G51570, AT5G51580, AT5G51590, AT5G51600, AT5G51610, AT5G51620, AT5G51630, AT5G51660, AT5G51550, AT5G51560, AT5G51510, AT5G51640, AT5G51670, AT5G51680, AT5G51690, AT5G51740, AT5G51750, AT5G51760, AT5G51770, AT5G51390, AT5G51400, AT5G51440, AT5G51210, AT5G51230, AT5G51260, AT5G51270, AT5G51280, AT5G51310, AT5G51170, AT5G51140, AT5G51120, AT5G51190, AT5G51110, AT5G51290, AT5G51180, AT5G51300, AT5G51340, AT5G51350, AT5G51380, AT5G51430, AT5G51020, AT5G50915, AT2G47510, AT5G50950, AT5G50960, AT5G51130, AT5G51160, AT5G51200, AT5G50720, AT5G50800, AT5G50820, AT5G50840, AT5G50860, AT5G50810, AT5G50850, AT5G50890, AT5G50900, AT5G50870, AT5G50920, AT5G50930, AT5G50990, AT5G51050, AT5G51060, AT5G51070, AT5G51080, AT5G50940, AT5G51010, AT5G51040, AT5G51100, AT5G50780, AT5G50380, AT5G50390, AT5G50400, AT5G50410, AT5G50440, AT5G50450, AT5G50360, AT5G50370, AT5G50420, AT5G50320, AT5G50315, AT5G50375, AT5G50460, AT5G50530, AT5G50640, AT5G50550, AT5G50650, AT5G50660, AT5G50560, AT5G50700, AT5G50600, AT5G50630, AT5G50520, AT5G50680, AT5G50580, AT5G50670, AT5G50570, AT5G50610, AT5G50710, AT5G50740, AT5G50760, AT5G50000, AT5G49945, AT5G49950, AT5G49940, AT5G50020, AT5G50120, AT5G50140, AT5G50100, AT5G50110, AT5G50130, AT5G50160, AT5G50180, AT5G50230, AT5G50260, AT5G50280, AT5G50300, AT5G50310, AT5G50210, AT5G50200, AT5G50250, AT5G50170, AT5G50330, AT5G50340, AT5G50350, AT5G49980, AT5G49990, AT5G50012, AT5G50011, AT5G50010, AT5G49970, AT5G49680, AT5G49700, AT5G49710, AT5G49740, AT5G49730, AT5G49750, AT5G49760, AT5G49770, AT5G49780, AT5G49790, AT5G49800, AT5G49720, AT5G49830, AT5G49840, AT5G49810, AT5G49870, AT5G49820, AT5G49880, AT5G49890, AT5G49900, AT5G49910, AT5G49930, AT5G49960, AT5G49640, AT5G49610, AT5G49550, AT5G49540, AT5G49510, AT5G49660, AT5G49650, AT5G49280, AT5G49210, AT5G49230, AT5G49160, AT5G49390, AT5G49400, AT5G49430, AT5G49470, AT5G49410, AT5G49450, AT5G49448, AT5G49480, AT5G49460, AT5G49500, AT5G49520, AT5G49530, AT5G49555, AT5G49560, AT5G49570, AT5G49580, AT5G49620, AT5G49630, AT5G49320, AT5G49350, AT5G49360, AT5G48790, AT5G48880, AT5G48940, AT5G48950, AT5G48960, AT5G48970, AT5G48990, AT5G49000, AT5G49010, AT5G49030, AT5G49060, AT5G49070, AT5G48930, AT5G48910, AT5G49110, AT5G49120, AT5G49130, AT5G49100, AT5G49220, AT5G49270, AT5G48760, AT5G48460, AT5G48570, AT5G48600, AT5G48640, AT5G48650, AT5G48690, AT5G48590, AT5G48580, AT5G48655, AT5G48630, AT5G48710, AT5G48720, AT5G48730, AT5G48740, AT5G48780, AT5G48800, AT5G48820, AT5G48850, AT5G48870, AT5G48830, AT5G48840, AT5G48900, AT5G48810, AT5G48490, AT5G48485, AT5G48500, AT5G48540, AT5G48300, AT5G48220, AT5G48150, AT5G48230, AT5G48310, AT4G15070, AT5G48320, AT5G48330, AT5G48340, AT5G48360, AT5G48370, AT5G48380, AT5G48400, AT5G48410, AT5G48420, AT5G48430, AT5G48450, AT5G48520, AT5G48530, AT5G48560, AT5G48470, AT5G48480, AT5G48270, AT5G48180, AT5G48290, AT5G48175, AT5G47770, AT5G47910, AT5G47750, AT5G47780, AT5G47810, AT5G47950, AT5G47970, AT5G47980, AT5G47960, AT5G47990, AT5G48000, AT5G48010, AT5G48060, AT5G48070, AT5G48090, AT5G48100, AT5G48110, AT5G48120, AT5G48020, AT5G48030, AT5G48170, AT5G48250, AT5G47890, AT5G47930, AT5G47840, AT5G47880, AT5G47530, AT5G47540, AT5G47600, AT5G47630, AT5G47560, AT5G47680, AT5G47620, AT5G47570, AT5G47635, AT5G47455, AT5G47550, AT5G47640, AT5G47650, AT5G47580, AT5G47710, AT5G47700, AT5G47730, AT5G47740, AT5G47790, AT5G47800, AT5G47820, AT5G47830, AT5G47920, AT5G47940, AT5G47720, AT5G47760, AT5G47870, AT5G47340, AT5G47360, AT5G47400, AT5G47410, AT5G47420, AT5G47430, AT5G47450, AT5G47350, AT5G47200, AT5G47240, AT5G47220, AT5G47390, AT5G47180, AT5G47210, AT5G47190, AT5G47230, AT5G47320, AT5G47370, AT5G47435, AT5G47460, AT5G47470, AT5G47480, AT5G47490, AT5G47500, AT5G47510, AT5G47520, AT5G47310, AT5G46860, AT5G46910, AT5G46920, AT5G47010, AT5G47020, AT5G47040, AT5G47050, AT5G47060, AT5G47070, AT5G47000, AT5G47030, AT5G47080, AT5G47100, AT5G47110, AT5G47130, AT5G47150, AT5G47170, AT5G47120, AT5G47090, AT5G47250, AT5G46800, AT5G46740, AT5G46850, AT5G46900, AT5G46890, AT5G46470, AT5G46500, AT5G46510, AT5G46520, AT5G46540, AT5G46550, AT5G46560, AT5G46570, AT5G46580, AT5G46590, AT5G46620, AT5G46640, AT5G46630, AT5G46700, AT5G46730, AT5G46750, AT5G46760, AT5G46790, AT5G46780, AT5G46840, AT5G46710, AT5G46680, AT5G46460, AT5G46140, AT5G46160, AT5G46180, AT5G46020, AT5G46210, AT5G46190, AT5G46030, AT5G46150, AT5G46110, AT5G46230, AT5G46270, AT5G46280, AT5G46320, AT5G46350, AT5G46360, AT5G46390, AT5G46400, AT5G46410, AT5G46290, AT5G46295, AT5G46250, AT5G46420, AT5G46340, AT5G45800, AT5G45670, AT5G45760, AT5G45750, AT5G45840, AT5G45890, AT5G45920, AT5G45940, AT5G45910, AT5G45900, AT5G45970, AT5G46040, AT5G46050, AT5G46070, AT5G46080, AT5G46100, AT5G45710, AT5G45780, AT5G45790, AT5G45400, AT5G45460, AT5G45470, AT5G45480, AT5G45490, AT5G45440, AT5G45500, AT5G45510, AT5G45530, AT5G45540, AT5G45560, AT5G45390, AT5G45550, AT5G45410, AT5G45430, AT5G45428, AT5G45420, AT5G45600, AT5G45610, AT5G45620, AT5G45590, AT5G45630, AT5G45650, AT5G45680, AT5G45700, AT5G45340, AT5G45370, AT5G45360, AT5G45350, AT5G45280, AT5G45380, AT5G45000, AT5G45010, AT5G45050, AT5G45060, AT5G44930, AT5G45020, AT5G45070, AT5G45080, AT5G45090, AT5G45110, AT5G45120, AT5G45130, AT5G45140, AT5G45160, AT5G45170, AT5G45190, AT5G45200, AT5G45210, AT5G45220, AT5G45250, AT5G45260, AT5G45300, AT5G45320, AT5G44980, AT5G44990, AT5G44520, AT5G44500, AT5G44630, AT5G44660, AT5G44650, AT5G44610, AT5G44580, AT5G44670, AT5G44700, AT5G44710, AT5G44730, AT5G44750, AT5G44760, AT5G44780, AT5G44800, AT5G44820, AT5G44850, AT5G44860, AT5G44720, AT5G44785, AT5G44790, AT5G44880, AT5G44900, AT5G44910, AT5G44920, AT5G44160, AT5G44190, AT5G44200, AT5G44130, AT5G44280, AT5G44320, AT5G44350, AT5G44370, AT5G44380, AT5G44400, AT5G44410, AT5G44340, AT5G44290, AT5G44390, AT5G44420, AT5G44450, AT5G44460, AT5G44480, AT5G44510, AT5G44530, AT5G44560, AT5G44550, AT5G44270, AT5G44110, AT5G44250, AT5G44260, AT5G43920, AT5G43960, AT5G43980, AT5G44030, AT5G44050, AT5G44060, AT5G44020, AT5G44080, AT5G44000, AT5G43970, AT5G43940, AT5G44070, AT5G43930, AT5G43990, AT5G44090, AT5G44150, AT5G44170, AT5G44180, AT5G44210, AT5G44240, AT5G43745, AT5G43860, AT5G43710, AT5G43880, AT5G43890, AT5G43900, AT5G43910, AT5G43520, AT5G43560, AT5G43580, AT5G43590, AT5G43600, AT5G43610, AT5G43640, AT5G43670, AT5G43680, AT5G43690, AT5G43700, AT5G43730, AT5G43780, AT5G43790, AT5G43810, AT5G43822, AT5G43870, AT5G43720, AT5G43750, AT5G43830, AT5G43850, AT5G43760, AT5G43740, AT5G43450, AT5G43380, AT5G43500, AT5G43440, AT5G43080, AT5G43100, AT5G43130, AT5G43150, AT5G43180, AT5G43140, AT5G43070, AT5G43170, AT5G43190, AT5G43230, AT5G43270, AT5G43280, AT5G43310, AT5G43330, AT5G43260, AT5G43320, AT5G43360, AT5G43350, AT5G43370, AT5G43400, AT5G43390, AT5G43490, AT5G48610, AT5G43430, AT5G43460, AT5G42660, AT5G42790, AT5G42765, AT5G42740, AT5G42820, AT5G42810, AT5G42840, AT5G42860, AT5G42870, AT5G42880, AT5G42940, AT5G42950, AT5G42900, AT5G42970, AT5G42980, AT5G42850, AT5G42890, AT5G43010, AT5G42920, AT5G42960, AT5G42990, AT5G43020, AT5G43030, AT5G43040, AT5G43060, AT5G42830, AT5G42780, AT5G42750, AT5G42760, AT5G42470, AT5G42480, AT5G42500, AT5G42510, AT5G42560, AT5G42380, AT5G42350, AT5G42360, AT5G42520, AT5G42540, AT5G42370, AT5G42580, AT5G42590, AT5G42570, AT5G42630, AT5G42650, AT5G42620, AT5G42670, AT5G42690, AT5G42700, AT5G42720, AT5G42800, AT5G42240, AT5G42300, AT5G42390, AT5G42400, AT5G42420, AT5G42440, AT5G42450, AT5G42130, AT5G42140, AT5G42170, AT5G42180, AT5G42190, AT5G42090, AT5G42070, AT5G42030, AT5G42080, AT5G42150, AT5G42200, AT5G42100, AT5G41950, AT5G42050, AT5G41970, AT5G41990, AT5G41992, AT5G42220, AT5G42250, AT5G42270, AT5G42280, AT5G42310, AT5G42320, AT5G42000, AT5G42010, AT5G42060, AT5G41610, AT5G41620, AT5G41650, AT5G41750, AT5G41740, AT5G41700, AT5G41670, AT5G41600, AT5G41685, AT5G41770, AT5G41790, AT5G41850, AT5G41880, AT5G41940, AT5G41760, AT5G41800, AT5G41810, AT5G41960, AT5G41580, AT5G41050, AT5G41040, AT5G41060, AT5G41210, AT5G41150, AT5G41260, AT5G41280, AT5G41330, AT5G41340, AT5G41460, AT5G41480, AT5G41400, AT5G41360, AT5G41370, AT5G41350, AT5G41410, AT5G41520, AT5G41550, AT5G41560, AT5G41180, AT5G41190, AT5G41220, AT5G41240, AT5G40870, AT5G40880, AT5G49200, AT5G40910, AT5G40920, AT5G40930, AT5G40810, AT5G40850, AT5G40840, AT5G40890, AT5G40820, AT5G40960, AT5G40970, AT5G40950, AT5G40980, AT5G40990, AT5G41000, AT5G41010, AT5G41070, AT5G41080, AT5G41090, AT5G41100, AT5G41140, AT5G41170, AT5G40660, AT5G40650, AT5G40770, AT5G40740, AT5G40780, AT5G40830, AT5G40440, AT5G40480, AT5G40500, AT5G40520, AT5G40490, AT5G40510, AT5G40470, AT5G40530, AT5G40540, AT5G40550, AT5G40590, AT5G40600, AT5G40610, AT5G40630, AT5G40640, AT5G40680, AT5G40710, AT5G40760, AT5G40670, AT5G40580, AT5G40730, AT5G40645, AT5G40690, AT5G40450, AT5G40460, AT5G40370, AT5G40050, AT5G39860, AT5G39890, AT5G39950, AT5G40060, AT5G40090, AT5G40100, AT5G40130, AT5G40150, AT5G40170, AT5G40190, AT5G40200, AT5G40230, AT5G40250, AT5G40290, AT5G40300, AT5G40260, AT5G40270, AT5G40160, AT5G40280, AT5G40210, AT5G40320, AT5G40330, AT5G40340, AT5G40390, AT5G39620, AT5G39700, AT5G39660, AT5G39570, AT5G39670, AT5G39785, AT5G39830, AT5G39760, AT5G40080, AT5G39800, AT5G39790, AT5G39840, AT5G39850, AT5G39900, AT5G39910, AT5G39940, AT5G39960, AT5G39970, AT5G39990, AT5G40010, AT5G40020, AT5G40030, AT5G39730, AT5G39590, AT5G39600, AT5G39380, AT5G39500, AT5G39510, AT5G39520, AT5G39410, AT1G66040, AT1G66050, AT5G39550, AT5G39560, AT5G39580, AT5G39630, AT5G39690, AT3G56530, AT5G39710, AT5G39740, AT5G39610, AT5G39320, AT5G39360, AT5G39340, AT5G39160, AT5G39130, AT5G39190, AT5G38890, AT5G38910, AT5G38940, AT5G38930, AT5G38960, AT5G38990, AT5G39000, AT5G38900, AT5G38980, AT5G38895, AT5G39020, AT5G39040, AT5G39070, AT5G39090, AT5G39110, AT5G39150, AT5G39120, AT5G39180, AT5G39050, AT5G39100, AT5G39200, AT5G39140, AT5G39170, AT5G39220, AT5G39250, AT5G38850, AT5G38880, AT5G38380, AT5G38470, AT5G38460, AT5G38480, AT5G38530, AT5G38550, AT5G38540, AT5G38610, AT5G38660, AT5G38690, AT5G38700, AT5G38630, AT5G38650, AT5G38560, AT5G38710, AT5G38720, AT5G38730, AT5G38770, AT5G38790, AT5G38820, AT5G38830, AT5G38840, AT5G38120, AT5G38140, AT5G38110, AT5G38030, AT5G38150, AT5G38170, AT5G38210, AT5G38220, AT5G38250, AT5G38240, AT5G38290, AT5G38300, AT5G38280, AT5G38310, AT5G38340, AT5G38350, AT5G38360, AT5G38440, AT5G38450, AT5G38020, AT5G38070, AT5G37620, AT5G37630, AT5G37640, AT5G37660, AT5G37670, AT5G37690, AT5G37710, AT5G37730, AT5G37680, AT5G37740, AT5G37600, AT5G37780, AT5G37770, AT5G37810, AT5G37820, AT5G37830, AT5G37790, AT5G37850, AT5G37890, AT5G37910, AT5G37930, AT5G38010, AT5G37950, AT5G37970, AT5G37990, AT5G37940, AT5G38000, AT5G37980, AT5G37210, AT5G37230, AT5G37260, AT5G37280, AT5G37310, AT5G37360, AT5G37370, AT5G37290, AT5G37380, AT5G37750, AT5G37440, AT5G37450, AT5G37490, AT5G37500, AT5G37530, AT5G37550, AT5G37570, AT5G37480, AT5G37540, AT5G37510, AT5G37590, AT5G52090, AT5G37150, AT5G37160, AT5G37170, AT5G37180, AT5G36870, AT5G36890, AT5G36880, AT5G36930, AT5G36940, AT5G36950, AT5G37010, AT5G37040, AT5G37055, AT5G37020, AT5G37070, AT5G37110, AT5G37130, AT5G36790, AT5G36700, AT5G36710, AT5G36800, AT5G36770, AT5G36680, AT5G35830, AT5G35840, AT5G35880, AT5G35910, AT5G35930, AT5G35940, AT5G35960, AT5G35970, AT5G35980, AT3G61730, AT5G36000, AT5G36090, AT5G36130, AT5G36110, AT5G36140, AT5G36150, AT5G36160, AT5G36210, AT5G36170, AT5G35750, AT5G35790, AT5G35520, AT5G35540, AT5G35530, AT5G35460, AT5G35570, AT5G35560, AT5G35580, AT5G35670, AT5G35630, AT5G35680, AT5G35620, AT5G35770, AT5G35730, AT5G35740, AT5G35735, AT5G35700, AT5G24655, AT5G35430, AT5G35450, AT5G24390, AT5G24410, AT5G24430, AT5G24400, AT5G24360, AT5G24450, AT5G24460, AT5G24480, AT5G24520, AT5G24500, AT5G24470, AT5G24490, AT5G24540, AT5G24550, AT5G24560, AT5G24590, AT5G24600, AT5G24620, AT5G24570, AT5G24650, AT5G24660, AT5G24610, AT5G24530, AT5G24580, AT5G24313, AT5G24316, AT5G24350, AT5G24380, AT5G24000, AT5G24010, AT5G24020, AT5G24030, AT5G24070, AT5G24090, AT5G24100, AT5G24120, AT5G24110, AT5G24080, AT5G24140, AT5G24150, AT5G24160, AT5G24170, AT5G24165, AT5G24230, AT5G24260, AT5G24270, AT5G24280, AT5G24300, AT5G24310, AT5G24320, AT5G24330, AT5G24340, AT5G24290, AT5G23810, AT5G23720, AT5G23680, AT5G23530, AT5G23740, AT5G23540, AT5G23750, AT5G23730, AT5G23670, AT5G23580, AT5G23770, AT5G23800, AT5G23850, AT5G23870, AT5G23890, AT5G23910, AT5G23920, AT5G23760, AT5G23830, AT5G23940, AT5G23840, AT5G23900, AT5G23880, AT5G23820, AT5G23860, AT5G23690, AT5G23710, AT5G23350, AT5G23360, AT5G23380, AT5G23390, AT5G23310, AT5G23330, AT5G23250, AT5G23290, AT5G23300, AT5G23340, AT5G23400, AT5G23430, AT5G23440, AT5G23490, AT5G23510, AT5G23420, AT5G23480, AT5G23460, AT5G23405, AT5G23450, AT5G23520, AT5G23550, AT5G23570, AT5G23610, AT5G23630, AT5G23210, AT5G23220, AT5G23230, AT5G23240, AT5G23260, AT5G23320, AT5G22960, AT5G22970, AT5G22980, AT5G22840, AT5G22860, AT5G22875, AT5G22920, AT5G22950, AT5G22830, AT5G22820, AT5G23010, AT5G23020, AT5G23050, AT5G23080, AT5G23110, AT5G23130, AT5G23140, AT5G23070, AT5G23120, AT5G23060, AT5G23150, AT5G23090, AT5G23170, AT5G23180, AT5G23190, AT5G22890, AT5G22940, AT5G22320, AT5G22310, AT5G37340, AT5G22480, AT5G22540, AT5G22570, AT5G22555, AT5G22580, AT5G22500, AT5G22530, AT5G22550, AT5G22510, AT5G22620, AT5G22640, AT5G22650, AT5G22690, AT5G22700, AT5G22710, AT5G22730, AT5G22760, AT5G22770, AT5G22780, AT5G22630, AT5G22740, AT5G22790, AT5G22850, AT5G22870, AT5G22880, AT5G19240, AT5G19250, AT5G19260, AT5G19140, AT5G19120, AT5G19280, AT5G19150, AT5G19180, AT5G19220, AT5G22250, AT5G22340, AT5G22360, AT5G22370, AT5G22400, AT5G22410, AT5G22420, AT5G22450, AT5G22330, AT5G22270, AT5G22300, AT5G22280, AT5G22290, AT5G22440, AT5G19200, AT5G19210, AT5G18930, AT5G18950, AT5G18960, AT5G18920, AT5G18840, AT5G18940, AT5G18900, AT5G18970, AT5G18800, AT5G18830, AT5G18770, AT5G19010, AT5G19020, AT5G19030, AT5G19050, AT5G19070, AT5G19090, AT5G19110, AT5G19040, AT5G19130, AT5G18780, AT3G06320, AT5G18790, AT5G18820, AT5G18850, AT5G18860, AT5G18890, AT5G18520, AT5G18470, AT5G18400, AT5G18500, AT5G18460, AT5G18490, AT5G18310, AT5G18525, AT5G18540, AT5G18550, AT5G18560, AT5G18570, AT5G18590, AT5G18620, AT5G18580, AT5G18640, AT5G18650, AT5G18680, AT5G18690, AT5G18750, AT5G18760, AT5G18660, AT5G18670, AT5G18630, AT5G18440, AT5G18450, AT5G18475, AT5G18040, AT5G17990, AT5G18070, AT5G18100, AT5G18140, AT5G18150, AT5G18180, AT5G18190, AT5G18210, AT5G18270, AT5G18090, AT5G18250, AT5G18130, AT5G18200, AT5G18120, AT5G18240, AT5G18170, AT5G18110, AT5G18280, AT5G18230, AT5G18340, AT5G18360, AT5G18370, AT5G18390, AT5G18410, AT5G18420, AT5G17680, AT5G17700, AT5G17780, AT5G17790, AT5G17800, AT5G17850, AT5G17860, AT5G17770, AT5G17660, AT5G17840, AT5G17870, AT5G17820, AT5G17690, AT5G17710, AT5G17760, AT5G17880, AT5G17890, AT5G17910, AT5G17930, AT5G17950, AT5G17960, AT5G17980, AT5G17210, AT5G17170, AT5G17310, AT5G17670, AT5G16660, AT5G16680, AT5G16690, AT5G16650, AT5G16620, AT5G17220, AT5G17240, AT5G17250, AT5G17260, AT5G17270, AT5G17290, AT5G17330, AT5G17360, AT5G17370, AT5G17380, AT5G17230, AT5G17190, AT5G17280, AT5G17350, AT5G17300, AT5G16570, AT5G16590, AT5G16600, AT5G16610, AT5G16630, AT5G16640, AT5G15330, AT5G15150, AT5G15230, AT5G15350, AT5G16290, AT5G16300, AT5G16320, AT5G16370, AT5G16340, AT5G16420, AT5G16430, AT5G16440, AT5G16470, AT5G16490, AT5G16520, AT5G16530, AT5G16480, AT5G16390, AT5G16310, AT5G16380, AT5G16540, AT5G16450, AT5G16510, AT5G16550, AT5G16560, AT5G16400, AT5G16505, AT5G15390, AT5G14610, AT5G14620, AT5G14640, AT5G14650, AT5G14670, AT5G14680, AT5G14690, AT5G14660, AT5G14600, AT5G14530, AT5G14700, AT5G14710, AT5G14570, AT5G15120, AT5G15130, AT5G15160, AT5G15170, AT5G15180, AT5G15190, AT5G15200, AT5G15210, AT5G15240, AT5G15280, AT5G15290, AT5G15300, AT5G15320, AT5G14260, AT5G14330, AT5G14340, AT5G14390, AT5G14420, AT5G14430, AT5G14440, AT5G14450, AT5G14460, AT5G14510, AT5G14240, AT5G14310, AT5G14500, AT5G14370, AT5G14250, AT5G14410, AT5G14320, AT5G14270, AT5G14520, AT5G14540, AT5G14550, AT5G14580, AT5G14590, AT5G14100, AT5G14130, AT5G14230, AT5G13970, AT5G13980, AT5G13990, AT5G14020, AT5G14040, AT5G13930, AT5G14000, AT5G14030, AT5G13890, AT5G13880, AT5G13960, AT5G13820, AT5G13870, AT5G14080, AT5G14120, AT5G14170, AT5G14180, AT5G14210, AT5G14220, AT5G14050, AT5G14150, AT5G14105, AT5G13780, AT5G13830, AT5G13840, AT5G13850, AT5G13900, AT5G13910, AT5G13950, AT5G13460, AT5G13420, AT5G13430, AT5G13440, AT5G13450, AT5G13560, AT5G13570, AT5G13580, AT5G13590, AT5G13630, AT5G13680, AT5G13690, AT5G13700, AT5G13720, AT5G13740, AT5G13760, AT5G13610, AT5G13750, AT5G13640, AT5G13710, AT5G13650, AT5G13770, AT5G13790, AT5G13800, AT5G13810, AT5G13400, AT5G13410, AT5G13470, AT5G12890, AT5G12900, AT5G12910, AT5G12930, AT5G12950, AT5G12960, AT5G12970, AT5G12980, AT5G12990, AT5G13000, AT5G13010, AT5G13020, AT5G13030, AT5G12940, AT5G12860, AT5G13200, AT5G13240, AT5G13290, AT5G13300, AT5G13320, AT5G13330, AT5G13190, AT5G13310, AT5G13280, AT5G13220, AT5G13370, AT5G13360, AT5G13380, AT5G13390, AT5G12020, AT5G11980, AT5G11860, AT5G11910, AT5G11890, AT5G11970, AT5G11920, AT5G12100, AT5G12110, AT5G12150, AT5G12170, AT5G12220, AT5G12230, AT5G12240, AT5G12260, AT5G12210, AT5G12190, AT5G12130, AT5G12140, AT5G12250, AT5G12200, AT5G12120, AT5G12840, AT5G12850, AT5G12870, AT5G12880, AT5G12040, AT5G12080, AT5G12050, AT5G11780, AT5G11800, AT5G11810, AT5G11680, AT5G11690, AT5G11770, AT5G11650, AT5G11720, AT5G11790, AT5G11760, AT5G11670, AT5G11840, AT5G11850, AT5G11870, AT5G11900, AT5G11930, AT5G11950, AT5G11960, AT5G11990, AT5G12010, AT5G12030, AT5G11490, AT5G11630, AT5G11640, AT5G11700, AT5G11710, AT5G11730, AT5G11740, AT5G11350, AT5G11370, AT5G11380, AT5G11390, AT5G11410, AT5G11420, AT5G11170, AT5G11200, AT5G11280, AT5G11300, AT5G11430, AT5G11450, AT5G11460, AT5G11470, AT5G11530, AT5G11540, AT5G11550, AT5G11560, AT5G11570, AT5G11590, AT5G11600, AT5G11580, AT5G11480, AT5G11500, AT5G11520, AT5G11510, AT5G11320, AT5G11340, AT5G10850, AT5G10900, AT5G10910, AT5G10950, AT5G10970, AT5G10980, AT5G11000, AT5G10750, AT5G10740, AT5G10780, AT5G10940, AT5G10920, AT5G10960, AT5G10790, AT5G10810, AT5G10870, AT5G10930, AT5G10860, AT5G11010, AT5G11150, AT5G11160, AT5G11210, AT5G11230, AT5G11240, AT5G11250, AT5G11260, AT5G11270, AT5G10840, AT5G10600, AT5G10610, AT5G10550, AT5G10510, AT5G10480, AT5G10470, AT5G10460, AT5G10400, AT5G10390, AT5G10380, AT5G10350, AT5G10430, AT5G10580, AT5G10450, AT5G10360, AT5G10540, AT5G10490, AT5G10520, AT5G10560, AT5G10770, AT5G10800, AT5G10820, AT5G10830, AT5G10280, AT5G10270, AT5G10630, AT5G10620, AT5G09830, AT5G09980, AT5G09900, AT5G09880, AT5G09810, AT5G09860, AT5G09850, AT5G10010, AT5G10020, AT5G10030, AT5G10040, AT5G10070, AT5G10100, AT5G10120, AT5G10130, AT5G10160, AT5G10170, AT5G10210, AT5G10230, AT5G10180, AT5G10190, AT5G10260, AT5G10290, AT5G10310, AT5G10320, AT5G10300, AT5G10240, AT5G09990, AT5G09672, AT5G09670, AT5G09690, AT5G09720, AT5G09710, AT5G09760, AT5G09790, AT5G09800, AT5G09740, AT5G09770, AT5G09650, AT5G09630, AT5G09530, AT5G09590, AT5G09820, AT5G09840, AT5G09870, AT5G09950, AT5G09920, AT5G09960, AT5G09995, AT5G09890, AT5G09550, AT5G09570, AT5G09620, AT5G08260, AT5G08139, AT5G08460, AT5G08470, AT5G08490, AT5G08500, AT5G08510, AT5G08520, AT5G08550, AT5G08570, AT5G08590, AT5G08600, AT5G08610, AT5G08630, AT5G08650, AT5G08540, AT5G08640, AT5G08530, AT5G08480, AT5G08535, AT5G08565, AT5G08620, AT5G08560, AT5G08580, AT5G09520, AT5G08270, AT5G08190, AT5G08080, AT5G08160, AT5G08180, AT5G08100, AT5G08120, AT5G07860, AT5G07870, AT5G07890, AT5G07960, AT5G07900, AT5G07950, AT5G07920, AT5G07842, AT5G07840, AT5G07990, AT5G08010, AT5G08020, AT5G08040, AT5G08050, AT5G08060, AT5G08000, AT5G08070, AT5G08110, AT5G08130, AT5G08170, AT5G08200, AT5G08210, AT5G08240, AT5G08250, AT5G07910, AT5G07440, AT5G07300, AT5G07580, AT5G07590, AT5G07620, AT5G07630, AT5G07710, AT5G07720, AT5G07740, AT5G07760, AT5G07770, AT5G07780, AT5G07680, AT5G07690, AT5G07730, AT5G07800, AT5G07810, AT5G07940, AT5G07980, AT5G07820, AT5G07830, AT5G07570, AT5G07370, AT5G07470, AT5G07270, AT5G07290, AT5G07220, AT5G07190, AT5G07250, AT5G07320, AT5G07330, AT5G07340, AT5G07360, AT5G07390, AT5G07460, AT5G07520, AT5G07150, AT5G06780, AT5G06750, AT5G06700, AT5G06720, AT5G06770, AT5G06760, AT5G06690, AT5G06850, AT5G06900, AT5G06930, AT5G06940, AT5G06960, AT5G06970, AT5G07000, AT5G07040, AT5G07030, AT5G07010, AT5G07110, AT5G07080, AT5G06980, AT5G07100, AT5G07090, AT5G07020, AT5G06860, AT5G06950, AT5G06910, AT5G07070, AT5G07130, AT5G06260, AT5G06320, AT5G06550, AT5G06570, AT5G06630, AT5G06640, AT5G06670, AT5G06680, AT5G06660, AT5G06510, AT5G06610, AT5G06530, AT5G06580, AT5G06560, AT5G06600, AT5G06710, AT5G06740, AT5G06800, AT5G06810, AT5G06830, AT5G06839, AT5G06730, AT5G06360, AT5G06265, AT5G06340, AT5G05920, AT5G05950, AT5G06080, AT5G06100, AT5G06110, AT5G06120, AT5G06130, AT5G06140, AT5G06160, AT5G06200, AT5G06240, AT5G06250, AT5G06180, AT5G06210, AT5G06190, AT5G06300, AT5G06330, AT5G06400, AT5G06410, AT5G06430, AT5G06440, AT5G06460, AT5G06490, AT5G06480, AT5G06290, AT5G06270, AT5G06280, AT1G01350, AT5G06420, AT5G06370, AT5G05730, AT5G05740, AT5G05760, AT5G05790, AT5G05800, AT5G05820, AT5G05850, AT5G05880, AT5G05900, AT5G05710, AT5G05780, AT5G05870, AT5G05890, AT5G05690, AT5G05860, AT5G05700, AT5G05750, AT5G05940, AT5G05980, AT5G06000, AT5G06050, AT5G05990, AT5G06060, AT5G05960, AT5G05930, AT5G05660, AT5G05670, AT5G05680, AT5G05390, AT5G05400, AT5G05430, AT5G05450, AT5G05460, AT5G05320, AT5G05440, AT5G05500, AT5G05470, AT5G05410, AT5G05480, AT5G05510, AT5G05540, AT5G05560, AT5G05570, AT5G05640, AT5G05610, AT5G05520, AT5G05600, AT5G05270, AT5G05300, AT5G05310, AT5G05340, AT5G05350, AT5G05370, AT5G04960, AT5G04970, AT5G04980, AT5G05030, AT5G05130, AT5G05140, AT5G05090, AT5G05110, AT5G04900, AT5G05000, AT5G05080, AT5G05040, AT5G05060, AT5G05010, AT5G04940, AT5G04930, AT5G05100, AT5G05160, AT5G05190, AT5G05230, AT5G05180, AT5G05200, AT5G05210, AT5G05220, AT5G05170, AT5G05250, AT5G04720, AT5G04910, AT5G04920, AT5G04950, AT5G04540, AT5G04560, AT5G04620, AT5G04490, AT5G04430, AT5G04470, AT5G04600, AT5G04590, AT5G04480, AT5G04510, AT5G04410, AT5G04550, AT5G04420, AT5G04670, AT5G04700, AT5G04680, AT5G04710, AT5G04810, AT5G04790, AT5G04760, AT5G04770, AT5G04740, AT2G04390, AT5G04800, AT2G05220, AT5G04750, AT5G03870, AT5G03900, AT5G03905, AT5G03880, AT5G03860, AT5G03930, AT5G03960, AT5G04020, AT5G04040, AT5G04050, AT5G04060, AT5G04070, AT5G04080, AT5G04000, AT5G03970, AT5G03940, AT5G03910, AT5G04440, AT5G04460, AT5G04520, AT5G04530, AT5G03760, AT5G03850, AT5G03560, AT5G03430, AT5G03300, AT5G03340, AT5G03470, AT5G03530, AT5G03640, AT5G03650, AT5G03670, AT5G03700, AT5G03720, AT5G03730, AT5G03740, AT5G03770, AT5G03780, AT5G03630, AT5G03690, AT5G03610, AT5G03660, AT5G03390, AT5G03460, AT5G03345, AT5G03455, AT5G03440, AT5G03370, AT5G03555, AT5G03270, AT5G03280, AT5G03290, AT5G03160, AT5G03200, AT5G03220, AT5G03500, AT5G03170, AT5G03030, AT5G03240, AT5G03230, AT5G03180, AT5G03040, AT5G03320, AT5G03360, AT5G03380, AT5G03420, AT5G03450, AT5G03490, AT5G03510, AT5G03550, AT5G03570, AT5G03415, AT5G03520, AT5G03190, AT5G03210, AT5G03260, AT5G02980, AT5G02990, AT5G03000, AT5G03020, AT5G02910, AT5G02790, AT5G02890, AT5G02740, AT5G02770, AT5G02810, AT5G02840, AT5G02870, AT5G02820, AT5G03050, AT5G03060, AT5G03070, AT5G03080, AT5G03130, AT5G03140, AT5G03150, AT5G02780, AT5G02800, AT5G02830, AT5G02850, AT5G02860, AT5G02880, AT5G02900, AT5G02290, AT5G02380, AT5G02260, AT5G02280, AT5G02490, AT5G02500, AT5G02530, AT5G02620, AT5G02630, AT5G02650, AT5G02670, AT5G02680, AT5G02690, AT5G02590, AT5G02600, AT5G02610, AT5G02710, AT5G02640, AT5G02550, AT5G02560, AT5G02580, AT5G02540, AT5G02520, AT5G02480, AT5G02760, AT5G02450, AT5G02420, AT5G02270, AT5G02140, AT5G02150, AT5G02170, AT5G02180, AT5G02200, AT5G02230, AT5G02050, AT5G02130, AT5G02120, AT5G02030, AT5G01960, AT5G02040, AT5G02160, AT5G02100, AT5G02240, AT5G02020, AT5G02250, AT5G02310, AT5G02350, AT2G13910, AT5G02360, AT5G02370, AT5G02410, AT5G02430, AT5G02440, AT5G02470, AT5G01490, AT5G01540, AT5G01712, AT5G01710, AT5G01770, AT5G01780, AT5G01790, AT5G01810, AT5G01820, AT5G01830, AT5G01840, AT5G01850, AT5G01860, AT5G01870, AT5G01880, AT5G01910, AT5G01920, AT5G01930, AT5G01940, AT5G01950, AT5G01740, AT5G01750, AT5G01800, AT5G01890, AT5G01970, AT5G01980, AT5G01990, AT5G02010, AT5G01530, AT5G01590, AT5G01520, AT5G01450, AT5G01460, AT5G01480, AT5G01390, AT5G01340, AT5G01410, AT5G01470, AT5G01360, AT5G01350, AT5G01510, AT5G01550, AT5G01650, AT5G01660, AT5G01670, AT5G01700, AT5G01720, AT5G01730, AT5G01500, AT5G01610, AT5G01620, AT5G01600, AT5G01260, AT5G01310, AT5G01320, AT5G01330, AT5G01370, AT5G01380, AT5G01400, AT3G63470, AT3G63390, AT3G63410, AT3G63510, AT3G63490, AT3G63420, AT5G01020, AT5G01030, AT5G01050, AT5G01040, AT5G01060, AT5G01070, AT5G01200, AT5G01240, AT5G01270, AT5G01290, AT5G01300, AT5G01160, AT5G01230, AT5G01090, AT5G01075, AT5G01015, AT5G01220, AT5G01210, AT3G63500, AT3G63520, AT3G63180, AT3G62940, AT3G63120, AT3G63170, AT3G63130, AT3G63000, AT3G63110, AT3G63140, AT3G63150, AT3G63240, AT3G63290, AT3G63300, AT3G63310, AT3G63330, AT3G63210, AT3G63260, AT3G63220, AT3G63190, AT3G63270, AT3G63200, AT3G63250, AT3G63380, AT3G63400, AT3G63460, AT3G62820, AT3G62600, AT3G62630, AT3G62840, AT3G62870, AT3G62790, AT3G62770, AT3G62730, AT3G62650, AT3G62690, AT3G62590, AT3G62720, AT3G62910, AT3G62920, AT3G62930, AT3G62970, AT3G62980, AT3G63010, AT3G63040, AT3G63070, AT3G63080, AT3G63090, AT3G63050, AT3G62880, AT3G62890, AT3G62810, AT3G62470, AT3G62540, AT5G14820, AT3G62510, AT4G27597, AT3G62520, AT3G62560, AT3G62390, AT3G62400, AT3G62580, AT3G62610, AT3G62620, AT3G62660, AT3G62680, AT3G62700, AT3G62740, AT3G62760, AT3G62780, AT3G62800, AT3G62830, AT2G47650, AT3G62860, AT3G62380, AT3G62420, AT3G62422, AT3G62430, AT3G62450, AT3G62460, AT3G62530, AT3G61860, AT3G61870, AT3G62060, AT3G62090, AT3G62100, AT3G62140, AT3G62150, AT3G62160, AT3G62220, AT3G62240, AT3G62270, AT3G62280, AT3G62300, AT3G62290, AT3G62260, AT3G62130, AT3G62110, AT3G62080, AT3G62190, AT3G62120, AT3G62310, AT3G62330, AT3G62350, AT3G62360, AT3G62370, AT3G62050, AT3G61890, AT3G61960, AT3G61740, AT3G61760, AT3G61780, AT3G61800, AT3G61640, AT3G61630, AT3G61790, AT3G61840, AT3G61770, AT3G61710, AT3G61820, AT3G61620, AT3G61830, AT3G61850, AT3G61900, AT3G61930, AT3G62000, AT3G62010, AT3G62020, AT3G62040, AT3G61950, AT3G61980, AT3G61970, AT3G61990, AT3G62030, AT3G61260, AT3G61200, AT3G61130, AT3G61220, AT3G61150, AT3G61140, AT3G61120, AT3G61360, AT3G61180, AT3G61410, AT5G28350, AT3G61480, AT3G61490, AT3G61570, AT3G61610, AT3G61530, AT3G61460, AT3G61440, AT3G61580, AT3G61430, AT3G61470, AT5G28450, AT3G61590, AT3G61540, AT3G61600, AT3G61550, AT3G61650, AT5G05620, AT3G61670, AT3G61690, AT3G61390, AT3G61400, AT3G61190, AT3G60820, AT3G60600, AT3G60830, AT3G60770, AT3G60690, AT3G60620, AT3G60980, AT3G61010, AT3G61020, AT3G11030, AT3G61030, AT3G60950, AT3G61050, AT3G61070, AT3G61080, AT3G61100, AT3G61060, AT3G61110, AT3G61160, AT3G61170, AT3G61210, AT3G61230, AT3G61240, AT3G61250, AT3G61310, AT3G61340, AT3G61350, AT3G61380, AT3G60480, AT3G60450, AT3G60360, AT3G60520, AT3G60530, AT3G60390, AT3G60410, AT3G60630, AT3G60660, AT3G60680, AT3G60700, AT3G60720, AT3G60740, AT3G60800, AT3G60810, AT3G60840, AT3G60850, AT3G60860, AT3G60910, AT3G60880, AT3G60640, AT3G60900, AT2G45470, AT3G60750, AT3G60570, AT3G60590, AT3G60420, AT3G60270, AT3G60300, AT3G60310, AT3G60330, AT3G60260, AT3G60350, AT3G60340, AT3G60245, AT3G60250, AT3G60220, AT3G60320, AT3G60370, AT3G60380, AT3G60400, AT3G60440, AT3G60470, AT3G60490, AT3G60510, AT3G60540, AT3G60550, AT3G60210, AT3G60180, AT3G60130, AT3G60140, AT3G60190, AT3G60110, AT3G60240, AT3G59820, AT3G59900, AT3G59910, AT3G59930, AT5G33355, AT3G59950, AT3G60010, AT3G59980, AT3G59940, AT3G59990, AT3G59870, AT3G59840, AT3G59810, AT3G59920, AT3G60030, AT3G60040, AT3G60050, AT3G60070, AT3G60080, AT3G60120, AT3G60970, AT3G60160, AT3G60200, AT3G59770, AT3G59790, AT3G59800, AT3G59380, AT3G59360, AT3G59470, AT3G59530, AT3G59630, AT3G59660, AT3G59670, AT3G59680, AT3G59690, AT3G59700, AT3G59710, AT3G59730, AT3G59750, AT3G59740, AT3G59650, AT3G59640, AT3G59550, AT3G59540, AT3G59760, AT3G59440, AT3G59460, AT3G59490, AT3G59480, AT3G59280, AT3G59300, AT3G59350, AT3G59020, AT3G59040, AT3G59100, AT3G59110, AT3G59120, AT3G59052, AT3G59050, AT3G59090, AT3G59080, AT3G58970, AT3G59010, AT3G59220, AT3G59310, AT3G59320, AT3G59370, AT3G59390, AT3G59410, AT3G59430, AT3G58975, AT3G58990, AT3G59000, AT3G58640, AT3G58650, AT3G58600, AT3G58570, AT3G58520, AT3G58550, AT3G58530, AT3G58460, AT3G58510, AT3G58540, AT3G58610, AT3G58470, AT3G58660, AT3G58690, AT3G58750, AT3G58760, AT3G58770, AT3G58790, AT3G58810, AT3G58830, AT3G58800, AT3G58680, AT3G58700, AT3G58710, AT3G58670, AT3G58840, AT3G58730, AT3G57810, AT3G58010, AT3G57920, AT3G57870, AT3G57890, AT3G57880, AT3G58190, AT3G58230, AT3G58280, AT3G58330, AT3G58140, AT3G58100, AT3G58120, AT3G58200, AT3G58350, AT3G58180, AT3G58170, AT3G58560, AT3G58580, AT3G58620, AT3G58630, AT3G58090, AT3G58040, AT3G57690, AT3G57680, AT3G57660, AT3G57630, AT3G57650, AT3G57750, AT3G57600, AT3G57610, AT3G57710, AT3G57800, AT3G57785, AT3G57760, AT3G57820, AT3G57830, AT3G57860, AT3G57930, AT3G57940, AT3G57950, AT3G57960, AT3G57970, AT3G57980, AT3G57990, AT3G58050, AT3G58060, AT3G57700, AT3G57340, AT3G57390, AT3G57280, AT3G57220, AT3G57290, AT3G57410, AT3G57420, AT3G57430, AT3G57440, AT3G57460, AT3G57480, AT3G57530, AT3G57570, AT3G57490, AT3G57560, AT3G57450, AT3G57470, AT3G57520, AT3G57550, AT3G57540, AT3G57790, AT3G57780, AT3G57330, AT3G57360, AT3G57370, AT3G57380, AT3G57130, AT3G57140, AT3G57170, AT3G57000, AT3G57020, AT3G57090, AT3G57160, AT3G56950, AT3G57080, AT3G57070, AT3G56940, AT3G57040, AT3G57050, AT3G57150, AT3G57010, AT3G57190, AT3G57230, AT3G57300, AT3G57320, AT3G56980, AT3G56990, AT3G57030, AT3G57060, AT3G57120, AT3G56410, AT3G56430, AT3G56440, AT3G56460, AT3G56480, AT3G56510, AT3G56590, AT3G56630, AT3G56640, AT3G56650, AT3G56360, AT3G56400, AT3G56620, AT3G56490, AT3G56580, AT3G56930, AT3G56960, AT3G56120, AT3G56080, AT3G56370, AT3G55850, AT3G56100, AT3G56140, AT3G56160, AT3G56450, AT3G56190, AT3G56200, AT3G56230, AT3G56290, AT3G56300, AT3G56310, AT3G56330, AT3G56350, AT3G56110, AT3G56240, AT3G56270, AT3G56090, AT3G56130, AT3G56340, AT3G56150, AT3G56170, AT3G56070, AT3G56040, AT3G56050, AT3G55890, AT3G56010, AT3G55980, AT3G56060, AT3G55690, AT3G55720, AT3G55740, AT3G55760, AT3G55780, AT3G55820, AT3G55640, AT3G55770, AT3G55630, AT3G55605, AT3G55700, AT3G55800, AT3G55730, AT3G55830, AT3G55910, AT3G55940, AT3G55950, AT3G55970, AT3G56000, AT3G55920, AT3G55960, AT3G55840, AT2G39800, AT3G55610, AT3G55620, AT3G55660, AT3G55300, AT3G55310, AT3G55290, AT3G55320, AT3G55260, AT3G55280, AT3G55270, AT3G55410, AT3G55420, AT3G55450, AT3G55470, AT3G55580, AT3G55390, AT3G55360, AT3G55560, AT3G55460, AT3G55520, AT3G55510, AT3G55440, AT3G55380, AT3G55350, AT3G55430, AT3G55530, AT3G55370, AT3G55400, AT3G55480, AT3G55600, AT3G55250, AT3G54990, AT3G54970, AT3G55000, AT3G54890, AT3G54900, AT3G55005, AT3G54840, AT3G54860, AT3G55030, AT3G55040, AT3G55050, AT3G55060, AT3G55080, AT3G55090, AT3G55110, AT3G55120, AT3G55070, AT3G55020, AT3G55010, AT3G55140, AT3G55150, AT3G55160, AT3G55170, AT3G55200, AT3G55220, AT3G54950, AT3G54960, AT3G54980, AT3G54580, AT3G54590, AT3G54630, AT3G54540, AT3G54560, AT3G54640, AT3G54620, AT3G54610, AT3G54650, AT3G54670, AT3G54750, AT3G54790, AT3G54800, AT3G54690, AT3G54720, AT3G54770, AT3G54820, AT3G54680, AT3G54660, AT3G54810, AT3G54850, AT3G54870, AT3G54920, AT3G54930, AT3G54350, AT3G54480, AT3G54500, AT3G54510, AT3G54230, AT3G54270, AT3G54280, AT3G54300, AT3G54310, AT3G54200, AT3G54290, AT3G54250, AT3G54140, AT3G54210, AT3G54150, AT3G54260, AT3G54170, AT3G54190, AT3G54080, AT3G54220, AT3G54320, AT3G54380, AT3G54420, AT3G54390, AT3G54360, AT3G54400, AT3G54430, AT3G54100, AT3G54110, AT3G54120, AT3G54130, AT3G53710, AT3G53760, AT3G53810, AT3G53930, AT3G53940, AT3G53960, AT3G53970, AT3G54040, AT3G53900, AT3G53890, AT3G54030, AT3G53880, AT3G53730, AT3G54000, AT3G53740, AT3G53990, AT3G53980, AT3G53920, AT3G53780, AT3G53950, AT3G54010, AT3G54060, AT3G54070, AT3G54090, AT3G53700, AT3G53400, AT3G53402, AT3G53430, AT3G53440, AT3G53450, AT3G53470, AT3G53480, AT3G53500, AT3G53510, AT3G53520, AT3G53540, AT3G53580, AT3G53590, AT3G53600, AT3G53630, AT1G55710, AT4G18692, AT3G53650, AT3G53668, AT3G53670, AT3G53460, AT3G53690, AT3G53560, AT3G53410, AT3G53610, AT3G53620, AT3G53420, AT3G53570, AT3G53370, AT3G53360, AT3G53380, AT3G53390, AT3G53100, AT3G53130, AT3G53150, AT3G53160, AT3G53180, AT3G53200, AT3G53230, AT3G53240, AT3G53270, AT3G53190, AT3G53210, AT3G53260, AT3G53220, AT3G53280, AT3G53110, AT3G53340, AT3G53350, AT3G52960, AT3G52890, AT3G52940, AT3G52840, AT3G53020, AT3G53090, AT3G52710, AT3G52750, AT3G52760, AT3G52780, AT3G52820, AT3G52610, AT3G52800, AT3G52740, AT3G52730, AT3G52860, AT3G52870, AT3G52950, AT3G53000, AT3G52930, AT3G52920, AT3G52880, AT3G52900, AT3G53030, AT3G52850, AT3G52640, AT3G52660, AT5G28390, AT3G52140, AT3G52150, AT3G51950, AT3G52040, AT3G52070, AT3G51920, AT3G52120, AT3G52155, AT3G52060, AT3G52090, AT3G51990, AT3G52390, AT3G52420, AT3G52450, AT3G52460, AT3G52490, AT3G52500, AT3G52510, AT3G52550, AT3G52570, AT3G52400, AT3G52580, AT3G52590, AT3G52480, AT3G52470, AT3G52560, AT3G52430, AT3G51520, AT3G51610, AT3G51550, AT3G51370, AT3G51440, AT3G51640, AT3G51650, AT3G51690, AT3G51710, AT3G51750, AT3G51730, AT3G51660, AT3G51720, AT3G51740, AT3G51630, AT3G51632, AT3G51670, AT3G51910, AT3G51940, AT3G51960, AT3G51970, AT3G52030, AT3G52110, AT3G51390, AT3G51500, AT3G51260, AT3G51000, AT3G51180, AT3G51090, AT3G51330, AT3G51250, AT3G51110, AT3G51300, AT3G50970, AT3G51410, AT3G51460, AT3G51470, AT3G51480, AT3G51490, AT3G51530, AT3G51540, AT3G51580, AT3G51620, AT3G51450, AT3G51600, AT3G51510, AT3G51430, AT3G51030, AT3G51130, AT3G51160, AT3G51140, AT3G51240, AT3G51010, AT3G51040, AT3G50950, AT3G50960, AT3G50870, AT3G50930, AT3G50790, AT3G50900, AT3G50910, AT3G50830, AT3G50770, AT3G50980, AT3G51050, AT3G51100, AT3G51120, AT3G51200, AT3G51270, AT3G51280, AT3G51290, AT3G51310, AT3G51320, AT3G51360, AT3G50880, AT3G50920, AT3G50430, AT3G50530, AT3G50590, AT3G50610, AT3G50620, AT3G50550, AT3G50500, AT3G50560, AT3G50520, AT3G50480, AT3G50640, AT3G50650, AT3G50690, AT3G50700, AT3G50750, AT3G50760, AT3G50630, AT3G50685, AT3G50670, AT3G50740, AT3G50660, AT3G50780, AT3G50850, AT3G50860, AT3G50070, AT3G50170, AT3G50180, AT3G50000, AT3G50060, AT3G50110, AT3G50190, AT3G50200, AT3G50230, AT3G50250, AT3G50280, AT3G50300, AT3G50340, AT3G50350, AT3G50360, AT3G50370, AT3G50380, AT3G50400, AT3G50410, AT3G50310, AT3G50210, AT3G50260, AT3G50240, AT3G50420, AT3G50140, AT3G50150, AT3G49940, AT3G49830, AT3G49845, AT3G49850, AT3G49860, AT3G49880, AT3G49920, AT3G49890, AT3G49810, AT3G49720, AT3G49760, AT3G49690, AT3G49780, AT3G49910, AT3G49930, AT3G49960, AT3G49990, AT3G50010, AT3G50050, AT3G50080, AT3G50130, AT3G49710, AT3G49725, AT3G49740, AT3G49750, AT3G49790, AT3G49820, AT3G49180, AT3G49250, AT3G49220, AT3G49430, AT3G49500, AT3G49590, AT3G49630, AT3G49640, AT3G49650, AT3G49580, AT3G49560, AT5G01430, AT3G49420, AT3G49670, AT3G49660, AT3G49680, AT3G49600, AT3G49490, AT3G49470, AT3G49530, AT3G49320, AT3G49360, AT3G48960, AT3G49050, AT3G49070, AT3G49080, AT3G49140, AT3G49120, AT3G49110, AT3G49000, AT3G48990, AT3G49010, AT3G49100, AT3G48970, AT3G48930, AT3G49060, AT3G49160, AT3G49190, AT3G49210, AT3G49240, AT3G49310, AT3G49340, AT3G49350, AT3G49370, AT3G49380, AT3G49390, AT3G48690, AT3G48700, AT3G48730, AT3G48710, AT3G48510, AT3G48550, AT3G48530, AT3G48570, AT3G48560, AT3G48680, AT3G48740, AT3G48760, AT3G48780, AT3G48790, AT3G48810, AT3G48830, AT3G48850, AT3G48860, AT3G48880, AT3G48750, AT3G48890, AT3G48900, AT3G48920, AT3G48940, AT3G48610, AT3G48620, AT3G44160, AT3G48640, AT3G48650, AT3G48140, AT3G48150, AT3G48190, AT3G48210, AT3G48185, AT3G48200, AT3G48170, AT3G48250, AT3G48410, AT3G48425, AT3G48440, AT3G48490, AT3G48500, AT3G48460, AT3G48450, AT3G48350, AT3G48360, AT3G48520, AT3G48070, AT3G48000, AT3G48090, AT3G48100, AT3G48040, AT3G47930, AT3G47960, AT3G47570, AT3G47730, AT3G47770, AT3G47780, AT3G47810, AT3G47800, AT3G47850, AT3G47833, AT3G47836, AT3G47860, AT3G47890, AT3G47910, AT3G47940, AT3G47950, AT3G47980, AT3G47990, AT3G48020, AT3G48030, AT3G48060, AT3G48050, AT3G48080, AT3G48110, AT3G48120, AT3G47670, AT3G47520, AT3G47600, AT3G47650, AT3G47450, AT3G47295, AT3G47370, AT3G47420, AT3G47340, AT3G47460, AT3G47480, AT3G47490, AT3G47510, AT3G47530, AT3G47540, AT3G47550, AT3G47590, AT3G47610, AT3G47620, AT3G47630, AT3G47640, AT3G47500, AT3G47470, AT3G47700, AT3G47680, AT3G47560, AT5G44415, AT1G36080, AT1G23930, AT3G47330, AT4G09370, AT3G47380, AT3G47390, AT3G47400, AT3G46940, AT3G46960, AT3G46980, AT3G47000, AT3G47010, AT3G47040, AT3G47050, AT3G47060, AT3G47110, AT3G47120, AT3G47210, AT3G47290, AT3G47220, AT3G47250, AT3G47070, AT3G47160, AT3G44500, AT3G47260, AT3G30440, AT3G47270, AT1G32830, AT3G47080, AT3G46970, AT3G46920, AT3G46930, AT3G46820, AT3G46830, AT3G46610, AT5G14350, AT3G46620, AT5G59570, AT3G46640, AT3G46540, AT3G46560, AT3G46580, AT3G46630, AT3G46670, AT3G46600, AT3G46690, AT3G46510, AT3G46700, AT3G46710, AT3G46720, AT3G46730, AT3G46740, AT3G46760, AT3G46790, AT3G46800, AT3G46810, AT3G46840, AT3G46850, AT3G46880, AT3G46900, AT3G46590, AT3G46180, AT3G46200, AT3G46210, AT3G46270, AT3G46280, AT3G46290, AT3G46220, AT3G46060, AT3G46230, AT3G46100, AT3G46340, AT3G46350, AT3G46360, AT3G46400, AT3G46430, AT3G46450, AT3G46440, AT3G46460, AT3G46490, AT3G46500, AT3G46110, AT3G46130, AT3G45700, AT3G45710, AT3G45720, AT3G45730, AT3G45740, AT3G45750, AT3G45770, AT3G45780, AT3G45830, AT3G45850, AT3G45870, AT3G45880, AT3G45900, AT3G46330, AT3G45920, AT3G45960, AT3G45990, AT3G46020, AT3G46030, AT3G45980, AT3G46320, AT3G45930, AT3G45970, AT3G46010, AT3G46000, AT3G46040, AT3G46070, AT3G45410, AT3G45420, AT3G45300, AT3G45290, AT3G45260, AT3G45430, AT3G45440, AT3G45450, AT3G48870, AT3G45530, AT3G45620, AT3G45630, AT3G45660, AT3G45650, AT3G45670, AT3G45600, AT3G45640, AT3G45590, AT3G45680, AT3G45330, AT3G45390, AT3G45030, AT5G62300, AT3G45040, AT3G45050, AT3G45070, AT3G45080, AT3G45010, AT3G44990, AT3G45090, AT3G45130, AT3G45160, AT3G45190, AT3G45230, AT3G45242, AT3G45240, AT3G45100, AT3G45140, AT3G45210, AT3G45310, AT3G44735, AT3G44750, AT3G44940, AT3G44960, AT3G44970, AT3G44470, AT2G15210, AT2G07250, AT3G47280, AT2G02190, AT5G34890, AT2G15150, AT3G44660, AT3G44490, AT4G04010, AT3G44510, AT3G44530, AT3G44540, AT3G44550, AT3G44590, AT3G44600, AT3G44610, AT3G44620, AT3G44630, AT3G44680, AT3G44690, AT3G44700, AT3G44720, AT3G44730, AT3G44740, AT3G44760, AT3G44430, AT3G44440, AT3G44450, AT3G44340, AT3G44070, AT3G44140, AT3G44200, AT3G44210, AT3G44100, AT3G44110, AT3G44190, AT3G44240, AT3G44280, AT3G44320, AT3G44300, AT3G44310, AT3G44260, AT3G44330, AT3G44370, AT3G44380, AT3G44400, AT3G44410, AT3G44030, AT3G44050, AT3G43960, AT3G44010, AT3G43980, AT3G43670, AT3G43690, AT3G43700, AT3G43730, AT3G43740, AT3G43790, AT3G43840, AT3G43720, AT3G43800, AT3G43810, AT3G43900, AT3G43920, AT3G43520, AT3G43540, AT3G43050, AT3G43160, AT3G43210, AT3G43220, AT3G43240, AT3G43250, AT3G43270, AT3G43280, AT3G43300, AT3G43230, AT3G43190, AT3G43430, AT3G43470, AT3G43480, AT3G43510, AT3G43550, AT1G58520, AT1G59030, AT1G58725, AT1G59406, AT3G43570, AT3G42630, AT3G42725, AT3G42790, AT3G42800, AT3G42820, AT3G42910, AT3G42960, AT3G42950, AT3G43020, AT3G42660, AT3G42670, AT1G21930, AT3G42150, AT3G42220, AT3G42290, AT3G42330, AT3G42600, AT3G42640, AT4G39860, AT4G39955, AT4G40045, AT4G40030, AT4G39890, AT4G40050, AT4G39950, AT4G39960, AT4G40060, AT4G39850, AT4G39980, AT4G39910, AT4G40090, AT4G40070, AT3G42070, AT3G42130, AT3G42050, AT3G42170, AT3G42190, AT4G40040, AT4G39880, AT4G40080, AT4G39900, AT4G39370, AT4G39660, AT4G39690, AT4G39770, AT4G39780, AT4G39790, AT4G39820, AT4G39830, AT4G39800, AT4G39740, AT4G39840, AT4G39870, AT4G39920, AT4G39930, AT4G39940, AT4G40000, AT4G40010, AT4G40020, AT4G39990, AT4G39630, AT4G39460, AT4G39390, AT4G39730, AT4G39610, AT4G39675, AT4G39520, AT4G39170, AT4G39260, AT4G39350, AT4G39240, AT4G39210, AT4G39380, AT4G39400, AT4G39420, AT4G39440, AT4G39450, AT4G39480, AT4G39530, AT4G39540, AT4G39550, AT4G39560, AT4G39570, AT4G39620, AT4G39720, AT4G39640, AT4G39670, AT4G39680, AT4G39590, AT4G39510, AT4G39200, AT4G39220, AT4G38810, AT4G38950, AT4G38960, AT4G38980, AT4G39030, AT4G39040, AT4G39070, AT4G39100, AT4G38920, AT4G39090, AT4G38940, AT4G38970, AT4G39010, AT4G38930, AT4G39080, AT4G39120, AT4G39150, AT4G39160, AT4G39190, AT4G39230, AT4G39270, AT4G39280, AT4G39300, AT4G39330, AT4G39320, AT4G39140, AT4G39235, AT4G38600, AT4G38670, AT4G38700, AT4G38710, AT4G38570, AT4G38660, AT4G38630, AT4G38580, AT4G38680, AT4G38620, AT4G38640, AT4G38750, AT4G38780, AT4G38830, AT4G38870, AT4G38880, AT4G38890, AT4G38900, AT4G38770, AT4G38840, AT4G38740, AT4G38800, AT4G38430, AT4G38550, AT4G38590, AT4G38225, AT4G38040, AT4G38260, AT4G38130, AT4G37990, AT4G38350, AT4G38380, AT4G38410, AT4G38420, AT4G38440, AT4G38470, AT4G38520, AT4G38540, AT4G38480, AT4G38370, AT4G38460, AT4G38400, AT4G38510, AT4G38500, AT4G38360, AT4G38490, AT4G38530, AT4G38300, AT4G38650, AT4G38330, AT4G38100, AT4G38250, AT4G38090, AT4G38210, AT4G37930, AT4G37750, AT4G37890, AT4G37900, AT4G37910, AT4G37940, AT4G37970, AT4G38000, AT4G38010, AT4G38020, AT4G38050, AT4G38060, AT4G38070, AT4G38080, AT4G38120, AT4G38150, AT4G38160, AT4G38170, AT4G38180, AT4G38190, AT4G38200, AT4G38220, AT4G38230, AT4G38240, AT4G38270, AT4G37790, AT4G37760, AT4G37800, AT4G37870, AT4G37550, AT4G37540, AT4G37290, AT4G37445, AT4G37370, AT4G37295, AT4G37560, AT4G37300, AT4G37450, AT4G37490, AT4G37310, AT4G37470, AT4G37580, AT4G37630, AT4G37650, AT4G37670, AT4G37660, AT4G37680, AT4G38320, AT4G37610, AT4G37590, AT4G37640, AT4G37730, AT4G37740, AT4G37820, AT4G37880, AT4G37830, AT4G37410, AT4G36140, AT4G36150, AT4G36160, AT4G36180, AT4G36190, AT4G36195, AT4G36210, AT4G36250, AT4G36260, AT4G36280, AT4G36350, AT4G36290, AT4G36360, AT4G37380, AT4G37460, AT4G37480, AT4G37510, AT4G37330, AT4G37320, AT4G37340, AT4G37530, AT4G37520, AT4G37400, AT4G37430, AT4G36110, AT4G36010, AT4G35870, AT4G35880, AT4G35900, AT4G35920, AT4G35930, AT4G35940, AT4G35970, AT4G35985, AT4G35860, AT4G35910, AT4G35850, AT4G35905, AT4G35950, AT4G35980, AT4G35790, AT4G35890, AT4G35987, AT4G36030, AT4G36040, AT4G36050, AT4G36020, AT4G35510, AT4G35550, AT4G35580, AT4G35800, AT4G35830, AT4G35470, AT4G35500, AT4G35490, AT4G35450, AT4G35480, AT4G35440, AT4G35530, AT4G35540, AT4G35560, AT4G35590, AT4G35600, AT4G35620, AT4G35660, AT4G35690, AT4G35720, AT4G35730, AT4G35740, AT4G35780, AT4G35570, AT4G35760, AT4G35770, AT4G35630, AT4G35750, AT4G35320, AT4G35310, AT4G35120, AT4G35130, AT4G35040, AT4G35060, AT4G35110, AT4G35090, AT4G35050, AT4G35150, AT4G35160, AT4G35170, AT4G35200, AT4G35180, AT4G35190, AT4G35220, AT4G35230, AT4G35240, AT4G35300, AT4G35335, AT4G35350, AT4G35380, AT4G35400, AT1G48250, AT1G17275, AT1G78350, AT4G35420, AT4G35260, AT4G35250, AT4G35360, AT4G35410, AT4G34720, AT4G34620, AT4G34555, AT4G34710, AT4G34460, AT4G34490, AT4G34640, AT4G34830, AT4G34840, AT4G34880, AT4G34890, AT4G34900, AT4G34910, AT4G34950, AT4G34960, AT4G34970, AT4G34980, AT4G34990, AT4G34930, AT4G34920, AT4G34870, AT4G35000, AT4G34860, AT4G35020, AT4G35030, AT4G34630, AT4G34660, AT4G34700, AT4G34450, AT4G34340, AT4G34290, AT4G34265, AT4G34350, AT4G34370, AT4G34240, AT4G34480, AT4G34500, AT4G34520, AT4G34540, AT4G34560, AT4G34590, AT4G34588, AT4G34600, AT4G34610, AT4G34670, AT4G34680, AT4G34730, AT4G34740, AT4G34750, AT4G34650, AT4G34760, AT4G34360, AT4G34390, AT4G34400, AT4G34410, AT4G34430, AT4G34000, AT4G33950, AT4G34040, AT4G34080, AT2G45260, AT4G34100, AT4G34131, AT4G34135, AT4G34140, AT4G34160, AT4G34190, AT4G34180, AT4G34200, AT4G34215, AT4G34050, AT4G34230, AT4G34220, AT4G34030, AT4G34110, AT4G34138, AT4G34120, AT4G34090, AT4G34150, AT4G34250, AT4G34260, AT4G34270, AT4G34310, AT4G34320, AT4G33865, AT4G33985, AT4G33905, AT4G33740, AT4G33760, AT4G33770, AT4G33810, AT4G33560, AT4G33800, AT4G33580, AT4G33720, AT4G33660, AT4G33780, AT4G33640, AT4G33666, AT4G33650, AT4G33670, AT4G33680, AT4G33790, AT4G33630, AT4G33840, AT4G33830, AT4G33870, AT4G33890, AT4G34300, AT4G33930, AT4G33960, AT4G34020, AT4G33910, AT4G33980, AT4G33920, AT4G33940, AT4G33440, AT4G33450, AT4G33460, AT4G33480, AT4G33530, AT4G33490, AT4G33420, AT4G33510, AT4G33360, AT4G33500, AT4G33250, AT4G33470, AT4G33430, AT4G33520, AT4G33270, AT4G33260, AT4G33410, AT4G33540, AT4G33550, AT4G33700, AT4G33710, AT4G33370, AT4G33400, AT4G32940, AT4G33080, AT4G33090, AT4G33100, AT4G33110, AT4G33120, AT4G33130, AT4G33160, AT4G33170, AT4G33180, AT4G33200, AT4G33210, AT4G33240, AT4G33220, AT4G33150, AT4G33140, AT4G33290, AT4G33300, AT4G33310, AT4G33330, AT4G33350, AT4G33040, AT4G32900, AT4G32915, AT4G32930, AT4G33030, AT4G33010, AT4G32910, AT4G32680, AT4G32750, AT4G32590, AT4G32650, AT4G32690, AT4G32770, AT4G32660, AT4G32720, AT4G32710, AT4G32850, AT4G32860, AT4G32870, AT4G32880, AT4G32830, AT4G32840, AT4G32890, AT4G32920, AT4G32960, AT4G32970, AT4G33000, AT4G33020, AT4G33050, AT4G33070, AT4G32730, AT4G32760, AT4G32260, AT4G32240, AT4G32280, AT4G32330, AT4G32190, AT4G32390, AT4G32410, AT4G32420, AT4G32430, AT4G32440, AT4G32450, AT4G32500, AT4G32400, AT4G32470, AT4G32460, AT4G32520, AT4G32570, AT4G32560, AT4G32530, AT4G32551, AT4G32600, AT4G32605, AT4G32620, AT4G32630, AT4G32640, AT4G32700, AT4G31930, AT4G31860, AT4G31875, AT4G32020, AT4G32050, AT4G32060, AT4G32070, AT4G32150, AT4G32105, AT4G32030, AT4G32120, AT4G32040, AT4G32140, AT4G32160, AT4G32180, AT4G32210, AT5G09600, AT4G32250, AT4G32270, AT4G32290, AT4G32300, AT4G32320, AT4G32350, AT4G32360, AT4G31840, AT4G31990, AT4G31985, AT4G31910, AT4G31720, AT4G31800, AT4G31600, AT4G31700, AT4G31780, AT4G31790, AT4G31770, AT4G31810, AT4G31820, AT4G31850, AT4G31870, AT4G31880, AT4G31890, AT4G31920, AT4G32010, AT4G31940, AT4G31950, AT4G31980, AT4G31710, AT4G31730, AT4G31750, AT4G31320, AT4G31360, AT4G31390, AT4G31400, AT4G31240, AT4G31410, AT4G31300, AT4G31290, AT4G31340, AT4G31430, AT4G31330, AT4G31420, AT4G31470, AT4G31480, AT4G31490, AT4G31520, AT4G31530, AT4G31540, AT4G31590, AT4G31500, AT4G31550, AT4G31580, AT4G31560, AT4G31460, AT4G31450, AT4G31615, AT4G31670, AT4G31270, AT4G31310, AT4G31040, AT4G30950, AT4G30993, AT4G30930, AT4G30960, AT4G30996, AT4G30890, AT4G31050, AT4G31080, AT4G31100, AT4G31120, AT4G31140, AT4G31160, AT4G31180, AT4G31130, AT4G31150, AT4G31170, AT4G31200, AT4G31210, AT4G31250, AT4G31000, AT4G31020, AT4G31030, AT4G31060, AT4G30340, AT4G30610, AT4G30400, AT4G30660, AT4G30670, AT4G30680, AT4G30700, AT4G30740, AT4G30710, AT4G30790, AT4G30825, AT4G30840, AT4G30760, AT4G30820, AT4G30830, AT4G30730, AT4G30750, AT4G30690, AT4G30800, AT4G30860, AT4G30810, AT4G30900, AT4G30920, AT4G30910, AT4G30935, AT4G30990, AT4G30530, AT4G30330, AT4G30290, AT4G30190, AT4G30200, AT4G30310, AT4G30350, AT4G30360, AT4G30410, AT4G30420, AT4G30460, AT4G30520, AT4G30560, AT4G30570, AT4G30580, AT4G30600, AT4G30640, AT4G30650, AT4G30280, AT4G30450, AT4G30490, AT4G30440, AT4G30430, AT4G30480, AT4G30590, AT4G30620, AT4G30500, AT4G30390, AT4G30470, AT4G30550, AT4G30630, AT4G29960, AT4G29780, AT4G29870, AT4G29830, AT4G29810, AT4G29950, AT4G29940, AT4G29790, AT4G30020, AT4G30050, AT4G30060, AT4G30120, AT4G30150, AT4G30140, AT4G30130, AT4G30080, AT4G30160, AT4G30210, AT4G30270, AT4G30170, AT4G30220, AT4G30000, AT4G30010, AT4G29990, AT4G29820, AT4G29530, AT4G29560, AT4G29580, AT4G29610, AT4G29640, AT4G29690, AT4G29750, AT4G29520, AT4G29590, AT4G29660, AT4G29720, AT4G29670, AT4G29735, AT4G29510, AT4G29740, AT4G29700, AT4G29680, AT4G29800, AT4G29840, AT4G29890, AT4G29900, AT4G29910, AT4G29490, AT4G29130, AT4G29120, AT4G29210, AT4G29220, AT4G29230, AT4G29330, AT4G29380, AT4G29390, AT4G29420, AT4G29440, AT4G29270, AT4G29190, AT4G29240, AT4G29285, AT4G29340, AT4G29430, AT4G29350, AT4G29410, AT4G29360, AT4G29480, AT4G29260, AT4G29140, AT4G29170, AT4G29180, AT4G29160, AT4G28703, AT4G28750, AT4G28760, AT4G28706, AT4G28890, AT4G28940, AT4G29100, AT4G29050, AT4G28990, AT4G29080, AT4G29000, AT4G29070, AT4G28910, AT4G29020, AT4G29040, AT4G28980, AT4G28950, AT4G29060, AT4G29010, AT4G28830, AT4G28860, AT4G28880, AT4G28740, AT4G28770, AT4G28520, AT4G28550, AT4G28540, AT4G28430, AT4G28360, AT1G52370, AT4G28530, AT4G28440, AT4G28390, AT4G28450, AT4G28480, AT4G28490, AT4G28400, AT4G28590, AT4G28600, AT4G28610, AT4G28620, AT4G28630, AT4G28650, AT4G28570, AT4G28660, AT4G28640, AT4G28710, AT4G28720, AT4G28460, AT4G28140, AT4G28180, AT4G28200, AT4G28230, AT4G28260, AT4G28270, AT4G28300, AT4G28320, AT4G28220, AT4G28190, AT4G28240, AT4G28150, AT4G28290, AT4G28250, AT4G28210, AT4G28310, AT4G28330, AT4G28350, AT4G28370, AT4G28410, AT4G28030, AT4G27940, AT4G28025, AT4G27960, AT5G53300, AT4G28085, AT4G27970, AT4G28040, AT4G27652, AT4G27654, AT4G27800, AT4G27820, AT4G27840, AT4G27850, AT4G27880, AT4G27890, AT4G27780, AT4G27830, AT4G27860, AT4G27910, AT4G27930, AT4G27980, AT4G28010, AT4G28020, AT4G28110, AT4G28130, AT4G27900, AT4G28050, AT4G28100, AT4G27990, AT4G27600, AT4G27657, AT4G27700, AT4G27680, AT4G27470, AT4G27480, AT4G27500, AT4G27510, AT4G27440, AT4G27410, AT4G27540, AT4G27450, AT4G27520, AT4G27430, AT4G27435, AT4G27560, AT4G27570, AT4G27590, AT4G27640, AT4G27650, AT4G27660, AT4G27690, AT4G27710, AT4G27730, AT4G27750, AT4G27760, AT4G27585, AT5G54100, AT4G27720, AT4G27620, AT4G27390, AT4G27120, AT5G54810, AT4G27070, AT4G27080, AT4G27130, AT4G27090, AT4G27180, AT4G27140, AT4G27250, AT4G27260, AT4G27270, AT4G27290, AT4G27300, AT4G27370, AT4G27400, AT4G27280, AT4G27380, AT4G27320, AT4G27350, AT4G27230, AT4G26900, AT4G26850, AT4G27060, AT4G26690, AT4G26650, AT4G26710, AT4G26720, AT4G26830, AT4G26870, AT4G26880, AT4G26890, AT4G26980, AT4G27010, AT4G27030, AT4G27040, AT4G27050, AT4G26790, AT4G26760, AT4G26940, AT4G26780, AT4G26910, AT4G26860, AT4G26840, AT4G26750, AT4G26970, AT4G27000, AT4G26700, AT4G26320, AT4G26400, AT4G26410, AT4G26460, AT4G26470, AT4G26480, AT4G26490, AT4G26550, AT4G26560, AT4G26430, AT4G26510, AT4G26500, AT4G26555, AT4G26600, AT4G26610, AT4G26630, AT4G26620, AT4G26670, AT4G26570, AT4G26640, AT4G26220, AT4G26210, AT4G26270, AT4G26310, AT4G26130, AT4G26160, AT4G26000, AT4G26060, AT4G26070, AT4G26080, AT4G26100, AT4G26110, AT4G26090, AT4G26010, AT4G26200, AT4G26300, AT1G60720, AT4G26360, AT4G26380, AT4G26240, AT4G26370, AT4G26230, AT4G26120, AT4G26140, AT4G26180, AT4G26190, AT4G25700, AT4G25650, AT4G25770, AT4G25790, AT4G25800, AT4G25835, AT4G25850, AT4G25860, AT4G25870, AT4G25890, AT4G25940, AT4G25960, AT4G25970, AT4G25760, AT4G25910, AT4G25840, AT4G25900, AT4G25830, AT4G25810, AT4G25820, AT4G25880, AT4G25680, AT4G25740, AT4G25670, AT4G25672, AT4G25690, AT4G25692, AT4G25270, AT4G25280, AT4G25310, AT4G25300, AT4G25320, AT4G25250, AT4G25170, AT4G25210, AT4G25350, AT4G25360, AT4G25380, AT4G25390, AT4G25480, AT4G25450, AT4G25434, AT4G25370, AT4G25500, AT4G25490, AT4G25470, AT4G25340, AT4G25640, AT4G25710, AT4G25730, AT4G25630, AT4G25660, AT4G25720, AT4G24920, AT4G24940, AT4G24960, AT4G24820, AT4G24840, AT4G24880, AT4G24800, AT4G25070, AT4G25090, AT4G25110, AT4G25120, AT4G25150, AT4G25160, AT4G25100, AT4G25130, AT4G25050, AT4G25030, AT4G25080, AT4G24990, AT4G25230, AT4G25240, AT4G25260, AT4G24890, AT4G24970, AT4G24900, AT4G24730, AT4G24750, AT4G24790, AT4G24780, AT4G24570, AT4G24760, AT4G24510, AT4G24500, AT4G24670, AT4G24770, AT4G24520, AT4G24560, AT4G24740, AT4G24810, AT4G24830, AT4G24860, AT4G24930, AT4G24610, AT4G24620, AT4G24680, AT4G24690, AT4G24260, AT4G24270, AT4G24280, AT4G24390, AT4G24450, AT4G24460, AT4G24480, AT4G24490, AT4G24220, AT4G24380, AT4G24240, AT4G24210, AT4G24370, AT4G24440, AT4G24340, AT4G24350, AT4G24470, AT4G24330, AT4G24190, AT4G24400, AT4G24250, AT4G24290, AT4G24430, AT4G24530, AT4G24550, AT4G24120, AT4G24050, AT4G24200, AT4G23860, AT4G23880, AT4G23910, AT4G23940, AT4G23950, AT4G23890, AT4G23920, AT4G24000, AT4G23885, AT4G23900, AT4G23850, AT4G23870, AT4G23980, AT4G24020, AT4G24040, AT4G24090, AT4G24110, AT4G24130, AT4G24140, AT4G24160, AT4G24170, AT4G24180, AT4G24060, AT4G24175, AT4G23490, AT4G23450, AT4G23570, AT4G23640, AT4G23700, AT4G23710, AT4G23740, AT4G23820, AT4G23730, AT4G23650, AT4G23670, AT4G23690, AT4G23630, AT4G23620, AT4G23610, AT4G23660, AT4G23810, AT4G23800, AT4G23680, AT4G23750, AT4G23840, AT4G23540, AT4G23400, AT4G23496, AT4G23190, AT4G23210, AT4G23260, AT4G23270, AT4G23280, AT4G23330, AT4G23180, AT4G23350, AT4G23360, AT4G23370, AT4G23410, AT4G23430, AT4G23420, AT4G23440, AT4G23460, AT4G23470, AT4G23493, AT4G23510, AT4G22930, AT4G23040, AT4G23050, AT4G23100, AT4G22850, AT4G22720, AT4G22770, AT4G22670, AT4G22820, AT4G22680, AT4G22740, AT4G22750, AT4G22756, AT4G22840, AT4G22910, AT4G22940, AT4G22950, AT4G22960, AT4G22970, AT4G22980, AT4G23000, AT4G23010, AT4G23060, AT4G23070, AT4G23090, AT4G22890, AT4G22920, AT4G22780, AT4G22790, AT4G22270, AT4G22330, AT4G22410, AT4G22450, AT1G61510, AT4G22460, AT4G22470, AT4G22530, AT4G22560, AT4G22580, AT4G22590, AT4G22592, AT4G22610, AT4G22490, AT4G22570, AT4G22540, AT4G22690, AT4G22710, AT4G22730, AT4G22753, AT4G22260, AT4G22080, AT4G22090, AT4G22120, AT4G22130, AT4G22140, AT4G21990, AT4G22110, AT4G22000, AT4G21980, AT4G22070, AT4G22150, AT4G22250, AT4G22280, AT4G22300, AT4G22320, AT4G22285, AT4G22350, AT4G22380, AT4G22190, AT4G22220, AT4G22310, AT4G22360, AT4G22340, AT4G22212, AT4G22010, AT4G21700, AT4G21710, AT4G21720, AT4G21750, AT4G21760, AT4G21620, AT4G21730, AT4G21780, AT4G21800, AT4G21790, AT4G21650, AT4G21810, AT4G21820, AT4G21890, AT4G21870, AT4G21830, AT4G21840, AT4G21960, AT4G21850, AT4G21860, AT4G21903, AT4G21910, AT4G21940, AT4G21590, AT4G21600, AT4G21580, AT4G21680, AT4G21690, AT4G21280, AT4G21110, AT4G21270, AT4G21300, AT4G21310, AT4G21323, AT4G21340, AT4G21350, AT4G21390, AT4G21400, AT4G21410, AT4G21430, AT4G21440, AT4G21326, AT4G21380, AT4G21470, AT4G21480, AT4G21560, AT4G21610, AT4G21510, AT4G21450, AT4G21190, AT4G21160, AT4G21105, AT4G20820, AT4G20840, AT4G20830, AT4G20850, AT4G20880, AT4G20900, AT3G06433, AT4G20980, AT4G20990, AT4G21000, AT4G21060, AT4G21070, AT4G20960, AT4G20930, AT4G20890, AT4G20860, AT4G20870, AT4G20910, AT4G21080, AT4G21090, AT4G21100, AT4G21120, AT4G21140, AT4G21150, AT4G21170, AT4G21180, AT4G21230, AT4G20150, AT4G20280, AT4G20400, AT4G20420, AT4G20430, AT4G20450, AT4G20460, AT4G20650, AT4G20570, AT4G20630, AT4G20530, AT4G20560, AT4G20640, AT4G20540, AT4G20520, AT4G20620, AT4G20590, AT4G20610, AT4G20580, AT4G20700, AT4G20550, AT4G20600, AT4G20390, AT4G20440, AT4G20410, AT4G20380, AT4G20330, AT4G20350, AT4G20360, AT4G20480, AT4G20740, AT4G20780, AT4G20320, AT4G20300, AT4G20260, AT4G20020, AT4G20050, AT4G20060, AT4G20070, AT4G20090, AT4G20100, AT4G20110, AT4G20130, AT4G20010, AT4G20030, AT4G19985, AT4G20140, AT4G20170, AT4G20210, AT4G20230, AT4G20235, AT4G20250, AT4G20270, AT4G20310, AT4G19960, AT4G19980, AT4G20000, AT4G19600, AT4G19610, AT4G19540, AT4G19640, AT4G19660, AT4G19670, AT4G19680, AT4G19710, AT4G19720, AT4G19790, AT4G17450, AT4G19810, AT4G19820, AT4G19830, AT4G19860, AT4G19865, AT4G19880, AT4G19690, AT4G19840, AT4G19220, AT4G19140, AT4G19185, AT4G19200, AT4G19110, AT4G19112, AT4G19160, AT4G19230, AT4G19120, AT4G19130, AT4G19320, AT5G36020, AT4G19370, AT4G19420, AT4G19430, AT4G19460, AT4G19350, AT4G19450, AT4G19410, AT4G19400, AT4G19390, AT4G19440, AT4G19500, AT4G19510, AT4G18840, AT4G18880, AT4G18900, AT4G18905, AT4G18930, AT4G18975, AT4G19010, AT4G19040, AT4G19045, AT4G19070, AT4G18950, AT4G19030, AT4G18910, AT4G18890, AT4G19100, AT4G19150, AT4G19191, AT4G19210, AT4G18710, AT5G45775, AT4G18730, AT4G18780, AT4G18590, AT4G18600, AT4G18480, AT4G18580, AT4G18470, AT4G18400, AT4G18395, AT4G18593, AT4G18425, AT4G18360, AT4G18610, AT4G18630, AT4G18640, AT4G18650, AT4G18670, AT4G18700, AT4G18740, AT4G18750, AT4G18800, AT4G18820, AT4G18510, AT4G18520, AT4G18530, AT4G18550, AT4G18570, AT4G18160, AT4G18170, AT4G18040, AT5G46430, AT4G18100, AT4G18070, AT4G18210, AT4G18220, AT4G18230, AT4G18240, AT4G18250, AT4G18260, AT4G18270, AT4G18300, AT4G18340, AT4G18280, AT4G18370, AT4G18390, AT4G18410, AT4G18430, AT4G18450, AT4G18465, AT4G18460, AT4G18490, AT4G18130, AT4G18140, AT4G13640, AT4G13800, AT4G13850, AT4G13720, AT4G13770, AT4G13830, AT4G13710, AT4G17830, AT4G17840, AT4G17880, AT4G17905, AT4G17970, AT4G17980, AT4G18030, AT4G17940, AT4G17960, AT4G18020, AT4G17870, AT4G17950, AT4G18010, AT4G17895, AT4G17890, AT4G18050, AT4G18060, AT4G18090, AT4G13550, AT4G13570, AT4G13580, AT4G13600, AT4G13610, AT4G13520, AT4G13530, AT4G13510, AT4G13630, AT4G13650, AT4G13660, AT4G13670, AT4G13730, AT4G13750, AT4G13780, AT4G13820, AT4G13930, AT4G13890, AT4G13900, AT4G13920, AT4G13430, AT4G13420, AT4G13345, AT4G13460, AT4G12980, AT4G13020, AT4G13120, AT4G13130, AT4G13150, AT4G13160, AT4G13190, AT4G13210, AT4G13220, AT4G13260, AT4G13180, AT4G13200, AT4G13195, AT4G13170, AT4G13250, AT4G13270, AT4G13310, AT4G13290, AT4G13330, AT4G13340, AT4G13380, AT4G13440, AT4G13360, AT4G12750, AT4G12760, AT4G12770, AT4G12780, AT4G12840, AT4G12720, AT4G12730, AT4G12690, AT4G12790, AT4G12880, AT4G12800, AT4G12910, AT4G12920, AT4G12990, AT4G13000, AT4G13030, AT4G13050, AT4G13070, AT4G13080, AT4G13100, AT4G13010, AT4G12480, AT4G12430, AT4G12432, AT4G12700, AT4G12740, AT4G12390, AT4G12230, AT4G12250, AT4G12350, AT4G12340, AT4G12420, AT4G12440, AT4G12460, AT4G12470, AT4G12500, AT4G12510, AT4G12520, AT4G12570, AT4G12580, AT4G12620, AT4G14700, AT4G12640, AT4G12650, AT4G12550, AT4G12590, AT4G12600, AT4G12490, AT4G12300, AT4G12320, AT4G12310, AT4G12330, AT4G12400, AT4G11950, AT4G11860, AT4G11790, AT4G11820, AT4G11830, AT4G11850, AT4G11960, AT4G11970, AT4G12000, AT4G12010, AT4G12020, AT4G12080, AT4G12130, AT4G12140, AT4G12150, AT4G12120, AT4G12070, AT4G12060, AT4G12110, AT4G12040, AT4G12030, AT4G11980, AT4G12220, AT4G12240, AT4G11890, AT4G11920, AT4G11560, AT4G11420, AT4G11570, AT4G11590, AT4G11610, AT4G11640, AT4G11660, AT4G11670, AT4G11780, AT4G11650, AT4G11600, AT4G11740, AT4G11800, AT4G11840, AT4G11880, AT4G11470, AT4G11480, AT4G11490, AT4G11530, AT4G11550, AT4G11150, AT4G11160, AT4G11170, AT4G11190, AT4G11200, AT4G11210, AT4G11175, AT4G11230, AT4G11270, AT4G11290, AT4G11320, AT4G11310, AT4G11340, AT4G11350, AT4G11260, AT4G11360, AT4G11220, AT4G11300, AT4G11370, AT4G11240, AT4G11330, AT4G11380, AT4G11280, AT4G11410, AT4G11440, AT4G11450, AT4G11120, AT4G11130, AT4G11140, AT4G10790, AT4G10750, AT4G10770, AT4G10800, AT4G10890, AT4G10930, AT4G10810, AT4G10840, AT4G10960, AT4G10955, AT4G10925, AT4G10920, AT4G10970, AT4G11010, AT4G11030, AT4G11050, AT4G11080, AT4G11090, AT4G11110, AT4G10420, AT4G10360, AT4G10340, AT4G10380, AT4G10460, AT4G10490, AT4G10500, AT4G10510, AT4G10550, AT4G10450, AT4G10480, AT4G10470, AT4G10590, AT4G10570, AT1G42605, AT4G10580, AT4G10640, AT4G10700, AT4G10660, AT4G10610, AT4G10620, AT4G10710, AT4G10730, AT4G10760, AT4G10370, AT4G10390, AT4G10400, AT4G09920, AT4G09760, AT4G09830, AT4G09800, AT4G09950, AT4G09970, AT4G09990, AT4G10060, AT4G10070, AT4G10050, AT4G10040, AT4G10030, AT4G10000, AT4G09980, AT4G10120, AT4G10100, AT4G10090, AT4G10210, AT4G10330, AT4G10350, AT4G09900, AT4G09890, AT4G09470, AT4G09490, AT4G09500, AT4G09520, AT4G09540, AT4G09550, AT4G09560, AT4G09460, AT4G09510, AT4G09570, AT4G09580, AT4G09620, AT4G09630, AT4G09640, AT4G09680, AT4G09650, AT4G09670, AT4G09710, AT4G09720, AT4G09730, AT4G09740, AT4G23560, AT4G09810, AT4G09840, AT3G33377, AT4G04393, AT2G14320, AT3G45340, AT3G34299, AT4G09410, AT4G09420, AT4G08930, AT4G08940, AT4G08950, AT4G08870, AT4G08900, AT4G08980, AT4G08960, AT4G08920, AT4G09020, AT4G09100, AT4G09110, AT4G09150, AT4G09000, AT1G35160, AT4G09030, AT4G09140, AT4G09300, AT4G09340, AT4G09320, AT4G08580, AT5G17900, AT4G08590, AT4G08500, AT4G08690, AT4G08685, AT4G08620, AT4G08730, AT4G08770, AT4G08780, AT4G08840, AT4G08850, AT5G41160, AT4G08700, AT4G08790, AT4G08890, AT4G08480, AT4G08530, AT4G08250, AT4G08300, AT4G08310, AT4G08290, AT4G08240, AT4G08280, AT4G08170, AT4G08320, AT4G08330, AT4G08360, AT4G08350, AT4G08410, AT4G08390, AT4G08460, AT4G08470, AT4G08150, AT4G08160, AT4G08180, AT4G08220, AT4G07790, AT4G07810, AT4G07820, AT4G07920, AT4G07990, AT4G08000, AT4G07960, AT4G07950, AT1G01210, AT4G08110, AT4G07730, AT4G07750, AT4G07360, AT4G07400, AT4G07410, AT4G07450, AT4G07390, AT4G07540, AT4G07586, AT4G07600, AT5G19740, AT4G07670, AT3G30465, AT4G07680, AT2G07395, AT4G05320, AT4G05150, AT4G05370, AT4G05400, AT4G05410, AT4G05420, AT4G05440, AT4G05390, AT4G05460, AT4G05330, AT4G05450, AT4G05520, AT4G05550, AT4G05580, AT4G05530, AT4G05590, AT4G05620, AT4G05630, AT4G05180, AT4G05090, AT4G05100, AT4G04990, AT4G05000, AT4G05070, AT4G05050, AT4G05060, AT4G05020, AT4G05040, AT4G05120, AT4G05110, AT4G05160, AT4G05170, AT4G05190, AT4G05200, AT4G05210, AT4G05250, AT4G05260, AT4G04930, AT4G04890, AT4G04940, AT4G04950, AT4G04960, AT4G04970, AT4G04600, AT4G04620, AT4G04610, AT4G04630, AT4G04670, AT4G04640, AT4G04710, AT4G04720, AT4G04750, AT4G04790, AT4G04840, AT4G04880, AT4G04870, AT4G04800, AT4G04830, AT4G04860, AT4G04850, AT4G04770, AT4G04740, AT4G04910, AT4G04920, AT4G04955, AT4G04170, AT4G04180, AT4G04190, AT4G04220, AT4G04230, AT4G04290, AT2G07420, AT4G04210, AT4G04320, AT4G04350, AT4G04370, AT4G04340, AT4G04440, AT4G04450, AT4G04470, AT4G04500, AT4G04570, AT4G04540, AT4G04460, AT4G03820, AT4G03830, AT4G03890, AT2G10140, AT3G30680, AT4G03900, AT1G40123, AT4G03950, AT4G03960, AT2G06430, AT2G04980, AT2G02210, AT4G22240, AT4G04020, AT4G04040, AT4G04110, AT4G03790, AT4G03500, AT4G03550, AT4G03520, AT4G03560, AT4G03510, AT4G03430, AT4G03610, AT4G03700, AT4G03740, AT4G03600, AT4G03400, AT4G03460, AT4G03440, AT4G03090, AT4G03100, AT4G03110, AT4G02980, AT4G03140, AT4G03160, AT4G03190, AT4G03200, AT4G03230, AT4G03240, AT4G03260, AT4G03270, AT4G03340, AT4G03390, AT4G03410, AT4G03320, AT4G03120, AT4G03210, AT4G03180, AT4G03280, AT4G03150, AT4G03060, AT4G03070, AT4G03080, AT4G02530, AT4G02570, AT4G02580, AT4G02700, AT4G02560, AT4G02750, AT4G02790, AT4G02810, AT4G02820, AT4G02850, AT4G02860, AT4G02880, AT4G02900, AT4G02930, AT4G02920, AT4G02770, AT4G02840, AT4G02890, AT4G02800, AT4G02940, AT4G02990, AT4G03020, AT4G03030, AT4G02430, AT4G02450, AT4G02480, AT4G02370, AT4G02440, AT4G02380, AT4G02485, AT4G02460, AT4G02510, AT4G02500, AT4G02540, AT4G02550, AT4G02600, AT4G02610, AT4G02630, AT4G02660, AT4G02680, AT4G02690, AT4G02710, AT4G02720, AT4G02640, AT4G02590, AT4G02620, AT4G02730, AT4G02390, AT4G02400, AT4G02410, AT4G02200, AT4G02080, AT4G02130, AT4G02150, AT4G02220, AT4G02210, AT4G02020, AT4G02075, AT4G02195, AT4G02060, AT4G02270, AT4G02290, AT4G02230, AT4G02280, AT4G02260, AT4G02330, AT4G02340, AT4G02350, AT4G02360, AT4G02090, AT4G02120, AT4G02160, AT4G02170, AT4G02180, AT4G01790, AT4G01575, AT4G01690, AT4G01680, AT4G01710, AT4G01800, AT4G01860, AT4G01870, AT4G01880, AT4G01910, AT4G01920, AT4G01930, AT4G01950, AT4G01970, AT4G01840, AT4G01850, AT4G01960, AT4G01897, AT4G01810, AT4G01940, AT4G01990, AT4G01900, AT4G02010, AT4G02030, AT4G02050, AT4G02070, AT4G01740, AT4G01770, AT4G01750, AT4G01760, AT4G01780, AT3G48670, AT4G01150, AT4G01250, AT4G01320, AT4G01100, AT4G01220, AT4G01050, AT4G01400, AT4G01430, AT4G01440, AT4G01450, AT4G01470, AT4G01500, AT4G01510, AT4G01530, AT4G01550, AT4G01560, AT4G01480, AT4G01570, AT4G35680, AT4G01590, AT4G01610, AT4G01640, AT2G34280, AT4G01650, AT4G01660, AT4G01700, AT4G01720, AT4G01730, AT4G00830, AT4G01010, AT4G01020, AT5G10370, AT4G01026, AT4G01040, AT4G01080, AT4G01090, AT4G01110, AT4G01140, AT4G01190, AT4G01210, AT4G01240, AT4G01270, AT4G01280, AT4G01290, AT4G01330, AT4G01350, AT5G59920, AT4G01380, AT4G01390, AT4G01070, AT4G01310, AT4G01370, AT4G01120, AT4G00780, AT4G00955, AT4G00850, AT4G00860, AT4G00700, AT4G00710, AT4G00680, AT4G00585, AT4G00650, AT4G00720, AT4G00730, AT4G00750, AT4G00740, AT4G00760, AT4G00800, AT4G00820, AT4G00880, AT4G00900, AT4G00910, AT5G37190, AT4G00930, AT4G00940, AT4G00950, AT4G00960, AT4G00990, AT4G00810, AT4G00770, AT4G00895, AT4G00755, AT4G00690, AT4G00400, AT4G00420, AT4G00440, AT4G00450, AT4G00240, AT4G00238, AT4G00250, AT4G00416, AT4G00335, AT4G00355, AT4G00340, AT4G00430, AT4G00480, AT4G00490, AT4G00500, AT4G00520, AT4G00530, AT4G00550, AT4G00560, AT4G00570, AT4G00590, AT4G00600, AT4G00620, AT4G00630, AT4G00660, AT4G00370, AT5G01630, AT4G00020, AT4G00050, AT4G00080, AT4G00110, AT4G00150, AT4G00200, AT4G00230, AT4G00040, AT4G00170, AT4G00100, AT4G00231, AT4G00030, AT4G00090, AT4G00290, AT4G00234, AT4G00300, AT4G00320, AT5G41830, AT4G00330, AT1G32070, AT1G32060, AT1G31940, AT3G29575, AT1G25540, AT1G25530, AT1G25510, AT1G25500, AT1G25472, AT1G25470, AT1G25460, AT1G25490, AT1G25450, AT1G25400, AT1G25550, AT1G25520, AT1G25380, AT1G25420, AT1G25570, AT1G25580, AT1G25390, AT1G25560, AT1G25375, AT1G32020, AT1G31970, AT1G31950, AT1G32050, AT1G18570, AT1G19940, AT4G00460, AT1G16800, AT1G16790, AT1G16780, AT1G16710, AT1G16720, AT1G16750, AT1G16740, AT1G18610, AT1G18560, AT1G18590, AT1G18620, AT1G18600, AT1G18540, AT1G18630, AT1G18640, AT1G18650, AT1G18580, AT1G19880, AT1G19850, AT1G19870, AT1G19860, AT1G19920, AT2G33590, AT2G33310, AT2G33370, AT2G33560, AT2G33430, AT2G33620, AT2G33250, AT2G33480, AT2G33380, AT2G33290, AT2G33630, AT2G33255, AT4G10180, AT4G10130, AT4G10170, AT4G10270, AT4G10280, AT4G10300, AT4G10140, AT4G10310, AT1G19900, AT2G33470, AT2G33330, AT2G33570, AT2G40590, AT2G40510, AT2G40570, AT2G40610, AT2G40470, AT2G40530, AT2G40475, AT2G40490, AT2G40600, AT2G40630, AT2G40540, AT2G33340, AT2G33360, AT2G33390, AT2G33410, AT2G33440, AT2G33460, AT2G33520, AT2G33530, AT2G33540, AT2G33580, AT2G33600, AT2G33610, AT2G33640, AT2G33450, AT1G67040, AT1G66970, AT1G67020, AT1G67050, AT1G66950, AT1G66940, AT1G67080, AT1G67030, AT5G34930, AT5G34940, AT2G30300, AT2G30320, AT2G30330, AT2G30350, AT2G30270, AT2G30280, AT2G30260, AT2G30360, AT2G30340, AT2G40550, AT2G40480, AT2G40460, AT2G40620, AT1G67060, AT1G67070, AT1G20280, AT1G20270, AT1G20310, AT1G20330, AT1G20340, AT1G20370, AT1G20300, AT1G17840, AT1G17870, AT1G17960, AT1G17980, AT1G18020, AT1G17990, AT1G17800, AT1G17930, AT1G17970, AT1G17830, AT1G17890, AT1G17880, AT1G17950, AT1G17860, AT1G17790, AT1G17920, AT1G18010, AT1G18000, AT1G18040, AT1G18030, AT1G66930, AT1G22240, AT1G22210, AT1G22180, AT1G22170, AT1G22200, AT1G22190, AT1G12640, AT1G12650, AT1G12710, AT1G12750, AT1G12740, AT1G12700, AT1G12680, AT1G12620, AT1G12730, AT1G20380, AT1G22360, AT5G28610, AT1G22020, AT1G22040, AT1G22060, AT1G22110, AT1G22050, AT1G22070, AT1G22030, AT1G22160, AT1G21980, AT1G22140, AT1G22330, AT1G22310, AT1G22290, AT1G22300, AT1G22280, AT1G22270, AT3G32400, AT1G34030, AT1G34010, AT1G33980, AT1G33970, AT1G34020, AT1G34000, AT1G33990, AT1G34120, AT1G34150, AT1G29820, AT1G29790, AT1G29760, AT1G29750, AT1G29910, AT1G29920, AT1G29930, AT1G29780, AT1G29860, AT1G29880, AT1G29850, AT1G29900, AT1G34065, AT1G34040, AT1G34060, AT1G19220, AT1G19230, AT1G19250, AT1G19270, AT1G19200, AT1G19240, AT1G19180, AT1G58300, AT1G58290, AT1G58270, AT1G58360, AT1G58340, AT1G58370, AT1G34160, AT1G34130, AT1G34110, AT1G07250, AT1G07080, AT1G07140, AT1G07110, AT1G19160, AT1G19170, AT1G19190, AT1G07270, AT1G07230, AT1G07220, AT1G07210, AT1G07150, AT1G07010, AT1G07000, AT1G06960, AT1G07260, AT1G07030, AT1G07240, AT1G07040, AT1G07090, AT1G07020, AT1G07070, AT1G07170, AT1G13690, AT1G13740, AT1G13730, AT1G13640, AT1G18080, AT1G18100, AT1G18260, AT1G18150, AT1G18060, AT1G18090, AT1G20670, AT1G20690, AT1G20700, AT1G20720, AT1G20760, AT1G20770, AT1G20800, AT1G20830, AT1G20693, AT1G20696, AT1G20823, AT1G20780, AT1G13670, AT1G13700, AT1G13710, AT1G13750, AT1G13540, AT1G16910, AT1G16870, AT1G16940, AT1G16900, AT1G16820, AT1G16920, AT1G16810, AT1G16850, AT1G16880, AT1G16858, AT1G16860, AT1G16840, AT1G16970, AT1G18070, AT1G18160, AT1G18180, AT1G18190, AT1G18250, AT1G18140, AT1G18210, AT1G18170, AT1G13600, AT1G13610, AT1G13570, AT1G13560, AT1G48690, AT1G48650, AT1G48640, AT1G48790, AT1G48830, AT1G48630, AT1G48750, AT1G48760, AT1G55080, AT1G55090, AT1G55110, AT1G55120, AT1G55130, AT1G55150, AT3G08510, AT1G13580, AT1G13590, AT1G30135, AT1G30120, AT1G30090, AT1G55390, AT1G48780, AT1G51800, AT1G51790, AT1G51730, AT1G51720, AT1G51640, AT1G51620, AT1G51760, AT1G51780, AT1G51710, AT1G51820, AT1G51740, AT1G51660, AT1G51650, AT1G51700, AT1G51680, AT1G51690, AT1G30130, AT1G30110, AT1G30200, AT1G58220, AT1G58250, AT1G58230, AT1G50840, AT1G50890, AT1G50910, AT1G50920, AT1G50930, AT1G50940, AT1G50950, AT1G50970, AT1G50990, AT1G50900, AT1G56340, AT1G56320, AT1G56270, AT1G56260, AT1G56230, AT1G56300, AT1G56210, AT1G56200, AT1G56330, AT1G56220, AT1G56280, AT1G56290, AT1G56190, AT1G58200, AT3G12340, AT3G12630, AT3G12570, AT3G12360, AT3G12370, AT3G12490, AT3G12350, AT3G12610, AT3G12400, AT3G12600, AT3G12390, AT3G12380, AT3G12500, AT3G12520, AT3G12580, AT3G66658, AT3G66652, AT3G11270, AT3G11320, AT3G11330, AT3G11340, AT3G11250, AT3G11290, AT3G11280, AT3G11230, AT3G11240, AT3G12480, AT3G12440, AT3G12150, AT3G12290, AT3G12210, AT3G12320, AT3G12260, AT3G12280, AT3G12250, AT3G12300, AT3G12140, AT3G12100, AT3G12090, AT3G12080, AT3G12110, AT3G12070, AT3G12120, AT3G12130, AT3G12620, AT3G12560, AT3G12550, AT3G12540, AT3G12530, AT3G12510, AT3G12180, AT3G12270, AT3G12240, AT3G12230, AT3G12200, AT1G69440, AT1G69450, AT1G69480, AT1G69530, AT1G69490, AT1G69510, AT1G69526, AT1G69550, AT1G30400, AT1G30370, AT1G30350, AT1G30420, AT1G30410, AT1G30380, AT1G30360, AT1G30330, AT1G35880, AT2G14470, AT5G34960, AT1G35940, AT1G35910, AT3G12170, AT1G55020, AT1G54990, AT1G54920, AT1G66760, AT3G02340, AT3G02350, AT3G02360, AT1G76850, AT1G76870, AT1G76880, AT1G76890, AT1G76860, AT1G72125, AT1G72030, AT1G72060, AT1G72070, AT1G72040, AT1G72020, AT1G72090, AT1G72050, AT1G55030, AT1G54890, AT1G54940, AT1G54960, AT1G54970, AT1G55000, AT5G43620, AT1G66500, AT1G66470, AT1G66440, AT1G66520, AT1G66450, AT1G66510, AT1G66530, AT1G66880, AT1G66810, AT1G66800, AT1G66780, AT1G66770, AT1G66750, AT1G66740, AT1G66730, AT1G66690, AT1G66700, AT1G66650, AT1G66680, AT1G66860, AT1G66820, AT1G66660, AT1G66580, AT1G66540, AT3G06170, AT3G06180, AT3G06090, AT3G06280, AT3G06290, AT3G06150, AT3G06110, AT3G06240, AT3G06140, AT3G06200, AT3G06130, AT3G06190, AT3G06250, AT1G50700, AT1G66620, AT1G66670, AT3G11220, AT3G11100, AT3G11200, AT3G11210, AT3G11050, AT3G11170, AT5G05580, AT1G33500, AT1G33520, AT1G33540, AT1G33490, AT1G33560, AT1G33420, AT3G11080, AT3G11040, AT3G11020, AT3G11010, AT5G27060, AT3G10985, AT3G11180, AT3G08530, AT3G11130, AT2G40205, AT3G11120, AT3G56020, AT3G08520, AT3G10920, AT3G10970, AT3G10940, AT3G10930, AT3G10960, AT1G33440, AT5G04781, AT1G75170, AT1G75270, AT1G75280, AT1G75190, AT1G75180, AT1G75230, AT1G75220, AT1G36180, AT1G36240, AT1G36280, AT4G18440, AT1G32580, AT1G32550, AT1G32540, AT1G42580, AT1G69710, AT1G42680, AT1G33400, AT1G33410, AT1G33430, AT1G31420, AT1G31410, AT1G31430, AT1G31440, AT1G31540, AT1G31470, AT1G31460, AT1G31500, AT1G31600, AT1G31580, AT1G36640, AT1G36620, AT1G36730, AT1G36732, AT1G75210, AT1G75200, AT1G75150, AT1G75140, AT1G33360, AT1G33390, AT1G33265, AT1G31480, AT1G66020, AT1G66150, AT1G66080, AT1G66160, AT1G66070, AT1G66200, AT1G66180, AT1G66090, AT1G66140, AT1G33260, AT1G33290, AT1G33320, AT1G33350, AT1G33250, AT1G33270, AT1G33280, AT1G33330, AT1G33340, AT3G32900, AT1G42540, AT1G42550, AT1G42480, AT3G14800, AT3G14840, AT3G14770, AT3G31320, AT3G31360, AT3G31300, AT3G31415, AT3G32030, AT3G29780, AT3G29770, AT3G19553, AT3G19520, AT3G19540, AT3G30730, AT3G30740, AT3G14860, AT3G14780, AT3G14790, AT3G28210, AT3G28200, AT3G28720, AT3G28850, AT3G28760, AT3G28740, AT3G28730, AT3G28510, AT1G36770, AT3G28530, AT3G28540, AT3G28500, AT3G30180, AT3G14850, AT3G29640, AT3G32940, AT3G32930, AT3G29270, AT3G29280, AT3G29290, AT3G29300, AT3G22250, AT3G22260, AT3G22240, AT3G24440, AT3G24480, AT3G24450, AT3G19960, AT3G19990, AT3G20010, AT3G20015, AT3G19970, AT3G20000, AT3G19980, AT3G28230, AT3G28330, AT3G28340, AT3G12030, AT3G19090, AT3G32260, AT3G32270, AT3G24250, AT3G13590, AT3G13610, AT3G13580, AT3G13570, AT3G13620, AT3G18850, AT3G18880, AT3G18890, AT3G18860, AT3G12010, AT3G12012, AT3G12020, AT3G11964, AT3G12050, AT3G12040, AT3G20700, AT3G20670, AT3G52290, AT3G52370, AT3G52360, AT3G52170, AT3G52180, AT3G52190, AT3G52380, AT3G52300, AT3G52230, AT3G52340, AT3G52200, AT3G52220, AT3G32040, AT3G32120, AT3G32140, AT3G20650, AT3G20660, AT3G20680, AT3G52260, AT3G30380, AT3G30260, AT3G30300, AT3G30320, AT3G30350, AT3G30390, AT2G34020, AT2G34060, AT2G34080, AT2G34140, AT2G34150, AT2G34050, AT2G34160, AT2G34040, AT2G34070, AT3G52240, AT3G25660, AT3G25680, AT3G30340, AT3G29390, AT3G29400, AT3G29410, AT3G29430, AT3G29460, AT2G11180, AT3G29375, AT3G29370, AT3G29350, AT3G29360, AT3G29320, AT3G29180, AT3G27150, AT3G27170, AT3G27160, AT3G25690, AT3G25600, AT3G25610, AT3G25620, AT3G25640, AT3G25655, AT3G16860, AT3G29310, AT3G22200, AT3G22231, AT3G13682, AT3G13690, AT3G13710, AT3G13700, AT3G13750, AT3G13760, AT3G13772, AT3G13780, AT3G13782, AT3G13784, AT3G13650, AT3G13670, AT3G13674, AT3G13720, AT3G13790, AT3G13730, AT3G13672, AT3G16857, AT3G22160, AT3G22230, AT3G22110, AT3G22210, AT3G18600, AT3G18630, AT3G18560, AT3G20900, AT3G20950, AT3G20960, AT3G20920, AT3G20930, AT3G20910, AT3G20970, AT3G21430, AT3G21340, AT3G21350, AT3G21360, AT3G21400, AT3G21420, AT3G21390, AT3G22170, AT3G22180, AT3G22190, AT3G22220, AT3G22120, AT3G22968, AT3G22970, AT3G22850, AT3G22870, AT3G22880, AT3G22910, AT3G22890, AT3G22960, AT3G22900, AT3G22950, AT3G22930, AT3G31970, AT3G27950, AT3G27960, AT3G27930, AT3G18640, AT3G15180, AT3G15190, AT3G15140, AT3G23840, AT3G23920, AT3G23940, AT3G24070, AT3G23890, AT3G23820, AT3G26380, AT3G26400, AT3G26420, AT3G26430, AT3G26300, AT3G26480, AT3G26490, AT3G26330, AT3G26470, AT3G26460, AT3G26370, AT3G26450, AT3G26410, AT3G26440, AT3G15120, AT3G15130, AT3G15150, AT3G15160, AT3G24010, AT3G23830, AT3G19030, AT3G19000, AT3G24660, AT3G24670, AT3G23910, AT3G24255, AT3G23960, AT3G23980, AT3G23990, AT3G24030, AT3G24040, AT3G24080, AT3G24090, AT3G23870, AT3G23880, AT3G23900, AT3G24050, AT3G18930, AT4G01580, AT3G18960, AT3G18970, AT3G18980, AT3G18940, AT3G19010, AT3G29635, AT3G22540, AT3G22550, AT3G22590, AT3G22460, AT3G22520, AT3G22600, AT3G22530, AT3G22570, AT3G22480, AT3G22620, AT3G22630, AT3G30720, AT3G23290, AT3G18910, AT3G18990, AT3G19040, AT3G19080, AT3G21160, AT3G29630, AT3G13550, AT3G13410, AT3G13430, AT3G13440, AT3G13510, AT3G13445, AT3G13560, AT3G13490, AT3G13520, AT3G13450, AT3G13400, AT3G21060, AT3G21070, AT3G21090, AT3G21140, AT3G20980, AT3G21110, AT3G21055, AT3G13380, AT3G13460, AT3G13470, AT3G13480, AT3G28700, AT3G28715, AT3G28710, AT3G28580, AT3G28670, AT3G25830, AT3G25820, AT3G25840, AT3G14067, AT3G14180, AT3G14200, AT3G14120, AT3G14080, AT3G14100, AT3G14110, AT3G14130, AT3G14150, AT3G14190, AT3G14075, AT3G14205, AT3G14210, AT3G14160, AT3G14090, AT3G26922, AT3G26930, AT3G28680, AT3G28690, AT3G19630, AT3G19640, AT3G19590, AT3G19710, AT3G19580, AT3G19760, AT3G19100, AT3G19190, AT3G19200, AT3G19230, AT3G19240, AT3G19280, AT3G19140, AT3G19170, AT3G19120, AT3G19130, AT3G19260, AT3G19160, AT3G19220, AT3G28640, AT3G28660, AT3G19720, AT3G19740, AT3G19570, AT3G15260, AT3G15290, AT3G15210, AT3G15353, AT3G15351, AT3G15350, AT3G15310, AT3G15352, AT3G15280, AT3G18230, AT3G18250, AT3G18290, AT3G18240, AT4G21460, AT3G18260, AT3G18280, AT3G18270, AT3G28170, AT3G28180, AT3G14220, AT3G19650, AT3G19660, AT3G19670, AT3G19680, AT3G15220, AT3G15240, AT3G20590, AT3G20600, AT3G20620, AT3G20630, AT3G20490, AT3G20500, AT3G20510, AT3G20520, AT3G20550, AT3G20570, AT3G20480, AT3G20560, AT3G25010, AT3G25050, AT3G25040, AT3G15300, AT3G29070, AT3G30450, AT3G20120, AT3G20130, AT3G20140, AT3G20150, AT3G20020, AT3G20160, AT3G20250, AT3G20040, AT3G20050, AT3G20060, AT3G20070, AT3G20080, AT3G20100, AT3G20240, AT3G20230, AT3G12870, AT3G12900, AT3G28860, AT3G28910, AT3G28900, AT3G20090, AT3G20110, AT3G27300, AT3G27320, AT3G27260, AT3G27270, AT3G27240, AT3G27280, AT3G27230, AT3G27200, AT3G27310, AT3G27220, AT3G27210, AT3G27290, AT3G24350, AT3G24300, AT3G24290, AT3G24310, AT3G24320, AT3G24340, AT3G24430, AT3G24315, AT3G23700, AT3G23810, AT3G27190, AT3G23480, AT3G23470, AT3G23530, AT3G23510, AT3G23490, AT3G13070, AT3G13170, AT3G13190, AT3G13210, AT5G45990, AT3G13090, AT3G13100, AT3G13150, AT3G13222, AT3G13120, AT3G13175, AT3G13200, AT3G13160, AT3G13110, AT3G23790, AT3G23800, AT3G23690, AT3G23710, AT3G23740, AT3G23750, AT3G23730, AT3G23805, AT3G16520, AT3G16530, AT3G14900, AT3G14910, AT3G14920, AT3G15080, AT3G15090, AT3G15020, AT3G15040, AT3G15070, AT3G14990, AT3G14940, AT3G15000, AT3G14930, AT3G27925, AT3G27870, AT3G27850, AT3G27830, AT3G27880, AT3G27820, AT3G27890, AT3G16490, AT3G16540, AT3G16500, AT3G15050, AT3G15060, AT3G15095, AT3G14890, AT3G17430, AT3G24200, AT3G24210, AT3G24230, AT3G24240, AT3G24110, AT3G24140, AT3G24150, AT3G24180, AT3G24120, AT3G24160, AT3G24170, AT3G24190, AT3G22090, AT3G22104, AT3G21890, AT3G22070, AT3G22060, AT3G14980, AT3G15010, AT3G17450, AT3G17440, AT3G28140, AT3G28007, AT3G28130, AT3G14510, AT3G14470, AT3G14430, AT3G14440, AT3G29680, AT3G29720, AT3G29650, AT3G29760, AT3G29765, AT4G09660, AT3G29766, AT3G29763, AT1G19260, AT3G29660, AT3G29670, AT3G15550, AT3G15590, AT3G15580, AT3G17420, AT3G28040, AT3G28155, AT3G28050, AT3G28080, AT3G28120, AT3G28150, AT3G26560, AT3G26570, AT3G26520, AT3G26590, AT3G30775, ATMG01090, AT2G07777, ATMG01100, AT2G07812, ATMG01130, ATMG01180, ATMG01200, AT2G07826, ATMG01210, ATMG01220, AT2G07798, ATMG01240, ATMG01250, AT2G07697, AT2G07813, ATMG01260, ATMG01290, ATMG01350, ATMG01360, ATMG01370, ATMG01400, ATMG01410, ATMG00060, ATMG00513, AT2G07711, ATMG00040, AT2G07671, ATMG01080, AT1G36540, AT3G33066, AT1G41700, AT3G30846, AT2G42140, AT2G33820, AT2G34900, AT2G23050, AT2G41050, AT2G34290, AT2G35550, AT2G47810, AT2G43140, AT2G43280, AT2G38640, AT2G28890, AT2G37950, AT2G40750, AT2G14510, AT2G14440, AT2G17970, AT2G32050, AT2G15630, AT2G01990, AT1G24250, AT1G23810, AT1G23550, AT1G62520, AT1G04050, AT1G24620, AT1G27060, AT1G27100, AT1G24340, AT1G22910, AT1G30850, AT1G77250, AT1G11940, AT1G62010, AT1G54850, AT1G78990, AT2G27940, AT2G36360, AT2G21850, AT2G24590, AT2G24880, AT2G04020, AT2G12550, AT2G04230, AT1G10530, AT1G05690, AT1G65130, AT1G65170, AT1G27870, AT2G16100, AT2G18120, AT1G24040, AT1G75580, AT1G75470, AT1G14820, AT1G20940, AT1G62840, AT1G31320, AT1G53770, AT1G59530, AT1G33840, AT1G80850, AT1G80960, AT1G10660, AT1G16130, AT1G16150, AT1G15640, AT1G08430, AT1G49620, AT1G01650, AT1G63580, AT1G71850, AT1G36950, AT4G00070, AT1G01380, AT1G32375, AT1G07960, AT2G43730, AT2G43740, AT1G51430, AT1G63850, AT5G36180, AT1G73300, AT1G78110, AT1G72530, AT1G52240, AT1G29600, AT1G55360, AT1G35250, AT1G12390, AT1G12340, AT1G13290, AT1G69040, AT3G03880, AT3G01210, AT3G08990, AT3G01330, AT3G09330, AT3G09340, AT3G03040, AT3G04700, AT3G10840, AT3G09670, AT3G09490, AT3G02800, AT3G21520, AT3G25950, AT3G26050, AT3G28960, AT3G20880, AT3G23200, AT3G13000, AT3G26130, AT3G16830, AT3G24890, AT3G24780, AT3G14960, AT3G24270, AT3G30480, AT3G19610, AT3G28610, AT3G23930, AT3G26350, AT3G16870, AT2G33990, AT3G20710, AT3G30160, AT3G06210, AT1G66480, AT1G56310, AT3G24880, AT3G24870, AT3G24900, AT3G24840, AT3G24740, AT3G24750, AT3G24760, AT3G24800, AT3G24830, AT3G24770, AT3G24820, AT3G13800, AT3G13840, AT3G13870, AT3G13880, AT3G13860, AT3G13845, AT3G13810, AT3G26580, AT3G26600, AT3G26610, AT3G26510, AT3G26540, AT3G26550, AT3G24715, AT3G24810, AT3G20410, AT3G20280, AT3G26210, AT3G26220, AT3G26230, AT3G26270, AT3G26290, AT3G26140, AT3G26150, AT3G26125, AT3G26200, AT3G25810, AT3G25760, AT3G25770, AT3G25710, AT3G25730, AT3G25780, AT3G25790, AT3G25740, AT3G25805, AT3G16840, AT3G16800, AT3G16850, AT3G16810, AT3G13225, AT3G13310, AT3G13350, AT3G13228, AT3G13235, AT3G13230, AT3G13226, AT3G13360, AT3G13340, AT3G13320, AT3G20260, AT3G20430, AT3G20270, AT3G20440, AT3G20460, AT3G20340, AT3G20450, AT3G20370, AT3G20390, AT3G20310, AT3G20320, AT3G20290, AT3G20420, AT3G20470, AT3G20330, AT3G13240, AT3G13280, AT3G13290, AT3G13300, AT3G12770, AT3G12800, AT3G12810, AT3G12820, AT3G12830, AT3G12860, AT3G12680, AT3G12690, AT3G12700, AT3G12730, AT3G12780, AT3G12740, AT3G12710, AT3G12670, AT3G12640, AT3G12720, AT3G12760, AT3G12685, AT3G12650, AT3G13330, AT3G27325, AT3G27350, AT3G27430, AT3G27420, AT3G27380, AT3G27360, AT3G12750, AT3G18300, AT3G18390, AT3G18400, AT3G18440, AT3G18450, AT3G18310, AT3G18490, AT3G18500, AT3G18510, AT3G18524, AT3G18380, AT3G18295, AT3G18520, AT3G18420, AT3G18430, AT3G18480, AT3G18350, AT3G18370, AT3G27400, AT3G27470, AT3G27330, AT3G27340, AT3G27390, AT3G29230, AT3G29240, AT3G29200, AT3G18700, AT3G18710, AT3G18780, AT3G18800, AT3G18690, AT3G18740, AT3G18820, AT3G18760, AT3G18680, AT3G18660, AT3G22980, AT3G23070, AT3G23090, AT3G23100, AT3G23120, AT3G23110, AT3G23020, AT3G23030, AT3G23050, AT3G22990, AT3G23000, AT3G23080, AT3G29185, AT3G29250, AT3G29210, AT3G27100, AT3G27120, AT3G26950, AT3G26980, AT3G26990, AT3G27000, AT3G27010, AT3G27020, AT3G27090, AT3G27110, AT3G27080, AT3G27050, AT3G15910, AT3G15940, AT3G15950, AT3G15890, AT3G15900, AT3G18750, AT3G18770, AT3G18790, AT3G18810, AT3G18830, AT3G18670, AT3G26650, AT3G26935, AT3G27060, AT3G27070, AT3G25100, AT3G25110, AT3G25130, AT3G25140, AT3G25150, AT3G25120, AT3G25220, AT3G25230, AT3G25190, AT3G25290, AT3G26700, AT3G26730, AT3G26630, AT3G26670, AT3G26680, AT3G26690, AT3G26710, AT3G26740, AT3G26640, AT3G26720, AT3G25210, AT3G25250, AT3G28400, AT3G28480, AT3G28430, AT3G13020, AT3G13030, AT3G13060, AT3G12940, AT3G12950, AT3G12980, AT3G13040, AT3G12930, AT3G60500, AT3G12990, AT3G12920, AT3G12955, AT3G13062, AT3G13050, AT3G17720, AT3G17760, AT3G17740, AT3G17750, AT3G17770, AT3G17780, AT3G25070, AT3G25160, AT3G17110, AT3G17120, AT3G17130, AT3G17160, AT3G16910, AT3G17180, AT3G16940, AT3G17090, AT3G16990, AT3G17170, AT3G17020, AT3G17000, AT3G17100, AT3G16950, AT3G16920, AT3G18030, AT3G28345, AT3G28420, AT3G28450, AT3G28460, AT3G25520, AT3G25470, AT3G25410, AT3G25480, AT3G25580, AT3G25530, AT3G25500, AT3G25540, AT3G25545, AT3G23210, AT3G23230, AT3G23250, AT3G23260, AT3G23270, AT3G23150, AT3G23160, AT3G23190, AT3G23170, AT3G23280, AT3G17030, AT3G17040, AT3G25585, AT3G25420, AT3G25440, AT3G19810, AT3G19820, AT3G19930, AT3G21840, AT3G21850, AT3G21710, AT3G21720, AT3G21740, AT3G21750, AT3G21700, AT3G21770, AT3G21865, AT3G21760, AT3G21810, AT3G25400, AT3G25490, AT3G25510, AT3G25560, AT3G25570, AT3G25572, AT3G19790, AT3G19840, AT3G19850, AT3G19900, AT3G19910, AT3G27550, AT3G27520, AT3G27570, AT3G27530, AT3G27560, AT3G20720, AT3G20820, AT3G20830, AT3G20840, AT3G20850, AT3G20860, AT3G20730, AT3G20770, AT3G20780, AT3G20790, AT3G20800, AT3G20810, AT3G20740, AT3G20870, AT3G19770, AT3G19860, AT3G19870, AT3G19950, AT3G27540, AT3G28940, AT3G28950, AT3G28930, AT3G19390, AT3G19400, AT3G19420, AT3G19430, AT3G19440, AT3G19300, AT3G19510, AT3G19310, AT3G19320, AT3G19340, AT3G19350, AT3G19370, AT3G19500, AT3G19470, AT3G19490, AT3G19380, AT3G19460, AT3G19450, AT3G19360, AT3G19290, AT3G19515, AT3G27473, AT3G27580, AT3G27480, AT3G27490, AT3G21250, AT3G21300, AT3G21180, AT3G21210, AT3G21230, AT3G21260, AT3G21200, AT3G21190, AT3G21175, AT3G21310, AT3G21290, AT3G21270, AT3G21280, AT3G21220, AT3G21240, AT3G16290, AT3G16220, AT3G16200, AT3G16260, AT3G16190, AT3G16230, AT3G16240, AT3G29010, AT3G28970, AT3G29035, AT3G26030, AT3G25910, AT3G26000, AT3G14620, AT3G14680, AT3G25960, AT3G25970, AT3G25980, AT3G26070, AT3G26080, AT3G26090, AT3G25890, AT3G25900, AT3G25920, AT3G25870, AT3G25940, AT3G25930, AT3G26085, AT3G25905, AT3G25855, AT3G26020, AT3G26100, AT3G25860, AT3G26060, AT3G14740, AT3G14600, AT3G14560, AT3G14595, AT3G14750, AT3G14690, AT3G23610, AT3G23660, AT3G23670, AT3G23540, AT3G23550, AT3G23590, AT3G23600, AT3G23630, AT3G23620, AT3G23605, AT3G23580, AT3G23560, AT3G23570, AT3G23640, AT3G14610, AT3G14630, AT3G14640, AT3G14650, AT3G14660, AT3G14710, AT3G14720, AT3G14730, AT3G14530, AT3G14550, AT3G14570, AT3G18040, AT3G18215, AT3G24490, AT3G24570, AT3G24590, AT3G24550, AT3G24500, AT3G24530, AT3G24506, AT3G24560, AT3G24515, AT3G24495, AT3G24520, AT3G24503, AT3G18035, AT3G18170, AT3G18200, AT3G18060, AT3G18090, AT3G23780, AT3G18110, AT3G18160, AT3G18080, AT3G18190, AT3G18165, AT3G18210, AT3G18130, AT3G18050, AT3G18100, AT3G17790, AT3G17840, AT3G17820, AT3G17930, AT3G17810, AT3G17940, AT3G17910, AT3G17900, AT3G21540, AT3G21560, AT3G21570, AT3G21600, AT3G21610, AT3G21630, AT3G21470, AT3G21640, AT3G21650, AT3G21680, AT3G21690, AT3G21670, AT3G21550, AT3G21510, AT3G21580, AT3G17850, AT1G48490, AT3G17880, AT3G17800, AT3G17860, AT3G29100, AT3G29110, AT3G29090, AT3G29170, AT3G13980, AT3G14000, AT3G14020, AT3G13910, AT3G13940, AT3G13950, AT3G13965, AT3G14010, AT3G14050, AT3G13930, AT3G14060, AT3G13920, AT3G17890, AT3G17920, AT3G17950, AT3G17970, AT3G17998, AT3G18000, AT3G17830, AT3G29160, AT3G15680, AT3G15630, AT3G15730, AT3G15620, AT3G27610, AT3G27700, AT3G27710, AT3G27720, AT3G27730, AT3G27670, AT3G27640, AT3G27740, AT3G27770, AT3G29075, AT5G39060, AT1G62766, AT3G29120, AT1G08105, AT3G15820, AT3G15830, AT3G15850, AT3G15810, AT3G15720, AT3G26760, AT3G26800, AT3G26890, AT3G26770, AT3G26790, AT3G26820, AT3G26840, AT3G26850, AT3G15740, AT3G15770, AT3G15780, AT3G15790, AT3G15800, AT3G15880, AT3G15690, AT3G15650, AT3G15610, AT3G15660, AT3G15640, AT3G15760, AT3G15710, AT3G26830, AT3G26880, AT3G26900, AT3G26910, AT3G26750, AT3G16080, AT3G16140, AT3G16060, AT3G15990, AT3G23310, AT3G23330, AT3G23430, AT3G23400, AT3G23390, AT3G23325, AT3G23300, AT3G23410, AT3G23340, AT3G30560, AT3G26744, AT3G26810, AT3G16170, AT3G15970, AT3G16100, AT3G16175, AT3G22660, AT3G22670, AT3G22680, AT3G22840, AT3G22740, AT3G22750, AT3G22780, AT3G22830, AT3G22760, AT3G16090, AT3G16110, AT3G15980, AT3G16180, AT3G16000, AT3G16010, AT3G16020, AT3G16050, AT3G16040, AT3G16150, AT3G16120, AT3G22770, AT3G22790, AT3G22800, AT3G22810, AT3G22845, AT3G22690, AT3G17520, AT3G17710, AT3G17609, AT3G17510, AT3G17700, AT3G17650, AT5G45450, AT3G14320, AT3G14330, AT3G14340, AT3G14350, AT3G14380, AT3G14415, AT3G14420, AT3G14270, AT3G14280, AT3G14225, AT5G11880, AT3G14390, AT3G14230, AT3G14370, AT3G14240, AT3G14310, AT3G14410, AT3G14400, AT3G14290, AT3G14360, AT3G17470, AT3G17680, AT3G17690, AT3G17490, AT3G16700, AT3G16650, AT3G16630, AT3G15355, AT3G15520, AT3G15370, AT3G15390, AT3G15410, AT3G15420, AT3G15480, AT3G15530, AT3G15500, AT3G15460, AT3G15357, AT3G15360, AT3G15540, AT3G15470, AT3G15430, AT3G15450, AT3G15380, AT3G17465, AT3G17590, AT3G17611, AT3G17630, AT3G16780, AT3G17280, AT3G17210, AT3G17300, AT3G17380, AT3G17390, AT3G17310, AT3G17365, AT3G16680, AT3G16690, AT3G16710, AT3G16730, AT3G16750, AT3G16760, AT3G16785, AT3G16620, AT3G16570, AT3G16640, AT3G16770, AT3G16740, AT3G16720, AT3G16560, AT3G17230, AT3G17240, AT3G17250, AT3G22380, AT3G22400, AT3G22430, AT3G22450, AT3G22290, AT3G22330, AT3G22310, AT3G22370, AT3G22320, AT3G22300, AT3G22440, AT3G22420, AT3G22425, AT3G22435, AT3G17330, AT3G17340, AT3G17350, AT3G17410, AT3G17360, AT3G06040, AT3G06060, AT3G06070, AT3G06050, AT3G06035, AT3G06030, AT3G06080, AT3G02650, AT3G02660, AT3G02400, AT3G02680, AT3G02710, AT3G02640, AT3G02600, AT3G02530, AT3G02570, AT3G02580, AT3G02630, AT3G02560, AT3G02550, AT3G02420, AT3G02520, AT3G02700, AT3G02555, AT3G02450, AT3G02690, AT3G02460, AT3G02380, AT3G02480, AT3G02540, AT3G02468, AT3G02470, AT3G06780, AT3G02490, AT3G02590, AT3G06530, AT3G06520, AT3G06500, AT3G06620, AT3G06600, AT3G06590, AT3G06510, AT3G06630, AT3G06610, AT3G06650, AT3G06490, AT3G06580, AT3G06570, AT3G06760, AT3G06680, AT3G06660, AT3G06830, AT3G06810, AT3G06790, AT3G06850, AT3G06770, AT3G06740, AT3G06840, AT3G06720, AT3G06700, AT3G06670, AT3G06730, AT3G06750, AT3G06540, AT3G06950, AT3G06990, AT3G07000, AT3G06870, AT3G07040, AT3G07050, AT3G07060, AT3G06910, AT3G06930, AT3G06940, AT3G06890, AT3G07010, AT3G06960, AT3G06980, AT3G06860, AT3G07020, AT3G05990, AT3G06010, AT3G06020, AT3G05980, AT3G05970, AT3G04350, AT3G04110, AT3G04080, AT3G04470, AT3G04400, AT3G04530, AT3G04420, AT3G04270, AT3G04260, AT3G04240, AT3G04230, AT3G04220, AT3G04200, AT3G04160, AT3G04150, AT3G04090, AT3G04340, AT3G04320, AT3G04330, AT3G04310, AT3G04120, AT3G04130, AT3G04480, AT3G04500, AT3G04520, AT3G02760, AT3G02750, AT3G02990, AT3G02980, AT3G02970, AT3G02840, AT3G02730, AT3G03020, AT3G02910, AT3G02875, AT3G02860, AT3G02920, AT3G02870, AT3G02770, AT3G02740, AT3G02880, AT3G03000, AT3G02885, AT3G02780, AT3G02940, AT3G02830, AT3G02720, AT3G02790, AT3G02950, AT3G04370, AT3G04460, AT3G02890, AT3G02850, AT3G02820, AT3G07970, AT3G07980, AT3G13530, AT3G07990, AT3G08000, AT3G08020, AT3G07890, AT3G07880, AT3G07950, AT3G07930, AT3G08030, AT3G07910, AT3G08010, AT3G07810, AT3G08040, AT3G07870, AT3G07900, AT3G09840, AT3G09830, AT3G09920, AT3G09910, AT3G09810, AT3G09900, AT3G09890, AT3G09820, AT3G09880, AT3G09850, AT3G02930, AT3G02900, AT3G08670, AT3G08700, AT3G08710, AT3G08550, AT3G08800, AT3G08810, AT3G08570, AT3G08620, AT3G08740, AT3G08770, AT3G08600, AT3G08730, AT3G08690, AT3G08590, AT3G08580, AT3G08610, AT3G08720, AT3G08760, AT3G08680, AT3G07860, AT3G07940, AT3G07960, AT3G08630, AT3G08640, AT3G08650, AT3G09650, AT3G09690, AT3G09470, AT3G09710, AT3G09720, AT3G09730, AT3G09780, AT3G09570, AT3G09480, AT3G09500, AT4G08520, AT3G09800, AT3G09680, AT5G02960, AT3G09735, AT3G09770, AT3G09630, AT3G09700, AT3G09740, AT3G09760, AT3G09540, AT3G09550, AT3G09560, AT3G09600, AT3G11745, AT3G11930, AT3G11800, AT3G11900, AT3G11730, AT3G11880, AT3G05850, AT3G05880, AT3G05900, AT3G05940, AT3G05750, AT3G05800, AT5G26751, AT3G05840, AT3G05830, AT3G05920, AT3G05950, AT3G05810, AT3G05760, AT3G05910, AT3G05890, AT3G09520, AT3G11945, AT3G11960, AT3G10912, AT3G10910, AT3G10810, AT3G10800, AT3G10760, AT3G10720, AT3G10710, AT3G10700, AT3G10880, AT3G10870, AT3G10830, AT3G10770, AT3G10860, AT3G10730, AT3G10740, AT3G10850, AT3G11830, AT3G11850, AT3G11890, AT3G11860, AT3G11910, AT3G11750, AT3G11760, AT3G11770, AT3G11820, AT3G11840, AT3G11780, AT3G04605, AT3G04610, AT3G04720, AT3G04640, AT3G04780, AT3G04710, AT3G04570, AT3G04630, AT3G04730, AT3G04770, AT3G04550, AT3G04560, AT3G04670, AT3G04760, AT3G04010, AT3G04020, AT3G04030, AT3G04070, AT3G03970, AT3G03990, AT3G03950, AT3G04060, AT3G03980, AT3G04000, AT3G03960, AT3G04750, AT3G04580, AT3G04590, AT3G04600, AT3G07230, AT3G07220, AT3G07260, AT3G07200, AT3G07190, AT3G07180, AT3G07160, AT3G07150, AT3G07130, AT3G07100, AT3G07090, AT3G07080, AT3G07274, AT3G07270, AT3G07210, AT3G07110, AT3G07140, AT3G07170, AT3G04620, AT3G04680, AT3G04740, AT3G03150, AT3G03070, AT3G03100, AT3G03300, AT3G03305, AT3G03250, AT3G03050, AT3G03190, AT3G06300, AT3G06440, AT3G02070, AT3G02040, AT3G02110, AT3G02080, AT3G02120, AT3G02050, AT3G02060, AT3G02090, AT3G03210, AT3G03200, AT3G03180, AT3G03130, AT3G03110, AT3G03090, AT3G03080, AT3G03060, AT3G03160, AT3G03120, AT3G03220, AT3G03170, AT3G03270, AT3G06420, AT3G06310, AT3G06330, AT3G09970, AT3G10050, AT3G10030, AT3G05620, AT3G05690, AT3G05670, AT3G05660, AT3G05650, AT3G05600, AT3G05710, AT3G05740, AT3G05675, AT3G05590, AT3G05640, AT3G06483, AT3G06410, AT3G06400, AT3G06390, AT3G06380, AT3G06370, AT3G06350, AT3G06340, AT3G06480, AT3G06470, AT3G06460, AT3G06450, AT3G10640, AT3G10670, AT3G10630, AT3G10525, AT3G10520, AT3G10500, AT3G10610, AT3G10450, AT3G10420, AT3G10490, AT3G10480, AT3G10160, AT3G10070, AT3G10060, AT3G10040, AT3G10010, AT3G09980, AT3G10140, AT3G10120, AT3G10090, AT3G10080, AT3G10020, AT3G09940, AT3G09960, AT3G10400, AT3G10390, AT3G10660, AT3G10650, AT3G01830, AT3G01910, AT3G01370, AT3G01460, AT3G01380, AT3G01410, AT3G01400, AT3G18530, AT3G01450, AT3G01440, AT3G01420, AT3G01390, AT3G10580, AT3G10550, AT3G10540, AT3G10530, AT3G10690, AT3G10680, AT3G10410, AT3G01920, AT3G01900, AT3G01890, AT3G01970, AT3G01980, AT3G02020, AT5G14060, AT3G09320, AT3G09440, AT3G08880, AT3G08860, AT3G08970, AT3G08960, AT3G08910, AT3G08950, AT3G08890, AT3G08840, AT3G08820, AT3G08930, AT3G08940, AT3G08850, AT3G01790, AT3G01800, AT3G01810, AT3G01820, AT3G01850, AT3G01860, AT3G02000, AT3G02010, AT3G01840, AT3G01930, AT3G09200, AT3G09370, AT3G09390, AT3G09260, AT3G07340, AT3G60990, AT3G07380, AT5G48190, AT3G07360, AT3G07430, AT3G07320, AT3G07350, AT3G07480, AT3G07310, AT3G07390, AT3G07370, AT3G07470, AT3G07540, AT3G07420, AT3G07510, AT3G09240, AT3G09280, AT3G09300, AT3G09310, AT3G09360, AT3G09380, AT3G09405, AT3G09410, AT3G09220, AT3G09350, AT3G09210, AT3G09250, AT3G09270, AT3G07330, AT3G03450, AT3G03440, AT3G03380, AT3G03370, AT3G03360, AT3G03330, AT3G03490, AT3G03320, AT3G03340, AT3G03420, AT3G03310, AT3G03470, AT3G07300, AT3G07400, AT3G07410, AT3G07440, AT3G07490, AT3G07530, AT3G07550, AT3G07560, AT3G11710, AT3G11670, AT3G11650, AT3G11700, AT3G02290, AT3G02130, AT3G02320, AT3G02140, AT3G02230, AT5G15650, AT3G05040, AT3G04910, AT3G05030, AT3G04820, AT3G04810, AT3G05000, AT3G04970, AT3G04920, AT3G04890, AT3G04870, AT3G04860, AT3G04940, AT3G05020, AT3G04840, AT3G04830, AT3G04790, AT3G05010, AT3G04880, AT3G11660, AT3G11690, AT3G02170, AT3G05500, AT3G05490, AT3G05540, AT3G05580, AT3G05570, AT3G05520, AT3G05560, AT3G05510, AT3G05530, AT3G01360, AT3G01350, AT3G01320, AT3G01310, AT3G01340, AT3G02240, AT3G02220, AT3G02210, AT3G02200, AT3G02300, AT3G02280, AT3G02260, AT3G02190, AT3G02180, AT3G02250, AT3G05400, AT3G05390, AT3G05480, AT3G05470, AT3G10300, AT5G04130, AT3G10270, AT3G10230, AT3G10220, AT3G10190, AT3G10180, AT3G10370, AT3G10350, AT3G10340, AT3G10320, AT3G10310, AT3G10210, AT3G10250, AT3G10260, AT3G10330, AT3G10380, AT3G05430, AT3G05420, AT3G05410, AT3G01640, AT3G01770, AT3G01470, AT3G01472, AT3G01670, AT3G03570, AT3G03520, AT3G03590, AT3G03630, AT3G03640, AT3G03690, AT3G01560, AT3G01610, AT3G01650, AT3G01660, AT3G01680, AT3G01690, AT3G01750, AT3G01520, AT3G01590, AT3G01510, AT3G01780, AT3G01720, AT3G01740, AT3G01480, AT3G01540, AT3G01730, AT3G03600, AT3G03670, AT3G03610, AT3G08980, AT3G09070, AT3G09100, AT3G09030, AT3G09040, AT3G09050, AT3G09060, AT3G09160, AT3G09150, AT3G09020, AT3G09180, AT3G09010, AT3G09085, AT3G09090, AT3G09000, AT3G03620, AT3G03580, AT3G03560, AT3G03550, AT3G03530, AT3G03540, AT3G03680, AT3G03660, AT3G07800, AT3G07590, AT3G07700, AT3G07750, AT3G07720, AT3G07740, AT3G07780, AT3G11410, AT3G11420, AT3G11620, AT3G11600, AT3G11530, AT3G11630, AT3G11400, AT3G11510, AT3G11590, AT3G33530, AT3G33520, AT3G07565, AT3G07650, AT3G07660, AT3G07730, AT4G01170, AT3G07770, AT3G07790, AT3G07580, AT3G07600, AT3G07610, AT3G07640, AT3G07630, AT3G07690, AT3G07680, AT3G07760, AT3G07670, AT3G11540, AT3G11370, AT3G11390, AT3G01260, AT3G01130, AT3G01070, AT3G01270, AT3G01280, AT3G01170, AT3G01290, AT5G15080, AT3G01300, AT3G01220, AT3G01060, AT3G01190, AT3G01180, AT3G01160, AT3G01120, AT3G01150, AT3G01140, AT3G11430, AT3G11440, AT3G11450, AT3G11490, AT3G11500, AT3G11520, AT3G11550, AT3G11560, AT3G11580, AT3G05350, AT3G05360, AT3G05370, AT3G05100, AT3G05120, AT3G05130, AT3G05210, AT3G05070, AT3G05290, AT3G05230, AT3G05180, AT3G05050, AT3G05160, AT3G05165, AT3G05060, AT3G05200, AT3G05090, AT3G01200, AT3G01175, AT3G01100, AT3G01090, AT3G01040, AT3G05270, AT3G05280, AT3G05320, AT3G16480, AT3G16460, AT3G16440, AT3G16360, AT3G16270, AT3G03810, AT3G03790, AT3G03930, AT3G03940, AT3G03750, AT3G03720, AT3G03900, AT3G03870, AT3G03740, AT3G03890, AT3G03780, AT5G17920, AT3G03710, AT3G03700, AT3G04440, AT3G03910, AT3G03920, AT3G03770, AT3G03860, AT3G05140, AT3G05150, AT3G05170, AT3G05190, AT3G05220, AT3G05240, AT3G05250, AT1G13380, AT1G13250, AT1G13460, AT1G13440, AT1G13350, AT3G53640, AT1G13270, AT1G13260, AT1G13300, AT1G13280, AT1G69070, AT3G43890, AT1G69150, AT1G69050, AT1G69080, AT3G16280, AT3G16370, AT3G16320, AT3G16380, AT3G16310, AT3G16350, AT3G16340, AT3G16410, AT3G16390, AT3G16400, AT3G16430, AT3G16420, AT3G16470, AT3G16450, AT1G13420, AT1G06350, AT1G06360, AT1G06380, AT1G06410, AT1G06420, AT1G06400, AT1G06390, AT1G17680, AT1G17710, AT1G17750, AT1G17760, AT1G17730, AT1G17745, AT1G17720, AT1G17690, AT1G13320, AT3G25800, AT1G13330, AT1G13340, AT1G13410, AT1G13450, AT1G02420, AT1G02330, AT1G02305, AT1G02340, AT1G02390, AT1G06310, AT1G13900, AT1G13910, AT1G13810, AT1G13880, AT1G13830, AT1G01470, AT1G01560, AT1G01600, AT1G01610, AT1G01620, AT1G01520, AT1G01570, AT1G01490, AT1G01500, AT1G01540, AT1G01510, AT1G01480, AT1G01550, AT1G02300, AT1G02310, AT1G02360, AT1G02370, AT1G02400, AT1G02410, AT1G02430, AT1G02440, AT1G13800, AT1G13870, AT1G44050, AT1G44030, AT1G44085, AT1G44000, AT1G18900, AT1G18940, AT1G18950, AT1G19050, AT1G19080, AT3G55490, AT1G19100, AT1G19110, AT1G18910, AT1G19025, AT1G19130, AT1G19140, AT1G19000, AT1G19120, AT1G18980, AT1G19020, AT1G19010, AT1G18970, AT1G12580, AT1G15700, AT1G15710, AT1G15720, AT1G15780, AT1G15790, AT1G15820, AT1G15840, AT1G15850, AT1G15890, AT1G15860, AT1G15880, AT1G15730, AT1G15740, AT1G15750, AT1G15670, AT1G15870, AT1G15690, AT1G15810, AT1G43900, AT1G43910, AT1G12520, AT1G12360, AT1G12430, AT1G20430, AT1G20450, AT1G20630, AT1G20510, AT1G12270, AT1G12320, AT1G12410, AT1G12500, AT1G12550, AT1G12560, AT1G12600, AT1G12330, AT1G12400, AT1G12450, AT1G12470, AT1G12530, AT1G12290, AT1G12280, AT1G12350, AT5G02080, AT1G12370, AT1G12310, AT1G20550, AT1G20640, AT1G20650, AT1G20575, AT1G20580, AT1G20620, AT1G20560, AT1G35350, AT1G35260, AT1G35230, AT1G21190, AT1G21310, AT1G21220, AT1G21210, AT1G21230, AT1G21270, AT1G21250, AT1G21200, AT1G20590, AT1G20610, AT1G20540, AT1G20500, AT1G20490, AT1G20440, AT1G20410, AT1G35320, AT1G35330, AT1G35340, AT1G28010, AT1G28030, AT1G28060, AT1G28070, AT1G28080, AT1G28090, AT1G28100, AT1G28120, AT1G27930, AT1G28150, AT1G27950, AT1G27970, AT1G28140, AT1G28050, AT1G28130, AT1G27900, AT1G27980, AT1G28110, AT1G35220, AT1G56500, AT1G56450, AT1G27910, AT1G27920, AT1G27960, AT1G52320, AT1G52280, AT1G52300, AT1G47960, AT1G47970, AT1G48000, AT1G43020, AT1G42970, AT1G42990, AT1G42960, AT1G56510, AT1G56350, AT1G56410, AT1G56345, AT1G52260, AT1G52360, AT1G52380, AT1G52400, AT1G69020, AT1G69030, AT1G68910, AT1G69010, AT1G55350, AT1G55325, AT1G55300, AT1G55290, AT1G55280, AT1G55250, AT1G55240, AT1G55210, AT1G55200, AT1G55180, AT1G55170, AT1G55260, AT1G55265, AT1G55190, AT1G55330, AT1G55160, AT1G55310, AT1G55270, AT1G52340, AT1G52310, AT1G52290, AT1G68990, AT1G77800, AT1G77700, AT1G77710, AT1G77730, AT1G77740, AT1G77750, AT1G77720, AT1G77690, AT1G77760, AT1G63050, AT1G63090, AT1G63100, AT1G63110, AT1G63120, AT1G63130, AT1G63160, AT1G63200, AT1G63080, AT1G63180, AT1G63170, AT1G68920, AT1G68930, AT1G68940, AT1G68980, AT1G77770, AT1G77780, AT1G77790, AT1G77680, AT1G77450, AT1G77540, AT1G77420, AT1G77670, AT1G77570, AT1G77440, AT1G77610, AT1G60990, AT1G60995, AT1G61010, AT1G61040, AT1G61070, AT1G60890, AT1G60930, AT1G60960, AT1G60940, AT1G61065, AT1G60950, AT1G60970, AT1G77640, AT1G77660, AT1G77460, AT1G77470, AT1G77480, AT1G77500, AT1G64405, AT1G64390, AT1G64400, AT1G64355, AT1G64350, AT1G64300, AT1G71020, AT1G71140, AT1G71190, AT1G71060, AT1G71170, AT1G71180, AT1G71100, AT1G71130, AT1G71030, AT1G71040, AT1G71050, AT1G71090, AT1G71070, AT1G71080, AT1G77510, AT1G77530, AT1G77520, AT1G77550, AT1G77580, AT1G77590, AT1G77600, AT1G77630, AT1G64280, AT1G64370, AT1G29370, AT1G29350, AT1G29390, AT1G29400, AT1G29410, AT5G05590, AT1G07780, AT1G29470, AT1G29520, AT1G29530, AT1G29590, AT1G29550, AT1G29610, AT1G29630, AT1G29660, AT1G29670, AT1G29395, AT1G29700, AT1G29690, AT1G64380, AT1G64330, AT1G64270, AT1G64310, AT1G72175, AT1G72230, AT1G72150, AT1G72160, AT1G47890, AT1G47900, AT1G47920, AT1G49790, AT1G49840, AT1G49860, AT1G49870, AT1G49850, AT1G49890, AT1G49820, AT1G66250, AT1G66240, AT1G66260, AT1G29340, AT1G72220, AT1G80630, AT1G69600, AT1G69580, AT1G69570, AT1G52160, AT1G52180, AT1G52220, AT1G52230, AT1G52200, AT1G73602, AT1G73600, AT1G73590, AT1G72140, AT1G72170, AT1G72180, AT1G72190, AT1G72240, AT1G72250, AT1G72280, AT1G72300, AT1G72200, AT1G68440, AT1G68430, AT1G68400, AT1G68410, AT1G80640, AT1G80620, AT1G72650, AT1G72630, AT1G72800, AT1G72710, AT1G76640, AT1G76740, AT1G76760, AT1G76770, AT1G76780, AT1G76800, AT1G76810, AT1G76720, AT1G76820, AT1G76660, AT1G76680, AT1G76690, AT1G76700, AT1G76710, AT1G76790, AT1G76650, AT1G76730, AT1G76670, AT1G76400, AT1G76390, AT1G76380, AT1G76360, AT1G76350, AT1G76405, AT1G72750, AT1G72730, AT1G72610, AT1G71430, AT1G71440, AT1G71500, AT1G71380, AT1G71210, AT1G71230, AT1G74180, AT1G74170, AT1G74160, AT1G74150, AT1G74140, AT1G74130, AT1G74120, AT1G60860, AT1G60850, AT1G60870, AT1G72700, AT1G72680, AT1G72670, AT1G72790, AT1G72560, AT1G72550, AT1G72540, AT1G72770, AT1G72740, AT1G75090, AT1G75100, AT1G34380, AT1G34355, AT1G34370, AT1G34350, AT1G71340, AT3G13080, AT1G71330, AT1G71310, AT1G71270, AT1G71300, AT1G71260, AT1G71480, AT1G71470, AT1G71460, AT1G71530, AT1G71350, AT1G71420, AT1G71410, AT1G71400, AT1G74810, AT1G75130, AT1G75080, AT1G19350, AT1G67890, AT1G53730, AT1G53720, AT1G53710, AT1G53700, AT1G53660, AT1G53680, AT1G53670, AT1G76500, AT1G76510, AT1G76530, AT1G76550, AT1G76570, AT1G76580, AT1G76630, AT1G76430, AT1G76560, AT1G76590, AT1G76540, AT1G76600, AT1G76520, AT1G76450, AT1G76410, AT1G76490, AT1G76460, AT1G76620, AT1G75030, AT1G68050, AT5G42730, AT5G23410, AT1G68040, AT1G67970, AT1G67960, AT1G68130, AT1G67930, AT1G67910, AT1G67880, AT1G68120, AT1G68080, AT1G68060, AT1G67990, AT1G67940, AT1G68100, AT1G67920, AT1G68000, AT1G67870, AT1G68070, AT1G67950, AT1G68020, AT1G67865, AT1G68010, AT1G67980, AT1G29990, AT1G30010, AT1G30020, AT1G30040, AT1G30080, AT1G30070, AT1G29970, AT1G29952, AT1G29951, AT1G29950, AT1G29980, AT1G30000, AT1G68880, AT1G68790, AT1G68760, AT1G68810, AT1G68850, AT1G68830, AT1G68840, AT1G68795, AT1G68765, AT1G68820, AT1G73655, AT1G73670, AT1G73805, AT1G73740, AT1G73750, AT1G29940, AT1G78210, AT1G78220, AT1G78120, AT1G78130, AT1G78150, AT1G78140, AT1G78200, AT1G78100, AT1G78090, AT1G73680, AT1G73690, AT1G73720, AT1G73730, AT1G73760, AT1G73610, AT1G73780, AT1G73820, AT1G73830, AT1G73840, AT1G73650, AT1G73660, AT1G73640, AT1G73630, AT1G73620, AT1G73790, AT5G33210, AT1G35460, AT1G78160, AT1G78170, AT1G78180, AT1G63220, AT1G63290, AT1G73200, AT1G73170, AT1G73310, AT1G73290, AT1G73280, AT1G73270, AT1G73250, AT1G73230, AT1G73240, AT1G73220, AT1G73320, AT1G73180, AT1G73177, AT1G73260, AT1G35470, AT1G35430, AT1G35410, AT1G35480, AT1G35420, AT1G66430, AT1G63260, AT1G63350, AT1G63310, AT1G63300, AT1G33880, AT1G33870, AT1G33940, AT5G18700, AT1G33890, AT1G36390, AT1G36370, AT1G36320, AT1G36310, AT1G36380, AT1G66280, AT1G66270, AT1G66340, AT1G66370, AT1G66400, AT1G66330, AT1G66410, AT1G66350, AT1G71900, AT1G71880, AT1G71960, AT1G52920, AT1G52810, AT1G52820, AT1G52910, AT1G52760, AT1G52740, AT1G52870, AT1G52880, AT1G52930, AT1G79890, AT1G79880, AT1G79860, AT1G79830, AT1G79870, AT1G79850, AT1G79840, AT1G71970, AT1G71980, AT1G71990, AT1G71910, AT5G10330, AT1G71920, AT1G71930, AT1G71940, AT1G71950, AT1G70650, AT1G70690, AT1G70660, AT1G70710, AT1G70750, AT1G35950, AT2G05084, AT5G34950, AT1G67550, AT1G67560, AT1G67580, AT1G67630, AT1G67530, AT1G67570, AT1G67623, AT1G67590, AT1G67620, AT1G67600, AT1G52840, AT1G52900, AT1G70740, AT1G70730, AT1G70670, AT1G70680, AT1G68552, AT1G68550, AT1G74420, AT1G74440, AT1G74490, AT1G74510, AT1G74530, AT1G74600, AT1G74670, AT1G74380, AT1G74400, AT1G74590, AT1G74660, AT1G74450, AT1G74370, AT1G74500, AT1G74410, AT1G74640, AT1G74550, AT1G74460, AT1G74560, AT1G74470, AT1G74430, AT1G74520, AT1G74360, AT1G68460, AT1G63650, AT1G63720, AT1G74320, AT1G74230, AT1G74310, AT1G74290, AT1G74280, AT1G74260, AT1G74250, AT1G74240, AT1G74210, AT1G74330, AT1G74350, AT1G74340, AT1G74270, AT1G74300, AT1G68540, AT1G68450, AT1G68470, AT1G68500, AT1G68520, AT1G68530, AT1G68490, AT1G63740, AT1G63730, AT1G63690, AT1G80570, AT1G80550, AT1G80460, AT1G80450, AT1G80520, AT1G80590, AT1G80420, AT1G80500, AT1G80410, AT1G80480, AT1G80380, AT1G80560, AT1G80600, AT1G80440, AT1G80530, AT1G80350, AT1G80510, AT1G63700, AT1G63680, AT1G63670, AT1G63660, AT1G63770, AT1G63750, AT1G80280, AT1G80340, AT1G80290, AT1G80310, AT1G70520, AT1G70480, AT1G70490, AT1G70620, AT1G70610, AT1G70580, AT1G70590, AT1G70570, AT1G63880, AT1G63830, AT1G63820, AT1G63810, AT1G63800, AT1G63900, AT1G63780, AT1G63970, AT1G63940, AT1G63910, AT1G63840, AT1G80360, AT1G80370, AT1G80400, AT1G80270, AT1G70470, AT1G70500, AT1G70510, AT1G74000, AT1G73990, AT1G69310, AT1G69250, AT1G69220, AT1G69170, AT1G69200, AT1G69240, AT1G69270, AT1G69420, AT1G69350, AT1G69400, AT1G69410, AT1G69380, AT1G69295, AT1G69230, AT1G69330, AT1G69390, AT1G69260, AT1G69340, AT1G69210, AT1G69370, AT1G69360, AT1G70530, AT1G70550, AT1G70560, AT1G70630, AT1G70460, AT1G69760, AT1G69700, AT1G69620, AT1G69740, AT1G69690, AT1G73980, AT1G73970, AT1G73960, AT1G73950, AT1G73930, AT1G73910, AT1G73850, AT1G74060, AT1G74050, AT1G74040, AT1G74090, AT1G74010, AT1G74100, AT1G74055, AT1G73940, AT1G74020, AT1G74030, AT1G73920, AT1G74080, AT1G69780, AT1G69680, AT1G72320, AT1G69840, AT1G69820, AT1G69810, AT1G69950, AT1G69930, AT1G69920, AT1G69910, AT1G69880, AT1G69935, AT1G69870, AT1G69890, AT1G69830, AT1G69800, AT1G69850, AT1G69790, AT1G69670, AT1G69770, AT1G69750, AT1G66590, AT1G69610, AT1G69640, AT1G72470, AT1G72440, AT1G72370, AT1G72430, AT1G72340, AT1G72450, AT1G68150, AT1G68330, AT1G68320, AT1G68140, AT1G68290, AT1G68340, AT1G68160, AT1G68260, AT1G68220, AT1G68185, AT1G68300, AT1G68370, AT1G72420, AT1G72410, AT1G72390, AT1G72360, AT1G72510, AT2G09970, AT1G72310, AT1G72500, AT1G72480, AT1G68250, AT1G68240, AT1G68230, AT1G10970, AT1G10910, AT1G10900, AT1G11100, AT1G10870, AT1G11120, AT1G11070, AT1G10990, AT1G11090, AT1G11080, AT1G11060, AT1G11050, AT1G11020, AT1G10940, AT1G10960, AT1G10950, AT1G11000, AT1G68360, AT1G51550, AT1G51510, AT1G51600, AT1G51610, AT1G51500, AT2G41850, AT2G41830, AT2G41820, AT2G41700, AT2G41840, AT2G41710, AT2G41760, AT2G41705, AT2G41770, AT1G47270, AT1G47250, AT1G47230, AT1G47210, AT1G47200, AT1G47260, AT1G47240, AT1G51580, AT1G51570, AT1G51520, AT1G51490, AT1G51480, AT1G51460, AT1G51450, AT1G51420, AT1G51540, AT1G51590, AT2G41740, AT2G41730, AT2G41720, AT2G47230, AT2G47250, AT2G47410, AT2G47270, AT2G47260, AT2G47400, AT2G47320, AT2G47240, AT2G47330, AT2G43390, AT2G43400, AT2G43450, AT2G43460, AT2G43480, AT2G43500, AT2G43560, AT2G43330, AT2G43540, AT2G43350, AT2G43550, AT2G43360, AT2G43535, AT2G43420, AT2G43510, AT2G43430, AT2G41800, AT2G41790, AT2G41780, AT2G43620, AT2G43610, AT2G43600, AT2G43590, AT2G43580, AT2G43850, AT2G43840, AT2G43810, AT2G43800, AT2G43750, AT2G43820, AT2G43570, AT2G43640, AT2G43710, AT2G43790, AT2G43760, AT2G47310, AT2G47350, AT2G47380, AT2G47390, AT2G47190, AT2G47200, AT2G43660, AT2G43650, AT2G43630, AT1G53210, AT1G53320, AT1G53400, AT1G53310, AT1G55850, AT1G55870, AT1G55880, AT1G55890, AT1G55900, AT1G55915, AT1G55930, AT1G55940, AT1G55910, AT1G55920, AT1G55960, AT1G55840, AT2G43780, AT2G43770, AT2G43700, AT2G43690, AT2G43680, AT2G43670, AT1G53360, AT1G53380, AT1G53390, AT1G53240, AT1G53280, AT1G53345, AT1G53230, AT1G08110, AT1G08070, AT1G07980, AT1G08090, AT1G08060, AT1G08040, AT1G62310, AT1G62330, AT1G62350, AT1G62422, AT1G62430, AT1G62380, AT1G62390, AT1G53180, AT1G53350, AT1G53200, AT1G53260, AT1G53270, AT1G53290, AT1G53300, AT1G53340, AT1G08030, AT1G08050, AT1G32370, AT1G32460, AT1G32360, AT1G32440, AT1G19290, AT1G19380, AT1G19410, AT1G19485, AT1G19540, AT1G19520, AT1G19360, AT1G19300, AT1G19440, AT1G19530, AT1G19340, AT1G19310, AT1G19480, AT1G19330, AT1G19370, AT1G19430, AT1G19450, AT1G07910, AT1G07970, AT1G07990, AT1G08010, AT1G08000, AT1G17510, AT1G17560, AT1G17590, AT1G17650, AT1G17620, AT1G17530, AT1G17665, AT1G32340, AT2G23880, AT1G32390, AT1G32430, AT1G32450, AT1G32490, AT1G32520, AT1G32530, AT1G32300, AT1G32260, AT1G32330, AT1G32270, AT1G32470, AT1G32400, AT1G32410, AT1G32310, AT1G32500, AT1G17580, AT1G17550, AT1G17615, AT1G14980, AT1G48200, AT1G48120, AT1G48110, AT1G48090, AT1G48040, AT1G48140, AT1G48170, AT1G48160, AT1G48100, AT1G48210, AT1G48050, AT1G48030, AT1G17500, AT1G17520, AT1G17640, AT1G17610, AT1G17630, AT1G14990, AT1G15000, AT1G15020, AT1G15040, AT1G15010, AT1G78370, AT1G78380, AT1G78420, AT1G49210, AT1G49040, AT1G49030, AT1G49230, AT1G48980, AT1G48970, AT1G48930, AT1G49180, AT1G49170, AT1G49220, AT1G48950, AT1G49240, AT1G48960, AT1G49140, AT3G18410, AT1G49245, AT1G49010, AT1G49200, AT1G49160, AT1G49050, AT1G78440, AT1G78290, AT1G78300, AT1G14580, AT1G14560, AT1G14640, AT1G14650, AT1G14610, AT1G14620, AT1G14570, AT1G06160, AT1G06180, AT1G06200, AT1G06220, AT5G25230, AT1G06230, AT3G06690, AT1G06290, AT1G06240, AT1G06250, AT1G06190, AT1G06210, AT1G06225, AT1G78360, AT1G78280, AT1G78240, AT1G78430, AT1G78340, AT1G78410, AT1G78320, AT1G78260, AT1G78310, AT1G43715, AT1G43730, AT1G43650, AT1G43700, AT1G43710, AT1G06950, AT1G06930, AT1G06900, AT1G06890, AT1G06850, AT1G06840, AT1G06820, AT1G06790, AT1G06770, AT1G06720, AT1G06710, AT1G06740, AT1G06870, AT2G30440, AT1G06760, AT1G06780, AT1G06800, AT1G06730, AT1G06700, AT1G43660, AT1G43670, AT1G43680, AT1G43690, AT1G29050, AT1G29195, AT1G29150, AT1G29060, AT1G29260, AT1G17300, AT1G17290, AT1G72330, AT1G21870, AT1G21900, AT1G21920, AT1G21910, AT1G21880, AT1G43770, AT1G43780, AT1G43850, AT1G43790, AT1G43860, AT1G43800, AT1G43890, AT1G29040, AT1G21350, AT1G21580, AT1G21410, AT1G21460, AT1G21450, AT1G21480, AT1G21380, AT1G21550, AT1G29280, AT1G29270, AT1G29240, AT1G29230, AT1G29160, AT1G29120, AT1G29180, AT1G29220, AT1G29250, AT1G29310, AT1G29330, AT1G29070, AT1G21370, AT5G34780, AT1G21400, AT1G21440, AT1G02460, AT1G02450, AT1G02570, AT1G02690, AT1G02560, AT1G02500, AT1G02640, AT1G02660, AT1G02475, AT1G21560, AT1G21570, AT1G21590, AT1G21360, AT1G05940, AT1G02720, AT1G02680, AT1G02620, AT1G02530, AT1G02520, AT1G02470, AT1G45110, AT1G45150, AT1G45160, AT1G45170, AT1G45180, AT1G45000, AT1G44970, AT1G45145, AT1G45130, AT1G06010, AT1G06100, AT1G06110, AT1G06120, AT1G06090, AT1G06145, AT1G06070, AT1G06130, AT1G06000, AT1G06040, AT1G06080, AT1G06060, AT1G06050, AT1G05960, AT1G44980, AT1G45050, AT1G12220, AT1G12230, AT1G12250, AT1G12240, AT1G53640, AT1G53530, AT1G53490, AT1G53440, AT1G53430, AT1G53650, AT1G53540, AT1G53510, AT1G53460, AT1G53520, AT1G53560, AT1G53645, AT1G53500, AT1G53580, AT1G53590, AT1G53570, AT1G53450, AT1G12160, AT1G12140, AT1G12120, AT1G12210, AT1G26610, AT1G26590, AT1G26580, AT1G26520, AT1G26450, AT1G26420, AT1G26410, AT1G26370, AT1G26350, AT1G26340, AT1G26440, AT1G26460, AT1G26560, AT1G26570, AT1G26550, AT1G26470, AT1G26390, AT1G26380, AT1G12260, AT1G12200, AT1G01225, AT1G01240, AT1G01340, AT1G26620, AT1G17330, AT1G17410, AT1G17360, AT1G17430, AT1G17380, AT1G59077, AT1G17450, AT1G59453, AT1G58766, AT1G17470, AT1G17490, AT1G17455, AT1G17370, AT1G17440, AT1G01200, AT1G01220, AT1G01290, AT1G01420, AT1G01430, AT1G01260, AT1G01440, AT1G01320, AT1G01300, AT1G01360, AT1G01230, AT1G01370, AT1G17340, AT1G07530, AT1G07520, AT1G07510, AT1G07500, AT1G07450, AT1G07410, AT1G07390, AT1G07380, AT1G07340, AT1G07300, AT1G07290, AT1G07280, AT1G07420, AT1G07430, AT1G07320, AT1G07480, AT1G07470, AT1G07370, AT1G07350, AT1G07360, AT1G07310, AT1G07440, AT1G17480, AT1G17460, AT1G17350, AT1G07590, AT1G07570, AT1G07560, AT1G07550, AT1G62930, AT1G62950, AT1G62970, AT1G62980, AT1G63020, AT1G62880, AT1G62910, AT1G63000, AT1G62990, AT1G63010, AT1G62960, AT1G75450, AT1G75440, AT1G75420, AT1G75400, AT1G75388, AT1G75390, AT1G75370, AT1G75310, AT1G75350, AT1G75410, AT1G75340, AT1G75330, AT1G62860, AT1G04900, AT1G04990, AT1G04950, AT1G04790, AT1G04860, AT1G04820, AT1G04870, AT1G19835, AT1G19800, AT1G19715, AT1G19610, AT1G19600, AT1G19830, AT1G19710, AT1G19700, AT1G19680, AT1G19740, AT1G19770, AT1G19660, AT1G75380, AT1G19730, AT1G19620, AT1G19690, AT1G19580, AT1G19570, AT1G19550, AT1G04930, AT1G04850, AT1G04970, AT1G04960, AT1G34490, AT1G34520, AT1G34500, AT1G34460, AT5G06150, AT1G34420, AT1G34440, AT1G34470, AT1G34430, AT3G15750, AT1G34570, AT1G04980, AT1G04945, AT1G04910, AT1G04830, AT1G04810, AT1G04780, AT1G04770, AT1G04760, AT1G04985, AT1G34580, AT1G34550, AT1G32860, AT1G33040, AT1G32990, AT1G32900, AT1G32870, AT1G32920, AT1G12775, AT1G12820, AT1G12860, AT1G12900, AT1G12950, AT1G12960, AT1G12850, AT1G12910, AT1G12845, AT1G12920, AT3G26618, AT1G12790, AT1G12800, AT1G12830, AT1G12930, AT1G12810, AT1G12840, AT1G12780, AT1G12880, AT1G12970, AT1G33030, AT1G19980, AT1G19970, AT1G19960, AT1G20120, AT1G19950, AT1G20160, AT1G20100, AT1G20190, AT1G20200, AT1G20050, AT1G20090, AT1G20010, AT1G20110, AT1G20220, AT1G32810, AT2G04970, AT2G14140, AT1G32840, AT1G32930, AT1G32940, AT1G32960, AT1G20180, AT1G20150, AT1G20070, AT1G20030, AT1G05880, AT1G05890, AT1G05810, AT1G05840, AT1G05805, AT1G05900, AT1G05760, AT1G05910, AT1G26640, AT1G26660, AT1G26730, AT1G26760, AT1G26790, AT1G26710, AT1G26800, AT1G26770, AT1G26750, AT1G26740, AT1G26690, AT1G26630, AT1G26795, AT1G26665, AT1G26650, AT1G26700, AT1G26670, AT1G20230, AT1G05850, AT1G05860, AT1G35720, AT1G35780, AT1G36990, AT1G36940, AT1G36980, AT1G48430, AT1G48450, AT1G48460, AT1G48510, AT1G48550, AT1G48560, AT1G48570, AT1G48580, AT1G48620, AT1G48440, AT1G48610, AT1G48520, AT1G48480, AT1G48598, AT1G48600, AT1G05790, AT1G52980, AT1G53170, AT1G53165, AT1G53030, AT1G53035, AT1G53090, AT1G53120, AT1G44750, AT1G44760, AT1G44770, AT1G44780, AT1G44180, AT1G44820, AT1G44830, AT1G44835, AT1G44900, AT1G44935, AT1G44910, AT1G44960, AT1G44800, AT1G44790, AT1G44810, AT1G44920, AT1G35710, AT1G79710, AT1G79760, AT1G79720, AT1G79730, AT1G79810, AT1G79790, AT1G79650, AT1G79600, AT1G79690, AT1G79750, AT1G79660, AT3G29450, AT1G41920, AT1G41880, AT3G55750, AT1G41830, AT1G53140, AT1G53110, AT1G53100, AT1G53070, AT1G53060, AT1G53050, AT1G53020, AT1G53010, AT1G53000, AT1G52990, AT1G53160, AT1G18660, AT1G18850, AT1G18890, AT1G18720, AT1G05460, AT1G05470, AT1G05380, AT1G05440, AT1G05450, AT1G05430, AT1G05410, AT1G05385, AT1G79580, AT1G79680, AT1G79700, AT1G79820, AT1G79610, AT1G79620, AT1G79630, AT1G79640, AT1G79670, AT1G79590, AT1G18690, AT1G18740, AT1G18800, AT1G07820, AT1G07660, AT1G07640, AT1G07610, AT1G07790, AT1G07890, AT1G07630, AT1G07750, AT1G07770, AT1G07700, AT1G07830, AT1G07840, AT1G07720, AT1G18840, AT1G18750, AT1G18700, AT1G18880, AT1G18670, AT1G18870, AT1G18860, AT1G07670, AT1G07810, AT1G07650, AT1G07615, AT1G07870, AT1G07600, AT1G28395, AT1G28510, AT1G28470, AT1G28480, AT1G28490, AT1G28380, AT1G21170, AT1G21160, AT1G21140, AT1G21120, AT1G21110, AT1G21060, AT5G44890, AT1G21020, AT2G29240, AT3G26530, AT1G08740, AT1G21130, AT1G21090, AT1G21070, AT1G21050, AT1G21065, AT1G21080, AT1G21100, AT1G07880, AT1G07740, AT1G07730, AT1G07705, AT1G07690, AT1G28520, AT1G28500, AT1G27860, AT1G28340, AT1G28370, AT1G14460, AT1G14470, AT1G14540, AT1G14550, AT1G14480, AT1G14310, AT1G14380, AT1G14280, AT1G14260, AT1G14530, AT1G14270, AT1G14400, AT1G14360, AT1G14510, AT1G14340, AT1G14345, AT1G14450, AT1G14320, AT1G14350, AT1G14290, AT1G14410, AT1G28420, AT1G28410, AT1G28360, AT1G28460, AT1G28440, AT1G28400, AT1G28390, AT1G71691, AT1G71692, AT1G71696, AT1G71697, AT1G71730, AT1G71740, AT1G71760, AT1G71770, AT1G71790, AT1G71840, AT1G71870, AT1G71800, AT1G71750, AT1G71780, AT1G71695, AT1G71810, AT1G71820, AT1G71830, AT1G71710, AT1G71860, AT1G14300, AT1G14330, AT1G14370, AT1G14390, AT1G63460, AT1G63490, AT1G01820, AT1G01725, AT1G01790, AT1G01800, AT1G01830, AT1G01840, AT1G63610, AT1G63550, AT1G63530, AT1G63500, AT1G63480, AT1G63470, AT1G63450, AT1G63440, AT1G63430, AT1G63420, AT1G63400, AT1G63630, AT1G63230, AT1G63640, AT1G01770, AT1G01780, AT1G01710, AT1G01730, AT1G01750, AT1G01630, AT1G01720, AT1G33230, AT1G33055, AT1G01030, AT1G01060, AT1G01120, AT1G01170, AT1G01180, AT1G01190, AT1G01070, AT1G01080, AT1G01100, AT1G01050, AT1G01110, AT1G01140, AT1G01160, AT1G01090, AT1G01040, AT1G01010, AT1G01640, AT1G01670, AT1G01740, AT1G33170, AT1G33240, AT1G33080, AT1G49780, AT1G49750, AT1G49700, AT1G49670, AT1G49630, AT1G49600, AT1G49590, AT1G49580, AT1G49570, AT1G49660, AT1G49740, AT1G49560, AT1G49730, AT1G49710, AT1G49720, AT1G49760, AT1G33050, AT1G33060, AT1G33100, AT1G33090, AT1G33110, AT1G33130, AT1G33140, AT1G33120, AT1G01960, AT1G01970, AT1G02000, AT1G01930, AT1G01990, AT1G01910, AT1G50000, AT1G49980, AT1G50120, AT1G49930, AT1G49970, AT1G50020, AT1G50110, AT1G49950, AT1G50010, AT1G49960, AT1G27670, AT1G27680, AT1G27690, AT1G27740, AT1G27730, AT1G27700, AT1G27770, AT1G27760, AT1G01860, AT1G01900, AT1G01920, AT1G01940, AT1G01950, AT1G50050, AT1G50030, AT1G18370, AT1G18360, AT1G18330, AT1G18320, AT3G10110, AT1G18440, AT1G18460, AT1G18500, AT1G18490, AT1G18340, AT1G18450, AT1G18270, AT1G18290, AT1G18480, AT1G18470, AT1G47350, AT1G47340, AT1G47330, AT1G47400, AT1G47290, AT1G47310, AT1G47380, AT1G50140, AT1G50090, AT1G50060, AT1G08450, AT1G08370, AT1G08360, AT1G08520, AT1G08470, AT1G32610, AT1G32630, AT1G32690, AT1G32750, AT1G32760, AT1G32740, AT1G32790, AT1G32730, AT1G32700, AT1G32780, AT1G32640, AT1G18400, AT1G18390, AT1G08460, AT1G08480, AT1G08510, AT1G76200, AT1G76185, AT1G76300, AT1G76270, AT1G76090, AT1G76160, AT1G47860, AT1G47840, AT1G47780, AT1G47786, AT1G47770, AT1G47830, AT1G47790, AT1G47813, AT1G47820, AT1G47750, AT1G47740, AT1G47870, AT1G08390, AT2G27210, AT1G08420, AT1G08490, AT1G08500, AT1G08380, AT1G08350, AT1G76070, AT1G76180, AT1G76120, AT1G76080, AT1G76340, AT1G76130, AT1G08315, AT1G08210, AT1G08250, AT1G15600, AT1G15660, AT1G15510, AT1G15520, AT1G15570, AT1G15500, AT1G80300, AT1G15550, AT1G76140, AT1G76150, AT1G76240, AT1G76260, AT1G76280, AT1G76190, AT1G76220, AT1G76230, AT1G76310, AT1G76320, AT1G08190, AT1G08220, AT1G08230, AT1G16020, AT1G15930, AT1G16000, AT1G16170, AT1G15950, AT1G16080, AT1G16060, AT1G16010, AT1G30440, AT1G30460, AT1G30490, AT1G30520, AT1G30550, AT1G30500, AT1G30530, AT1G30540, AT1G30510, AT1G30515, AT1G30480, AT1G08340, AT1G08130, AT1G08260, AT1G08270, AT1G08280, AT1G08310, AT1G08320, AT1G08135, AT1G08180, AT1G11390, AT1G11410, AT1G11420, AT1G11530, AT1G11380, AT1G11400, AT1G11430, AT1G11545, AT1G11580, AT1G11480, AT1G11360, AT1G10630, AT1G10650, AT1G10670, AT1G10640, AT1G16090, AT1G15910, AT4G00380, AT1G16030, AT1G16040, AT1G16070, AT1G15920, AT1G16160, AT1G16180, AT1G15940, AT1G11540, AT1G11560, AT1G50470, AT1G50460, AT1G50440, AT1G50660, AT1G50670, AT1G50620, AT1G50570, AT1G50490, AT1G50600, AT1G50450, AT1G50410, AT1G50630, AT1G50480, AT2G12280, AT2G12200, AT1G50430, AT1G50420, AT1G50575, AT1G11450, AT1G11460, AT1G11570, AT1G11440, AT1G11520, AT4G21660, AT1G11350, AT1G50640, AT1G50590, AT1G50580, AT1G50560, AT1G50520, AT1G50500, AT1G80760, AT1G80770, AT1G80870, AT1G80910, AT1G80930, AT1G80700, AT1G80980, AT1G80780, AT1G80800, AT1G80810, AT1G80790, AT1G80840, AT1G80690, AT1G80900, AT1G80830, AT1G80670, AT1G80950, AT1G80720, AT1G80820, AT1G80940, AT1G80920, AT1G80860, AT1G65070, AT1G65080, AT1G65060, AT1G80680, AT1G80730, AT1G80750, AT1G66060, AT1G66000, AT1G65910, AT1G65920, AT1G65980, AT1G65930, AT1G65900, AT1G22380, AT1G22550, AT1G22540, AT1G22530, AT1G22500, AT1G22460, AT1G22440, AT1G22430, AT1G22410, AT1G22400, AT1G22610, AT1G22600, AT1G22570, AT1G22510, AT1G22450, AT1G22520, AT1G22490, AT1G64650, AT1G64530, AT1G64470, AT1G64670, AT1G64620, AT1G64490, AT1G64440, AT1G64510, AT1G64520, AT1G64590, AT1G64660, AT1G64500, AT3G11970, AT1G36590, AT1G36550, AT1G36470, AT2G10630, AT1G50860, AT2G12260, AT1G65850, AT1G65950, AT1G65960, AT1G64600, AT1G64610, AT1G64628, AT1G64630, AT1G64640, AT1G37130, AT1G33780, AT1G33770, AT1G33750, AT1G33680, AT1G33660, AT1G33700, AT1G33640, AT1G33800, AT1G33810, AT1G33790, AT1G64460, AT1G64480, AT1G64550, AT1G64570, AT1G64580, AT1G35612, AT1G35625, AT1G35630, AT1G35660, AT1G35663, AT1G35513, AT1G35550, AT1G35617, AT1G35620, AT1G35510, AT1G35670, AT1G35560, AT1G35680, AT1G37160, AT1G37150, AT1G35530, AT1G35580, AT1G80050, AT1G80100, AT1G80190, AT3G06820, AT1G80210, AT1G80240, AT1G80160, AT1G80230, AT1G80180, AT1G80130, AT1G79990, AT1G80020, AT1G79940, AT1G79920, AT1G79930, AT1G79970, AT1G80245, AT1G80040, AT1G80030, AT1G80170, AT1G80110, AT1G80260, AT1G20570, AT1G80000, AT1G56070, AT1G56180, AT1G79950, AT1G80060, AT1G80070, AT1G80010, AT1G59510, AT1G59590, AT1G59560, AT1G59580, AT1G59610, AT1G59520, AT1G59600, AT1G59650, AT1G59540, AT1G56140, AT1G56130, AT1G56120, AT1G56080, AT1G56060, AT1G56050, AT1G56020, AT1G56000, AT1G55980, AT1G55970, AT1G56160, AT1G56145, AT1G56110, AT1G56090, AT1G56010, AT1G55990, AT1G56170, AT1G59500, AT4G37390, AT1G02980, AT1G02940, AT1G02910, AT1G02810, AT1G02970, AT1G02750, AT1G02740, AT1G02730, AT1G02840, AT1G02960, AT1G02870, AT1G02860, AT1G02816, AT1G02780, AT1G02850, AT1G02930, AT1G02920, AT1G02950, AT1G59660, AT1G59620, AT1G52550, AT1G52690, AT1G52500, AT1G02890, AT1G02900, AT1G78000, AT1G78080, AT1G77850, AT1G77920, AT1G52660, AT1G52470, AT1G52640, AT1G52630, AT1G52620, AT1G52730, AT1G52570, AT1G52560, AT1G52520, AT1G52430, AT1G52450, AT1G52700, AT1G52420, AT1G52670, AT1G52540, AT1G52720, AT1G52590, AT1G52600, AT1G78020, AT1G77940, AT1G78070, AT1G75020, AT1G74840, AT1G74990, AT1G74730, AT1G74860, AT1G74940, AT1G74950, AT1G74970, AT1G74920, AT1G74910, AT1G74960, AT1G74710, AT1G78050, AT1G78060, AT1G77890, AT1G77910, AT1G77810, AT1G78010, AT1G77840, AT1G77930, AT1G78040, AT1G53910, AT1G53830, AT1G53800, AT2G01070, AT2G01120, AT2G01110, AT2G01060, AT2G01100, AT2G01080, AT2G01090, AT1G74850, AT1G74800, AT1G74720, AT1G74690, AT1G74930, AT1G74790, AT1G74780, AT1G74770, AT1G74750, AT1G74740, AT1G75010, AT1G74700, AT1G74680, AT1G74900, AT1G53840, AT1G53903, AT1G53885, AT1G53780, AT1G53750, AT1G68690, AT1G68620, AT1G68560, AT1G68740, AT1G48310, AT1G48270, AT1G48350, AT1G48330, AT1G48320, AT1G48300, AT1G48230, AT1G48360, AT1G48260, AT1G48240, AT1G48410, AT1G48420, AT1G48370, AT1G48380, AT1G48280, AT1G53760, AT1G53880, AT1G53900, AT1G53920, AT1G53790, AT1G53860, AT1G53850, AT1G53890, AT1G53870, AT1G70850, AT1G70895, AT1G70782, AT1G70780, AT1G70940, AT1G42470, AT1G42460, AT1G42410, AT1G42370, AT1G42360, AT1G42440, AT1G68720, AT1G68710, AT1G68650, AT1G68640, AT1G68630, AT1G68580, AT1G68570, AT1G68610, AT1G68590, AT1G68670, AT1G68680, AT1G68585, AT1G68660, AT1G70760, AT1G70770, AT1G70790, AT1G27595, AT1G27610, AT1G27650, AT1G27630, AT1G27600, AT1G27530, AT1G27520, AT1G70880, AT1G70920, AT1G70890, AT1G70950, AT5G56680, AT1G70980, AT1G71000, AT1G71010, AT1G70830, AT1G70900, AT1G70810, AT1G70990, AT2G48120, AT2G48140, AT1G27580, AT1G27540, AT1G27570, AT1G27590, AT1G64190, AT1G64170, AT1G64160, AT1G64140, AT1G64142, AT1G64090, AT1G64070, AT1G64050, AT1G64220, AT1G64185, AT1G64000, AT1G64100, AT1G64105, AT1G64230, AT1G64150, AT1G64040, AT1G64060, AT1G64180, AT1G63980, AT1G64110, AT2G48160, AT2G48130, AT2G48150, AT1G72990, AT1G64210, AT1G64200, AT1G72820, AT1G73040, AT1G73080, AT1G73150, AT1G72840, AT1G72850, AT1G72860, AT1G72870, AT1G72890, AT1G73030, AT1G73060, AT1G73010, AT1G73090, AT1G73160, AT1G73130, AT1G73120, AT1G72910, AT1G72930, AT1G73100, AT1G72970, AT1G73110, AT1G72830, AT1G73020, AT1G72810, AT1G72900, AT1G72920, AT1G72940, AT1G72950, AT1G49370, AT1G49340, AT1G51040, AT1G49320, AT1G49260, AT1G49530, AT1G49520, AT1G49510, AT1G49540, AT1G49470, AT1G49380, AT1G49350, AT1G49500, AT1G49480, AT1G49300, AT1G49410, AT1G34670, AT1G34630, AT1G34750, AT1G34770, AT1G34640, AT1G34760, AT1G34780, AT1G49430, AT1G49400, AT1G49390, AT1G50170, AT1G50320, AT1G50380, AT1G50300, AT1G50290, AT1G23450, AT1G47640, AT1G47600, AT1G51470, AT1G47570, AT1G47550, AT1G47560, AT1G47530, AT1G47500, AT1G47490, AT1G47670, AT1G47620, AT1G47610, AT1G47410, AT1G47710, AT1G47720, AT1G47420, AT1G47655, AT1G47480, AT1G49310, AT1G49450, AT1G11160, AT1G11320, AT1G11130, AT1G11210, AT1G11240, AT1G11190, AT1G11310, AT1G11260, AT1G11200, AT1G11280, AT1G50400, AT1G06030, AT1G50390, AT1G50360, AT1G50350, AT1G50310, AT1G50240, AT1G50200, AT1G50220, AT1G50190, AT1G50250, AT1G50340, AT1G50370, AT1G11170, AT1G17080, AT1G17020, AT1G17220, AT1G21700, AT1G21730, AT1G21740, AT1G21610, AT1G21830, AT1G21840, AT1G21650, AT1G21630, AT1G21660, AT1G21780, AT1G21790, AT1G21720, AT1G21710, AT1G21770, AT1G21760, AT1G21690, AT1G21600, AT1G21670, AT1G21750, AT1G21680, AT1G21640, AT1G11330, AT1G11270, AT1G17145, AT1G34270, AT1G34190, AT1G34200, AT1G17190, AT1G17180, AT1G17170, AT1G17160, AT1G17150, AT1G17130, AT1G17120, AT1G17110, AT1G17070, AT1G17060, AT1G17050, AT1G17260, AT1G17250, AT1G17240, AT1G17210, AT1G17090, AT1G17040, AT1G17100, AT1G17280, AT5G50430, AT1G17140, AT1G17200, AT1G34260, AT1G34180, AT1G15420, AT1G31280, AT1G31290, AT1G31310, AT1G31350, AT1G31360, AT1G31330, AT1G31335, AT1G31300, AT1G34280, AT1G34340, AT1G34220, AT1G34210, AT1G34320, AT1G34300, AT1G15200, AT1G15430, AT1G15140, AT1G15390, AT1G15230, AT1G15210, AT1G15280, AT1G15290, AT1G15300, AT1G15330, AT1G15370, AT1G15410, AT1G15110, AT1G15440, AT1G15460, AT1G15480, AT1G15490, AT1G15130, AT1G15150, AT1G15160, AT1G15100, AT1G15220, AT1G15400, AT1G15120, AT1G15250, AT1G15080, AT1G15470, AT1G15350, AT1G15340, AT1G15310, AT1G15270, AT1G15380, AT1G15060, AT1G15170, AT1G13990, AT1G14120, AT1G13930, AT1G14140, AT1G14060, AT1G14150, AT1G13960, AT1G13980, AT1G13950, AT1G06620, AT1G06590, AT1G06560, AT1G06550, AT1G06540, AT1G06530, AT1G06510, AT1G06470, AT1G06450, AT1G06440, AT1G06430, AT1G06580, AT1G06490, AT1G06460, AT1G06520, AT1G06500, AT1G06680, AT1G06630, AT1G06690, AT1G06570, AT1G06670, AT1G06640, AT1G06650, AT1G62820, AT1G62730, AT1G62770, AT1G62710, AT1G62750, AT1G62800, AT1G14020, AT1G14030, AT1G14040, AT1G14090, AT1G14130, AT1G14190, AT1G14185, AT1G14200, AT1G14210, AT1G14220, AT1G14240, AT1G14000, AT1G14230, AT1G14250, AT1G13920, AT1G13940, AT1G13970, AT1G14070, AT1G14080, AT1G62810, AT1G62830, AT1G62850, AT1G76040, AT1G76050, AT1G76060, AT1G75910, AT1G75950, AT1G75860, AT1G75810, AT1G75830, AT1G75900, AT1G20260, AT1G76030, AT1G76010, AT1G75990, AT1G62680, AT1G62700, AT1G62740, AT1G62780, AT1G62790, AT1G75850, AT1G75940, AT1G75980, AT1G76020, AT1G16480, AT1G16220, AT1G16510, AT1G16260, AT1G16280, AT1G16290, AT1G16190, AT1G16240, AT1G16210, AT1G16500, AT1G16460, AT1G16520, AT1G07950, AT1G16430, AT1G16490, AT1G16470, AT1G16410, AT1G16400, AT1G43560, AT1G43620, AT1G43630, AT4G00540, AT1G43330, AT1G43580, AT1G75800, AT1G75820, AT1G75840, AT1G16390, AT1G16420, AT1G28660, AT1G28670, AT1G28640, AT1G28570, AT1G28560, AT1G28530, AT1G28760, AT1G29020, AT1G28680, AT1G28600, AT1G28960, AT1G28540, AT1G28610, AT1G28580, AT1G28710, AT1G16310, AT1G16330, AT1G16340, AT1G16350, AT1G16360, AT1G16370, AT1G10780, AT1G10800, AT1G10770, AT1G10720, AT1G10700, AT1G13160, AT1G13170, AT1G12990, AT1G13180, AT1G13190, AT1G13210, AT1G13220, AT1G13230, AT1G13000, AT1G13020, AT1G13030, AT1G13050, AT1G13070, AT1G13090, AT3G26340, AT1G13060, AT1G13195, AT1G10850, AT1G10760, AT1G10750, AT1G10740, AT1G10730, AT1G10690, AT1G10840, AT1G10830, AT1G13110, AT1G13120, AT1G13130, AT1G20850, AT1G20840, AT1G20980, AT1G20960, AT1G21010, AT1G20930, AT1G21000, AT1G20880, AT1G20900, AT1G20950, AT1G11740, AT1G11730, AT1G11720, AT1G11710, AT1G11700, AT1G11670, AT1G11660, AT1G11780, AT1G11770, AT1G11755, AT1G11600, AT1G11680, AT1G11800, AT1G11630, AT1G11750, AT1G11650, AT1G11790, AT1G13080, AT1G13100, AT1G14950, AT1G14970, AT1G14700, AT1G14730, AT1G14870, AT1G14880, AT1G14750, AT1G14660, AT1G14830, AT1G14960, AT1G14770, AT1G14900, AT1G14810, AT1G14720, AT1G14890, AT1G14740, AT1G14670, AT1G14685, AT1G14710, AT1G14920, AT1G20920, AT1G20910, AT1G20890, AT1G20870, AT1G14910, AT1G64790, AT1G64810, AT1G64910, AT1G64920, AT1G64960, AT1G64980, AT1G64990, AT4G27630, AT1G65010, AT1G64700, AT1G65020, AT1G64970, AT1G64750, AT1G64850, AT1G64770, AT1G64860, AT1G64880, AT1G64890, AT1G64780, AT1G64720, AT1G64740, AT1G14780, AT1G14790, AT1G14850, AT1G79460, AT1G79440, AT1G59790, AT1G59780, AT1G59820, AT1G59840, AT1G59870, AT1G59890, AT1G59910, AT1G59725, AT1G59730, AT1G59760, AT1G59900, AT1G59830, AT1G59710, AT1G59860, AT1G07400, AT1G59740, AT1G59960, AT1G59750, AT1G59940, AT1G59700, AT1G64800, AT1G65000, AT1G79380, AT1G79500, AT1G79430, AT1G79420, AT1G75660, AT1G75630, AT1G19910, AT1G65730, AT1G65720, AT1G65690, AT1G65700, AT1G65820, AT1G65840, AT1G16270, AT1G79570, AT1G79410, AT1G79560, AT1G79540, AT1G79530, AT1G16300, AT1G79520, AT1G79490, AT1G79450, AT1G79470, AT1G79550, AT1G79510, AT1G79390, AT1G75750, AT1G75560, AT1G75510, AT1G75500, AT1G75670, AT1G54500, AT1G54520, AT1G54270, AT1G54250, AT3G59600, AT1G54410, AT1G54290, AT1G54320, AT1G54490, AT1G54340, AT1G54220, AT1G54380, AT1G54310, AT1G75760, AT1G75730, AT1G75720, AT1G75710, AT1G75640, AT1G75620, AT1G75540, AT1G75520, AT1G75780, AT1G13370, AT1G75600, AT1G75610, AT1G75680, AT1G75590, AT1G54390, AT1G54550, AT1G54460, AT1G23290, AT1G23390, AT1G23240, AT1G23310, AT1G23260, AT1G54210, AT1G54230, AT1G54440, AT1G54470, AT1G54350, AT1G54360, AT1G54200, AT1G54450, AT1G54510, AT1G54540, AT1G54260, AT1G54370, AT1G23330, AT1G23380, AT1G23400, AT1G23440, AT1G23410, AT2G17620, AT1G23870, AT1G23880, AT1G23940, AT1G23900, AT1G23960, AT1G23980, AT1G24030, AT1G24050, AT1G24070, AT1G23850, AT1G23970, AT1G23950, AT1G23860, AT1G23890, AT1G23230, AT1G23280, AT1G23340, AT1G23360, AT2G05380, AT2G17630, AT4G13500, AT2G05310, AT2G05260, AT2G05170, AT2G13430, AT2G13440, AT2G04620, AT2G07660, AT2G07773, ATMG00910, AT2G18190, AT2G18180, AT2G18150, AT2G18140, AT2G18162, AT2G18160, AT2G18170, AT2G17540, AT2G17550, AT2G17580, AT2G17530, AT2G17500, AT2G17560, AT2G17570, AT2G17520, AT2G17510, AT2G05350, AT2G27200, AT2G27190, AT2G16090, AT2G16110, AT2G16000, AT2G16210, AT2G16020, AT2G16060, AT2G16070, AT2G16005, AT2G05200, AT2G05210, AT2G05250, AT2G05230, AT2G05270, AT2G05120, AT2G05320, AT2G05160, AT2G05180, AT1G65180, AT1G65220, AT1G03080, AT1G03150, AT1G03130, AT1G03055, AT1G54590, AT1G03140, AT1G03040, AT1G03090, AT1G03110, AT1G78490, AT1G78530, AT1G78510, AT1G78480, AT1G78460, AT1G78610, AT1G78600, AT1G78620, AT1G78650, AT1G78630, AT1G78560, AT1G78570, AT1G78550, AT1G78580, AT1G78660, AT1G03100, AT1G54090, AT1G53980, AT1G53970, AT1G54050, AT1G54120, AT1G54060, AT1G54010, AT1G54000, AT1G54110, AT1G54140, AT1G54030, AT1G54100, AT1G54130, AT1G54080, AT1G54150, AT1G03160, AT1G03070, AT1G03060, AT1G03030, AT1G03010, AT1G03000, AT1G54115, AT1G54170, AT1G54180, AT1G05530, AT1G05710, AT1G05620, AT1G05720, AT1G05575, AT1G05570, AT1G05560, AT1G05520, AT1G36160, AT1G36050, AT1G36060, AT1G36070, AT1G53990, AT1G05590, AT1G05600, AT1G05610, AT1G05630, AT1G05490, AT1G05500, AT1G10590, AT1G10550, AT1G10480, AT1G10522, AT1G10460, AT1G10510, AT1G30660, AT1G30720, AT1G30730, AT1G30570, AT1G30600, AT1G30610, AT1G30620, AT1G30640, AT1G30630, AT1G30580, AT1G30650, AT1G30690, AT1G30750, AT1G30700, AT1G05660, AT1G05650, AT1G05670, AT1G05680, AT1G05700, AT1G10430, AT1G10470, AT1G10610, AT2G16580, AT2G16570, AT2G16530, AT1G72590, AT2G16500, AT2G31400, AT2G31350, AT2G31470, AT2G31440, AT2G31450, AT2G31360, AT2G31390, AT2G31410, AT2G31380, AT2G31370, AT1G10490, AT1G10500, AT1G10540, AT1G10560, AT1G10570, AT2G38830, AT2G38840, AT2G38810, AT2G38820, AT2G16510, AT2G11590, AT2G11520, AT2G14170, AT2G14100, AT2G14110, AT2G14120, AT2G14130, AT2G14160, AT2G14095, AT2G36090, AT2G36100, AT2G36120, AT2G36160, AT2G36130, AT2G36170, AT2G10930, AT2G10870, AT2G14210, AT2G38800, AT2G15310, AT2G15290, AT2G11570, AT2G11600, AT2G15240, AT2G01930, AT2G12480, AT2G05760, AT2G05755, AT2G24710, AT2G24720, AT2G24740, AT1G17770, AT2G24762, AT2G47160, AT2G47180, AT2G47170, AT2G04240, AT2G04280, AT2G15300, AT2G15280, AT2G15320, AT2G15270, AT2G03870, AT2G03890, AT2G03820, AT2G06440, AT2G04940, AT2G05070, AT2G05100, AT2G05720, AT2G05710, AT2G13370, AT2G13360, AT2G22120, AT2G22080, AT2G22070, AT2G22060, AT2G22100, AT2G22090, AT3G32901, AT1G32820, AT4G03980, AT3G30455, AT2G04950, AT2G14150, AT2G06450, AT2G15230, AT2G03830, AT2G03840, AT2G20550, AT2G20480, AT2G20500, AT2G20490, AT2G20450, AT2G20515, AT2G20560, AT4G28510, AT2G20530, AT2G40180, AT2G40140, AT2G40200, AT2G40230, AT2G40120, AT2G40130, AT2G40150, AT2G40160, AT2G11830, AT2G11810, AT2G31710, AT2G31540, AT2G31550, AT2G31680, AT2G31490, AT3G53870, AT2G31610, AT2G04070, AT2G04050, AT2G04040, AT2G04090, AT2G04100, AT2G04080, AT2G04066, AT2G04160, AT2G04038, AT2G28710, AT2G28720, AT2G21240, AT2G21230, AT2G17250, AT2G17220, AT2G17240, AT2G17230, AT2G17190, AT2G17200, AT2G31510, AT2G31530, AT2G31570, AT2G22260, AT2G22310, AT2G22250, AT2G22270, AT2G22170, AT2G22230, AT2G22240, AT2G28600, AT2G28690, AT2G28670, AT2G28660, AT2G28650, AT2G28620, AT2G28605, AT2G28630, AT2G31650, AT1G05830, AT2G31660, AT2G31670, AT2G22190, AT2G22125, AT2G22300, AT2G31820, AT2G31810, AT2G31800, AT2G31940, AT2G31740, AT2G31730, AT2G31900, AT2G31890, AT2G31955, AT2G31750, AT2G31725, AT2G31945, AT2G31870, AT2G31790, AT2G31880, AT2G04032, AT2G04025, AT2G03980, AT2G04030, AT2G29900, AT2G29890, AT2G31840, AT2G31830, AT2G42620, AT2G42600, AT2G42520, AT2G42590, AT2G42530, AT2G42570, AT2G42540, AT2G42580, AT2G07675, ATMG00980, AT2G07676, ATMG00970, ATMG00920, AT2G07678, AT2G07685, ATMG00770, ATMG00730, AT2G07687, AT2G12400, AT2G21640, AT2G21600, AT2G21620, AT2G21580, AT2G42640, AT2G24960, AT2G24830, AT2G24800, AT2G24765, AT5G37350, AT2G24990, AT2G24820, AT2G24940, AT2G24945, AT2G24970, AT2G25000, AT2G24790, AT2G25010, AT2G24850, AT2G24860, AT2G21630, AT2G21610, AT2G21590, AT2G21560, AT2G21550, AT2G21660, AT2G17033, AT2G17110, AT2G24980, AT2G16430, AT2G16460, AT2G16365, AT2G16400, AT2G16380, AT2G15380, AT2G15350, AT2G15370, AT2G15410, AT2G15400, AT2G15430, AT2G15390, AT2G15340, AT2G15440, AT2G27170, AT2G27150, AT2G17050, AT2G17060, AT2G17150, AT2G16990, AT2G17070, AT2G17080, AT2G17020, AT2G17030, AT2G17140, AT2G17120, AT2G17130, AT2G25210, AT2G25350, AT2G25340, AT2G25330, AT2G25320, AT2G25280, AT2G01820, AT2G01910, AT2G01840, AT2G01860, AT2G01880, AT2G01890, AT2G01900, AT2G01870, AT2G01850, AT2G01830, AT2G16390, AT4G34570, AT2G16370, AT2G16360, AT2G16340, AT2G06610, AT2G05480, AT5G28487, AT2G06690, AT1G45080, AT2G12140, AT2G16330, AT3G42620, AT2G16485, AT2G16280, AT2G16270, AT2G16260, AT2G16250, AT2G16230, AT2G16440, AT2G25250, AT2G25240, AT2G04680, AT2G04670, AT2G04660, AT2G04650, AT2G04630, AT2G04700, AT2G04920, AT2G04880, AT2G04780, AT2G04850, AT2G04845, AT2G04900, AT2G04795, AT2G04890, AT2G11910, AT2G11890, AT2G25310, AT2G25300, AT2G25270, AT2G04750, AT2G04740, AT2G04690, AT1G04390, AT1G04360, AT1G04340, AT1G04310, AT1G04300, AT1G04500, AT1G04240, AT1G04440, AT1G04490, AT1G04260, AT1G04290, AT1G04430, AT1G04410, AT1G04250, AT1G04480, AT1G04510, AT1G04270, AT1G04350, AT1G04400, AT2G04800, AT1G09340, AT1G04520, AT1G59990, AT1G26930, AT1G26840, AT1G26870, AT1G26900, AT1G26830, AT1G26910, AT1G26940, AT1G26920, AT1G26820, AT1G26960, AT1G26880, AT1G26850, AT1G31200, AT1G31210, AT3G60565, AT1G31220, AT1G31230, AT1G31120, AT1G31150, AT1G31160, AT1G31240, AT1G31170, AT1G31130, AT1G31190, AT1G31180, AT5G14200, AT1G09300, AT1G09320, AT1G09310, AT1G09330, AT2G20630, AT2G20585, AT2G20610, AT2G20580, AT2G13560, AT2G13600, AT2G13650, AT2G13510, AT2G13610, AT2G13540, AT1G60070, AT1G60080, AT1G60090, AT1G59970, AT1G60030, AT1G59810, AT1G60040, AT1G60000, AT1G60010, AT2G21320, AT2G20625, AT2G21390, AT2G21420, AT2G21450, AT2G21470, AT2G21480, AT2G21520, AT2G21300, AT2G21340, AT2G21270, AT2G21430, AT2G21260, AT2G21250, AT2G21290, AT2G21330, AT2G21380, AT2G21385, AT2G21410, AT2G21540, AT2G21440, AT2G06390, AT2G06255, AT2G40280, AT2G46410, AT2G46440, AT2G46430, AT2G46450, AT2G46470, AT2G46340, AT2G46380, AT2G46400, AT2G46390, AT2G46370, AT2G46420, AT2G24580, AT2G24560, AT2G24530, AT2G24520, AT2G24630, AT2G24610, AT2G24540, AT2G24570, AT2G24640, AT2G24550, AT2G24600, AT2G40430, AT2G40270, AT2G40400, AT2G04305, AT2G04400, AT2G04340, AT2G04520, AT2G04350, AT2G10070, AT5G18380, AT2G09990, AT2G40240, AT2G40340, AT2G40350, AT2G40360, AT2G40370, AT2G40410, AT2G40420, AT2G40435, AT2G40260, AT2G40300, AT2G40316, AT2G40290, AT2G40330, AT2G04500, AT2G36880, AT2G36900, AT2G36885, AT2G36870, AT2G36835, AT2G37020, AT2G36930, AT2G37040, AT2G36990, AT2G36970, AT2G04490, AT2G04480, AT2G04450, AT2G04430, AT2G04410, AT2G04360, AT2G04560, AT2G04550, AT2G04540, AT2G04530, AT2G36910, AT2G36950, AT2G36830, AT2G36840, AT2G22000, AT2G22010, AT2G21860, AT2G21870, AT2G21970, AT2G21880, AT2G21780, AT3G18140, AT2G22040, AT2G21960, AT2G21820, AT2G21790, AT2G21830, AT2G36940, AT2G36960, AT2G36980, AT2G36850, AT2G36890, AT2G21910, AT2G21930, AT2G21940, AT2G21950, AT2G36290, AT2G36320, AT2G36230, AT2G36380, AT2G36250, AT2G36270, AT2G36480, AT2G36490, AT2G36390, AT2G36570, AT2G36400, AT2G36430, AT2G36590, AT2G36470, AT2G36410, AT2G36460, AT2G36580, AT3G52990, AT2G36485, AT2G36530, AT2G21800, AT2G21890, AT2G21730, AT2G36330, AT2G36310, AT2G36300, AT2G36220, AT2G35990, AT2G35950, AT2G35930, AT2G35920, AT2G35910, AT2G35900, AT2G36070, AT2G35860, AT2G35800, AT2G35980, AT2G36060, AT2G35960, AT2G35830, AT2G35810, AT2G35840, AT2G36010, AT2G35940, AT2G35880, AT2G36240, AT2G36210, AT2G36200, AT2G36370, AT2G36350, AT2G36080, AT1G36270, AT3G30790, AT1G42510, AT2G13000, AT2G42760, AT2G42740, AT2G42720, AT2G42710, AT2G42700, AT2G42660, AT2G42680, AT2G42840, AT2G42770, AT2G42870, AT2G42780, AT2G42670, AT2G42750, AT2G42790, AT2G42690, AT2G42880, AT2G42810, AT2G22530, AT2G22510, AT2G22370, AT2G22500, AT2G22420, AT2G22360, AT2G22475, AT2G22425, AT2G22470, AT2G22430, AT2G21120, AT2G21100, AT2G21090, AT2G21080, AT2G21070, AT4G38790, AT2G21190, AT2G21170, AT2G21130, AT2G21160, AT2G21185, AT2G21195, AT2G21180, AT2G21050, AT2G21060, AT2G03670, AT2G03680, AT2G03720, AT2G03780, AT2G03800, AT2G03580, AT2G03600, AT2G03590, AT2G03750, AT2G03690, AT2G03730, AT2G03760, AT2G22490, AT2G22480, AT2G22450, AT2G22410, AT2G22400, AT2G22390, AT2G22330, AT2G22560, AT2G22540, AT2G28590, AT2G28510, AT2G28550, AT2G31305, AT2G27980, AT2G27970, AT2G27950, AT2G27900, AT2G28000, AT2G27960, AT2G27920, AT2G28540, AT2G28470, AT2G28520, AT2G28570, AT2G31230, AT2G31240, AT2G31190, AT2G31340, AT2G31200, AT1G79370, AT1G79110, AT1G79040, AT1G79220, AT1G79200, AT1G79230, AT1G16700, AT1G79010, AT1G79260, AT1G79050, AT1G79000, AT1G79270, AT2G13660, AT2G13790, AT2G13800, AT2G13720, AT2G13690, AT2G31260, AT2G31270, AT2G31290, AT2G31320, AT1G79150, AT1G79180, AT1G79340, AT1G02280, AT1G02130, AT1G02270, AT1G79360, AT1G79350, AT1G79280, AT1G79190, AT1G79160, AT1G79080, AT1G79070, AT1G79030, AT1G78960, AT1G78950, AT1G78940, AT1G79210, AT1G79090, AT1G78930, AT1G79330, AT1G79320, AT1G79310, AT1G02220, AT1G02250, AT1G02090, AT1G02140, AT1G65430, AT1G65440, AT1G65280, AT1G65310, AT1G65260, AT1G65270, AT1G65420, AT1G65290, AT1G65295, AT1G65410, AT1G65370, AT1G02060, AT1G02080, AT1G02020, AT1G02260, AT1G02100, AT1G02120, AT1G02160, AT1G02050, AT1G02110, AT1G02150, AT1G02170, AT1G02180, AT1G65350, AT1G65360, AT1G65380, AT1G54790, AT1G54780, AT1G54570, AT1G54690, AT1G54580, AT1G54630, AT1G54830, AT1G54730, AT1G54710, AT1G54610, AT1G22720, AT1G22690, AT1G22670, AT1G22620, AT1G22800, AT1G22700, AT1G22650, AT1G22780, AT1G22710, AT1G22790, AT1G22730, AT1G22750, AT1G22740, AT1G22640, AT1G22770, AT1G22660, AT1G65390, AT1G60190, AT1G60200, AT1G60420, AT1G60170, AT3G60610, AT3G16030, AT1G67440, AT1G67420, AT1G67510, AT1G67500, AT1G67490, AT1G67480, AT1G67460, AT1G67470, AT1G67430, AT1G54560, AT1G54680, AT1G54740, AT1G54770, AT1G54840, AT1G54650, AT1G60440, AT1G60140, AT1G60160, AT1G78700, AT1G78920, AT1G78680, AT1G09180, AT1G09170, AT1G09150, AT1G09140, AT1G09270, AT1G09230, AT1G09220, AT1G09290, AT1G09210, AT1G09240, AT1G09200, AT1G09250, AT1G09280, AT1G09160, AT1G60220, AT1G60270, AT1G60380, AT1G60390, AT1G60130, AT1G78820, AT1G78830, AT1G61820, AT1G61830, AT1G61840, AT1G61850, AT1G61860, AT1G62040, AT1G61870, AT1G61930, AT1G62045, AT1G61890, AT1G78810, AT1G78800, AT1G78790, AT1G78770, AT1G78750, AT1G78720, AT1G78690, AT1G78850, AT1G78860, AT1G78670, AT1G78780, AT1G78900, AT1G16890, AT1G78870, AT1G78895, AT1G78815, AT1G78890, AT1G61900, AT1G61990, AT1G62020, AT1G70450, AT1G70410, AT1G70420, AT1G70370, AT1G70330, AT1G70310, AT1G04220, AT1G04110, AT1G04090, AT1G04200, AT1G04190, AT1G04180, AT1G04160, AT1G04150, AT1G04230, AT1G04170, AT1G04100, AT1G04140, AT1G04120, AT1G04130, AT1G70320, AT1G55860, AT1G70360, AT1G70350, AT1G70300, AT1G70290, AT1G70280, AT1G70270, AT1G12080, AT1G11910, AT1G12010, AT1G12040, AT1G12110, AT1G11930, AT1G11870, AT1G03370, AT1G03270, AT1G03260, AT1G03200, AT1G03240, AT1G03210, AT1G03190, AT1G03360, AT1G03180, AT1G03310, AT1G03300, AT1G03290, AT1G03330, AT1G03220, AT1G03230, AT1G03250, AT1G03350, AT1G03280, AT1G70430, AT1G12100, AT1G12090, AT1G11840, AT1G11890, AT2G25180, AT2G25090, AT2G25070, AT2G25060, AT2G25200, AT2G25190, AT2G25100, AT2G25110, AT2G25080, AT2G25170, AT1G12020, AT1G12000, AT1G11960, AT1G11950, AT1G11900, AT1G11880, AT1G11860, AT1G12070, AT1G12050, AT1G61730, AT1G61780, AT2G25140, AT2G25150, AT2G25160, AT1G10210, AT1G10290, AT1G10180, AT1G10240, AT1G43205, AT4G07523, AT1G43230, AT5G27180, AT1G43160, AT1G43140, AT1G43190, AT5G33320, AT1G43310, AT1G43170, AT1G43130, AT1G61690, AT1G61740, AT1G61750, AT1G61760, AT1G61770, AT1G61790, AT1G61710, AT1G61810, AT1G10340, AT1G10360, AT1G10370, AT1G27510, AT1G27400, AT1G27450, AT1G27340, AT1G27435, AT1G27385, AT1G27360, AT1G27290, AT1G27310, AT1G27300, AT1G27320, AT1G27460, AT1G10450, AT1G10250, AT1G10270, AT1G10300, AT1G10320, AT1G10330, AT1G10390, AT1G10400, AT1G10410, AT1G10160, AT1G10170, AT1G10190, AT4G15050, AT1G10200, AT1G10150, AT1G10350, AT1G10230, AT1G10380, AT1G10140, AT1G10310, AT1G67100, AT1G67110, AT1G67120, AT1G67140, AT1G67180, AT5G38430, AT5G38410, AT1G67090, AT5G38420, AT1G77150, AT1G77170, AT1G77130, AT1G77240, AT1G77270, AT1G77280, AT1G77300, AT1G77210, AT1G77230, AT1G77260, AT1G77220, AT1G77180, AT1G77140, AT1G27330, AT1G27350, AT1G27370, AT1G27390, AT1G27410, AT1G27440, AT1G27380, AT1G30840, AT1G30825, AT1G09390, AT1G09430, AT1G09380, AT1G09560, AT1G09415, AT1G09570, AT1G09520, AT1G09350, AT1G09575, AT1G09420, AT1G10090, AT1G10120, AT1G09980, AT1G10000, AT1G10020, AT1G10050, AT1G10030, AT1G10070, AT1G10130, AT1G30760, AT1G30810, AT1G30820, AT1G55740, AT1G55720, AT1G55730, AT1G55700, AT1G55690, AT1G55610, AT1G55590, AT1G55630, AT1G55830, AT1G55780, AT1G55800, AT1G55790, AT1G55670, AT1G55805, AT1G55620, AT1G55680, AT1G09440, AT1G09450, AT1G09460, AT1G09480, AT1G09490, AT1G09510, AT1G09600, AT1G55820, AT1G55810, AT1G55760, AT1G55750, AT3G61420, AT1G05280, AT1G05270, AT1G05250, AT1G05240, AT1G05150, AT1G05350, AT1G05310, AT1G05300, AT1G05190, AT1G05360, AT1G05260, AT1G05205, AT1G05340, AT1G05210, AT1G05170, AT1G05140, AT1G05180, AT1G05160, AT1G05200, AT2G17730, AT2G17710, AT2G17670, AT2G17720, AT2G17800, AT2G17640, AT1G04750, AT1G04620, AT1G04610, AT1G04600, AT1G04730, AT1G04700, AT1G04670, AT1G04650, AT1G04550, AT1G04690, AT1G04710, AT1G04530, AT1G04680, AT1G04560, AT1G04640, AT1G04590, AT2G17740, AT2G17660, AT2G17680, AT2G17760, AT2G17700, AT2G17790, AT1G09000, AT1G08930, AT1G09020, AT1G65520, AT1G65540, AT1G65470, AT1G65630, AT1G65660, AT1G65670, AT1G65500, AT1G65490, AT1G65560, AT1G65650, AT1G65680, AT1G09130, AT1G09030, AT1G08990, AT1G08960, AT1G08940, AT1G09090, AT1G09060, AT1G08970, AT1G08880, AT1G08920, AT1G08980, AT1G08900, AT1G08890, AT1G09070, AT1G09010, AT1G09100, AT1G09910, AT1G09940, AT1G09950, AT1G09960, AT1G09970, AT1G09660, AT1G09680, AT1G58350, AT1G09780, AT1G09630, AT1G09640, AT1G57720, AT1G09920, AT1G09750, AT1G09795, AT1G09815, AT1G09830, AT1G09870, AT1G09700, AT1G09760, AT1G09690, AT1G09590, AT1G65510, AT1G65570, AT1G65580, AT1G65590, AT1G65610, AT1G09840, AT1G09850, AT1G09900, AT1G70060, AT1G70000, AT1G70250, AT1G70230, AT1G70210, AT1G70200, AT1G69980, AT1G70100, AT1G70160, AT1G70190, AT1G69960, AT1G70090, AT1G09620, AT1G09720, AT1G09730, AT1G09740, AT1G09770, AT1G09790, AT1G09800, AT1G09810, AT1G09820, AT1G69990, AT1G70180, AT1G70170, AT1G70140, AT1G70130, AT1G23000, AT1G22970, AT1G22960, AT1G22920, AT1G22885, AT1G22985, AT1G22840, AT1G22850, AT1G22990, AT1G62085, AT1G62150, AT1G62170, AT1G62280, AT1G62200, AT1G62250, AT1G62260, AT1G62130, AT1G62180, AT1G62300, AT1G70070, AT1G22830, AT1G22870, AT1G23020, AT1G23010, AT1G61490, AT1G61400, AT1G61370, AT1G61360, AT1G61340, AT1G61480, AT1G61280, AT1G61460, AT1G61450, AT1G61440, AT1G61430, AT1G61420, AT1G61380, AT1G61350, AT1G23030, AT1G22940, AT1G22930, AT1G22900, AT1G22890, AT1G22880, AT1G22860, AT1G08780, AT1G08570, AT1G08720, AT1G08540, AT1G08810, AT1G08650, AT2G17830, AT5G42460, AT2G17840, AT2G17880, AT2G17870, AT2G17820, AT1G08620, AT1G08660, AT1G08670, AT1G08680, AT1G08710, AT1G08730, AT1G08550, AT1G08800, AT1G08840, AT1G08560, AT1G08580, AT1G08590, AT1G08600, AT1G08610, AT1G08700, AT1G08770, AT1G08830, AT1G08750, AT1G08640, AT2G17890, AT2G17900, AT1G03620, AT1G03560, AT1G03530, AT1G03510, AT1G03475, AT1G03470, AT1G03457, AT1G03420, AT1G03410, AT1G03380, AT1G03687, AT1G03660, AT1G03590, AT1G03730, AT1G03550, AT1G03610, AT1G03600, AT1G03430, AT1G03630, AT1G03440, AT1G03740, AT1G03700, AT1G03400, AT1G03520, AT1G03680, AT2G17850, AT2G17380, AT2G17350, AT2G17360, AT2G17340, AT2G17290, AT2G17480, AT2G17260, AT2G17450, AT2G17265, AT2G17370, AT1G24170, AT1G24190, AT1G24280, AT1G24320, AT1G24330, AT1G24310, AT1G24120, AT1G24140, AT1G24150, AT1G24090, AT1G24350, AT1G24180, AT1G24100, AT1G24240, AT2G17390, AT2G17410, AT1G61260, AT3G01760, AT1G61270, AT1G61110, AT1G61250, AT1G11180, AT1G61170, AT1G61190, AT1G61180, AT1G61300, AT1G61310, AT1G23120, AT1G23180, AT1G23140, AT1G23040, AT1G23100, AT1G23220, AT1G23205, AT1G23080, AT1G23090, AT1G23060, AT1G23190, AT4G35460, AT2G17420, AT2G17430, AT2G17270, AT2G17280, AT2G17440, AT2G17300, AT1G61100, AT1G60690, AT1G60750, AT1G60770, AT1G60780, AT1G60810, AT1G60550, AT1G60650, AT1G60830, AT1G60900, AT1G65970, AT1G60740, AT1G60620, AT1G60670, AT1G60660, AT1G60490, AT1G60710, AT1G60730, AT1G60800, AT1G61240, AT1G61150, AT1G61140, AT1G60610, AT1G60630, AT1G60470, AT1G60680, AT1G67340, AT1G76940, AT1G77080, AT1G77010, AT1G77020, AT1G77030, AT1G76970, AT1G76900, AT1G77120, AT1G77060, AT1G76920, AT1G76990, AT1G77000, AT1G77090, AT1G76930, AT1G76950, AT1G60600, AT1G60460, AT1G60560, AT1G60570, AT1G67250, AT1G67360, AT1G67320, AT1G67280, AT1G67370, AT1G27050, AT1G27070, AT1G27080, AT1G27090, AT1G27120, AT1G27170, AT1G27180, AT1G27190, AT1G27240, AT1G27000, AT1G27150, AT1G27130, AT1G27030, AT1G27140, AT1G27200, AT1G27210, AT1G67300, AT1G67290, AT1G67230, AT1G67330, AT1G67310, AT1G67410, AT1G67350, AT1G26970, AT1G61667, AT1G61570, AT1G61560, AT1G24510, AT1G24490, AT1G24460, AT1G24430, AT1G24420, AT2G13770, AT1G24370, AT1G24610, AT1G24440, AT1G24600, AT1G24575, AT1G24360, AT1G24450, AT1G24530, AT1G24625, AT1G61590, AT1G61580, AT1G61520, AT1G61660, AT1G61620, AT1G04080, AT1G03860, AT1G03920, AT1G04000, AT1G03970, AT1G04070, AT1G04040, AT1G03900, AT1G03950, AT5G44100, AT1G03930, AT1G52050, AT1G52060, AT1G52070, AT2G05570, AT1G52010, AT3G32397, AT3G43360, AT1G51980, AT1G52140, AT1G52080, AT1G52150, AT1G61640, AT1G61550, AT1G61500, AT1G61630, AT1G03980, AT1G03870, AT1G03850, AT1G55545, AT1G55510, AT1G55520, AT1G55580, AT1G55480, AT1G55540, AT1G55450, AT1G55490, AT1G55530, AT1G55500, AT1G55460, AT1G03840, AT1G03830, AT1G03820, AT1G03780, AT1G03770, AT1G03750, AT1G03960, AT1G03910, AT1G03905, AT1G04030, AT1G04020, AT1G04010, AT1G31010, AT1G30880, AT1G30870, AT1G31070, AT1G31020, AT1G63340, AT1G62580, AT1G62590, AT1G63330, AT1G63380, AT1G62610, AT1G62620, AT1G63370, AT1G62510, AT4G09455, AT1G62460, AT1G62440, AT1G62450, AT1G62480, AT1G62500, AT1G62660, AT1G62570, AT1G62640, AT1G62560, AT1G62540, AT1G55430, AT1G55380, AT1G55420, AT1G30970, AT1G30890, AT1G23760, AT1G23830, AT1G51260, AT1G51270, AT1G51300, AT1G51310, AT1G51350, AT1G51360, AT1G51170, AT1G51190, AT1G51210, AT1G51380, AT1G51160, AT1G51400, AT1G51200, AT1G51340, AT1G30940, AT1G30935, AT1G30960, AT1G30990, AT1G31000, AT1G31040, AT1G31060, AT1G31050, AT1G30900, AT1G30910, AT1G23700, AT1G23720, AT1G23820, AT1G23530, AT1G23470, AT1G23460, AT1G23480, AT1G23740, AT1G23750, AT1G23710, AT1G23670, AT1G23560, AT1G23490, AT1G23800, AT1G23840, AT1G23780, AT1G05120, AT1G05060, AT1G05070, AT1G05010, AT2G36730, AT2G36740, AT2G36810, AT2G36650, AT2G36660, AT2G36670, AT2G36680, AT2G36690, AT2G36620, AT1G05030, AT1G05020, AT1G05000, AT1G05100, AT1G05090, AT4G20720, AT1G05055, AT2G02050, AT2G02040, AT2G02000, AT2G02010, AT2G01970, AT2G36720, AT2G28430, ATMG01280, AT2G07695, ATMG01190, AT2G07698, ATMG00430, ATMG01150, AT2G07666, AT2G07701, ATMG00480, AT2G07707, AT2G07715, ATMG00560, ATMG00590, AT2G07718, AT2G07721, AT2G07719, ATMG00610, AT2G07706, ATMG00470, AT2G07696, ATMG01270, ATMG01300, AT2G07692, AT2G07691, ATMG01310, AT2G07705, AT2G43040, AT2G43018, AT2G43020, AT2G43060, AT2G43050, AT2G43030, AT2G43010, AT2G01940, AT2G01950, AT2G01960, AT2G01980, AT2G02020, AT2G28380, AT2G28390, AT2G20420, AT2G20390, AT2G20330, AT2G43000, AT2G42980, AT2G42900, AT2G42890, AT2G42960, AT2G42950, AT2G42910, AT2G28360, AT2G28350, AT2G28320, AT2G28450, AT2G28400, AT2G28410, AT2G28330, AT2G28460, AT2G28370, AT2G20370, AT2G20230, AT2G20410, AT2G20360, AT2G20260, AT2G20270, AT2G22690, AT2G22590, AT2G22720, AT2G14080, AT2G13940, AT2G13960, AT5G02320, AT2G13970, AT2G20340, AT2G20320, AT2G20310, AT2G20300, AT2G20290, AT2G20280, AT2G20240, AT2G20210, AT2G20400, AT2G22650, AT2G22670, AT2G18280, AT2G18270, AT2G18250, AT2G18240, AT2G18220, AT2G18210, AT2G18200, AT2G18450, AT2G18440, AT2G18410, AT2G18350, AT2G18370, AT2G18245, AT2G18290, AT2G18390, AT2G18400, AT2G18230, AT2G18330, AT2G18360, AT2G22570, AT2G22660, AT2G22680, AT2G22740, AT2G22610, AT2G22620, AT2G22630, AT2G16600, AT2G16800, AT2G16700, AT2G16760, AT2G16595, AT2G16740, AT2G16710, AT2G16720, AT2G13330, AT2G13290, AT2G01029, AT2G10770, AT3G30415, AT2G06460, AT2G10790, AT2G06480, AT2G06510, AT2G06520, AT2G06530, AT2G05810, AT2G05790, AT2G18380, AT2G16790, AT2G16780, AT2G20760, AT2G20900, AT2G20930, AT2G20670, AT2G21010, AT2G20990, AT2G20690, AT2G20820, AT2G20810, AT2G20860, AT2G20830, AT2G20725, AT2G20850, AT2G11000, AT2G10950, AT2G16750, AT2G16810, AT2G16630, AT2G16640, AT2G16650, AT5G60430, AT2G16660, AT2G20890, AT2G37120, AT2G20920, AT2G20840, AT1G31880, AT2G21030, AT2G20616, AT2G20815, AT2G20790, AT2G20780, AT2G20770, AT2G20740, AT2G20710, AT2G20680, AT2G20650, AT2G20950, AT1G31885, AT2G21020, AT2G20980, AT2G21045, AT2G20960, AT2G20870, AT2G20940, AT2G20750, AT2G37170, AT2G37180, AT2G37190, AT2G37110, AT2G46570, AT2G46590, AT2G46610, AT2G46620, AT2G46490, AT2G46510, AT2G46520, AT2G46530, AT2G46560, AT2G46505, AT2G46550, AT2G46580, AT2G46540, AT2G46600, AT2G46500, AT2G37090, AT2G37080, AT2G37050, AT2G37160, AT2G37150, AT2G37130, AT2G15580, AT2G15690, AT2G15620, AT2G15570, AT2G15890, AT2G15760, AT2G15970, AT2G15960, AT2G15790, AT2G15820, AT2G15450, AT2G15470, AT2G15460, AT2G15670, AT2G15680, AT2G15695, AT2G15720, AT2G15480, AT2G15490, AT2G15500, AT2G15520, AT2G15530, AT2G05630, AT2G05540, AT2G05590, AT2G06090, AT2G06040, AT2G06010, AT2G06210, AT2G06160, AT2G06050, AT2G11370, AT2G11390, AT2G15900, AT2G15880, AT2G15860, AT2G15830, AT2G28290, AT2G28270, AT2G28260, AT2G28250, AT2G28305, AT2G05530, AT2G05520, AT2G05510, AT2G05440, AT2G12120, AT5G28482, AT2G05470, AT3G24370, AT1G34720, AT1G27800, AT5G35020, AT5G36736, AT5G35650, AT3G32394, AT3G43380, AT2G05567, AT4G05300, AT3G42750, AT5G36840, AT4G07944, AT2G14777, AT2G05620, AT2G28310, AT2G28230, AT2G28020, AT2G28200, AT2G14260, AT2G20190, AT2G20100, AT2G20050, AT2G20030, AT2G20010, AT2G20180, AT2G20000, AT2G19990, AT2G19970, AT2G20110, AT2G20120, AT2G20130, AT2G20060, AT2G20140, AT2G20020, AT2G20142, AT2G14230, AT2G25540, AT2G25380, AT2G25420, AT2G25430, AT2G25510, AT2G25560, AT2G25355, AT2G25450, AT2G25500, AT2G25520, AT2G25460, AT2G27320, AT2G27310, AT2G27260, AT2G27285, AT2G27290, AT2G27228, AT2G27230, AT2G27350, AT2G25490, AT2G25530, AT2G27480, AT2G27490, AT2G27375, AT2G27330, AT2G27450, AT2G27390, AT2G27370, AT2G27360, AT2G27500, AT2G27510, AT2G27460, AT2G13890, AT2G13920, AT2G13820, AT2G13840, AT2G13810, AT2G25440, AT2G24360, AT2G24500, AT2G24290, AT2G24420, AT2G27440, AT2G27470, AT2G32020, AT2G32040, AT2G32190, AT2G32210, AT2G32060, AT2G31980, AT2G32090, AT2G32120, AT2G32070, AT2G32080, AT2G31970, AT2G32235, AT2G32240, AT2G32150, AT2G24390, AT2G24400, AT2G24430, AT2G24300, AT2G24310, AT2G24320, AT2G24330, AT2G24440, AT2G24490, AT2G32170, AT2G32160, AT2G03470, AT2G03450, AT2G03430, AT2G03420, AT2G03390, AT2G03360, AT3G11810, AT2G03330, AT2G03530, AT2G03480, AT1G13860, AT2G03350, AT2G03510, AT2G03340, AT2G03500, AT2G40110, AT2G40090, AT2G40100, AT2G32140, AT2G32100, AT2G32030, AT2G32010, AT2G32000, AT2G31990, AT2G31960, AT2G32220, AT2G01130, AT2G01300, AT2G01190, AT2G01260, AT2G01140, AT2G01250, AT2G01180, AT2G01350, AT2G01270, AT2G01150, AT2G01320, AT2G01290, AT2G10740, AT2G10690, AT2G10650, AT2G10640, AT2G13100, AT2G01220, AT2G01330, AT2G48060, AT2G48080, AT2G48110, AT2G48020, AT2G48090, AT2G48100, AT2G48030, AT2G48010, AT2G48070, AT2G07340, AT2G07360, AT2G07300, AT2G01210, AT2G01170, AT2G35780, AT2G35730, AT2G35710, AT2G35790, AT2G35680, AT2G35630, AT2G35670, AT2G35733, AT2G18030, AT4G36130, AT2G18020, AT2G18010, AT2G17990, AT2G18100, AT2G18090, AT2G18060, AT2G17930, AT2G17975, AT1G30230, AT2G18110, AT2G18050, AT2G18040, AT2G17972, AT2G17980, AT2G35760, AT2G35650, AT2G35635, AT2G35795, AT2G42400, AT2G14520, AT2G14460, AT2G14560, AT2G14530, AT2G35700, AT2G35690, AT2G35620, AT2G35610, AT2G35770, AT2G35750, AT2G35736, AT2G35720, AT2G42340, AT2G42350, AT2G42360, AT2G42370, AT2G42390, AT2G42430, AT2G42500, AT3G58500, AT2G01720, AT2G01790, AT2G01750, AT2G01740, AT2G01710, AT2G01620, AT2G01650, AT2G01760, AT2G01660, AT2G01680, AT2G01670, AT2G01630, AT2G01640, AT2G01690, AT2G42290, AT2G42450, AT2G42300, AT2G42490, AT2G42310, AT2G42320, AT2G42330, AT2G25620, AT2G25710, AT2G01735, AT2G15050, AT2G15000, AT2G25660, AT2G25690, AT2G25700, AT1G20140, AT2G25730, AT2G25580, AT2G25590, AT2G25640, AT2G25650, AT4G00270, AT2G25720, AT2G25610, AT2G25670, AT2G25570, AT2G25625, AT3G31920, AT2G15090, AT2G15120, AT2G15220, AT2G18480, AT2G18470, AT2G18620, AT2G18600, AT2G18590, AT2G18560, AT2G18570, AT2G18510, AT2G18520, AT2G19560, AT2G19580, AT2G19600, AT2G19620, AT2G19650, AT2G19480, AT2G19490, AT3G32920, AT2G19470, AT2G19570, AT2G19640, AT2G19500, AT2G19520, AT4G29730, AT2G19540, AT2G19590, AT3G46160, AT2G18530, AT2G18500, AT2G37478, AT2G37480, AT2G37260, AT2G37280, AT2G37300, AT2G37250, AT2G37240, AT2G37470, AT2G37400, AT2G37460, AT3G11940, AT2G37270, AT2G37510, AT2G37500, AT2G37220, AT2G37410, AT2G11270, AT2G11140, AT2G18550, AT2G24220, AT2G24230, AT2G24240, AT2G24255, AT2G24260, AT2G24280, AT2G24120, AT2G24130, AT2G24160, AT2G24170, AT2G24140, AT2G24200, AT2G24250, AT2G24270, AT2G24100, AT2G24180, AT2G24150, AT2G37310, AT2G37320, AT2G37330, AT2G37340, AT2G37360, AT2G37380, AT2G37390, AT2G37420, AT2G37430, AT2G37440, AT2G07699, ATMG00410, AT2G07741, ATMG01170, AT2G07670, AT2G07702, ATMG00440, ATMG01140, ATMG00620, ATMG00170, AT2G07722, AT2G24190, AT2G18670, AT2G18690, AT2G18710, AT2G18750, AT2G05910, AT2G05920, AT2G05930, AT2G06000, AT2G05870, AT2G05890, AT2G05830, AT2G06005, AT2G05990, AT2G05840, AT5G35590, AT2G05940, AT2G07680, AT2G07739, ATMG00370, ATMG00290, AT2G07734, AT2G07724, ATMG00200, ATMG00210, AT2G07725, ATMG00220, AT2G07727, AT2G40800, AT2G40780, AT2G40770, AT2G40760, AT2G40730, AT2G40640, AT2G40650, AT2G40810, AT2G40660, AT2G40690, AT2G40765, AT2G18630, AT2G18730, AT2G18760, AT2G18780, AT2G18790, AT2G18800, AT2G18640, AT2G18660, AT2G18680, AT2G18700, AT2G18770, AT2G18740, AT4G38290, AT2G40710, AT2G40700, AT2G40680, AT2G37860, AT2G37840, AT2G37830, AT2G37820, AT2G38060, AT2G37790, AT2G37780, AT2G38010, AT2G38000, AT2G37920, AT2G37990, AT2G37975, AT2G38050, AT2G38020, AT2G37970, AT2G37870, AT2G38040, AT2G37980, AT2G37940, AT2G40720, AT2G45140, AT2G45150, AT2G45070, AT2G45170, AT2G37890, AT2G02080, AT2G02061, AT2G02140, AT2G02180, AT2G02170, AT2G02130, AT2G02100, AT2G02070, AT2G02160, AT2G02090, AT2G45180, AT2G45080, AT2G45050, AT1G45332, AT2G45030, AT2G44990, AT2G44980, AT2G45160, AT2G45110, AT1G65681, AT2G45100, AT2G45060, AT2G45010, AT2G28060, AT2G28120, AT2G02120, AT2G39030, AT2G38905, AT2G12230, AT1G50850, AT2G12250, AT2G12290, AT4G19700, AT2G12190, AT2G28110, AT2G28100, AT2G28070, AT2G28130, AT2G28050, AT2G28190, AT2G28080, AT2G38860, AT2G38870, AT2G39050, AT2G38880, AT2G39010, AT2G02410, AT2G02250, AT2G02300, AT2G02470, AT2G02390, AT2G02370, AT3G54700, AT2G38940, AT2G38950, AT2G38960, AT2G38970, AT2G39000, AT2G39020, AT2G39040, AT2G39080, AT2G39090, AT2G39110, AT2G39120, AT2G38910, AT2G39060, AT2G02400, AT2G02230, AT1G08200, AT2G27860, AT2G27730, AT2G27680, AT2G27760, AT2G27550, AT2G27530, AT2G06820, AT2G06840, AT2G06850, AT2G28810, AT2G28880, AT2G28755, AT2G28760, AT2G28780, AT2G28790, AT2G28800, AT2G28900, AT2G28740, AT2G28870, AT2G28910, AT2G28840, AT2G28830, AT2G02220, AT2G02310, AT2G02340, AT2G02350, AT2G02360, AT2G02380, AT2G29540, AT2G29400, AT2G29530, AT2G29580, AT2G27800, AT5G27300, AT2G27790, AT2G27740, AT2G27700, AT2G27690, AT2G27660, AT2G27650, AT2G27540, AT5G52080, AT2G18320, AT5G53410, AT2G27840, AT2G27810, AT2G27710, AT2G27820, AT2G27720, AT2G27830, AT2G27600, AT2G27580, AT2G27590, AT2G27775, AT2G29340, AT2G29560, AT2G29460, AT2G29510, AT2G29480, AT2G29440, AT2G29600, AT2G29380, AT2G29370, AT2G29330, AT2G29310, AT2G29300, AT2G29260, AT2G29210, AT2G29190, AT2G29180, AT2G29200, AT2G29140, AT2G29390, AT2G29490, AT2G29320, AT2G29350, AT2G29500, AT2G29550, AT2G29420, AT2G29570, AT2G29590, AT2G29450, AT2G01420, AT2G27050, AT2G27060, AT2G27090, AT2G26970, AT2G27000, AT2G27010, AT2G27140, AT2G26990, AT2G27130, AT2G27020, AT2G26980, AT2G27040, AT2G27100, AT2G27080, AT2G27030, AT2G27110, AT3G10280, AT2G46720, AT2G46640, AT2G46660, AT2G46690, AT2G46700, AT2G46710, AT2G46630, AT2G46650, AT2G46680, AT2G01600, AT2G01590, AT2G01530, AT2G01570, AT2G32415, AT2G32410, AT2G32380, AT2G32440, AT2G32270, AT2G32390, AT2G32400, AT2G01480, AT2G01510, AT2G01540, AT2G01550, AT2G01580, AT2G01410, AT2G01430, AT2G01440, AT2G01450, AT2G01460, AT2G01470, AT2G01490, AT2G01610, AT2G01520, AT2G01400, AT2G32300, AT2G32280, AT2G32260, AT2G32250, AT2G32450, AT2G32430, AT2G41250, AT2G41230, AT2G41220, AT2G41420, AT2G41380, AT2G41370, AT2G41350, AT2G41410, AT2G41310, AT2G14650, AT2G14620, AT2G14680, AT2G14660, AT2G32320, AT2G31580, AT2G41290, AT2G43130, AT2G43100, AT2G38790, AT2G38690, AT2G38670, AT2G38660, AT2G38620, AT3G54180, AT2G38780, AT2G38600, AT2G38590, AT2G38580, AT2G38570, AT2G38560, AT2G38770, AT2G38730, AT2G38740, AT2G38700, AT2G38530, AT2G38710, AT2G38550, AT2G38750, AT2G38760, AT2G38610, AT2G38540, AT2G38650, AT2G41340, AT2G41330, AT2G41300, AT2G07140, AT3G44120, AT2G07170, AT2G07180, AT2G07190, AT2G07200, AT2G43160, AT2G43180, AT2G43200, AT2G43235, AT2G43250, AT2G43260, AT2G43150, AT2G43310, AT2G43290, AT2G43210, AT2G43080, AT2G43190, AT2G43090, AT2G43320, AT2G43230, AT2G22770, AT2G22780, AT2G47710, AT2G47970, AT2G47930, AT2G47730, AT2G47770, AT2G47840, AT2G47800, AT2G47750, AT2G47790, AT2G47760, AT2G47960, AT2G31180, AT2G31060, AT2G31110, AT2G31090, AT2G31085, AT2G31170, AT2G31160, AT2G31130, AT2G31070, AT2G47640, AT2G47980, AT2G47630, AT2G47950, AT2G47670, AT2G35190, AT2G07030, AT2G07050, AT2G06980, AT2G06925, AT2G06990, AT2G47720, AT2G47780, AT2G47820, AT2G47830, AT2G47850, AT2G47860, AT2G47940, AT2G47990, AT2G47680, AT2G47690, AT2G47700, AT2G47890, AT2G47900, AT2G35120, AT2G35170, AT2G35155, AT2G23980, AT2G24020, AT2G16920, AT2G16950, AT2G16960, AT2G16970, AT2G16980, AT2G16880, AT2G16890, AT4G35100, AT2G16850, AT2G16940, AT2G16930, AT5G15220, AT2G16900, AT2G16860, AT2G35230, AT2G35110, AT2G35100, AT2G35290, AT2G35270, AT2G35320, AT2G35150, AT2G46330, AT2G46170, AT2G46290, AT2G46280, AT2G46270, AT2G46230, AT2G46080, AT2G23950, AT2G23960, AT2G23970, AT2G23990, AT2G24040, AT2G24050, AT2G24070, AT2G24090, AT2G23830, AT2G23890, AT2G24060, AT2G23940, AT2G23945, AT2G23910, AT2G23930, AT2G46260, AT2G46140, AT2G46090, AT2G46220, AT2G14920, AT1G13430, AT2G14880, AT2G14890, AT2G46250, AT2G46240, AT2G46225, AT2G46210, AT2G46200, AT2G46180, AT2G46150, AT2G46130, AT2G46110, AT2G46070, AT2G46060, AT2G46030, AT2G46020, AT2G46310, AT2G35510, AT2G14960, AT2G14860, AT2G14900, AT2G14910, AT2G29720, AT2G35660, AT2G29680, AT2G29670, AT2G35480, AT2G35460, AT2G35450, AT2G35390, AT1G32380, AT2G35380, AT2G35360, AT2G35340, AT2G35330, AT2G35590, AT2G35540, AT2G35530, AT2G35470, AT2G35370, AT2G35490, AT2G35520, AT2G35585, AT2G35605, AT2G35410, AT2G29730, AT2G29710, AT2G29660, AT2G29700, AT2G25840, AT2G25810, AT2G25800, AT2G25760, AT2G25740, AT2G25880, AT2G25900, AT2G25910, AT2G25735, AT2G25850, AT2G25920, AT2G25830, AT2G25790, AT2G29780, AT2G29760, AT2G29750, AT2G29740, AT2G29690, AT2G29650, AT2G29630, AT2G29620, AT2G25870, AT2G03270, AT2G19720, AT2G19710, AT2G19670, AT2G19660, AT2G19930, AT2G19900, AT2G19960, AT2G19800, AT2G19790, AT2G19810, AT2G19680, AT2G19830, AT2G19730, AT2G19740, AT2G19760, AT2G19860, AT2G19880, AT2G19940, AT2G19750, AT2G03200, AT2G03120, AT2G46850, AT2G46740, AT2G46750, AT2G46760, AT2G46780, AT2G46820, AT2G46735, AT2G46800, AT2G46830, AT2G46790, AT2G46670, AT2G03220, AT2G03170, AT2G03150, AT2G03140, AT2G03260, AT2G03240, AT2G03280, AT2G46900, AT2G46930, AT2G47140, AT2G47010, AT2G03030, AT2G03300, AT2G03050, AT2G02990, AT2G02970, AT2G02950, AT2G02930, AT4G02520, AT2G02870, AT2G47060, AT2G47020, AT2G47000, AT2G46990, AT2G46950, AT2G46940, AT2G46920, AT2G47130, AT2G47070, AT2G46910, AT2G47110, AT3G62250, AT1G18930, AT3G29440, AT3G44390, AT4G12426, AT3G47165, AT5G33315, AT5G26590, AT3G42883, AT1G12935, AT5G28466, AT2G03080, AT3G50490, AT2G04140, AT2G04130, AT5G56605, AT4G17820, AT4G20180, AT2G03090, AT2G03010, AT4G16530, AT4G16540, AT2G03020, AT2G29050, AT2G29060, AT2G29070, AT2G29080, AT2G29110, AT2G28940, AT2G29130, AT2G28960, AT2G28970, AT2G28980, AT2G28990, AT2G29020, AT2G28950, AT2G02960, AT2G02860, AT2G02980, AT2G03070, AT2G02880, AT2G22840, AT2G22850, AT2G22860, AT2G22880, AT2G22870, AT2G22900, AT2G28930, AT2G29960, AT2G30010, AT2G30000, AT2G29920, AT2G29995, AT2G29970, AT2G44850, AT2G44830, AT2G44920, AT2G44910, AT2G44900, AT2G44970, AT2G44950, AT2G44870, AT2G44940, AT2G44840, AT2G44860, AT2G22800, AT2G22890, AT2G22910, AT2G22920, AT2G22930, AT2G22830, AT2G30040, AT3G27140, AT4G08260, AT2G30020, AT2G29990, AT2G26000, AT2G25990, AT2G25980, AT2G25930, AT2G26150, AT2G26140, AT2G26135, AT2G26110, AT2G25970, AT2G26060, AT2G25950, AT2G25940, AT2G26850, AT2G26820, AT2G26810, AT2G26940, AT2G26920, AT2G26910, AT2G26900, AT2G26890, AT2G26870, AT2G26830, AT2G26930, AT2G26800, AT2G29980, AT2G45770, AT2G44630, AT2G44660, AT2G44710, AT2G44730, AT2G44740, AT2G44760, AT2G44800, AT2G44820, AT2G44770, AT2G44680, AT2G44670, AT2G44790, AT2G44610, AT2G44650, AT2G44750, AT1G02880, AT2G44640, AT2G44620, AT2G44690, AT2G26080, AT2G26070, AT2G26040, AT2G45810, AT2G45820, AT2G45990, AT2G34600, AT2G34570, AT2G34590, AT2G34560, AT2G34585, AT2G34650, AT2G45920, AT2G45910, AT2G45890, AT2G45880, AT2G45870, AT2G45860, AT2G45830, AT2G45750, AT2G45970, AT2G45950, AT3G61415, AT2G45980, AT2G45730, AT2G45740, AT2G46000, AT2G45960, AT2G45790, AT2G45850, AT1G63250, AT2G07750, AT2G18900, AT2G18915, AT2G18950, AT2G18960, AT2G18980, AT2G18990, AT2G18820, AT2G18890, AT2G18850, AT4G30260, AT2G18840, AT2G18910, AT2G18940, AT2G34555, AT2G34540, AT2G34530, AT2G34520, AT2G34510, AT2G34640, AT2G34630, AT2G07690, AT2G39435, AT2G39450, AT2G39480, AT2G39510, AT2G39530, AT2G39360, AT2G39540, AT2G39560, AT2G39580, AT2G39620, AT2G39370, AT2G39380, AT2G39410, AT2G39420, AT2G39430, AT2G39470, AT2G39390, AT2G39460, AT2G39500, AT2G39400, AT2G39570, AT2G39550, AT2G39630, AT2G39280, AT2G39310, AT2G39330, AT2G39190, AT2G39290, AT2G39200, AT2G39210, AT2G34500, AT2G34490, AT2G34310, AT2G34470, AT2G34430, AT2G34420, AT2G34340, AT2G34260, AT2G34460, AT2G34190, AT2G34480, AT2G39350, AT2G39260, AT2G39230, AT2G39220, AT2G39180, AT2G39170, AT2G39340, AT2G39160, AT2G39140, AT2G39130, AT2G39320, AT2G39300, AT2G34230, AT2G34250, AT2G34390, AT2G29870, AT2G38340, AT2G38330, AT2G38470, AT2G38460, AT2G38440, AT2G38420, AT2G38490, AT2G38450, AT2G38310, AT2G38400, AT2G38465, AT2G38320, AT2G38480, AT2G34300, AT2G34315, AT2G34357, AT2G34180, AT2G34200, AT2G41060, AT2G38410, AT2G38380, AT2G38390, AT2G38370, AT2G38360, AT2G32500, AT2G32510, AT2G32520, AT2G32580, AT2G32480, AT2G32600, AT2G41120, AT2G41110, AT2G41080, AT2G41040, AT2G41020, AT2G41010, AT2G41000, AT2G40980, AT2G41160, AT2G41070, AT2G41090, AT2G40970, AT2G40960, AT2G41200, AT2G41190, AT2G41210, AT2G41140, AT2G41100, AT2G41180, AT2G32620, AT2G38140, AT2G38300, AT2G38270, AT2G38185, AT2G38120, AT2G38170, AT2G38080, AT2G38280, AT2G38180, AT2G41460, AT2G41480, AT2G41500, AT2G41490, AT2G41430, AT2G14720, AT2G14740, AT2G14800, AT2G14750, AT2G14850, AT2G14835, AT2G32560, AT2G32590, AT2G32610, AT2G23530, AT2G23540, AT2G23560, AT2G23600, AT2G23590, AT2G23610, AT2G23620, AT2G23380, AT2G23390, AT2G23420, AT2G23450, AT2G23430, AT2G23460, AT2G23410, AT2G38230, AT2G38210, AT2G38250, AT2G38090, AT2G38290, AT2G38100, AT2G38110, AT2G38130, AT2G38160, AT2G38240, AT2G30970, AT2G31040, AT2G30860, AT2G30870, AT2G37630, AT2G37650, AT2G37670, AT2G37690, AT2G05140, AT2G37520, AT2G37710, AT2G37740, AT2G37540, AT2G37585, AT2G37590, AT2G37660, AT2G37560, AT2G37600, AT2G37620, AT3G53750, AT2G37730, AT2G37580, AT2G37750, AT2G37550, AT2G37570, AT2G37760, AT2G23360, AT2G44020, AT2G44150, AT2G43950, AT2G44090, AT3G59970, AT2G44160, AT2G44050, AT2G44180, AT2G44170, AT2G44110, AT2G30890, AT2G31300, AT2G30910, AT2G30950, AT2G30960, AT2G30810, AT2G30990, AT2G31010, AT2G31020, AT2G31030, AT2G30840, AT2G30980, AT2G30930, AT2G30920, AT2G44065, AT2G44060, AT2G44120, AT2G43970, AT2G02630, AT2G02620, AT2G02610, AT2G02590, AT2G02500, AT2G02480, AT2G43880, AT2G43980, AT2G43990, AT2G44010, AT5G38640, AT2G44070, AT2G44080, AT2G44190, AT2G43910, AT2G43920, AT2G44100, AT2G44040, AT3G59890, AT2G44130, AT2G02510, AT2G02680, AT2G02640, AT2G30250, AT2G30170, AT2G30120, AT2G23070, AT2G23030, AT2G23000, AT2G23010, AT2G23080, AT2G23090, AT2G23120, AT2G22990, AT2G23110, AT2G22970, AT2G22980, AT2G23150, AT2G02580, AT2G02570, AT2G02560, AT2G30160, AT2G30130, AT2G30050, AT2G19350, AT2G19460, AT2G19450, AT2G19390, AT2G23700, AT2G23690, AT2G23630, AT2G23770, AT2G23740, AT2G23810, AT2G23660, AT2G23780, AT2G23760, AT2G30150, AT2G30140, AT2G30110, AT2G30100, AT2G30080, AT2G30070, AT2G30060, AT2G30210, AT2G30200, AT2G19385, AT2G34680, AT2G34670, AT2G34750, AT2G34710, AT2G34770, AT2G34660, AT2G19340, AT2G19380, AT2G19270, AT2G19280, AT2G19430, AT2G19440, AT1G64760, AT2G19310, AT2G39980, AT2G39990, AT2G39870, AT2G34690, AT2G44200, AT2G44520, AT2G44260, AT2G44230, AT2G39940, AT2G39950, AT2G40010, AT2G40030, AT2G40070, AT2G39880, AT2G39900, AT2G39930, AT2G40000, AT2G39890, AT2G40020, AT2G40060, AT2G39910, AT2G39970, AT2G40080, AT2G44530, AT2G44310, AT2G44210, AT2G44350, AT2G44420, AT2G44525, AT2G44510, AT2G26290, AT2G26280, AT2G26230, AT2G26300, AT2G26250, AT2G26200, AT2G26340, AT2G26170, AT2G26190, AT2G44300, AT2G44360, AT2G44370, AT2G44380, AT2G44430, AT2G44440, AT2G44450, AT2G44470, AT2G44480, AT2G44490, AT2G44500, AT2G44270, AT1G76170, AT2G26240, AT2G26210, AT2G26180, AT2G33740, AT2G34780, AT2G34910, AT2G34920, AT2G34930, AT2G34940, AT2G34790, AT2G34970, AT2G34980, AT2G35000, AT2G35010, AT2G35030, AT2G35040, AT2G35050, AT2G35060, AT2G34810, AT2G34830, AT2G34840, AT2G34850, AT2G34860, AT2G35020, AT2G26350, AT2G26260, AT2G33800, AT2G19200, AT2G19060, AT2G19070, AT2G19080, AT2G19180, AT2G33680, AT2G33840, AT1G28350, AT2G33700, AT2G33710, AT2G33860, AT2G33880, AT2G33730, AT2G33760, AT2G33790, AT2G33670, AT2G33850, AT2G33810, AT2G33830, AT2G33735, AT2G33845, AT2G19150, AT2G19170, AT2G19010, AT2G30580, AT2G30530, AT2G30590, AT2G30490, AT2G30390, AT2G02850, AT2G02740, AT2G02730, AT2G02710, AT2G02780, AT2G02770, AT3G11470, AT2G02810, AT2G02760, AT2G02820, AT2G02800, AT2G19110, AT2G19120, AT4G30100, AT2G19130, AT2G30620, AT2G30400, AT2G30410, AT2G30550, AT2G45720, AT2G45500, AT2G45510, AT2G44890, AT2G45540, AT2G45550, AT2G45530, AT2G45560, AT2G45520, AT2G45710, AT2G45700, AT2G45660, AT2G45640, AT2G45670, AT2G45690, AT2G45620, AT2G45680, AT2G30520, AT2G30500, AT2G30470, AT2G30460, AT2G30480, AT2G30600, AT2G30560, AT2G30570, AT2G45490, AT2G41890, AT2G42040, AT2G42070, AT2G41900, AT2G42010, AT2G41880, AT2G42030, AT2G32760, AT2G32750, AT2G32740, AT2G32730, AT2G32720, AT2G32670, AT2G32660, AT2G32800, AT2G32180, AT2G32650, AT2G32765, AT2G32810, AT2G32710, AT2G32700, AT2G45580, AT2G45590, AT2G39670, AT2G30740, AT2G30750, AT2G30770, AT2G30780, AT2G30790, AT2G30800, AT2G30660, AT2G30670, AT2G30680, AT2G30640, AT2G30710, AT2G30695, AT2G41990, AT2G41980, AT2G41970, AT2G41960, AT2G41950, AT2G42080, AT2G42060, AT2G39700, AT2G39705, AT2G39710, AT2G33000, AT2G32990, AT2G33060, AT2G33050, AT2G33020, AT2G32850, AT2G33040, AT2G32980, AT2G32970, AT2G32900, AT2G32930, AT2G32920, AT2G26640, AT2G26740, AT2G26750, AT2G26770, AT2G26780, AT2G26650, AT2G26660, AT2G26690, AT2G26700, AT2G26710, AT2G26680, AT2G26670, AT2G26760, AT2G26730, AT2G39650, AT2G39660, AT2G42240, AT2G42250, AT2G42270, AT2G42280, AT2G42120, AT2G42130, AT2G42150, AT2G42160, AT2G42110, AT2G42190, AT2G42210, AT2G32950, AT2G32910, AT2G32860, AT2G26580, AT2G23180, AT2G39850, AT2G45380, AT2G45350, AT2G45190, AT2G45270, AT1G67790, AT4G25530, AT4G17245, AT4G14400, AT4G15210, AT4G16515, AT4G15680, AT4G17470, AT4G16870, AT4G16880, AT4G16900, AT4G16040, AT4G15620, AT4G15660, AT4G15690, AT4G15700, AT4G15530, AT4G14540, AT4G14480, AT4G14080, AT4G14090, AT1G56650, AT1G56710, AT5G04230, AT5G04190, AT5G04140, AT5G04400, AT1G51080, AT1G51110, AT1G66920, AT1G66910, AT1G35180, AT1G35170, AT1G45474, AT1G26210, AT1G57850, AT1G57830, AT1G57790, AT1G58430, AT5G11050, AT1G26220, AT5G11080, AT5G13170, AT5G20740, AT5G20630, AT5G21100, AT5G28870, AT5G20140, AT5G20935, AT4G36470, AT4G36600, AT1G31920, AT1G77390, AT1G57770, AT5G16920, AT5G17490, AT5G16030, AT5G15780, AT5G15840, AT5G15720, AT5G15800, AT5G14870, AT1G31690, AT1G29720, AT1G31720, AT5G33340, AT5G33370, AT5G34790, AT5G28030, AT5G28090, AT5G27560, AT5G27440, AT5G27200, AT5G25840, AT5G25860, AT5G25130, AT5G24930, AT5G67020, AT5G67180, AT5G66740, AT5G66340, AT5G66500, AT5G66550, AT5G65890, AT5G65730, AT5G65410, AT5G65070, AT5G65080, AT5G64770, AT5G64800, AT5G63900, AT5G63630, AT5G63180, AT5G62840, AT5G62080, AT5G62140, AT5G60910, AT5G61290, AT5G60970, AT5G60010, AT5G59660, AT5G59670, AT5G59810, AT5G59130, AT5G58830, AT5G58390, AT5G58310, AT5G58260, AT5G58120, AT5G57760, AT5G57780, AT5G57570, AT5G57180, AT5G57250, AT5G56780, AT5G56850, AT5G56860, AT5G56490, AT5G55930, AT5G55450, AT5G55580, AT5G55720, AT5G55470, AT5G55320, AT5G54740, AT5G54250, AT5G53190, AT5G52970, AT5G53030, AT5G52780, AT5G52290, AT5G51950, AT5G51850, AT5G51720, AT5G52100, AT5G51030, AT5G50770, AT5G50790, AT5G50645, AT5G50540, AT5G49330, AT5G48210, AT5G47610, AT5G47900, AT5G47380, AT5G47330, AT5G46690, AT5G46260, AT5G46490, AT5G46660, AT5G46830, AT5G46450, AT5G46240, AT5G46330, AT5G46440, AT5G45820, AT5G45830, AT5G45930, AT5G45950, AT5G45310, AT5G45040, AT5G45230, AT5G44680, AT5G44620, AT5G44740, AT5G44870, AT5G43800, AT5G43840, AT5G43200, AT5G42680, AT5G41720, AT5G41780, AT5G41870, AT5G41900, AT5G41320, AT5G40380, AT5G39980, AT5G39210, AT5G39030, AT5G39080, AT5G38870, AT5G38390, AT5G38510, AT5G38520, AT5G38780, AT5G38130, AT5G37300, AT5G36990, AT5G36910, AT5G37080, AT5G35480, AT5G35490, AT5G24420, AT5G23970, AT5G24210, AT5G23660, AT5G23270, AT5G22460, AT5G22390, AT5G22430, AT5G22380, AT5G19170, AT5G18600, AT5G18060, AT5G18430, AT5G17320, AT5G15310, AT5G13730, AT5G13270, AT5G12000, AT5G11610, AT5G10420, AT5G10760, AT5G10250, AT5G09780, AT5G09660, AT5G09640, AT5G09930, AT5G08230, AT5G07240, AT5G07230, AT5G07540, AT5G07180, AT5G07060, AT5G06870, AT5G06790, AT5G05840, AT5G04660, AT5G03810, AT5G03820, AT5G03620, AT5G03120, AT5G03350, AT5G02920, AT5G02940, AT5G02340, AT5G02330, AT5G02460, AT5G01180, AT3G63160, AT3G63060, AT3G62950, AT3G62410, AT3G62550, AT3G61700, AT3G61040, AT3G60790, AT3G60290, AT3G59580, AT3G59060, AT3G59140, AT3G59030, AT3G59180, AT3G59400, AT3G58860, AT3G58070, AT3G57180, AT3G57240, AT3G56260, AT3G55710, AT3G55330, AT3G55130, AT3G55500, AT3G54050, AT3G53720, AT3G53800, AT3G53680, AT3G53170, AT3G53140, AT3G53310, AT3G53320, AT3G52720, AT3G53040, AT3G52540, AT3G52130, AT3G51420, AT3G51400, AT3G50820, AT3G51150, AT3G50570, AT3G50440, AT3G50270, AT3G49900, AT3G49300, AT3G49260, AT3G49270, AT3G48720, AT3G48420, AT3G47760, AT3G47750, AT3G47430, AT3G46780, AT3G47090, AT3G47580, AT3G46530, AT3G46770, AT3G46570, AT3G45860, AT3G45110, AT3G44780, AT3G44020, AT3G43630, AT3G43880, AT3G43290, AT3G42700, AT3G42260, AT3G42460, AT3G42140, AT4G39710, AT4G39970, AT4G39410, AT4G38690, AT4G38760, AT4G38860, AT4G38850, AT4G37980, AT4G37770, AT4G36220, AT4G37360, AT4G36105, AT4G35710, AT4G35290, AT4G34770, AT4G34940, AT4G34850, AT4G34440, AT4G33280, AT4G32980, AT4G32340, AT4G32540, AT4G31805, AT4G31630, AT4G30720, AT4G30110, AT4G29200, AT4G28780, AT4G29030, AT4G28680, AT4G28500, AT4G28160, AT4G28000, AT4G28080, AT4G27240, AT4G26920, AT4G26950, AT4G26520, AT4G26530, AT4G26660, AT4G26150, AT4G26170, AT4G25780, AT4G25920, AT4G25980, AT4G25990, AT4G25420, AT4G25020, AT4G25000, AT4G24710, AT4G24540, AT4G24660, AT4G24630, AT4G24700, AT4G23930, AT4G23990, AT4G24010, AT4G23780, AT4G23600, AT4G23240, AT4G23290, AT4G23300, AT4G23220, AT4G23140, AT4G23160, AT4G22870, AT4G22880, AT4G22830, AT4G22200, AT4G22440, AT4G21370, AT4G21210, AT4G20370, AT4G20760, AT4G20200, AT4G19530, AT4G19170, AT4G19380, AT4G18960, AT4G18970, AT4G19095, AT4G18375, AT4G18810, AT4G18290, AT4G13810, AT4G13840, AT4G13410, AT4G13450, AT4G12830, AT4G12970, AT4G12410, AT4G12280, AT4G12290, AT4G11900, AT4G11180, AT4G11100, AT4G11460, AT4G10940, AT4G10950, AT4G10540, AT4G10010, AT4G08990, AT4G14140, AT4G09010, AT4G09350, AT4G09390, AT4G08710, AT4G08830, AT4G07740, AT4G05270, AT4G04730, AT4G04900, AT4G04280, AT4G04330, AT4G03010, AT4G02420, AT4G01460, AT4G01130, AT4G00970, AT4G00310, AT4G00180, AT1G32080, AT1G16730, AT1G19670, AT4G10150, AT4G10160, AT2G33500, AT2G40500, AT2G40560, AT1G66980, AT1G22370, AT1G34070, AT1G19210, AT1G19150, AT1G07180, AT1G07050, AT1G06980, AT1G20750, AT1G20810, AT1G13650, AT1G51805, AT1G51670, AT1G30160, AT1G69500, AT1G69523, AT1G66460, AT1G66830, AT1G66840, AT1G66850, AT3G06160, AT3G11090, AT3G11110, AT1G75240, AT1G32560, AT1G31550, AT1G75250, AT1G66130, AT1G66100, AT3G19550, AT3G28220, AT3G28770, AT3G14760, AT3G28290, AT3G28300, AT3G28310, AT3G28270, AT3G24460, AT3G28920, AT3G18840, AT3G18870, AT3G11980, AT3G52310, AT2G34090, AT3G25670, AT3G13770, AT3G13740, AT3G22150, AT3G22121, AT3G15110, AT3G26310, AT3G26320, AT3G21870, AT3G24630, AT3G24650, AT3G29590, AT3G13390, AT3G13420, AT3G26932, AT3G13540, AT3G28630, AT3G19620, AT3G19270, AT3G15270, AT3G30460, AT3G20210, AT3G28890, AT3G24710, AT3G23770, AT3G23760, AT3G13180, AT3G13220, AT3G27920, AT3G27840, AT3G21950, AT3G22020, AT3G15030, AT3G14450, AT3G15570, AT3G30220, AT3G30250, AT1G34967, AT5G17125, AT1G34904, AT2G26020, AT2G20875, AT1G78840, AT4G00260, AT2G24650, AT2G17000, AT1G29440, AT1G43000, AT3G14490, AT3G23950, AT3G23970, AT3G11000, AT3G26160, AT3G26180, AT3G26170, AT3G26280, AT3G20300, AT3G27440, AT3G26960, AT3G13065, AT3G42570, AT3G17070, AT3G21790, AT3G19800, AT3G29030, AT3G19480, AT3G16250, AT3G25880, AT3G14700, AT3G13900, AT3G13960, AT3G15840, AT3G27750, AT3G27690, AT3G26870, AT3G22710, AT3G15440, AT3G15510, AT3G15400, AT3G17640, AT3G16670, AT3G01015, AT3G04210, AT3G04290, AT3G04450, AT3G08660, AT3G09580, AT3G04650, AT3G05630, AT3G05730, AT3G10620, AT3G10150, AT3G10600, AT3G10570, AT3G08870, AT3G08920, AT3G01960, AT3G03480, AT3G07450, AT3G07520, AT3G04980, AT3G04960, AT3G02310, AT3G02150, AT3G01500, AT3G01490, AT3G01550, AT3G09080, AT3G03840, AT3G03830, AT1G69120, AT1G69160, AT1G17700, AT1G01530, AT1G13790, AT1G12480, AT1G12570, AT1G12460, AT1G21320, AT1G20390, AT1G35310, AT1G27990, AT1G28160, AT1G52410, AT1G47980, AT1G56430, AT1G68890, AT1G55370, AT1G63150, AT1G77490, AT1G71160, AT1G64360, AT1G29500, AT1G29510, AT1G29450, AT1G29460, AT1G29430, AT5G27780, AT1G72260, AT1G49880, AT1G66220, AT1G66230, AT1G52190, AT1G75040, AT1G76420, AT1G76470, AT1G67860, AT1G68780, AT1G73710, AT1G73700, AT1G63240, AT1G52770, AT1G52830, AT1G52890, AT1G70700, AT1G74580, AT1G74620, AT1G74630, AT1G74390, AT1G63710, AT1G73870, AT1G74070, AT1G69720, AT1G69730, AT1G72460, AT1G68190, AT1G10920, AT1G51440, AT2G43530, AT2G43520, AT1G62320, AT1G19510, AT1G32320, AT1G49150, AT1G43740, AT1G43750, AT1G06830, AT1G43810, AT1G21500, AT1G02670, AT1G21540, AT1G26540, AT1G26500, AT1G26600, AT1G17420, AT1G01390, AT1G01250, AT1G07485, AT1G62940, AT1G75460, AT1G19640, AT1G19650, AT1G34480, AT1G04800, AT1G20020, AT1G48470, AT1G79770, AT1G41870, AT1G18810, AT1G18730, AT1G18710, AT1G14440, AT1G71720, AT1G01150, AT1G49650, AT1G49975, AT1G18485, AT1G15580, AT1G76100, AT1G76110, AT1G15980, AT1G30455, AT1G15960, AT1G50530, AT1G65860, AT1G65870, AT1G22590, AT1G64680, AT1G33811, AT1G33730, AT1G33720, AT1G80080, AT1G56150, AT1G02820, AT1G02813, AT1G59680, AT1G02830, AT1G77990, AT1G52530, AT1G52510, AT1G74880, AT1G77870, AT1G74890, AT1G68600, AT1G70985, AT1G70820, AT1G64260, AT1G49490, AT1G50280, AT1G50180, AT1G11220, AT1G11340, AT1G21810, AT1G11300, AT1G34245, AT1G34310, AT1G15180, AT1G15260, AT1G75880, AT1G62720, AT1G75960, AT1G43590, AT1G13140, AT1G10710, AT1G14680, AT1G14840, AT1G64710, AT1G64690, AT1G64900, AT1G14760, AT1G59720, AT1G65800, AT1G65790, AT1G75790, AT1G75770, AT1G75690, AT1G24020, AT2G05390, AT2G16010, AT1G65190, AT1G65250, AT1G78450, AT1G65230, AT1G54160, AT1G54020, AT1G03020, AT1G54040, AT1G05540, AT1G10585, AT2G36145, AT2G04270, AT2G15260, AT2G04039, AT2G28640, AT2G31770, AT2G42610, AT2G21650, AT2G17040, AT2G04820, AT2G04790, AT1G04420, AT1G31100, AT2G20570, AT1G60060, AT2G21280, AT2G21370, AT2G46360, AT2G04570, AT2G04460, AT2G04420, AT2G21770, AT2G36560, AT2G36040, AT2G36000, AT2G35820, AT2G42800, AT2G13020, AT2G21140, AT2G21210, AT2G21200, AT2G03710, AT1G78995, AT1G78970, AT2G13680, AT1G78980, AT1G02205, AT1G65450, AT1G02190, AT1G22630, AT1G60430, AT1G60230, AT1G67450, AT1G54820, AT1G62030, AT1G11850, AT2G25220, AT1G61800, AT1G27480, AT1G09530, AT1G09500, AT1G10060, AT1G30790, AT1G05230, AT1G04645, AT1G65620, AT1G65480, AT1G09890, AT1G70110, AT1G62050, AT2G17910, AT1G24260, AT1G23070, AT1G23130, AT1G60590, AT1G77110, AT1G67400, AT3G43400, AT1G24400, AT1G24470, AT1G24520, AT1G52000, AT1G52040, AT1G52030, AT1G55550, AT1G03940, AT1G03495, AT1G63360, AT1G62630, AT1G23730, AT1G23790, AT2G36750, AT2G36800, AT2G36790, AT2G36630, AT2G28420, AT2G42990, AT2G42940, AT2G20380, AT2G14060, AT2G20250, AT2G18300, AT2G13350, AT2G10940, AT2G20700, AT2G15740, AT2G27420, AT2G03550, AT2G35740, AT2G15020, AT2G15042, AT2G25680, AT2G15080, AT2G37450, AT2G31720, AT2G18810, AT2G40670, AT2G37900, AT2G02450, AT2G39100, AT2G06890, AT2G27520, AT2G29410, AT2G29290, AT2G29360, AT2G32290, AT2G14595, AT2G14610, AT2G47910, AT2G06960, AT2G47880, AT2G16840, AT2G16910, AT2G35310, AT2G35260, AT2G23840, AT2G35500, AT2G35600, AT2G19780, AT2G03310, AT2G46870, AT2G29090, AT2G29120, AT2G44930, AT2G22810, AT2G29940, AT2G34620, AT2G45900, AT2G34450, AT2G39250, AT2G32540, AT2G32530, AT2G23550, AT2G23470, AT2G30925, AT2G37640, AT2G37720, AT2G30820, AT2G44030, AT2G43900, AT2G23130, AT2G02540, AT2G02520, AT2G23670, AT2G39920, AT2G26330, AT2G44460, AT2G34960, AT2G34800, AT2G19050, AT2G30540, AT2G45600, AT2G45630, AT2G41940, AT2G41870, AT2G32690, AT2G32680, AT2G32640, AT2G30690, AT2G39640, AT2G39690, AT2G42220, AT2G42200, AT2G32880, AT2G32870
