# Supplementary material for: Deep-sequencing transcriptome analysis of chilling tolerance mechanisms of a subnival alpine plant, Chorispora bungeana
Source: BMC Plant Biol. 2012 Nov 21;12:222. doi: 10.1186/1471-2229-12-222 (PMC3571968; doi:10.1186/1471-2229-12-222)

**1. Primers for reference gene selection**

| Primer Name | Sequence | Product Len |
| --- | --- | --- |
| qACTIN2F: | TGACACCATGATGTCTCGGTCTAC | 72bp |
| qACTIN2R: | GTTTTGCCGGTGACGATGC |  |
| qCBT10307F: | CCAATGTCCTACGCGAGTTTTC | 65bp |
| qCBT10307R: | TTTCCGAGGGTTCTTACCCAGT |  |
| qCBT10872F: | CACTTAAAGCAAACGCCAAGTTC | 107bp |
| qCBT10872R: | GCCGCATTTCATTGCGTTCT |  |
| qCBT12464F: | TGGGATAAACTGCCCCATTGT | 77bp |
| qCBT12464R: | AGAAACCAGTCATACGCAAGAATC |  |
| qCBT16484F | CAAAGATTGCAGGATTCGCAT | 131bp |
| qCBT16484R | TTCTCATGATGCCGACGGTAA |  |
| qCBT27997F: | CCCAGTCAGACCAGAGCGTA | 87bp |
| qCBT27997R: | GAAGACATGGACATGCACGAA |  |
| qCBT28565F: | TCAACCTCCATCTCGGACTCA | 159bp |
| qCBT28565R: | TGGATACAACGGACGTTACAACA |  |
| qCBT32485F: | TCCTTATGTCCGCTACTGTTAATTC | 90bp |
| qCBT32485R: | CCGGGTGTGTTCGTCCTTTAG |  |

**2. Primers for qPCR verification**

| Primer Name | Sequence | Product Len |
| --- | --- | --- |
| qCBT11719F: | AATCGTGGAGTGGCCAGTTG | 69bp |
| qCBT11719R: | CCCATAATACGGCGACCTTTG |  |
| qCBT1251F: | TCGTCGACGGTGGGTATGA | 87bp |
| qCBT1251R: | CACCACGTCGTTGGTCGAT |  |
| qCBT13319F: | CGAGGAACCGCACGATGTA | 93bp |
| qCBT13319R: | CCACGTGTCTCCCCATTCAA |  |
| qCBT13614F: | TACTATGCGTTCCCTAAGGGAGT | 70bp |
| qCBT13614R: | CGGAATAAGCCGGTTTTCGACT |  |
| qCBT13817F: | AAGCCAACGCTGGGTGTCT | 70bp |
| qCBT13817R: | CAGGAGCTGTTCTCAGTGGGAT |  |
| qCBT15934F: | TTCTCCTCCTAAGGCCTTCATC | 95bp |
| qCBT15934R: | AAGAAGTGAGAAAACTGTGCGAGA |  |
| qCBT19519F: | CACTCGATTCCTTGTATAAGGCTCT | 97bp |
| qCBT19519R: | GGAAGTGTGGCTGGAGAATTTG |  |
| qCBT22504F: | CCGTAGGGGAAATAAGTCACTTTG | 86bp |
| qCBT22504R: | GACTCAAAGCATGCAGCGAA |  |
| qCBT22708F: | ACTTCGCGTATGGAGCCTTC | 75bp |
| qCBT22708R: | TCAAGCCTACAAACAATTCGTCA |  |
| qCBT22908F: | CCGGACACGAAATCGCAGT | 73bp |
| qCBT22908R: | AGCAAGCCGCCTCCAATG |  |
| qCBT25137F: | GGCGACCGTGGTTGACAGT | 75bp |
| qCBT25137R: | GTCGGAAGGCTGCCTGTCT |  |
| qCBT45404F: | CCCCACGGATTCCGATCAT | 78bp |
| qCBT45404R: | TTTTCGAGAGATCGGTGAATGA |  |
| qCBT47699F: | CCTCCTTCTAGTGGCATATGCTCT | 101bp |
| qCBT47699R: | TCTGCCCTTCCAGCGCTCA |  |
| qCBT4773F: | GTGGCTCGAACTATGAGGTTTAC | 65bp |
| qCBT4773R: | ATCTGTCAAAGCCGCACTAAC |  |
| qCBT47787F: | TTTCCACTGACTGAAACCAATTCT | 106bp |
| qCBT47787R: | ACGATTACAGAATCCCTGCTGTT |  |
| qCBT47948F: | ATCCATCGGCGCTTGACAT | 133bp |
| qCBT47948R: | TTGCTAGTCATGAAAGCTCTGTCT |  |
| qCBT52238F: | CGGGAGAATCGTAATTACGAACA | 156bp |
| qCBT52238R: | TTTGGCGATCGGATCTCAAAC |  |
| qCBT52823F: | ATCCTTGGGTATTCACATCAAGC | 149bp |
| qCBT52823R: | GTTTAACGATGCCGTTGCCA |  |
| qCBT6902F: | TCGGCATAAGCGACGACAA | 61bp |
| qCBT6902R: | AACTCCGCCTTCGCTGCTA |  |
| qCBT7920F: | AGTCGGAGCGGGTATTTGAA | 93bp |
| qCBT7920R: | TCCGGGTCATGGGTTTACTAG |  |

**3. Unigenes for qPCR reference gene selection**

| **Unigene** | **AGI model** | **Functional Description** | **% identity** | **log2 ratio (RNA-seq** | **q-value** |
| --- | --- | --- | --- | --- | --- |
| CBT7542 | AT3G18780.2 | actin 2 (ACT2) | 100 | -0.769 | 0.000 |
| CBT10307 | AT5G49720.1* | glycosyl hydrolase 9A1 (GH9A1) | 93.4 | 0.147 | 0.000 |
| CBT10872 | AT3G60800.1 | DHHC-type zinc finger family protein | 98.68 | -0.003 | 0.990 |
| CBT12464 | AT2G44160.1 | methylenetetrahydrofolate reductase 2 (MTHFR2) | 96.5 | 0.001 | 0.988 |
| CBT16484 | AT2G28390.1* | SAND family protein | 89.67 | -0.119 | 0.061 |
| CBT27997 | AT1G09770.1 | cell division cycle 5 (CDC5) | 92.59 | -0.002 | 0.945 |
| CBT28565 | AT5G27630.1* | acyl-CoA binding protein 5 (ACBP5) | 84.15 | -0.024 | 0.926 |
| CBT32485 | AT1G58060.1* | RNA helicase family protein | 74.56 | 0.048 | 0.932 |

*Paper recommended reference genes.

**4. geNorm results of reference gene selection**


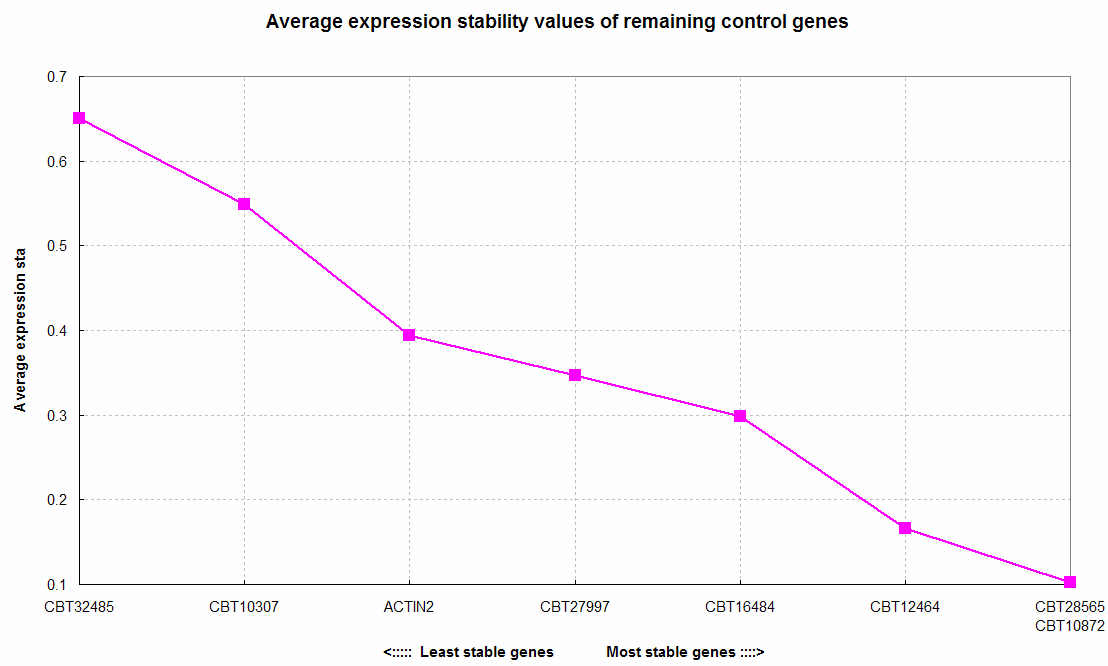


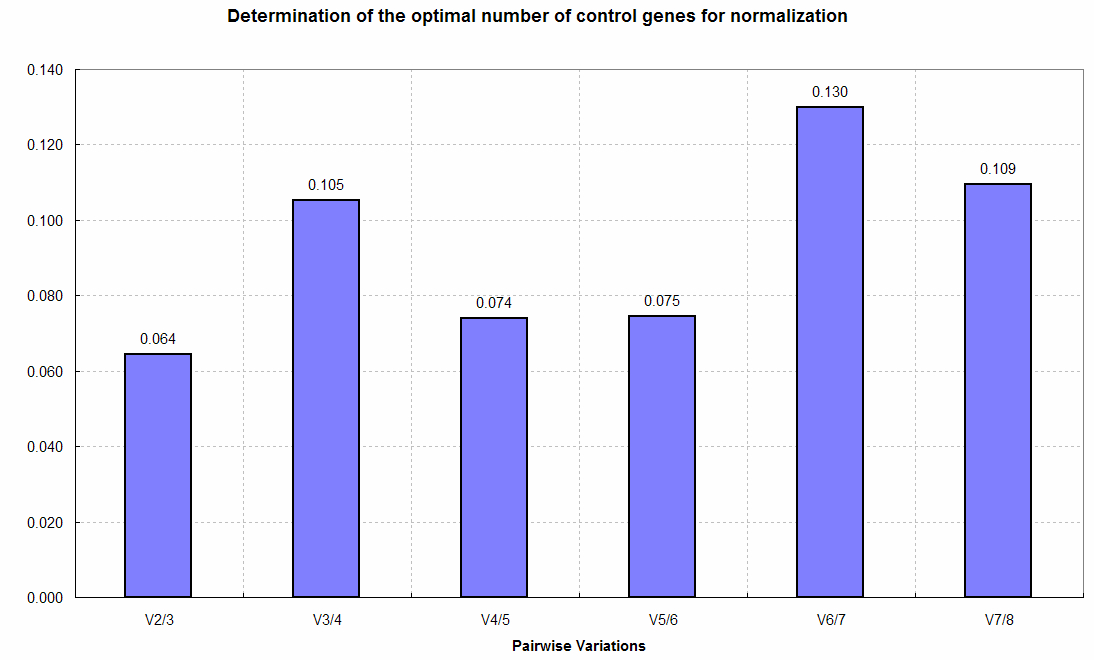

Supplement: Additional file 8 — Primers and reference gene selections. 1. Primers for reference gene selection. 2. Primers for qPCR verification. 3. Unigenes for qPCR reference gene selection. 4. geNorm results of reference gene selection. [file 1471-2229-12-222-S8.docx]
